# Gold(I) Catalysis Applied to the Stereoselective Synthesis of Indeno[2,1-*b*]thiochromene Derivatives and Seleno Analogues

**DOI:** 10.1021/acs.orglett.2c03411

**Published:** 2022-10-24

**Authors:** Cintia Virumbrales, Mahmoud A. E. A. A. A. El-Remaily, Samuel Suárez-Pantiga, Manuel A. Fernández-Rodríguez, Félix Rodríguez, Roberto Sanz

**Affiliations:** † Área de Química Orgánica, Departamento de Química, Facultad de Ciencias, Universidad de Burgos, Pza. Misael Bañuelos s/n, 09001 Burgos, Spain; ⊥ Chemistry Department, Faculty of Science, Sohag University, 82524 Sohag, Egypt; ∇ Facultad de Farmacia, Departamento de Química Orgánica y Química Inorgánica, Instituto de Investigación Química “Andrés M. del Río” (IQAR), Campus Científico-Tecnológico, Universidad de Alcalá (IRYCIS), Autovía A-II, Km 33.1, 28805 Alcalá de Henares, Spain; ‡ Departamento de Química Orgánica e Inorgánica, Facultad de Química, Universidad de Oviedo, C/Julián Clavería, 8, 33006 Oviedo, Spain

## Abstract

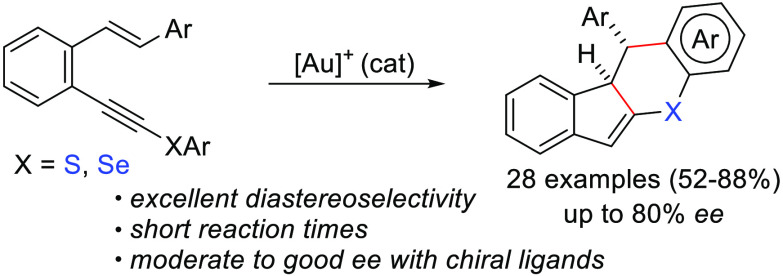

A gold(I)-catalyzed cascade reaction for the stereoselective synthesis of sulfur- or selenium-containing indeno[1,2-*b*]chromene derivatives from *o*-(alkynyl)styrenes substituted at the triple bond with a thio- or seleno-aryl group is described. The reaction involves a double cyclization process through a proposed key gold–cyclopropyl carbene intermediate that evolves by the intramolecular addition of an aromatic to the cyclopropane ring, affording polycyclic structures. The enantioselective version was studied using gold(I) complexes bearing chiral ligands.

S- and Se-containing heterocyclic compounds have received increased attention due to their unique chemical, physical, and biological properties.^1^ The presence of these heteroatoms results in substantial alterations of the cyclic structure. Moreover, their size and electronegativity and the availability of unshared electrons lead to heterocycles with particular characteristics that find applications in fields such as medicine and materials science.^2^ On the other hand, gold catalysis has become one of the most valuable tools for the straightforward synthesis of (hetero)cyclic compounds.^3^ Thus, the π-activation of alkynes by gold complexes toward intramolecular attack by nucleophiles is nowadays a common strategy for constructing cyclic molecules from acyclic compounds. In particular, the use of olefins as the internal nucleophiles has been extensively considered and therefore a wide number of synthetically useful Au-catalyzed cycloisomerization reactions of enyne derivatives have been described.^4^ In this context, we have reported practical methods for the synthesis of 1*H*-indenes and benzofulvenes from appropriately substituted *o*-(alkynyl)styrenes.^5^ These reactions proceed through the initial activation of the alkyne by the coordination of the gold catalyst. The subsequent reaction of the alkene with the activated alkyne through a 5-*endo*-*dig* pathway leads to a cyclopropyl gold carbene derivative,^6^ which can also be represented as a gold-containing indene derivative with an exocyclic carbocation.^7^ This species evolves in the presence of an external nucleophile to give the final 1*H*-indene derivatives (Scheme 1a).

**Scheme 1:**
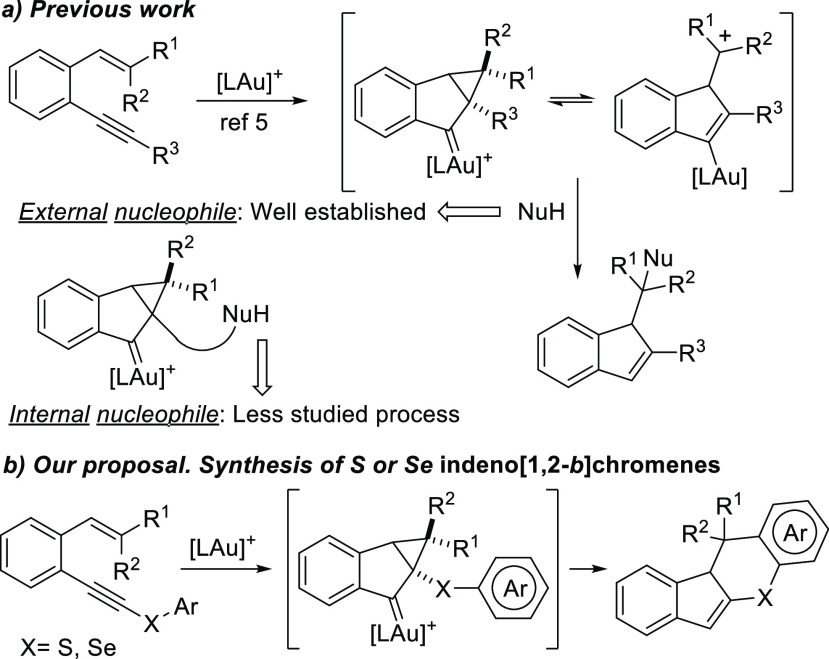
Previous Results in the Au(I)-Catalyzed Reaction of *o*-(Alkynyl)styrenes and Present Work

Although the Au-catalyzed reaction of *o*-(alkynyl)styrenes with external nucleophiles has been studied extensively,^5,8^ investigations of the intramolecular version where the cyclopropyl gold carbene intermediate reacts with an internal nucleophile are scarce.^9^ In fact, the intramolecular reaction with C-based nucleophiles was not considered before. With all this in mind, we envisioned that unique S- or Se-containing heterocyclic compounds could be obtained from *o*-(alkynyl)styrenes substituted at the triple bond with a thio- or seleno-aryl group, respectively (Scheme 1b). The importance of S- and Se-heterocycles along with the easy availability of the starting materials encouraged us to attempt the synthesis of potentially useful indeno[1,2-*b*]thiochromene derivatives, or their seleno-analogues, through a gold-catalyzed reaction. This unprecedented reaction would proceed through a cascade process involving enyne cycloisomerization and subsequent intramolecular Friedel–Crafts-type cyclization. Apart from proving the reactivity of the cyclopropyl gold carbene intermediate with an internal nucleophile, the intriguing stereochemical issues of the proposal were another motivation to work on the project.

As noted above, the starting materials necessary to test our hypothesis are readily available. For example, we prepared *o*-(alkynyl)styrene **3a**, functionalized with a thioaryl group at the terminal position of the alkyne, from readily available 2-(trimethylsilylethynyl)benzaldehyde (**1a**) by a straightforward sequence involving a Wadsworth–Emmons reaction followed by the deprotection of the alkyne, to give enyne derivative **2a**, and further thiolation to provide **3a** (Scheme 2).^10^

**Scheme 2:**
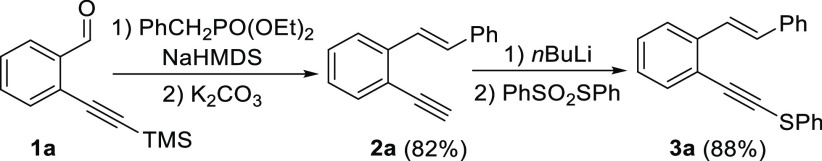
Preparation of (*E*)-**3a**

With *o*-(alkynyl)styrene (*E*)-**3a** in hand, we tried our planned reaction. Gratifyingly, when **3a** was treated with different gold complexes (2.5 mol %) in DCM at room temperature, the desired indeno[1,2-*b*]thiochromene **4a** was obtained in a high yield after a short reaction time (30 min, Table 1). Interestingly, the final product was obtained as a single isomer in all attempts. As IPrAuNTf_2_ is a stable, easy-to-handle complex that does not require a silver salt cocatalyst, it was selected for the subsequent experiments.

**Table 1:**
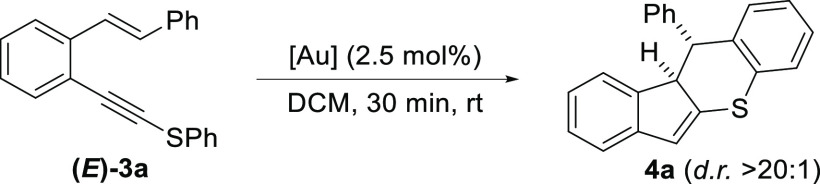
Gold-Catalyzed Synthesis of Dihydroindeno[2,1-*b*]thiochromene **4a** from (*E*)-**3a**^a^

entry	catalyst [Au]	yield (%)^b^
1	[3,5-(*t*-Bu)_2_C_6_H_3_O_3_]_3_PAuCl/AgNTf_2_	83
2	IPrAuCl/AgSbF_6_	84
3	IPrAuCl/AgNTf_2_	85
4	Ph_3_PAuNTf_2_	71
5	XPhosAuNTf_2_	76
6	IPrAuNTf_2_	89 (81)

a Reaction conditions are as follows: **3a** (0.1 mmol) and catalyst (2.5 mol %) in DCM (0.4 mL) at rt for 30 min. XPhos = dicyclohexyl[2′,4′,6′-tris(propan-2-yl)[1,1′-biphenyl]-2-yl]phosphane. IPr = 1,3-bis(2,6-diisopropylphenyl)imidazol-2-ylidene.

b Determined by ^1^H NMR analysis of the crude mixture using CH_2_Br_2_ (1 M) as an internal standard. Isolated yield is shown in parentheses.

To evaluate the scope of the process, a representative set of S- and Se-substituted *o*-(alkynyl)styrenes **3** and **5** was subjected to the optimized reaction conditions (Table 2). As shown, the expected indeno[1,2-*b*]thiochromene derivatives **4** were isolated in high yields and as single diastereoisomers in all cases. Different aryl substituents were allowed at the alkene moiety (R^3^). Regarding the aromatic ring on the S or Se atom (Ar), nonfunctionalized rings (entries 1–7), aryl groups substituted with electron-donating groups (entries 8 and 9), and halogen-substituted aryl groups (entries 10–14) were allowed. The reaction was also performed with *o*-(alkynyl)styrenes substituted at the aromatic ring core (entries 15–17). Interestingly, the Se-containing products **6** (entries 18–21) were obtained as efficiently as their S-analogues. The structures and relative configurations of the stereogenic centers of these compounds were determined by NMR experiments and unambiguously confirmed by X-ray analysis of **4m**.^11^

**Table 2:**
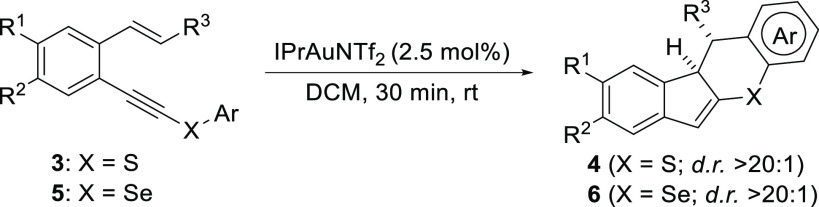
Synthesis of Dihydroindeno[2,1-*b*]thiochromenes **4** and Dihydroindeno[2,1-*b*]selenochromenes **6** from *o*-(Alkynyl)styrenes (*E*)-**3** and (*E*)-**5**^a^

entry	**3** or **5**	R^1^	R^2^	R^3^	Ar	**4** or **6**	yield (%)^b^
1	**3a**	H	H	Ph	Ph	**4a**	81
2	**3b**	H	H	4-MeC_6_H_4_	Ph	**4b**	82
3	**3c**	H	H	4-(MeO)C_6_H_4_	Ph	**4c**	88
4	**3d**	H	H	4-ClC_6_H_4_	Ph	**4d**	80
5	**3e**	H	H	2,6-F_2_C_6_H_3_	Ph	**4e**	87
6	**3f**	H	H	Ph	4-MeC_6_H_4_	**4f**	85
7	**3g**	H	H	Ph	1-naphthyl	**4g**	83
8	**3h**	H	H	Ph	4-(MeO)C_6_H_4_	**4h**	87
9	**3i**	H	H	Ph	3-(MeO)C_6_H_4_	**4i**^c^	77
10	**3j**	H	H	Ph	4-ClC_6_H_4_	**4j**	82
11	**3k**	H	H	Ph	4-BrC_6_H_4_	**4k**	88
12	**3l**	H	H	Ph	2-ClC_6_H_4_	**4l**	83
13	**3m**	H	H	Ph	2-FC_6_H_4_	**4m**	78
14	**3n**	H	H	Ph	4-FC_6_H_4_	**4n**	79
15	**3o**	F	H	Ph	Ph	**4o**	77
16	**3p**	–OCH_2_O–		Ph	Ph	**4p**	80
17	**3q**	–OCH_2_O–		Ph	4-ClC_6_H_4_	**4q**	76
18	**5a**	Η	Η	Ph	Ph	**6a**	86
19	**5b**	Η	Η	4-MeC_6_H_4_	Ph	**6b**	79
20	**5c**	Η	Η	4-(MeO)C_6_H_4_	Ph	**6c**	88
21	**5d**	Η	Η	4-ClC_6_H_4_	Ph	**6d**	70

a Reaction conditions are as follows: **3** or **5** (0.3 mmol) and IPrAuNf_2_ (2.5 mol %) in DCM (1.2 mL) at rt for 30 min.

b Isolated yield referred to the corresponding starting *o*-(alkynyl)styrene **3** or **5**.

c Obtained as a 6:1 mixture of regioisomers.

Apart from these reactions conducted with *o*-(alkynyl)styrenes **3** with the *E*-configuration at the C=C bond, we also performed some experiments from starting materials with the *Z*-configuration, i.e., (*Z*)**-3** (Table 3). These reactions worked perfectly, and the corresponding products **4** were obtained in high yields; surprisingly, however, mixtures of the two possible diastereoisomers (**4**/*diast***-4**) were observed. Only when the aromatic ring at the alkene (Ar) was electronically rich (*p*-methoxyphenyl) was a single diastereoisomer (**4c**) observed (entry 4). Interestingly, the structure of this isomer matches that of the product previously obtained with (*E*)-**3c** (see Table 2, entry 3).^12^

**Table 3:**
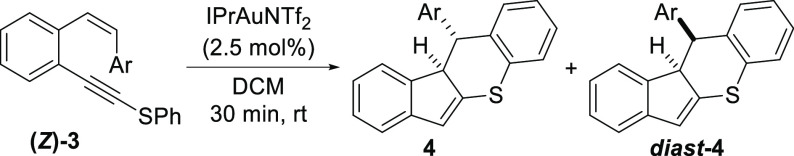
Synthesis of Dihydroindeno[2,1-*b*]thiochromenes **4** and *diast*-**4** from (*Z*)-**3**^a^

entry	(*Z*)-**3**	Ar	product	dr^b^	yield (%)^c^
1	**3a**	Ph	**4a/***diast***-4a**	1:2	83
2^d^	**3e**	2,6-F_2_C_6_H_3_	**4f/***diast***-4f**	1:1.6	73
3^d^	**3d**	4-ClC_6_H_4_	**4d/***diast***-4d**	4:1	75
4	**3c**	4-MeOC_6_H_4_	**4c**	>20:1	88

a Reaction conditions are as follows: **3** (0.3 mmol) and IPrAuNf_2_ (2.5 mol %) in DCM (1.2 mL) at rt for 30 min.

b Diastereoisomeric ratio of **4** determined by ^1^H NMR analysis of the crude reaction mixture.

c Isolated yield refers to the corresponding starting *o*-(alkynyl)styrene **3**.

d Reaction time of 10 min.

We also performed some experiments with *o*-(alkynyl)styrenes **3r**–**w**, which were fully substituted at the terminal position of the alkene (Scheme 3). As shown, when the alkene was substituted with two phenyl groups (**3r**–**t**), the expected products **4r**–**t** were isolated in high yields. Next, we tried the reaction with **3u** and **3v** bearing two methyl groups. Thus, when the aromatic ring linked to the sulfur atom (Ar) was a simple phenyl group (**3u**), we only isolated the indene **7u**.^5c^ However, the formation of the expected indeno[1,2-*b*]thiochromene derivative occurred when an electronically richer aromatic ring was used (**3v**, Ar = 3,5-(MeO)_2_C_6_H_3_), although even in this case indene **7v** was isolated along with major product **4v**. Finally, we carried out the reaction with *o*-(alkynyl)styrene **3w** containing a phenyl group and a methyl group at the terminal position of the alkene. In this case, we observed the exclusive formation of the indeno [1,2-*b*]thiochromene **4w** in a high yield. It should be noted that although the starting material was a 2:1 mixture of *E*/*Z* isomers, the final product **4w** was obtained as a single diastereoisomer. The structure and relative configuration of the stereogenic centers of this compound were unambiguously determined by X-ray analysis.^11^

**Scheme 3:**
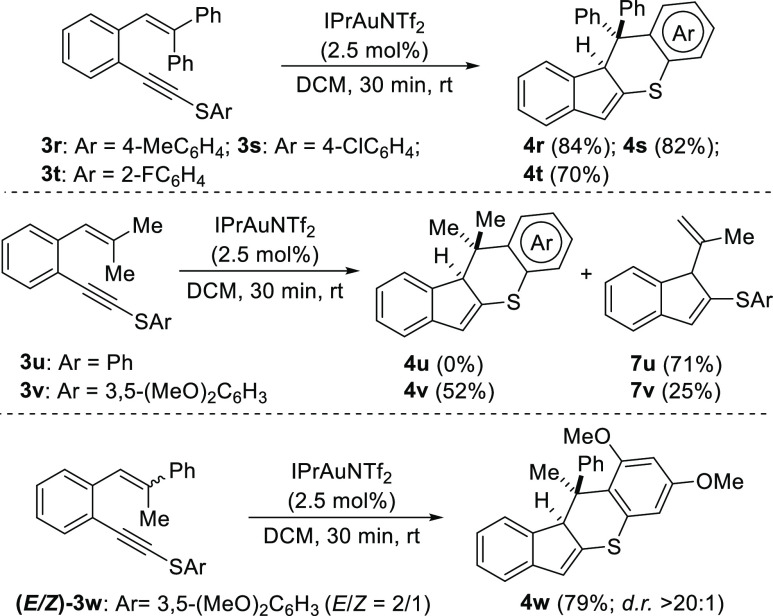
Cycloisomerization of β,β-Disubstituted *o*-(Alkynyl)styrenes **3r**–**w**

Finally, the enantioselective version of this new cascade reaction was attempted (Table 4).^10^ When the reaction was performed with the gold(I) complex containing (*S*)-DM-SEGPHOS as a chiral ligand in DCE at −10 ^ο^C, the corresponding final indeno[1,2-*b*]thiochromenes **4** were isolated in high yields with moderate to high enantioselectivities.

**Table 4:**
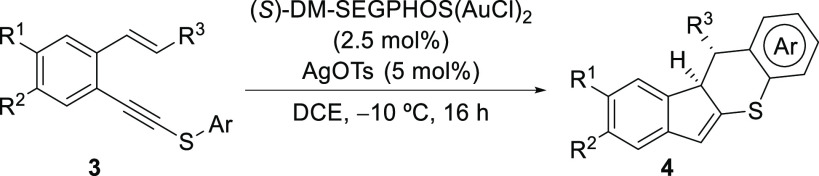
Gold(I)-Catalyzed Enantioselective Synthesis of Dihydroindeno[2,1-*b*]thiochromenes **4** from (*E*)-**3**^a^

entry	**3**	R^1^	R^2^	R^3^	Ar	**4**	yield (%)^b^	er^c^
1	**3a**	H	H	Ph	Ph	**4a**	89	90:10
2	**3b**	H	H	4-MeC_6_H_4_	Ph	**4b**	84	86:14
3	**3d**	H	H	4-ClC_6_H_4_	Ph	**4d**	85	78:22
4	**3j**	H	H	Ph	4-ClC_6_H_4_	**4j**	87	88:12
5	**3o**	F	H	Ph	Ph	**4o**	74	72:28
6	**3p**	–OCH_2_O–		Ph	Ph	**4p**	88	84:16

a Reactions conditions are as follows: **3** (0.3 mmol), (*S*)-DM-SEGPHOS(AuCl)_2_ (2.5 mol %), and AgOTs (5 mol %) in DCE (1.2 mL) at −10 ^ο^C for 16 h.

b Yield of isolated **4** based on **3**.

c Determined by HPLC on a chiral stationary phase using a Chiralpak AD-H column (*n*-hexane/*i*-PrOH eluent).

A plausible mechanism that explains the formation of indeno[1,2-*b*]chromene derivatives **4** from *o*-(alkynyl)styrenes **3** is shown in Scheme 4a (for simplicity, the reaction of **3a** is taken as model). Thus, the initial coordination of the catalyst to the alkyne generates the first intermediate **I**. This coordination favors the reaction with the alkene through a 5-*endo*-*dig* pathway to form the cyclopropyl gold carbene derivative **II** in a stereospecific manner. The addition of the electron-rich phenylthio group to the cyclopropyl ring and the subsequent ring opening of the cyclopropane yield the cationic species **III**. This intermediate evolves through a rearomatization process followed by protodemetalation to give the final product **4a** as a single diastereoisomer.

**Scheme 4:**
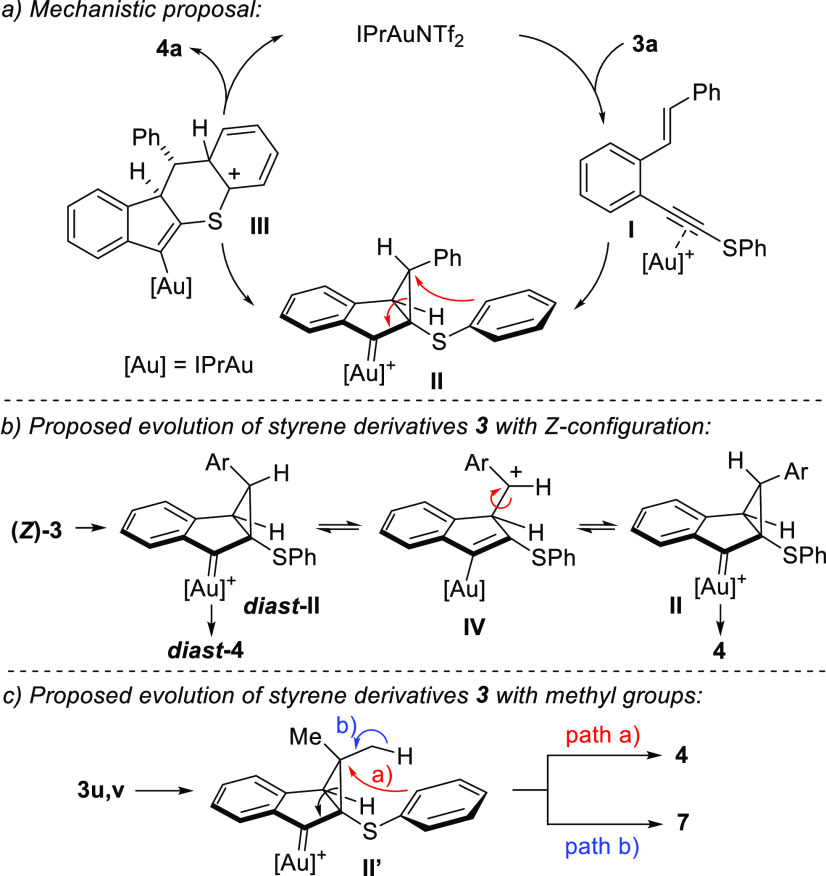
Mechanistic Proposal

As previously noted, when the starting *o*-(alkynyl)styrenes **3** have the *Z*-configuration, the reaction usually leads to the formation of corresponding indeno[1,2-*b*]chromene derivatives as mixtures of diastereoisomers **4**/*diast***-4** (see Table 3). This fact can be explained by the pathway shown in Scheme 4b. In this way, the initial cycloisomerization of (*Z*)**-3** leads to cyclopropane derivative *diast***-II**, the precursor of *diast***-4**. It should be noted that the aryl group in the cyclopropane derivative *diast***-II** points to the same face where the bulky gold atom (with its ligand) is placed. The ring opening of *diast***-II** to give the cationic species **IV** and subsequent cyclization lead to the sterically less crowded cyclopropyl gold carbene **II**, which is the precursor of **4**. It seems that in those cases where the aromatic ring at the terminal position of the alkene (Ar in Scheme 4b) is electronically rich (for example, in (*Z*)**-3c** with a 4-methoxyphenyl group), the interconversion of *diast***-II** to **II** is fast and, finally, a single diastereoisomer (**4c**) is obtained. In this case, the cyclopropane ring opening in *diast***-IIc** is probably a particularly favored process because a relatively stable cationic intermediate **IVc** is formed. Subsequent evolution of **IVc** to the most favored cyclopropyl gold carbene **IIc** should occur before the intramolecular reaction with the aryl thio group. It has also been shown that variable amounts of indene derivatives **7** are formed when *o*-(alkynyl)styrene derivatives **3u** and **3v** substituted with methyl groups are used (Scheme 3). The generation of these products can be accounted as shown in Scheme 4c. Thus, apart from the usual evolution of cyclopropyl gold carbene **II′** to give indeno[1,2-*b*]chromene derivatives such as **4v** (pathway a), an alternative route consists of the simple ring opening of the cyclopropane favored by an elimination reaction (pathway b) to render indene derivatives **7u** and **7v**.

In conclusion, we have reported a simple method for the synthesis of S- or Se-containing indeno[1,2-*b*]chromene derivatives from readily available starting materials that involves a double-cyclization process. More precisely, we have found that simple *o*-(alkynyl)styrenes substituted at the triple bond with a thio- or seleno-aryl group react in the presence of a gold(I) catalyst through a cascade process that involves the initial formation of a cyclopropyl gold carbene intermediate, followed by a cyclopropane ring opening promoted by the intramolecular addition of the arene from the thio- or seleno-aryl group. The stereoselectivity of the process is determined by a key gold–cyclopropyl carbene intermediate, which controls the attack of the aromatic ring. This work further expands the utility of gold catalysis to access unusual complex heterocyclic compounds from easily available starting materials. The possibility of performing the reaction in an enantioselective way using a chiral gold(I) catalyst is demonstrated.

## Notes

The data underlying this study are available in the published article and its online Supporting Information.

## Supporting Information Available

Experimental details, characterization data, X-ray crystallographic data for **4m** and **4w**, and copies of NMR spectra (PDF)
FAIR data, including the primary NMR FID files, for compounds (*E*)-**2a**–**e**, (*E*)-**2o**, (*E*)-**2p**, (*Z*)-**2a**, (*Z*)-**2d**, (*Z*)-**2e**, **2r**, **2u**, **2w**, (*E*)-**3a**–**q**, (*Z*)-**3a**, (*Z*)-**3c**–**e**, **3r**–**t**, **3v**–**w**, **4a**–**t**, **4v**–**w**, ***diast*-4d**, (*E*)-**5a**–**d**, and **6a**–**d** (ZIP)
