# Supplementary material for: Gold(I) Catalysis Applied to the Stereoselective Synthesis of Indeno[2,1-b]thiochromene Derivatives and Seleno Analogues
Source: Org Lett. 2022 Oct 24;24(43):8077–82. doi: 10.1021/acs.orglett.2c03411 (PMC9641678; doi:10.1021/acs.orglett.2c03411)

Supporting Information

Gold(I)-Catalysis Applied to the Stereoselective Synthesis of Indeno[2,1-*b*]thiochromene Derivatives and Seleno-Analogues

Cintia Virumbrales,† Mahmoud A. E. A. A. A. El-Remaily,ϛ Samuel Suárez-Pantiga,† Manuel A. Fernández-Rodríguez,Ŧ Félix Rodríguez,‡ and Roberto Sanz†*

†Área de Química Orgánica, Departamento de Química, Facultad de Ciencias, Universidad de Burgos, Pza. Misael Bañuelos s/n, 09001-Burgos, Spain

e-mail: rsd@ubu.es

ϛChemistry Department, Faculty of Science, Sohag University, 82524 Sohag, Egypt

ŦUniversidad de Alcalá (IRYCIS). Departamento de Química Orgánica y Química Inorgánica, Instituto de Investigación Química "Andrés M. del Río" (IQAR). Campus Científico-Tecnológico, Facultad de Farmacia. Autovía A-II, Km 33.1, 28805-Alcalá de Henares, Madrid, Spain

‡Departamento de Química Orgánica e Inorgánica, Facultad de Química, Universidad de Oviedo, C/ Julián Clavería, 8, 33006 Oviedo, Spain

Table of Contents

Experimental Procedures S2
- General information........ S2
- Synthesis of *o*-(ethynyl)styrenes **(*E*)-2a-e,o,p** S2
- Synthesis of *o*-(ethynyl)styrenes **(*E*)-2r,u,w** S3
- Synthesis of *o*-(ethynyl)styrenes **(*Z*)-2** S3
- Synthesis of *o*-(alkynyl)styrenes **3** S4
- Synthesis of *o*-(alkynyl)styrenes **5** S4
- Synthesis of dihydroindeno[2,1-*b*]thio(seleno)chromenes **4**,**6** S5
- Synthesis of disubstituted dihydroindeno[2,1-*b*]thiochromenes **4r-w** S5
- Synthesis of dihydroindeno[2,1-*b*]thiochromene **4d** (1 mmol scale) S5

Characterization Data S6

Enantioselective Synthesis of Selected Dihydroindeno[2,1-*b*]thiochromenes **4** S22
- Optimization of the reaction conditions for the enantioselective synthesis of **4a** S22
- Enantioselective synthesis of selected thiochromenes **4** S24
- Chiral HPLC-traces S24

X-Ray Crystallographic Data for **4m** S30

X-Ray Crystallographic Data for **4w** S31

^{1}H and ^{13}C Spectra of Characterized Compounds S32

Experimental Procedures

General information:

All reactions involving air-sensitive compounds were carried out under a N_2 atmosphere in oven-dried glassware with magnetic stirring. Temperatures are reported as bath temperatures. Solvents used for extraction and purification were distilled prior to use. TLC was performed on alumina-backed plates coated with silica gel 60 with F254 indicator. The chromatograms were visualized by UV light (254 nm) and R_f values refer to silica gel. Flash column chromatography was carried out on silica gel 60, 230−400 mesh. ^{1}H NMR spectra were recorded at 300 or 400 MHz. Chemical shifts are reported in ppm with the residual solvent resonance as the internal standard ($CDCl_3$: δ 7.26; C_6D_6: δ 7.16). Data are reported as follows: chemical shift, multiplicity (s: singlet, d: doublet, dd: doublet of doublets, ddd: doublet of doublet of doublets, td: triplet of doublets, t: triplet, dq: doublet of quartets, sex: sextet, sep: septet, m: multiplet), coupling constants (*J* in Hz) and integration. ^{13}C NMR spectra were recorded at 75.4 or 100.6 MHz using broadband proton decoupling. Chemical shifts are reported in ppm with the solvent resonance as internal standard ($CDCl_3$: δ 77.16; C_6D_6: δ 128.06). Carbon multiplicities were assigned by DEPT techniques. Gas chromatography–mass spectra (GC-MS) were recorded on an instrument equipped with a 30 m × 0.25 mm capillary apolar column (stationary phase: 5% diphenyldimethylpolysiloxane film, 0.25 μm). Low-resolution electron impact mass spectra (EI-LRMS) were obtained at 70 eV and only the molecular ions and/or base peaks as well as significant peaks in MS are given. High-resolution mass spectra (HRMS) were recorded on an instrument equipped with a magnetic sector ion analyzer using EI at 70 eV or on an instrument equipped with a QTOF analyzer using ESI (+). Melting points were measured on a microscopic apparatus using open capillary tubes and are uncorrected. All commercially available reagents were used without purification unless otherwise indicated and were purchased from standard chemical suppliers.

Synthesis of *o*-(ethynyl)styrenes (*E*)-2a-e,o,p:

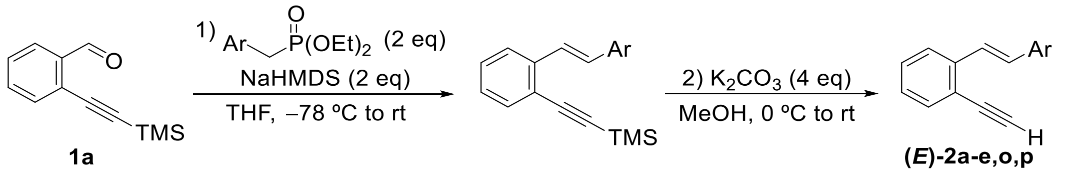

1a **(*E*)-2a-e,o,p**

Step 1: NaHMDS (10 mmol, 10 mL, 1 M in hexanes, 2 eq) was added to a solution of the appropriate diethyl phosphonate (10 mmol, 2.0 mL, 2 eq) in THF (20 mL, 0.25 M) at −78 ºC and the resulting mixture was stirred for 30 min at rt.[1] After cooling to −50 ºC, the 2-alkynylbenzaldehyde derivative[2] (5 mmol, 1 eq) was added to the solution, and the reaction was stirred at rt until the aldehyde was consumed as determined by GC-MS (overnight). The crude was partitioned between aqueous NH_4Cl/DCM and the aqueous layer further extracted with DCM (2 × 20 mL). The organic phase was dried over anhydrous Na_2SO_4 and the solvents were removed under reduced pressure. The residue was purified by flash column chromatography using a mixture of hexane/EtOAc as eluent to obtain the corresponding *o*-(alkynyl)styrene as almost pure *E*-isomers which are used in the next step (not reported).

Step 2: To a solution of the corresponding *o*-(trimethylsilyl)alkynyl styrene derivative (3 mmol) in MeOH (10 mL, 0.3 M) a 0 ºC was added K_2CO_3 (1.65 g, 12 mmol, 4 eq). The resulting mixture was

[1] Bera, K.; Schneider, C. *Chem. Eur. J.* **2016**, *22*, 7074–7078.

[2] Sanjuán, A. M.; Rashid, M. A.; García-García, P.; Martínez-Cuezva, A.; Fernández-Rodríguez, M. A.; Rodríguez, F.; Sanz, R. *Chem. Eur. J.* **2015**, *21*, 3042–3052.

stirred at rt during 1 h. Then crude was partitioned between aqueous NH_4Cl/DCM and the aqueous layer further extracted with DCM (2 × 10 mL). The organic phase was dried over anhydrous Na_2SO_4 and the solvents were removed under reduced pressure. The residue was purified by flash column chromatography using a mixture of hexane/EtOAc as eluent to obtain the corresponding *o*-(ethynyl)styrenes as *E*-isomers **2a-e,o,p**.

Synthesis of *o*-(ethynyl)styrenes 2r,u,w:

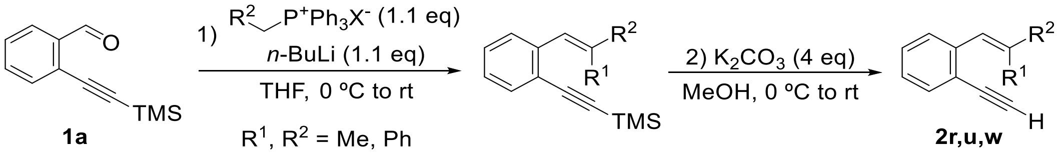

Step 1: To a solution of the corresponding phosphonium salt (5.5 mmol, 1.1 eq) in THF (10 mL) was added dropwise a solution of *n*-BuLi in hexane (5.5 mmol, 6.8 mL of a 1.6 M solution in hexane, 1.1 eq) under nitrogen at 0 ºC. After stirring at rt for 30 min, the reaction was cooled to 0 ºC and 2-alkynylbenzaldehyde derivative **1a**[2] (1.01 g, 5 mmol, 1 eq) was added to the solution. The resulting mixture was stirred until complete consumption of the starting material. Then, water (10 mL) was added to the mixture and the aqueous layer was extracted with diethyl ether (2 × 20 mL). The organic phase was washed with saturated brine solution, dried over anhydrous Na_2SO_4 and the solvents were removed under reduced pressure. The residue was purified by flash column chromatography on silica gel, eluting with a mixture of hexane/EtOAc to obtain the corresponding *o*-(trimethylsilyl)alkynyl styrene derivative which were used in the next step (not reported).
Step 3: To a solution of the corresponding *o*-(trimethylsilyl)alkynyl styrene derivative (3 mmol, 1 eq) in MeOH (10 mL, 0.3 M) at 0 ºC was added K_2CO_3 (1.65 g, 12 mmol, 4 eq). The resulting mixture was stirred for 1 h at rt. Then, crude was partitioned between aqueous NH_4Cl/DCM and the aqueous layer further extracted with DCM (2 × 10 mL). The organic phase was dried over anhydrous Na_2SO_4 and the solvents were removed under reduce pressure. The residue was purified by flash column chromatography using a mixture of hexane/EtOAc as eluent to obtain the corresponding *o*-(ethynyl)styrenes **2r,u,w**.

Synthesis of *o*-(ethynyl)styrenes (*Z*)-2:

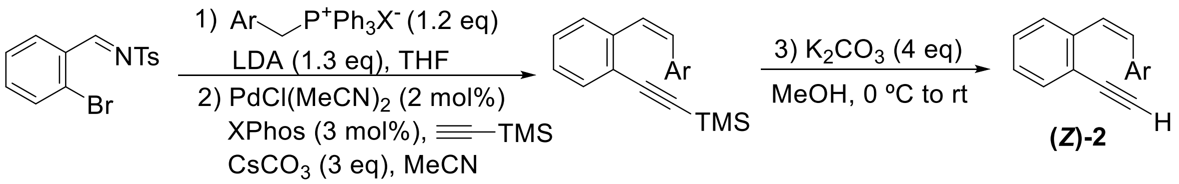

Step 1: To a stirred solution of diisopropylamine (0.97 mL, 6.5 mmol, 1.3 eq) in THF (5.0 mL) under nitrogen at –78 ºC was added dropwise *n*-BuLi (2.6 mL of a 2.5 M solution in hexane, 6.5 mmol, 1.3 eq). The mixture was stirred for 1 h and the corresponding benzyltriphenylphosphonium chloride (6 mmol) was added. The resulting mixture was stirred for 1 h and *N*-(2-bromobenzylidene)-4-methylbenzenesulfonamide[3] (1.68 g, 5 mmol, 1 eq) was added. The resulting mixture was stirred at –78 ºC for 3 h, warmed slowly to rt and stirred at rt for additional 4 h. Water (5.0 mL) was added and the organic phase was extracted with hexane (2 × 20 mL), dried over anhydrous Na_2SO_4, and the solvents

[3] Dong, D.-J.; Li, H.-H.; Tian, S.-K. *J. Am. Chem. Soc.* **2010**, *132*, 5018–5020.

were removed under reduced pressure. The residue was purified by flash column chromatography on silica gel, eluting with a mixture of hexane/EtOAc to obtain the corresponding alkenes which were used in the next step (not reported).

Step 2: A mixture of the corresponding bromostyrene (3 mmol), $PdCl_2(MeCN)_2$ (42 mg, 0.06 mmol, 0.02 eq), XPhos (43 mg, 0.09 mmol, 0.03 eq), and Cs_2CO_3 (2.91 g, 9 mmol, 3 eq) in anhydrous MeCN (4 mL, 0.75 M) was stirred under N_2 at rt for 25 min. Then trimethylsilylacetylene (442 mg, 4.5 mmol, 1.5 eq) was added, and the reaction was stirred at 80 ºC for 3 h (the complete consumption of starting material was monitored by GC-MS). After cooling the reaction mixture, EtOAc and water were added. The separated aqueous phase was extracted with EtOAc. The combined organic layers were dried over anhydrous Na_2SO_4 and concentrated under reduced pressure. The remaining residue was purified by column chromatography on silica gel using hexane and EtOAc as eluents to afford *o*-(trimethylsilyl)alkynyl styrene derivatives which were used in the next step (not reported).[4]

Step 3: To a solution of *o*-(trimethylsilyl)alkynyl styrene derivative (2.5 mmol, 1 eq) in MeOH (10 mL, 0.25 M) a 0 ºC was added K_2CO_3 (1.38 g, 10 mmol, 4 eq). The resulting mixture was stirred for 1 h at rt. Then crude was partitioned between aqueous NH_4Cl/DCM and the aqueous layer further extracted with DCM (2 × 10 mL). The organic phase was dried over anhydrous Na_2SO_4 and the solvents were removed under reduced pressure. The residue was purified by flash column chromatography using a mixture of hexane/EtOAc as eluent to obtain the corresponding *o*-(ethynyl)styrenes as *Z*-isomers **2a,d,e**.

Synthesis of *o*-(alkynyl)styrenes 3:

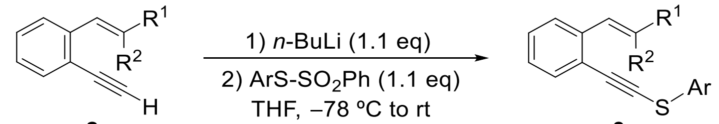

2 **3**

To a solution of the corresponding *o*-(ethynyl)styrenes **2** (2 mmol, 1 eq) in THF (4 mL, 0.5 M) was added *n*-BuLi (1.4 mL of a 1.6 M solution in hexane, 2.2 mmol, 1.1 eq) at −78 ºC and the resulting mixture was stirring at rt during 30 min. Then, the reaction is cooled to 0 ºC for adding the corresponding thiosulfonate, previously synthesized,[5] (2.2 mmol, 1.1 eq). The reaction was stirred until total consumption of starting material as determined by GC-MS (ca. 16 h). Then crude was partitioned between aqueous NH_4Cl/DCM and the aqueous layer further extracted with DCM (2 × 10 mL). The organic phase was dried over anhydrous Na_2SO_4 and the solvents were removed under reduced pressure. The residue was purified by flash column chromatography using a mixture of hexane/EtOAc as eluent to obtain the corresponding *o*-(alkynyl)styrenes **3**.

Synthesis of *o*-(alkynyl)styrenes 5:

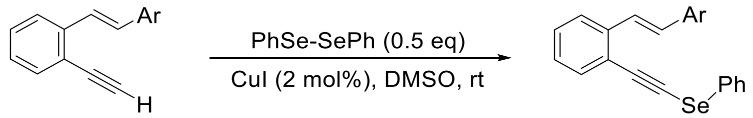

(*E*)-2 **(*E*)-5**

To a solution of the corresponding *o*-(ethynyl)styrene (***E***)-**2** (2 mmol, 1 eq) in DMSO (5 mL, 0.4 M) diphenyl diselenide (312 mg, 1 mmol, 0.5 eq) and CuI (7 mg, 0.04 mmol, 0.02 eq) were added. The

[4] Sanjuán, A. M.; Virumbrales, C.; García-García, P.; Fernández-Rodríguez, M. A.; Sanz, R. *Org. Lett.* **2016**, *18*, 1072–1075.

[5] Fujiki, K.; Tanifuji, N.; Sasaki, Y.; Yokoyama, T. *Synthesis* **2002**, *3*, 343–348.

reaction mixture was stirred until total consumption of starting material monitored by GC-MS (ca. 16 h).[6] The crude was partitioned between aqueous NH_4Cl/DCM and the aqueous layer further extracted with DCM (2 × 5 mL). The organic phase was dried over anhydrous Na_2SO_4 and the solvents were removed under reduced pressure. The residue was purified by flash column chromatography using a mixture of hexane/EtOAc as eluent to obtain the corresponding *o*-(alkynyl)styrenes **5**.

Synthesis of dihydroindeno[2,1-*b*]thio(seleno)chromenes 4 and 6:

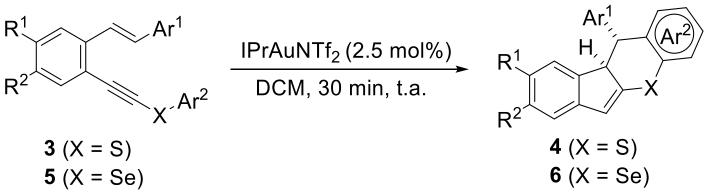

$IPrAuNTf_2$ (7 mg, 0.008 mmol, 0.025 eq) was added to a solution of the corresponding starting *o*-(alkynyl)styrene **3** or **5** (0.3 mmol, 1 eq) in DCM (0.6 mL, 0.5 M). The resulting reaction mixture was stirred at rt until total consumption of the styrene derivative was determined by GC-MS (~30 min). Then the mixture was filtered through a short pad of silica gel using a 100:1 mixture of hexane/EtOAc as eluent. The solvent was removed under reduced pressure, and the crude mixture was purified by flash column chromatography on silica gel using mixtures of hexane/EtOAc or hexane/DCM as eluents to obtain the corresponding dihydroindeno[2,1-*b*]thio(seleno)chromene **4** or **6** in the yields reported in Table 2.

Synthesis of disubstituted dihydroindeno[2,1-*b*]thiochromenes 4r-w:

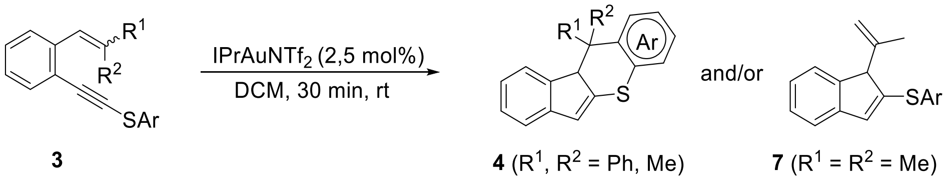

$IPrAuNTf_2$ (7 mg, 0.008 mmol, 0.025 eq) was added to a solution of the corresponding *β,β*-disubstituted *o*-(alkynyl)styrene **3** (0.3 mmol, 1 eq) in DCM (0.6 mL, 0.5 M). The resulting reaction mixture was stirred at rt until total consumption of the styrene derivative was determined by GC-MS (~30 min). Then the mixture was filtered through a short pad of silica gel using a 100:1 mixture of hexane/EtOAc as eluent, the solvent was removed under reduced pressure, and the crude mixture was purified by flash column chromatography on silica gel using mixtures of hexane/EtOAc or hexane/DCM as eluents to obtain the corresponding disubstituted dihydroindeno[2,1-*b*]thiochromenes **4** and/or indenes **7** in the yields reported in Scheme 3.

Synthesis of dihydroindeno[2,1-*b*]thiochromene 4d (1 mmol scale):

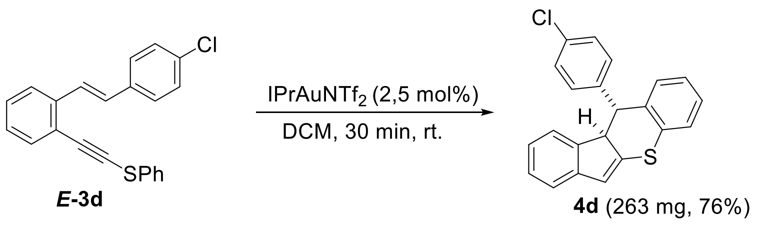

[6] Bieber, L. W.; da Silva, M. F.; Menezes, P. H. *Tetrahedron Lett.* **2004**, *45*, 2735–2737.

IPrAuNTf$_2$ (21 mg, 0.025 mmol, 0.025 eq) was added to a solution of (*E*)-((2-(4-chlorostyryl)phenyl)ethynyl)(phenyl)sulfane ***E*-3d** (346 mg, 1 mmol, 1 eq) in DCM (2 mL, 0.5 M). The resulting mixture was stirred at rt for 2 h. Then, the mixture was filtered through a short pad of silica gel using a 100:1 mixture of hexane/EtOAc as eluent. The solvent was removed under reduced pressure, and the crude mixture was purified by flash column chromatography on silica gel using a 40:1 mixture of hexane/EtOAc as eluent yielding (10*bR**,11*R**)-11-(4-chlorophenyl)-10*b*,11-dihydroindeno[2,1-*b*]thiochromene **4d** as a yellow oil (263 mg, 76%).

Characterization Data

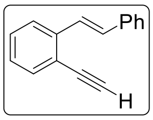

(*E*)-1-Ethynyl-2-styrylbenzene (*E*-2a):[7] yellow oil; R_f = 0.30 (hex/EtOAc, 50/1); 82% yield (502 mg); isolated as a > 20:1 mixture of *E*:*Z* isomers; ^{1}H NMR (300 MHz, CDCl$_3$) δ (ppm) 7.79–7.73 (m, 2H), 6.64–7.59 (m, 3H), 6.46–7.20 (m, 6H), 3.46 (s, 1H); ^{13}C NMR (75.4 MHz, CDCl$_3$) δ (ppm) 139.4 (C), 137.3 (C), 133.4 (CH), 130.7 (CH), 129.1 (CH), 128.8 (2 × CH), 128.0 (CH), 127.2 (CH), 126.9 (2 × CH), 126.5 (CH), 124.6 (CH), 121.1 (C), 82.3 (C), 82.2 (CH); LRMS (EI) *m/z* (%) 204 (M$^+$, 100), 202 (98), 101 (21).

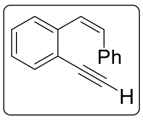

(*Z*)-1-Ethynyl-2-styrylbenzene (*Z*-2a): yellow oil; R_f = 0.23 (hex/EtOAc, 100/1); 62% yield (316 mg); isolated as a < 1:20 mixture of *E*:*Z* isomers; ^{1}H NMR (300 MHz, CDCl$_3$) δ (ppm) 7.58 (dd, *J* = 7.4, 1.0 Hz, 1H), 7.29–7.15 (m, 8H), 6.91 (d, *J* = 12.2 Hz, 1H), 6.76 (d, *J* = 12.2 Hz, 1H), 3.35 (s, 1H); ^{13}C NMR (75.4 MHz, CDCl$_3$) δ (ppm) 140.2 (C), 137.0 (C), 133.1 (CH), 131.6 (CH), 129.2 (CH), 129.1 (2 × CH), 128.7 (CH), 128.5 (CH), 128.3 (2 × CH), 127.4 (CH), 127.1 (CH), 121.8 (C), 82.4 (C), 81.8 (CH); LRMS (EI) *m/z* (%) 204 (M$^+$, 71), 202 (100), 101 (57); HRMS (ESI) calcd for $C_{16}H_{13}$ [M+H]$^+$ 205.1012, found 205.1015.

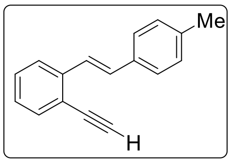

(*E*)-1-Ethynyl-2-(4-methylstyryl)benzene (*E*-2b):[8] yellow oil; R_f = 0.35 (hex/EtOAc, 50/1); 88% yield (575 mg); isolated as a > 20:1 mixture of *E*:*Z* isomers; ^{1}H NMR (300 MHz, CDCl$_3$) δ (ppm) 7.72 (d, *J* = 8.0 Hz, 1H), 7.63 (d, *J* = 16.4 Hz, 1H), 7.55–7.38 (m, 4H), 7.22–7.15 (m, 4H), 3.41 (s, 1H), 2.40 (s, 3H); ^{13}C NMR (75.4 MHz, CDCl$_3$) δ (ppm) 139.7 (C), 138.0 (C), 134.6 (C), 133.4 (CH), 130.7 (CH), 129.5 (2 × CH), 129.1 (CH), 127.1 (CH), 126.9 (2 × CH), 125.6 (CH), 124.6 (CH), 121.0 (C), 82.3 (C), 82.2 (CH), 21.4 (CH$_3$); LRMS (EI) *m/z* (%) 218 (M$^+$, 96), 202 (100).

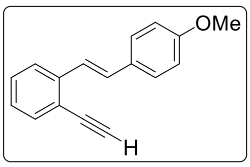

(*E*)-1-Ethynyl-2-(4-methoxystyryl)benzene (*E*-2c):[9] yellow oil; R_f = 0.30 (hex/EtOAc, 50/1); 83% yield (583 mg); isolated as a > 20:1 mixture of *E*:*Z* isomers; ^{1}H NMR (300 MHz, CDCl$_3$) δ (ppm) 7.71 (d, *J* = 7.9 Hz, 1H), 7.61–7.52 (m, 4H), 7.39–7.34 (m, 1H), 7.25–7.22 (m, 1H), 7.16 (d, *J* = 16.4 Hz, 1H), 6.95 (d, *J* = 8.7 Hz, 2H), 3.85 (s, 3H), 3.44 (s, 1H); ^{13}C NMR (75.4 MHz, CDCl$_3$) δ (ppm) 159.6 (C), 139.7 (C), 133.3 (CH), 130.2 (CH), 130.1 (C), 129.0 (CH), 128.2 (2 × CH), 126.8 (CH), 124.4 (CH), 124.3 (CH), 120.8 (C), 114.2 (2 × CH), 82.3 (C), 82.2 (CH), 55.3 (CH$_3$); LRMS (EI) *m/z* (%) 234 (M$^+$, 100), 219 (94), 189 (98).

---

[7] Vasu, D.; Hung, H.-H.; Bhunia, S.; Gawade, S. A.; Das, A.; Liu, R.-S. *Angew. Chem. Int. Ed.* **2011**, *50*, 6911–6914.
[8] Naveen, K.; Muralidharan, D.; Perumal, P. T. *Eur. J. Org. Chem.* **2014**, 1172–1176.
[9] Shen, H.-C.; Pal, S.; Lian, J.-J.; Liu, R.-S. *J. Am. Chem. Soc.* **2003**, *125*, 15762–15763.

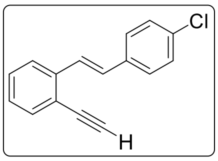

(*E*)-1-(4-Chlorostyryl)-2-ethynylbenzene (*E*-2d):[8] yellow oil; R_f = 0.34 (hex/EtOAc, 50/1); 85% yield (612 mg); isolated as a > 20:1 mixture of *E*:*Z* isomers; ^{1}H NMR (300 MHz, $CDCl_3$) δ (ppm) 7.69–7.62 (m, 2H), 7.57–7.46 (m, 2H), 7.39–7.33 (m, 3H), 7.26–7.00 (m, 3H), 3.43 (s, 1H); ^{13}C NMR (75.4 MHz, $CDCl_3$) δ (ppm) 139.1 (C), 135.8 (C), 133.6 (C), 133.4 (CH), 129.3 (CH), 129.1 (CH), 129.0 (2 × CH), 128.9 (CH), 128.0 (2 × CH), 127.5 (CH), 124.7 (CH), 121.3 (C), 82.5 (C), 82.1 (CH); LRMS (EI) *m/z* (%) 240 (M^++2, 25), 238 (M^+, 82), 202 (100).

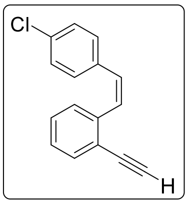

(*Z*)-1-(4-Chlorostyryl)-2-ethynylbenzene (*Z*-2d): yellow oil; R_f = 0.14 (hex/EtOAc, 100/1); 80% yield (480 mg); isolated as a < 1:20 mixture of *E*:*Z* isomers; ^{1}H NMR (300 MHz, $CDCl_3$) δ (ppm) 5.75 (d, *J* = 7.4 Hz, 1H), 7.23–7.13 (m, 7H), 6.91 (d, *J* = 12.2 Hz, 1H), 6.68 (d, *J* = 12.2 Hz, 1H), 3.34 (s, 1H); ^{13}C NMR (75.4 MHz, $CDCl_3$) δ (ppm) 139.8 (C), 135.4 (C), 133.2 (CH), 133.0 (C), 130.4 (2 × CH), 130.3 (CH), 129.4 (CH), 129.0 (CH), 128.6 (CH), 128.5 (2 × CH), 127.4 (CH), 121.8 (C), 82.3 (C), 82.0 (CH); LRMS (EI) *m/z* (%) 240 (M^++2, 18), 238 (M^+, 60), 202 (100).

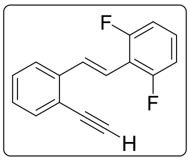

(*E*)-2-(2-Ethynylstyryl)-1,3-difluorobenzene (*E*-2e): yellow oil; R_f = 0.34 (hex/EtOAc, 40/1); 74% yield (533 mg); isolated as a > 20:1 mixture of *E*:*Z* isomers; ^{1}H NMR (300 MHz, $CDCl_3$) δ (ppm) 8.00 (d, *J* = 16.8 Hz, 1H), 7.75 (d, *J* = 8.0 Hz, 1H), 7.56 (dd, *J* = 7.5, 1.2 Hz, 1H), 7.40 (td, *J* = 7.5, 1.2 Hz, 1H), 7.30–7.18 (m, 3H), 6.95 (t, *J* = 8.0 Hz, 2H), 3.41 (s, 1H); ^{13}C NMR (75.4 MHz, $CDCl_3$) δ (ppm) 161.3 (dd, *J* = 252.0, 7.6 Hz, 2 × C), 139.9 (C), 133.4 (CH), 133.0 (t, *J* = 8.6 Hz, CH), 129.2 (CH), 128.4 (t, *J* = 10.8 Hz, CH), 127.8 (CH), 124.8 (CH), 121.6 (C), 117.3 (CH), 114.9 (t, *J* = 15.1 Hz, C), 111.7 (dd, *J* = 25.0, 8.0 Hz, 2 × CH), 82.4 (C), 81.9 (CH); LRMS (EI) *m/z* (%) 240 (M^+, 100), 238 (52), 220 (42); HRMS (APCI) calcd for $C_{16}H_{11}F_2$ $[M+H]^+$ 241.0823, found 241.0822.

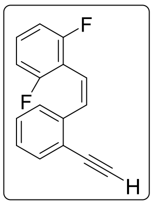

(*Z*)-2-(2-Ethynylstyryl)-1,3-difluorobenzene (*Z*-2e): colorless oil; R_f = 0.30 (hex/EtOAc, 40/1); 85% yield (510 mg); isolated as a < 1:20 mixture of *E*:*Z* isomers; ^{1}H NMR (300 MHz, $CDCl_3$) δ (ppm) 7.55 (d, *J* = 7.8 Hz, 1H), 7.26–7.10 (m, 5H), 6.84 (t, *J* = 7.8 Hz, 2H), 6.55 (d, *J* = 12.1 Hz, 1H), 3.37 (s, 1H); ^{13}C NMR (75.4 MHz, $CDCl_3$) δ (ppm) 160.4 (dd, *J* = 250.1, 7.8 Hz, 2 × C), 140.1 (C), 133.8 (CH), 133.0 (CH), 129.0 (t, *J* = 10.2 Hz, CH), 128.5 (CH), 127.5 (CH), 127.3 (CH), 121.6 (C), 117.4 (CH), 114.7 (t, *J* = 19.7 Hz, C), 111.5 (dd, *J* = 25.4, 10.5 Hz, 2 × CH), 82.2 (CH), one aromatic C does not appear due to overlapping; LRMS (EI) *m/z* (%) 240 (M^+, 100), 238 (48), 220 (52); HRMS (APCI) calcd for $C_{16}H_{11}F_2$ $[M+H]^+$ 241.0823, found 241.0823.

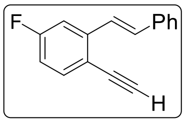

(*E*)-1-Ethynyl-4-fluoro-2-styrylbenzene (*E*-2o): yellow oil; R_f = 0.35 (hex/EtOAc, 50/1); 80% yield (533 mg); isolated as a > 20:1 mixture of *E*:*Z* isomers; ^{1}H NMR (300 MHz, $CDCl_3$) δ (ppm) 7.70–7.54 (m, 4H), 7.43–7.28 (m, 4H), 7.17 (d, *J* = 16.3 Hz, 1H), 6.99–6.93 (m, 1H), 3.42 (s, 1H); ^{13}C NMR (75.4 MHz, $CDCl_3$) δ (ppm) 162.9 (d, *J* = 249.1 Hz, C), 141.9 (d, *J* = 8.3 Hz, C), 136.8 (C), 135.1 (d, *J* = 8.8 Hz, CH), 131.8 (CH), 128.8 (2 × CH), 128.4 (CH), 127.0 (2 × CH), 125.4 (d, *J* = 2.6 Hz, CH), 117.2 (d, *J* = 2.6 Hz, C), 114.7 (d, *J* = 22.7 Hz, CH), 111.2 (d, *J* = 22.7 Hz, CH), 82.0 (C), 81.3 (CH); LRMS (EI) *m/z* (%) 222 (M^+, 100), 202 (8); HRMS (ESI) calcd for $C_{16}H_{12}F$ $[M+H]^+$ 223.0918, found 223.0918.

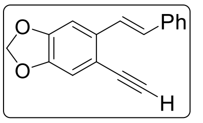

(*E*)-5-Ethynyl-6-styrylbenzo[*d*][1,3]dioxole (*E*-2p):[9] white solid; mp = 100–112 ºC; R_f = 0.28 (hex/EtOAc, 50/1); 84% yield (625 mg); isolated as a > 20:1 mixture of *E*:*Z* isomers; ^{1}H NMR (300 MHz, $CDCl_3$) δ (ppm) 7.64 (d, J = 16.3 Hz, 1H), 7.61–7.53 (m, 2H), 7.40–7.28 (m, 3H), 7.16 (s, 1H), 7.00 (d, J = 16.3 Hz, 1H), 6.95 (s, 1H), 5.99 (s, 2H), 3.35 (s, 1H); ^{13}C NMR (75.4 MHz, $CDCl_3$) δ (ppm) 149.0 (C), 147.0 (C), 137.4 (C), 135.1 (C), 129.1 (CH), 128.8 (2 × CH), 127.8 (CH), 126.8 (2 × CH), 126.3 (CH), 114.8 (C), 112.2 (CH_2), 104.2 (CH), 101.7 (CH), 82.2 (C), 81.2 (CH); LRMS (EI) *m/z* (%) 248 (M^+, 20), 218 (100), 202 (85); HRMS (ESI) calcd for $C_{17}H_{13}O_2$ $[M+H]^+$ 249.0910, found 249.0913.

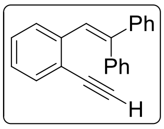

(*E*)-(2-(2-Ethynylphenyl)ethene-1,1-diyl)dibenzene (2r): yellow solid; mp = 90–92 ºC; R_f = 0.13 (hexane); 83% yield (617 mg); ^{1}H NMR (300 MHz, $CDCl_3$) δ (ppm) 7.50 (dd, J = 7.6, 1.1 Hz, 1H), 7.38–7.28 (m, 9H), 7.22–7.19 (m, 2H), 7.09 (td, J = 7.6, 1.1 Hz, 1H), 6.98 (td, J = 7.6, 1.1 Hz, 1H), 6.82 (dd, J = 7.6, 1.1 Hz, 1H), 3.34 (s, 1H); ^{13}C NMR (75.4 MHz, $CDCl_3$) δ (ppm) 144.3 (C), 143.4 (C), 140.33 (C), 140.28 (C), 132.9 (CH), 130.8 (2 × CH), 129.4 (CH), 128.6 (2 × CH), 128.3 (2 × CH), 128.2 (2 × CH), 128.1 (CH), 127.9 (CH), 127.6 (CH), 126.6 (CH), 126.4 (CH), 122.7 (C), 82.7 (C), 82.3 (CH); LRMS (EI) *m/z* (%) 280 (M^+, 56), 202 (100); HRMS (ESI) calcd for $C_{22}H_{17}$ $[M+H]^+$ 281.1325, found 281.1325.

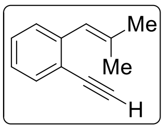

(*E*)-1-Ethynyl-2-(2-methylprop-1-en-1-yl)benzene (2u):[7] yellow oil; R_f = 0.33 (hex/EtOAc, 100/1); 85% yield (398 mg); ^{1}H NMR (300 MHz, $CDCl_3$) δ (ppm) 7.55–7.52 (m, 1H), 7.34–7.29 (m, 2H), 7.21–7.16 (m, 1H), 6.53 (s, 1H), 3.29 (s, 1H), 1.98 (d, J = 1.4 Hz, 3H), 1.86 (d, J = 1.4 Hz, 3H); ^{13}C NMR (75.4 MHz, $CDCl_3$) δ (ppm) 141.2 (C), 137.1 (C), 133.0 (CH), 129.3 (CH), 128.4 (CH), 126.0 (CH), 123.8 (CH), 121.5 (C), 82.9 (C), 81.1 (CH), 26.8 (CH_3), 19.7 (CH_3); LRMS (EI) *m/z* (%) 156 (M^+, 36), 141 (100), 115 (48); HRMS (APCI) calcd for $C_{12}H_{13}$ $[M+H]^+$ 157.1012, found 157.1016.

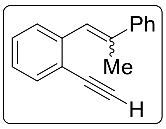

(*E*)-1-Ethynyl-2-(2-phenylprop-1-en-1-yl)benzene (2w):[7] yellow oil; R_f = 0.29 (hex/EtOAc, 100/1); 78% yield (510 mg); isolated as a 2:1 mixture of *E*:*Z* isomers; data for both isomers: ^{1}H NMR (300 MHz, $CDCl_3$) δ (ppm) 7.65–7.62 (m, 2H, both), 7.30–7.22 (m, 14H, both), 7.08 (td, J = 7.5, 1.3 Hz, 1H, maj), 6.98 (td, J = 7.5, 1.3 Hz, 1H, maj), 6.88 (d, J = 1.1 Hz, 1H, maj), 6.82–6.79 (m, 1H, maj), 3.39 (s, 1H, maj), 3.34 (s, 1H, min), 2.33 (d, J = 1.1 Hz, 3H, maj), 2.29 (d, J = 1.1 Hz, 3H, min); ^{13}C NMR (75.4 MHz, $CDCl_3$) δ (ppm) 143.6 (C), 141.8 (C), 141.0 (C), 140.5 (C), 140.4 (C), 138.6 (C), 133.0 (C), 132.8 (C), 129.6 (CH), 129.3 (CH), 128.4 (4 × CH), 128.2 (CH), 128.1 (CH), 127.5 (CH), 127.1 (CH), 126.6 (CH), 126.3 (CH), 126.2 (CH), 126.1 (CH), 125.1 (CH), 122.2 (C), 121.6 (C), 82.8 (C), 82.6 (C), 81.8 (CH), 81.6 (CH), 26.8 (CH_3), 17.6 (CH_3), 3 CH do not appear due to overlapping; LRMS (EI) *m/z* (%) 218 (M^+, 40), 202 (100); HRMS (ESI) calcd for $C_{17}H_{15}$ $[M+H]^+$ 219.1168; found 219.1165.

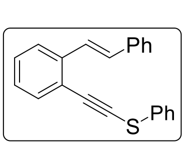

(*E*)-Phenyl-((2-styrylphenyl)ethynyl)sulfane (*E*-3a): brown solid; mp = 66–68 ºC; R_f = 0.25 (hex/EtOAc, 100/1); 88% yield (549 mg), isolated as a > 20:1 mixture of *E*:*Z* isomers; ^{1}H NMR (300 MHz, $CDCl_3$) δ (ppm) 7.79–7.65 (m, 2H), 7.62–7.56 (m, 4H), 7.47–7.28 (m, 9H), 7.25 (d, J = 16.0 Hz, 1H); ^{13}C NMR (75.4 MHz, $CDCl_3$) δ (ppm) 139.8 (C), 136.9 (C), 133.1 (C), 132.4 (CH), 131.7 (CH), 129.4 (2 × CH), 129.2 (CH), 129.1 (2 × CH), 128.8 (CH), 128.3 (CH), 128.2 (CH), 127.2 (CH), 126.7 (2 × CH), 126.62 (2 × CH), 126.59

(CH), 122.6 (C), 97.0 (C), 80.0 (C); LRMS (EI) *m/z* (%) 312 (M^+, 60), 235 (100); HRMS (ESI) calcd for $C_{22}H_{16}NaS$ $[M+Na]^+$ 335.1330; found 335.1336.

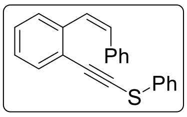

(*Z*)-Phenyl-((2-styrylphenyl)ethynyl)sulfane (*Z*-3a): brown solid, mp = 66–68 ºC, R_f = 0.20 (hexane); 65% yield (405 mg); obtained and isolated as a *Z*-isomer; ^{1}H NMR (300 MHz, $CDCl_3$) δ (ppm) 7.78–7.27 (m, 14H), 6.93 (d, J = 12.2 Hz, 1H), 6.77 (d, J = 12.2 Hz, 1H); ^{13}C NMR (75.4 MHz, $CDCl_3$) δ (ppm) 138.8 (C), 137.2 (C), 133.0 (C), 132.5 (CH), 130.7 (CH), 129.4 (2 × CH), 128.8 (2 × CH), 128.7 (CH), 128.0 (CH), 127.3 (CH), 126.9 (2 × CH), 126.7 (CH), 126.5 (CH), 126.4 (2 × CH), 124.8 (CH), 122.0 (C), 97.0 (C), 80.0 (C); LRMS (EI) *m/z* (%) 312 (M^+, 60), 235 (100); HRMS (ESI) calcd for $C_{22}H_{17}S$ $[M+H]^+$ 313.1045; found 313.1039.

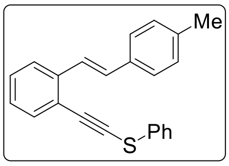

(*E*)-((2-(4-Methylstyryl)phenyl)ethynyl)(phenyl)sulfane (*E*-3b): orange oil; R_f = 0.18 (hex/EtOAc, 100/1); 80% yield (522 mg); isolated as a > 20:1 mixture of *E*:*Z* isomers; ^{1}H NMR (300 MHz, $CDCl_3$) δ (ppm) 7.80–7.23 (m, 15H), 2.47 (s, 3H); ^{13}C NMR (75.4 MHz, $CDCl_3$) δ (ppm) 138.9 (C), 138.0 (C), 134.5 (C), 133.2 (C), 132.6 (CH), 130.8 (CH), 129.52 (2 × CH), 129.50 (2 × CH), 128.8 (CH), 127.2 (CH), 126.9 (2 × CH), 126.7 (CH), 126.5 (2 × CH), 125.6 (CH), 124.8 (CH), 121.9 (C), 96.8 (C), 80.5 (C), 21.4 (CH_3); LRMS (EI) *m/z* (%) 326 (M^+, 23), 217 (100), 202 (85); HRMS (ESI) could not be recorded.

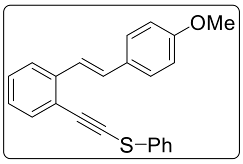

(*E*)-((2-(4-Methoxystyryl)phenyl)ethynyl)(phenyl)sulfane (*E*-3c): yellow oil; R_f = 0.22 (hex/EtOAc, 100/1); 75% yield (513 mg); isolated as a > 20:1 mixture of *E*:*Z* isomers; ^{1}H NMR (300 MHz, $CDCl_3$) δ (ppm) 7.70 (d, J = 7.9 Hz, 1H), 7.58–7.18 (m, 12H), 6.95–6.88 (m, 2H), 3.84 (s, 3H); ^{13}C NMR (75.4 MHz, $CDCl_3$) δ (ppm) 159.6 (C), 139.2 (C), 133.1 (C), 132.5 (C), 130.3 (CH), 130.0 (CH), 129.5 (2 × CH), 128.7 (CH), 128.2 (2 × CH), 126.9 (CH), 126.6 (2 × CH), 126.4 (CH), 124.5 (CH), 124.3 (CH), 121.6. (C), 114.2 (2 × CH), 96.8 (C), 80.4 (C), 55.4 (CH_3); LRMS (EI) *m/z* (%) 342 (M^+, 32), 233 (100), 189 (35); HRMS (ESI) calcd for $C_{23}H_{19}OS$ $[M+H]^+$ 343.1151; found 343.1143.

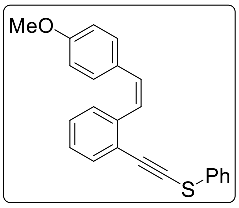

(*Z*)-((2-(4-Methoxystyryl)phenyl)ethynyl)(phenyl)sulfane (*Z*-3c): yellow oil; R_f = 0.23 (hex/EtOAc, 100/1); isolated as a < 1:20 mixture of *E*:*Z* isomers; ^{1}H NMR (300 MHz, $CDCl_3$) δ (ppm) 7.57–7.24 (m, 3H), 7.40–7.35 (m, 3H), 7.29–7.23 (m, 5H), 6.85–6.81 (m, 3H), 6.71 (d, J = 12.2 Hz, 1H), 3.82 (s, 3H); ^{13}C NMR (75.4 MHz, $CDCl_3$) δ (ppm) 158.9 (C), 140.2 (C), 133.2 (C), 132.4 (CH), 131.2 (CH), 130.4 (2 × CH), 129.4 (C), 129.3 (2 × CH), 129.1 (CH), 128.2 (CH), 127.1 (CH), 127.0 (CH), 126.5 (CH), 126.2 (2 × CH), 122.6 (C), 113.7 (2 × CH), 97.1 (C), 79.8 (C), 55.3 (CH_3); LRMS (EI) *m/z* (%) 342 (M^+, 32), 233 (100), 189 (35); HRMS (ESI) could not be recorded.

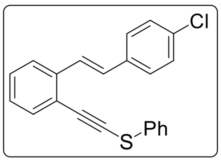

(*E*)-((2-(4-Chlorostyryl)phenyl)ethynyl)(phenyl)sulfane (*E*-3d): yellow oil; R_f = 0.16 (hex/EtOAc, 100/1); 85% yield (588 mg); isolated as a > 20:1 mixture of *E*:*Z* isomers; ^{1}H NMR (300 MHz, $CDCl_3$) δ (ppm) 7.72–7.54 (m, 4H), 7.44–7.27 (m, 9H), 7.15 (d, J = 16.3 Hz, 1H); ^{13}C NMR (75.4 MHz, $CDCl_3$) δ (ppm) 138.5 (C), 135.8 (C), 133.6 (C), 133.1 (C), 132.6 (CH), 129.5 (2 × CH), 129.4 (CH), 129.0 (2 × CH), 128.8 (CH), 128.1 (2 × CH), 127.6 (CH), 127.2 (CH), 126.8 (CH), 126.6 (2 × CH),

124.9 (CH), 122.1 (C), 96.5 (C), 81.0 (C); LRMS (EI) *m/z* (%) 348 (M^++2, 4), 346 (M^+, 12), 234 (41), 202 (100); HRMS (APCI) calcd for $C_{22}H_{15}ClS$ $[M+H]^+$ 347.0656, found 347.0659.

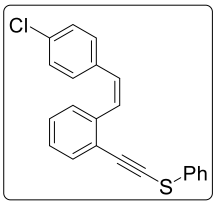

(*Z*)-((2-(4-Chlorostyryl)phenyl)ethynyl)(phenyl)sulfane (*Z*-3d): yellow oil; R_f= 0.18 (hex/EtOAc, 100/1); 45% yield (311 mg); isolated as < 1:20 mixture of *E*:*Z* isomers; ^{1}H NMR (300 MHz, $CDCl_3$) δ (ppm) 7.58–7.19 (m, 13H), 6.93 (d, *J* = 12.2 Hz, 1H), 6.70 (d, *J* = 12.2 Hz, 1H); ^{13}C NMR (75.4 MHz, $CDCl_3$) δ (ppm) 139.4 (C), 135.4 (C), 132.5 (C), 130.45 (2 × C), 130.41 (CH), 129.5 (CH), 129.4 (2 × CH), 129.2 (2 × CH), 129.1 (CH), 128.5 (2 × CH), 128.4 (CH), 127.6 (CH), 126.4 (CH), 127.3 (CH), 126.6 (CH), 126.3 (C), 96.8 (C), 80.2 (C); LRMS (EI) *m/z* (%) 348 (M^++2, 6), 346 (M^+, 18), 234 (42), 202 (100); HRMS (ESI) calcd for $C_{22}H_{15}ClS$ $[M+H]^+$ 347.0656, found 347.0635.

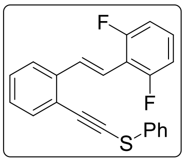

(*E*)-((2-(2,6-Difluorostyryl)phenyl)ethynyl)(phenyl)sulfane (*E*-3e): yellow oil; R_f = 0.16 (hex/EtOAc, 100/1); 85% yield (592 mg); isolated as a > 20:1 mixture of *E*:*Z* isomers; ^{1}H NMR (300 MHz, $CDCl_3$) δ (ppm) 8.03 (d, *J* = 16.8 Hz, 1H) 7.77 (d, *J* = 7.8 Hz, 1H) 7.57 (d, *J* = 7.8 Hz, 3H), 7.36–7.22 (m, 7H), 6.94 (t, *J* = 8.5 Hz, 2H); ^{13}C NMR (75.4 MHz, $CDCl_3$) δ (ppm) 161.1 (dd, *J* = 252.2, 7.6 Hz, 2 × C), 139.3 (C), 133.1 (t, *J* = 8.6 Hz, CH), 133.0 (C), 132.7 (CH), 129.4 (2 × CH), 128.9 (CH), 128.4 (t, *J* = 8.8 Hz, CH), 127.9 (CH), 126.7 (CH), 126.5 (2 × CH), 124.9 (CH), 122.5 (C), 117.3 (CH), 114.9 (t, *J* = 15.0 Hz, C), 111.7 (dd, *J* = 25.8, 7.7 Hz, 2 × CH), 96.4 (C), 80.8 (C); LRMS (EI) *m/z* (%) 348 (M^+, 12), 234 (41), 202 (100); HRMS (ESI) calcd for $C_{22}H_{15}F_2S$ $[M+H]^+$ 349.0857, found 349.0862.

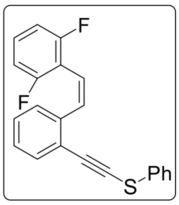

(*Z*)-((2-(2,6-Difluorostyryl)phenyl)ethynyl)(phenyl)sulfane (*Z*-3e): yellow oil; R_f= 0.18 (hex/EtOAc, 100/1); 51% yield (355 mg); isolated as < 1:20 mixture of *E*:*Z* isomers; ^{1}H NMR (300 MHz, $CDCl_3$) δ (ppm) 7.57–7.54 (m, 3H), 7.41–7.36 (m, 2H), 7.29–7.22 (m, 4H), 7.12–7.10 (m, 2H), 6.87–6.82 (m, 2H), 6.54 (d, *J* = 12.0 Hz, 1H); ^{13}C NMR (75.4 MHz, $CDCl_3$) δ (ppm) 160.5 (dd, *J* = 250.3, 7.8 Hz, 2 × C), 139.7 (C), 134.0 (t, *J* = 8.3 Hz, CH), 133.2 (C), 132.4 (CH), 129.4 (2 × CH), 129.0 (t, *J* = 8.3 Hz, CH), 128.2 (CH), 127.6 (CH), 127.5 (CH), 126.6 (CH), 126.3 (2 × CH), 122.4 (C), 117.5 (CH), 114.6 (t, *J* = 19.6 Hz, C), 111.5 (dd, *J* = 25.8, 2.4 Hz, 2 × CH), 96.7 (C), 80.5 (C); LRMS (EI) *m/z* (%) 348 (M^+, 8), 271 (100), 238 (98); HRMS (ESI) could not be recorded.

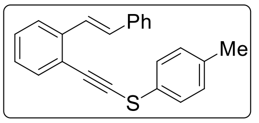

(*E*)-((2-Styrylphenyl)ethynyl)(*p*-tolyl)sulfane (*E*-3f): yellow oil; R_f = 0.20 (hex/EtOAc, 100/1); 85% yield (554 mg); isolated as a 11:1 mixture of *E*:*Z* isomers; ^{1}H NMR (300 MHz, $CDCl_3$) δ (ppm) 7.75 (d, *J* = 8.0 Hz, 1H), 7.68 (d, *J* = 16.3 Hz, 1H), 7.56–7.16 (m, 13H), 2.38 (s, 3H); ^{13}C NMR (75.4 MHz, $CDCl_3$) δ (ppm) 138.9 (C), 137.3 (C), 136.9 (C), 132.4 (CH), 130.7 (CH), 130.3 (2 × CH), 130.1 (C), 128.8 (2 × CH), 128.6 (CH), 128.0 (CH), 127.4 (CH), 127.0 (2 × CH), 126.9 (2 × CH), 126.7 (CH), 124.9 (CH), 122.2 (C), 96.0 (C), 81.5 (C), 21.1 (CH_3); LRMS (EI) *m/z* (%) 326 (M^+, 10), 234 (21), 203 (100); HRMS (ESI) calcd for $C_{23}H_{19}S$ $[M+H]^+$ 327.1202, found 327.1205.

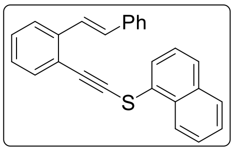

(*E*)-Naphthalen-1-yl-((2-styrylphenyl)ethynyl)sulfane (*E*-3g): white solid; mp = 102–104 ºC; R_f = 0.30 (hex/EtOAc, 50/1); 82% yield (594 mg); isolated as a > 20:1 mixture of *E*:*Z* isomers; ^{1}H NMR (300 MHz, $CDCl_3$) δ (ppm) 8.04 (s, 1H), 7.83–7.21 (m, 17H); ^{13}C NMR (75.4 MHz, $CDCl_3$) δ (ppm) 139.0 (C), 137.2 (C),

133.8 (C), 132.6 (CH), 132.2 (C), 130.9 (CH), 130.3 (C), 129.3 (CH), 128.8 (3 × CH), 128.0 (CH), 127.9 (CH), 127.4 (CH), 127.3 (CH), 127.0 (CH), 126.9 (2 × CH), 126.6 (CH), 126.1 (CH), 125.0 (CH), 124.9 (CH), 124.4 (CH), 122.0 (C), 96.7 (C), 80.9 (C); LRMS (EI) *m/z* (%) 362 (M^+, 20), 281 (34), 203 (100); HRMS (ESI) calcd for $C_{26}H_{19}S$ $[M+H]^+$ 363.1202; found 363.1190.

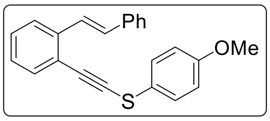

(*E*)-(4-Methoxyphenyl)-((2-styrylphenyl)ethynyl)sulfane (*E*-3h): brown oil; R_f = 0.15 (hex/EtOAc, 100/1); 88% yield (602 mg); isolated as a > 20:1 mixture of *E*:*Z* isomers; ^{1}H NMR (300 MHz, $CDCl_3$) δ (ppm) 7.76–7.69 (m, 3H), 7.59–7.54 (m, 4H), 7.45–7.21 (m, 6H), 6.93 (d, J = 8.9 Hz, 2H), 3.80 (s, 3H); ^{13}C NMR (75.4 MHz, $CDCl_3$) δ (ppm) 159.1 (C), 138.5 (C), 137.2 (C), 132.1 (CH), 130.5 (CH), 129.2 (2 × CH), 128.7 (2 × CH), 128.4 (CH), 127.9 (CH), 127.3 (CH), 126.8 (2 × CH), 126.5 (CH), 124.8 (CH), 122.9 (C), 122.1 (C), 115.2 (2 × CH), 95.1 (C), 82.5 (C), 55.3 (CH_3); LRMS (EI) *m/z* (%) 342 (M^+, 10), 234 (23), 202 (100); HRMS (ESI) calcd for $C_{23}H_{19}OS$ $[M+H]^+$ 343.1151, found 343.1152.

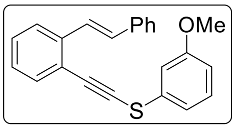

(*E*)-(3-Methoxyphenyl)-((2-styrylphenyl)ethynyl)sulfane (*E*-3i): yellow oil; R_f = 0.25 (hex/EtOAc, 100/1); 81% yield (554 mg); isolated as a 14:1 mixture of isomers; ^{1}H NMR (300 MHz, $CDCl_3$) δ (ppm) 7.74 (d, J = 16.0 Hz, 1H), 7.59–7.56 (m, 3H), 7.41–7.19 (m, 10H), 6.85 (ddd, J = 8.1, 2.4, 1.1 Hz, 1H), 3.79 (s, 3H); ^{13}C NMR (75.4 MHz, $CDCl_3$) δ (ppm) 160.4 (C), 138.9 (C), 137.2 (C), 134.3 (C), 132.6 (CH), 130.8 (CH), 130.3 (CH), 128.8 (2 × CH), 128.7 (CH), 128.0 (CH), 127.4 (CH), 126.9 (2 × CH), 126.5 (CH), 124.8 (CH), 122.0 (C), 118.7 (CH), 112.7 (CH), 111.8 (CH), 96.9 (C), 80.6 (C), 55.3 (CH_3); LRMS (EI) *m/z* (%) 342 (M^+, 4), 234 (55), 203 (100); HRMS (ESI) calcd for $C_{23}H_{19}OS$ $[M+H]^+$ 343.1151, found 343.1153.

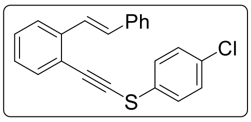

(*E*)-(4-Chlorophenyl)-((2-styrylphenyl)ethynyl)sulfane (*E*-3j): brown solid; mp = 78–80 ºC; R_f = 0.23 (hex/EtOAc, 100/1); 91% yield (630 mg); isolated as a > 20:1 mixture of *E*:*Z* isomers; ^{1}H NMR (300 MHz, $CDCl_3$) δ (ppm) 7.73 (d, J = 8.0 Hz, 1H), 7.63 (d, J = 16.3 Hz, 1H), 7.56–7.47 (m, 5H), 7.41–7.18 (m, 8H); ^{13}C NMR (75.4 MHz, $CDCl_3$) δ (ppm) 139.0 (C), 137.2 (2 × C), 132.5 (CH), 131.7 (C), 131.0 (CH), 129.6 (2 × CH), 128.94 (CH), 128.90 (2 × CH), 128.2 (CH), 127.8 (2 × CH), 127.4 (CH), 126.9 (2 × CH), 126.5 (CH), 124.9 (CH), 121.7 (C), 97.1 (C), 80.1 (C); LRMS (EI) *m/z* (%) 348 (M^++2, 4), 346 (M^+, 6), 234 (18), 203 (100); HRMS (ESI) calcd for $C_{22}H_{16}ClS$ $[M+H]^+$ 347.0656; found 347.0657.

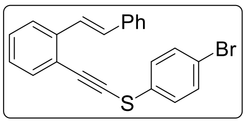

(*E*)-(4-Bromophenyl)-((2-styrylphenyl)ethynyl)sulfane (*E*-3k): yellow oil; R_f = 0.20 (hex/EtOAc, 100/1); 78% yield (608 mg); isolated as a > 20:1 mixture of *E*:*Z* isomers; ^{1}H NMR (300 MHz, $CDCl_3$) δ (ppm) 7.74 (d, J = 7.9 Hz, 1H), 7.64 (d, J = 16.3 Hz, 1H), 7.55–7.52 (m, 3H), 7.41–7.19 (m, 10H); ^{13}C NMR (75.4 MHz, $CDCl_3$) δ (ppm) 139.0 (C), 137.2 (C), 132.5 (3 × CH), 132.4 (C), 131.0 (CH), 129.0 (CH), 128.9 (2 × CH), 128.2 (CH), 128.0 (2 × CH), 127.4 (CH), 126.9 (2 × CH), 126.5 (CH), 125.0 (CH), 121.7 (C), 120.5 (C), 97.3 (C), 79.9 (C); LRMS (EI) *m/z* (%) 392 (M^++2, 12), 390 (M^+, 12), 203 (100); HRMS (ESI) calcd for $C_{22}H_{16}BrS$ $[M+H]^+$ 391.0151, found 391.0152.

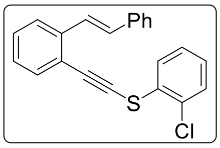

(*E*)-(2-Chlorophenyl)-((2-styrylphenyl)ethynyl)sulfane (*E*-3l): yellow oil; R_f = 0.15 (hex/EtOAc, 100/1); 84% yield (581 mg); isolated as a > 20:1 mixture of *E*:*Z* isomers; ^{1}H NMR (300 MHz, $CDCl_3$) δ (ppm) 7.98–7.50 (m, 15H); ^{13}C NMR (75.4 MHz, $CDCl_3$) δ (ppm) 139.0 (C), 137.0 (C), 132.72 (C), 132.68 (CH), 130.9 (CH), 130.3 (C), 129.5 (CH), 129.0 (CH), 128.8 (2 × CH), 128.1 (CH), 127.8 (CH), 127.3 (2 × CH), 127.0 (CH), 126.9 (2 × CH), 126.3 (CH), 124.8 (CH), 121.6 (C), 98.5 (C), 79.2 (C); LRMS (EI) *m/z* (%) 348 (M^++2, 32), 346 (M^+, 88), 269 (100), 234 (30); HRMS (ESI) calcd for $C_{22}H_{16}ClS$ $[M+H]^+$ 347.0656, found 347.0660.

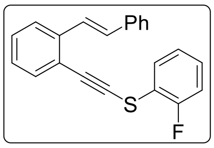

(*E*)-(2-Fluorophenyl)-((2-styrylphenyl)ethynyl)sulfane (*E*-3m): yellow oil; R_f = 0.17 (hex/EtOAc, 100/1); 81% yield (535 mg); isolated as a > 20:1 mixture of *E*:*Z* isomers; ^{1}H NMR (300 MHz, $CDCl_3$) δ (ppm) 7.76–7.73 (m, 2H), 7.67 (d, *J* = 16.3 Hz, 1H), 7.56–7.53 (m, 3H), 7.40–7.08 (m, 9H); ^{13}C NMR (75.4 MHz, $CDCl_3$) δ (ppm) 159.1 (d, *J* = 246.0 Hz, C), 139.2 (C), 137.3 (C), 132.8 (CH), 131.0 (CH), 129.1 (CH), 128.9 (2 × CH), 128.6 (d, *J* = 3.3 Hz, CH), 128.4 (d, *J* = 7.4 Hz, CH), 128.1 (CH), 127.4 (CH), 127.0 (2 × CH), 126.5 (CH), 125.3 (d, *J* = 3.4 Hz, CH), 125.0 (CH), 121.8 (C), 120.6 (d, *J* = 16.2 Hz, C), 115.6 (d, *J* = 20.3 Hz, CH), 96.9 (C), 78.7 (d, *J* = 3.3 Hz, C); LRMS (EI) *m/z* (%) 330 (M^+, 21), 234 (30), 203 (100); HRMS (ESI) calcd for $C_{22}H_{16}FS$ $[M+H]^+$ 331.0951, found 331.0947.

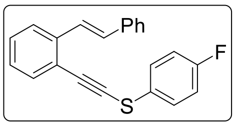

(*E*)-(4-Fluorophenyl)-((2-styrylphenyl)ethynyl)sulfane (*E*-3n): colorless solid; R_f = 0.25 (hex/DCM, 10/1); 85% yield (561 mg); isolated as a > 20:1 mixture of *E*:*Z* isomers; ^{1}H NMR (300 MHz, $CDCl_3$) δ (ppm) 7.75–7.72 (m, 1H), 7.66–7.51 (m, 5H), 7.42–7.27 (m, 7H), 7.05 (t, *J* = 8.6 Hz, 2H); ^{13}C NMR (75.4 MHz, $CDCl_3$) δ (ppm) 162.0 (d, *J* = 246.8 Hz, C), 139.0 (C), 137.2 (C), 132.5 (CH), 130.9 (CH), 128.84 (2 × CH), 128.80 (d, *J* = 7.7 Hz, 2 × CH), 128.2 (CH), 128.1 (d, *J* = 3.2 Hz, C), 127.4 (CH), 126.9 (2 × CH), 126.6 (CH), 124.9 (CH), 121.9 (C), 116.7 (d, *J* = 22.5 Hz, 2 × CH), 96.3 (C), 81.0 (C), one aromatic CH do not appear due to overlapping; LRMS (EI) *m/z* (%) 330 (M^+, 24), 234 (38), 203 (100), 202 (61); HRMS (ESI) calcd for $C_{22}H_{16}FS$ $[M+H]^+$ 331.0951, found 331.0948.

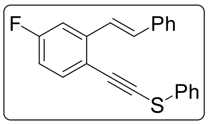

(*E*)-((4-Fluoro-2-styrylphenyl)ethynyl)(phenyl)sulfane (*E*-3o): yellow solid; mp = 30–32 ºC; R_f = 0.18 (hex/EtOAc, 100/1); 68% yield (449 mg); isolated as a > 20:1 mixture of *E*:*Z* isomers; ^{1}H NMR (300 MHz, $CDCl_3$) δ (ppm) 7.62–7.47 (m, 6H), 7.38–7.26 (m, 7H), 7.16 (d, *J* = 16.3 Hz, 1H), 6.95 (td, *J* = 8.3, 2.6 Hz, 1H); ^{13}C NMR (75.4 MHz, $CDCl_3$) δ (ppm) 164.5 (d, *J* = 249.2 Hz, C), 141.5 (d, *J* = 8.8 Hz, C), 136.7 (C), 134.6 (d, *J* = 8.8 Hz, CH), 133.0 (C), 131.9 (CH), 129.5 (2 × CH), 128.9 (2 × CH), 128.4 (CH), 127.1 (2 × CH), 126.8 (CH), 126.5 (2 × CH), 125.6 (d, *J* = 2.6 Hz, CH), 118.2 (d, *J* = 2.6 Hz, C), 114.9 (d, *J* = 22.6 Hz, CH), 111.4 (d, *J* = 22.8 Hz, CH), 95.5 (C), 80.4 (C); LRMS (EI) *m/z* (%) 330 (M^+, 15), 252 (25), 221 (100); HRMS (ESI) calcd for $C_{22}H_{16}FS$ $[M+H]^+$ 331.0951, found 331.0951.

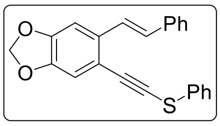

(*E*)-5-((Phenylthio)ethynyl)-6-styrylbenzo[*d*][1,3]dioxole (*E*-3p): brown solid; mp = 120–122 ºC; R_f = 0.20 (hex/EtOAc, 50/1); 72% yield (513 mg); isolated as a > 20:1 mixture of *E*:*Z* isomers; ^{1}H NMR (300 MHz, $CDCl_3$) δ (ppm) 7.65 (d, *J* = 16.3 Hz, 1H), 7.57–7.49 (m, 4H), 7.38–7.28 (m, 6H), 7.20 (s, 1H), 7.03 (d, *J* = 16.3 Hz, 1H), 6.98 (s, 1H), 6.03 (s, 2H); ^{13}C NMR (75.4 MHz, $CDCl_3$) δ (ppm) 149.0 (C), 147.1 (C), 137.3 (C), 135.0 (C), 133.3 (C), 129.5 (2 × CH), 129.2 (CH), 128.8 (2 × CH), 127.9 (CH), 126.8 (2 × CH),

126.7 (CH), 126.4 (3 × CH), 115.8 (C), 111.7 (CH), 104.4 (CH), 101.8 (CH_2), 96.5 (C), 78.2 (C); LRMS (EI) *m/z* (%) 356 (M^+, 87), 217 (100); HRMS (ESI) calcd for $C_{23}H_{17}O_2S$ $[M+H]^+$ 357.0944, found 357.0945.

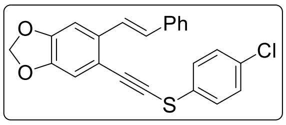

(*E*)-5-(((4-Chlorophenyl)thio)ethynyl)-6-styrylbenzo[*d*][1,3]dioxole (*E*-3q): yellow solid; mp = 165–167 ºC; R_f = 0.21 (hex/EtOAc, 50/1); 74% yield (577 mg); isolated as a > 20:1 mixture of *E*:*Z* isomers; ^{1}H NMR (300 MHz, $CDCl_3$) δ (ppm) 7.57 (d, *J* = 16.3 Hz, 1H), 7.48–7.26 (m, 9H), 7.15 (s, 1H), 6.99 (d, *J* = 16.3 Hz, 1H), 6.92 (s, 1H), 5.98 (s, 2H); ^{13}C NMR (75.4 MHz, $CDCl_3$) δ (ppm) 149.0 (C), 147.1 (C), 137.2 (C), 135.1 (C), 132.6 (C), 131.9 (C), 129.5 (2 × CH), 129.3 (CH), 128.8 (2 × CH), 127.9 (CH), 127.6 (2 × CH), 126.7 (CH), 126.2 (2 × CH), 115.4 (C), 111.6 (CH), 104.4 (CH), 101.7 (CH_2), 97.0 (C), 78.5 (C); LRMS (EI) *m/z* (%) 392 (M^++2, 10), 390 (M^+, 42), 217 (100), 189 (65); HRMS (ESI) could not be recorded.

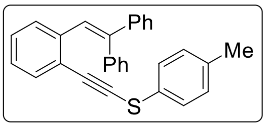

((2-(2,2-Diphenylvinyl)phenyl)ethynyl)(*p*-tolyl)sulfane (3r): yellow oil; R_f = 0.12 (hex/EtOAc, 100/1); 80% yield (627 mg); ^{1}H NMR (300 MHz, $CDCl_3$) δ (ppm) 7.48–7.45 (m, 1H), 7.40–7.31 (m, 11H), 7.24–7.21 (m, 2H), 7.11 (td, *J* = 7.6, 1.3 Hz, 1H), 6.96 (td, *J* = 7.6, 1.3 Hz, 1H), 6.86–6.82 (m, 3H), 2.27 (s, 3H); ^{13}C NMR (75.4 MHz, $CDCl_3$) δ (ppm) 144.4 (C), 143.3 (C), 140.2 (C), 139.3 (C), 136.5 (C), 131.4 (CH), 130.8 (2 × CH), 130.1 (2 × CH), 129.4 (CH), 129.2 (C), 128.5 (2 × CH), 128.4 (2 × CH), 128.3 (2 × CH), 127.8 (CH), 127.6 (CH), 127.4 (CH), 126.7 (CH), 126.6 (CH), 126.4 (2 × CH), 123.8 (C), 97.0 (C), 81.4 (C), 21.0 (CH_3); LRMS (EI) *m/z* (%) 402 (M^+, 2), 279 (100); HRMS (ESI) could not be recorded.

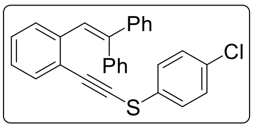

(4-Chlorophenyl)-((2-(2,2-diphenylvinyl)phenyl)ethynyl)sulfane (3s): yellow oil; R_f = 0.15 (hex/EtOAc, 100/1); 81% yield (687 mg); ^{1}H NMR (300 MHz, $CDCl_3$) δ (ppm) 7.46 (d, *J* = 7.7 Hz, 1H), 7.38–7.30 (m, 11H), 7.22–7.20 (m, 2H), 7.10 (t, *J* = 7.5 Hz, 1H), 7.00–6.91 (m, 3H), 6.85 (d, *J* = 7.7 Hz, 1H); ^{13}C NMR (75.4 MHz, $CDCl_3$) δ (ppm) 144.4 (C), 142.9 (C), 139.7 (C), 139.1 (C), 132.1 (C), 131.2 (C), 131.0 (CH), 130.5 (2 × CH), 129.2 (CH), 129.1 (2 × CH), 128.3 (2 × CH), 128.2 (2 × CH), 128.0 (2 × CH), 127.9 (CH), 127.4 (CH), 127.1 (2 × CH), 126.4 (CH), 126.3 (CH), 123.1 (C), 97.8 (C), 79.9 (C), one aromatic CH does not appear due to overlapping; LRMS (EI) *m/z* (%) 424 (M^++2, 4), 422 (M^+, 12), 279 (100); HRMS (ESI) could not be recorded.

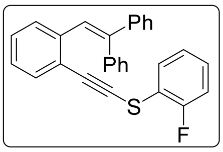

((2-(2,2-Diphenylvinyl)phenyl)ethynyl)-(2-fluorophenyl)sulfane (3t): pale yellow solid; mp = 113–115 ºC; R_f = 0.17 (hexane); 73% yield (593 mg); ^{1}H NMR (300 MHz, $CDCl_3$) δ (ppm) 7.60 (td, *J* = 7.8, 1.5 Hz, 1H), 7.50 (dd, *J* = 7.8, 1.5 Hz, 1H), 7.37–7.31 (m, 9H), 7.24–7.23 (m, 2H), 7.13–7.10 (m, 2H), 7.01–6.97 (m, 2H), 6.89–6.85 (m, 1H), 6.64 (td *J* = 7.6, 1.3 Hz, 1H); ^{13}C NMR (75.4 MHz, $CDCl_3$) δ (ppm) 158.8 (d, *J* = 245.5 Hz, C), 144.7 (C), 143.2 (C), 140.1 (C), 139.6 (C), 131.7 (CH), 130.8 (2 × CH), 129.5 (CH), 128.6 (2 × CH), 128.4 (2 × CH), 128.2 (2 × CH), 128.0 (d, *J* = 8.0 Hz, CH), 127.8 (d, *J* = 8.0 Hz, CH), 127.7 (CH), 126.7 (CH), 126.5 (CH), 125.1 (d, *J* = 3.3 Hz, CH), 123.3 (C), 120.5 (d, *J* = 16.2 Hz, C), 115.5 (d, *J* = 20.2 Hz, CH), 97.9 (C), 78.6 (d, *J* = 2.9 Hz, C), two aromatic CH do not appear due to overlapping; LRMS (EI) *m/z* (%) 406 (M^+, 8), 279 (100), 202 (16); HRMS (ESI) calcd for $C_{28}H_{20}FS$ $[M+H]^+$ 407.1264, found 407.1272.

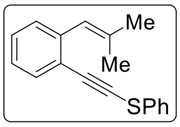

((2-(2-Methylprop-1-en-1-yl)phenyl)ethynyl)(phenyl)sulfane (3u): the spectroscopic data matches those reported in our previous work.[2] Light yellow oil; R_f = 0.31 (hex/EtOAc, 100/1); 72% yield (380 mg); ^{1}H NMR (300 MHz, $CDCl_3$) δ (ppm) 7.57–7.53 (m, 4H), 7.39–7.29 (m, 5H), 6.57 (s, 1H), 2.00 (d, J = 1.4 Hz, 3H), 1.87 (d, J = 1.4 Hz, 3H); ^{13}C NMR (75.4 MHz, $CDCl_3$) δ (ppm) 140.5 (C), 137.2 (CH), 133.4 (C), 131.8 (CH), 131.3 (C), 128.0 (CH), 127.6 (2 × CH), 127.3 (CH), 126.5 (CH), 126.2 (2 × CH), 124.0 (CH), 122.5 (C), 97.8 (C), 79.1 (C), 26.8 (CH_3), 19.7 (CH_3); LRMS (EI) *m/z* (%) 264 (M^+, 21), 172 (46), 121 (100); HRMS (ESI) calcd for $C_{18}H_{17}S^+$ $[M+H]^+$ 265.1045, found 265.1048.

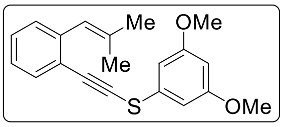

(3,5-Dimethoxyphenyl)-((2-(2-methylprop-1-en-1-yl)phenyl)ethynyl)sulfane (3v): yellow oil; R_f = 0.24 (hex/EtOAc, 100/1); 83% yield (538 mg); ^{1}H NMR (300 MHz, $CDCl_3$) δ (ppm) 7.51 (d, J = 7.6 Hz, 1H), 7.32–7.29 (m, 2H), 7.21–7.18 (m, 1H), 6.70 (d, J = 2.1 Hz, 2H), 6.53 (s, 1H), 6.34 (t, J = 2.1 Hz, 1H), 3.81 (s, 6H), 1.95 (s, 3H), 1.85 (s, 3H); ^{13}C NMR (75.4 MHz, $CDCl_3$) δ (ppm) 161.4 (2 × C), 140.7 (C), 137.4 (C), 135.4 (C), 132.2 (CH), 129.4 (CH), 128.2 (CH), 126.0 (CH), 123.8 (CH), 122.4 (C), 104.3 (2 × CH), 99.0 (CH), 98.3 (C), 78.8 (C), 55.6 (2 × CH_3), 26.7 (CH_3), 19.8 (CH_3); LRMS (EI) *m/z* (%) 324 (M^+, 20), 181 (100), 172 (68), 128 (74); HRMS (ESI) calcd for $C_{20}H_{21}O_2S$ $[M+H]^+$ 325.1257, found 325.1264.

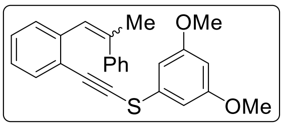

(3,5-Dimethoxyphenyl)-((2-(2-phenylprop-1-en-1-yl)phenyl)ethynyl)sulfane (3w): yellow oil; R_f = 0.23 (hex/EtOAc, 50/1); 82% yield (633 mg); obtained and isolated as a 1:1 mixture of *E*:*Z* isomers; data for both isomers; ^{1}H NMR (300 MHz, $CDCl_3$) δ (ppm) 7.59–7.17 (m, 17H, both diast), 6.98 (td, J = 7.6, 1.2 Hz, 1H, one diast), 6.89 (s, 1H, other diast), 6.84 (d, J = 7.6 Hz, 1H, one diast), 6.77 (d, J = 2.2 Hz, 2H, other diast), 6.71 (d, J = 2.2 Hz, 2H, one diast), 6.39 (t, J = 2.2 Hz, 1H, other diast), 6.34 (d, J = 2.2 Hz, 1H, one diast), 3.83 (s, 6H, other diast), 3.75 (s, 6H, one diast), 2.31 (d, J = 1.4 Hz, 3H, other diast), 2.29 (d, J = 1.4 Hz, 3H, one diast); ^{13}C NMR (75.4 MHz, $CDCl_3$) δ (ppm) 161.4 (2 × C), 161.3 (2 × C), 143.4 (C), 141.7 (C), 140.6 (C), 140.5 (C), 140.0 (C), 138.7 (C), 135.3 (C), 135.1 (C), 132.4 (CH), 132.1 (CH), 129.6 (CH), 129.4 (CH), 128.4 (4 × CH), 128.3 (CH), 127.8 (CH), 127.4 (CH), 127.1 (CH), 126.6 (CH), 126.2 (2 × CH), 126.0 (CH), 125.1 (CH), 122.9 (C), 122.4 (C), 104.3 (2 × CH), 104.2 (2 × CH), 99.3 (CH), 99.0 (CH), 98.1 (C), 97.7 (C), 79.8 (C), 79.3 (C), 55.5 (2 × CH_3), 55.4 (2 × CH_3), 26.6 (CH_3), 17.7 (CH_3), one CH and 2 × CH (aromatics) do not appear due to overlapping; LRMS (EI) *m/z* (%) 386 (M^+, 2), 234 (90), 202 (100), 181 (98); HRMS (ESI) calcd for $C_{25}H_{23}O_2S$ $[M+H]^+$ 387.1413, found 387.1415.

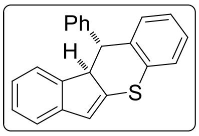

(10*bR,11*R**)-11-Phenyl-10*b*,11-dihydroindeno[2,1-*b*]thiochromene (4a):** brown solid; mp = 166–168 ºC; R_f = 0.22 (hex/EtOAc, 100/1); 81% yield (76 mg); obtained and isolated with d.r. > 20/1 starting from (*E*)-**3a**; ^{1}H NMR (300 MHz, $CDCl_3$) δ (ppm) 7.50–7.18 (m, 10H), 7.02 (td, J = 7.6, 3.2 Hz, 1H), 6.80–6.76 (m, 2H), 5.89 (d, J = 7.6 Hz, 1H), 3.86 (d, J = 13.5 Hz, 1H), 3.77 (d, J = 13.5 Hz, 1H); ^{13}C NMR (75.4 MHz, $CDCl_3$) δ (ppm) 144.4 (C), 143.5 (C), 142.5 (C), 139.9 (C), 135.7 (C), 131.4 (C), 130.6 (CH), 130.4 (CH), 129.0 (2 × CH), 127.7 (CH), 127.33 (CH), 127.29 (CH), 126.0 (CH), 125.3 (CH), 124.5 (CH), 123.8 (CH), 123.5 (CH), 119.6 (CH), 52.0 (CH), 50.0 (CH), one aromatic CH does not appear due to

overlapping; LRMS (EI) *m/z* 312 (M^+, 60), 235 (100); HRMS (ESI) calcd for $C_{22}H_{17}S$ $[M+H]^+$ 313.1045, found 313.1039.

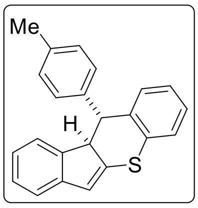

(10*bR,11*R**)-11-(*p*-Tolyl)-10*b*,11-dihydroindeno[2,1-*b*]thiochromene (4b):** yellow solid; mp = 169–171 ºC; R_f = 0.24 (hex/EtOAc, 100/1); 82% yield (80 mg); obtained and isolated with d.r. > 20/1 starting from (*E*)-**3b**; ^{1}H NMR (300 MHz, $CDCl_3$) δ (ppm) 7.35 (d, J = 7.7 Hz, 2H), 7.33–7.22 (m, 6H), 7.08–7.02 (m, 1H), 6.86 (m, 2H), 6.80 (s, 1H), 6.04 (d, J = 7.6 Hz, 1H), 3.86 (d, J = 13.4 Hz, 1H), 3.80 (d, J = 13.4 Hz, 1H), 2.56 (s, 3H); ^{13}C NMR (75.4 MHz, $CDCl_3$) δ (ppm) 144.3 (C), 143.6 (C), 139.9 (C), 139.4 (C), 137.1 (C), 135.9 (C), 131.4 (C), 130.6 (CH), 130.2 (CH), 129.7 (2 × CH), 127.24 (CH), 127.15 (CH), 125.9 (CH), 125.2 (CH), 124.5 (CH), 123.7 (CH), 123.4 (CH), 119.5 (CH), 51.9 (CH), 49.5 (CH), 21.4 (CH_3), one aromatic CH does not appear due to overlapping; LRMS (EI) *m/z* 326 (M^+, 60), 235 (100); HRMS (ESI) calcd for $C_{23}H_{19}S$ $[M+H]^+$] 327.1202, found 327.1198.

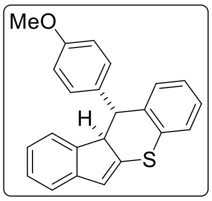

(10*bR,11*R**)-11-(4-Methoxyphenyl)-10*b*,11-dihydroindeno[2,1-*b*]thiochromene (4c):** yellow oil; R_f = 0.18 (hex/EtOAc, 100/1); 88% yield (90 mg); obtained and isolated with d.r. > 20/1 starting from (*E*)-**3c**; ^{1}H NMR (300 MHz, $CDCl_3$) δ (ppm) 7.30–7.17 (m, 6H), 7.07–7.03 (m, 3H), 6.86–6.81 (m, 2H), 6.77 (s, 1H), 6.02 (d, J = 7.0 Hz, 1H), 3.96 (s, 3H), 3.80 (d, J = 13.0 Hz, 1H), 3.76 (d, J = 13.0 Hz, 1H); ^{13}C NMR (75.4 MHz, $CDCl_3$) δ (ppm) 159.0 (C), 144.4 (C), 143.7 (C), 140.0 (C), 136.1 (C), 134.5 (C), 131.4 (CH), 131.3 (C), 130.6 (CH), 127.3 (CH), 127.2 (CH), 126.0 (CH), 125.3 (CH), 124.6 (CH), 123.7 (CH), 123.5 (CH), 119.5 (CH), 114.4 (CH), 55.5 (CH_3), 52.1 (CH), 49.2 (CH), two aromatic CH do not appear due to overlapping; LRMS (EI) *m/z* 342 (M^+, 100), 235 (90); HRMS (ESI) calcd for $C_{23}H_{19}OS$ $[M+H]^+$ 343.1151, found 343.1145.

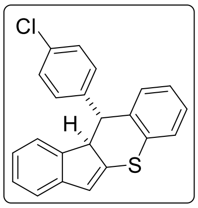

(10*bR,11*R**)-11-(4-Chlorophenyl)-10*b*,11-dihydroindeno[2,1-*b*]thiochromene (4d):** yellow oil; R_f = 0.17 (hexane); 80% yield (84 mg); obtained and isolated with d.r. > 20/1 starting from (*E*)-**3d**; ^{1}H NMR (300 MHz, $CDCl_3$) δ (ppm) 7.50 (d, J = 8.6 Hz, 2H), 7.32–7.21 (m, 6H), 7.06–7.01 (m, 1H), 6.88–6.83 (m, 1H), 6.78 (s, 1H), 6.74 (d, J = 7.9 Hz, 1H), 6.00 (d, J = 7.5 Hz, 1H), 3.81 (d, J = 13.4 Hz, 1H), 3.76 (d, J = 13.4 Hz, 1H); ^{13}C NMR (75.4 MHz, $CDCl_3$) δ (ppm) 144.3 (C), 143.2 (C), 141.2 (C), 139.5 (C), 135.1 (C), 133.4 (C), 131.7 (CH), 131.5 (C), 130.4 (CH), 129.3 (2 × CH), 127.50 (CH), 127.47 (CH), 126.2 (CH), 125.4 (CH), 124.3 (CH), 124.0 (CH), 123.6 (CH), 119.7 (CH), 51.8 (CH), 49.5 (CH), one aromatic CH does not appear due to overlapping; LRMS (EI) *m/z* 348 (M^++2, 8), 346 (M^+, 25), 235 (100); HRMS (ESI) calcd for $C_{22}H_{16}ClS$ $[M+H]^+$ 347.0656, found 347.0657.

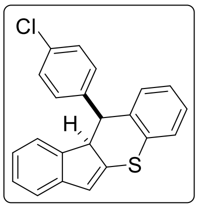

(10*bR,11*S**)-11-(4-Chlorophenyl)-10*b*,11-dihydroindeno[2,1-*b*]thiochromene (diast-4d):** yellow oil; R_f = 0.15 (hexane); 75% yield (78 mg); obtained with d.r. = 4/1 and isolated with d.r. > 20/1 starting from (*Z*)-**3d**; ^{1}H NMR (300 MHz, $CDCl_3$) δ (ppm) 7.53 (d, J = 7.1 Hz, 1H), 7.36–7.33 (m, 1H), 7.29–7.08 (m, 6H), 6.96–6.87 (m, 4H), 6.62 (s, 1H), 4.79 (d, J = 5.6 Hz, 1H), 3.98 (d, J = 5.6 Hz, 1H); ^{13}C NMR (75.4 MHz, $CDCl_3$) δ (ppm) 144.8 (C), 142.2 (C), 137.7 (C), 137.5 (C), 134.3 (C), 132.4 (C), 131.6 (CH), 131.5 (CH), 129.9 (2 × CH), 128.1 (CH), 127.7 (2 × CH), 127.4 (CH), 127.0 (CH), 126.0 (CH), 125.1 (CH), 123.6 (CH), 123.3 (CH), 119.9 (CH), 51.9 (CH), 47.6 (CH); LRMS (EI)

m/z 348 (M^++2, 6), 346 (M^+, 25), 235 (100); HRMS (APCI) calcd for $C_{22}H_{16}ClS$ $[M+H]^+$ 347.0656, found 347.0651.

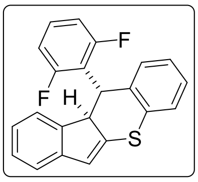

(10*bR,11*R**)-11-(2,6-Difluorophenyl)-10*b*,11-dihydroindeno[2,1-*b*]thiochromene (4e):** red solid; mp = 155–157 ºC; R_f = 0.15 (hex/DCM, 20/1); 87% yield (91 mg); obtained and isolated with d.r. > 20/1 starting from (*E*)-**3e**; ^{1}H NMR (300 MHz, $CDCl_3$) δ (ppm) 7.52–7.42 (m, 1H), 7.31–7.22 (m, 4H), 7.10–7.05 (m, 3H), 6.89–6.83 (m, 2H), 6.78 (s, 1H), 6.17 (d, *J* = 7.6 Hz, 1H), 4.41 (d, *J* = 13.0 Hz, 1H), 4.09 (d, *J* = 13.0 Hz, 1H); ^{13}C NMR (75.4 MHz, $CDCl_3$) δ (ppm) 162.5 (dd, *J* = 250.7, 10.1 Hz, C), 162.2 (dd, *J* = 250.7, 10.1 Hz, C), 144.5 (C), 143.2 (C), 139.9 (C), 133.0 (C), 131.1 (C), 129.7 (t, *J* = 10.1 Hz, CH), 128.0 (CH), 127.6 (CH), 127.5 (CH), 126.5 (CH), 125.7 (CH), 124.1 (CH), 123.9 (CH), 123.2 (CH), 119.9 (CH), 117.8 (t, *J* = 17.3 Hz, C), 112.4 (dd, *J* = 21.6, 3.5 Hz, CH), 111.9 (dd, *J* = 21.6, 3.5 Hz, CH), 48.9 (d, *J* = 3.5 Hz, CH), 39.4 (d, *J* = 3.5 Hz, CH); LRMS (EI) *m/z* 348 (M^+, 70), 235 (100); HRMS (ESI) calcd for $C_{22}H_{15}F_2S$ $[M+H]^+$ 349.0857, found 349.0854.

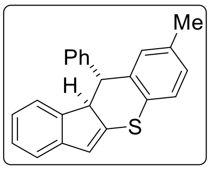

(10*bR,11*R**)-2-Methyl-11-phenyl-10*b*,11-dihydroindeno[2,1-*b*]thiochromene (4f):** yellow oil; R_f = 0.22 (hexane); 85% yield (83 mg); obtained and isolated with d.r. > 20/1 starting from (*E*)-**3f**; ^{1}H NMR (300 MHz, $CDCl_3$) δ (ppm) 7.50–7.48 (m, 3H), 7.36–7.33 (m, 2H), 7.24–7.15 (m, 3H), 7.02–7.00 (m, 1H), 6.79–6.73 (m, 2H), 6.60 (s, 1H), 5.85 (d, *J* = 7.6 Hz, 1H), 3.82 (d, *J* = 13.4 Hz, 1H), 3.75 (d, *J* = 13.4 Hz, 1H), 2.17 (s, 3H); ^{13}C NMR (75.4 MHz, $CDCl_3$) δ (ppm) 144.5 (C), 143.5 (C), 142.6 (C), 140.3 (C), 135.6 (C), 135.0 (C), 131.2 (CH), 130.5 (C), 129.0 (2 × CH), 128.2 (CH), 127.8 (C), 127.6 (CH), 127.3 (CH), 125.9 (CH), 124.5 (CH), 123.5 (CH), 123.3 (CH), 119.4 (CH), 52.3 (CH), 50.0 (CH), 21.2 (CH_3), one aromatic CH does not appear due to overlapping; LRMS (EI) *m/z* 326 (M^+, 60), 249 (100), 234 (30); HRMS (ESI) calcd for $C_{23}H_{19}S$ $[M+H]^+$ 327.1202, found 327.1201.

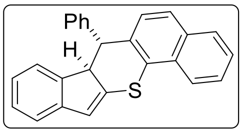

(7*S,7*aR**)-7-Phenyl-7,7*a*-dihydrobenzo[*h*]indeno[2,1-*b*]thiochromene (4g):** yellow solid; mp = 98–100 ºC; R_f = 0.23 (hexane); 83% yield (90 mg); obtained and isolated with d.r. > 20/1 starting from (*E*)-**3g**; ^{1}H NMR (300 MHz, $CDCl_3$) δ (ppm) 8.29 (d, *J* = 8.5 Hz, 1H), 7.84–7.65 (m, 3H), 7.56–7.35 (m, 7H), 7.22–7.05 (m, 4H), 6.23 (s, 1H), 3.71 (d, *J* = 21.8 Hz, 1H), 3.57 (d, *J* = 21.8 Hz, 1H); ^{13}C NMR (75.4 MHz, $CDCl_3$) δ (ppm) 143.82 (C), 143.80 (C), 142.5 (C), 135.2 (C), 133.1 (C), 132.4 (C), 130.9 (C), 129.2 (C), 128.9 (CH), 128.72 (CH), 128.68 (2 × CH), 128.1 (2 × CH), 127.7 (CH), 127.0 (CH), 126.8 (CH), 126.6 (CH), 125.42 (CH), 125.39 (CH), 124.3 (CH), 123.6 (CH), 122.9 (CH), 117.6 (CH), 40.9 (CH), 40.1 (CH); LRMS (EI) *m/z* 362 (M^+, 60), 235 (100); HRMS (ESI) calcd for $C_{26}H_{18}NaS$ $[M+Na]^+$ 385.1021, found 385.1018.

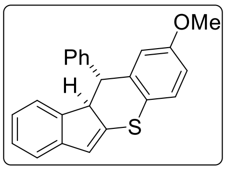

(10*bR,11*R**)-2-Methoxy-11-phenyl-10*b*,11-dihydroindeno[2,1-*b*]thiochromene (4h):** white solid; mp = 190–192 ºC; R_f = 0.10 (hex/EtOAc, 100/1); 87% yield (89 mg); obtained and isolated with d.r. > 20/1 starting from (*E*)-**3h**; ^{1}H NMR (300 MHz, $CDCl_3$) δ (ppm) 7.48–7.42 (m, 3H), 7.34–7.26 (m, 2H), 7.19–7.14 (m, 3H), 6.78–6.71 (m, 3H), 6.35 (s, 1H), 5.85 (d, *J* = 7.6 Hz, 1H), 3.79 (d, *J* = 13.7 Hz, 1H), 3.73 (d, *J* = 13.7 Hz, 1H), 3.60 (s, 3H); ^{13}C NMR (75.4 MHz, $CDCl_3$) δ (ppm) 157.6 (C), 144.6 (C), 143.3 (C), 142.2 (C), 140.5 (C), 137.4 (C), 130.4 (CH), 129.1 (2 × CH), 127.8 (CH), 127.3 (CH), 126.8 (CH), 124.5 (CH), 123.3 (2 × CH), 122.2 (C), 119.4 (CH), 117.1 (CH), 112.9

(CH), 55.4 (CH_3), 52.1 (CH), 50.2 (CH), one aromatic CH does not appear due to overlapping; LRMS (EI) *m/z* 342 (M^+, 74), 265 (100); HRMS (ESI) calcd for $C_{23}H_{19}OS$ $[M+H]^+$ 343.1151, found 343.1146.

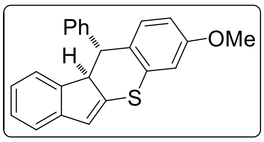

(10*bR*,11*R*)-3-Methoxy-11-phenyl-10*b*,11-dihydroindeno[2,1-*b*]thiochromene (4i): orange solid; mp = 161–163 ºC: R_f = 0.23 (hex/EtOAc, 100/1); 77% yield (79 mg); obtained as a 6/1 mixture of regioisomers starting from (*E*)-**3i** and isolated as a 7/1 mixture, data for the major regioisomer: ^{1}H NMR (300 MHz, $CDCl_3$) δ (ppm) 7.50–7.47 (m, 2H), 7.38–7.18 (m, 6H), 6.81–6.76 (m, 2H), 6.67 (d, *J* = 8.7 Hz, 1H), 6.56 (dd, *J* = 8.7, 2.5 Hz, 1H), 5.90 (d, *J* = 7.6 Hz, 1H), 3.81 (s, 3H), 3.80 (d, *J* = 13.1 Hz, 1H), 3.72 (d, *J* = 13.1 Hz, 1H); ^{13}C NMR (75.4 MHz, $CDCl_3$) δ (ppm) 158.5 (C), 144.3 (C), 143.7 (C), 142.8 (C), 139.9 (C), 132.4 (C), 131.6 (CH), 130.4 (C), 129.0 (2 × CH), 128.1 (CH), 127.7 (CH), 127.3 (CH), 124.5 (CH), 123.9 (CH), 123.5 (CH), 119.6 (CH), 111.6 (CH), 110.6 (CH), 55.5 (CH_3), 52.5 (CH), 49.4 (CH), one aromatic CH does not apear due to overlapping; LRMS (EI) *m/z* 342 (M^+, 40), 265 (100); HRMS (ESI) calcd for $C_{23}H_{19}OS$ $[M+H]^+$ 343.1151, found 343.1146.

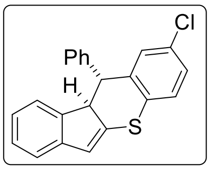

(10*bR,11*R**)-2-Chloro-11-phenyl-10*b*,11-dihydroindeno[2,1-*b*]thiochromene (4j):** yellow oil; R_f = 0.15 (hexane); 82% yield (85 mg); obtained and isolated with d.r. = 20/1 starting from (*E*)-**3j**, data for major diastereoisomer: ^{1}H NMR (300 MHz, $CDCl_3$) δ (ppm) 7.50–7.47 (m, 3H), 7.32–7.15 (m, 6H), 6.80–6.75 (m, 3H), 5.84 (d, *J* = 7.6 Hz, 1H), 3.76 (d, *J* = 13.4 Hz, 1H), 3.70 (d, *J* = 13.4 Hz, 1H); ^{13}C NMR (75.4 MHz, $CDCl_3$) δ (ppm) 144.2 (C), 143.2 (C), 141.6 (C), 139.0 (C), 137.6 (C), 130.9 (C), 130.5 (CH), 130.3 (CH), 130.0 (C), 129.3 (2 × CH), 128.0 (CH), 127.5 (CH), 127.4 (CH), 127.1 (CH), 124.5 (CH), 124.4 (CH), 123.7 (CH), 119.7 (CH), 51.6 (CH), 49.9 (CH), one aromatic CH do not appear due to overlapping; LRMS (EI) *m/z* 348 (M^++2, 20), 346 (M^+, 50), 269 (100), 234 (67), 207 (35); HRMS (ESI) could not be recorded.

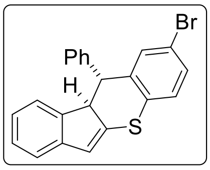

(10*bR,11*R**)-2-Bromo-11-phenyl-10*b*,11-dihydroindeno[2,1-*b*]thiochromene (4k):** yellow solid; mp = 150–152 ºC: R_f = 0.20 (hex/EtOAc, 100/1); 88% yield (103 mg); obtained and isolated with d.r. > 20/1 starting from (*E*)-**3k**; ^{1}H NMR (300 MHz, $CDCl_3$) δ (ppm) 7.52–7.50 (m, 3H), 7.34–7.12 (m, 6H), 6.90 (d, *J* = 2.0 Hz, 1H), 6.81–6.76 (m, 2H), 5.85 (d, *J* = 7.6 Hz, 1H), 3.78 (d, *J* = 13.3 Hz, 1H), 3.74 (d, *J* = 13.3 Hz, 1H); ^{13}C NMR (75.4 MHz, $CDCl_3$) δ (ppm) 144.2 (C), 143.3 (C), 141.6 (C), 138.9 (C), 138.0 (C), 133.3 (CH), 130.8 (C), 130.3 (2 × CH), 129.3 (2 × CH), 128.1 (CH), 127.5 (CH), 127.4 (CH), 124.5 (CH), 124.4 (CH), 123.7 (CH), 119.7 (CH), 118.7 (C), 51.7 (CH), 49.9 (CH), one aromatic CH does not appear due to overlapping; LRMS (EI) *m/z* 394 (M^++2, 61), 392 (M^++2, 61), 390 (M^+, 62), 313 (100), 234 (89), 207 (40); HRMS (ESI) calcd for $C_{22}H_{16}BrS$ $[M+H]^+$ 391.0151, found 391.0149.

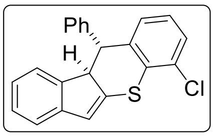

(10*bR,11*R**)-4-Chloro-11-phenyl-10*b*,11-dihydroindeno[2,1-*b*]thiochromene (4l):** white solid; mp = 46–48 ºC; R_f = 0.26 (hex/EtOAc, 100/1); 83% yield (86 mg); obtained and isolated with d.r. > 20/1 starting from de (*E*)-**3l**; ^{1}H NMR (300 MHz, $CDCl_3$) δ (ppm) 7.53–7.51 (m, 3H), 7.36–7.22 (m, 5H), 6.97 (t, *J* = 7.9 Hz, 1H), 6.87 (s, 1H), 6.86–6.81 (m, 1H), 6.73 (d, *J* = 7.9 Hz, 1H), 5.92 (d, *J* = 7.6 Hz, 1H), 3.81 (d, *J* = 13.5 Hz, 1H), 3.76 (d, *J* = 13.5 Hz, 1H); ^{13}C NMR (75.4 MHz, $CDCl_3$) δ (ppm) 144.0 (C), 143.5 (C), 142.4 (C), 139.0 (C), 138.0 (C), 131.3 (C), 130.5 (C), 130.3 (CH), 129.1 (2 × CH), 129.0 (CH), 128.0 (CH), 127.8 (CH), 127.5 (CH), 125.4 (CH), 125.2 (CH), 124.5 (CH), 123.8 (CH), 119.9 (CH), 51.3 (CH), 50.5 (CH),

one aromatic CH does not appear due to overlapping; LRMS (EI) *m/z* 348 (M^++2, 28), 346 (M^+, 88), 269 (100), 234 (67); HRMS (ESI) calcd for $C_{22}H_{16}ClS$ $[M+H]^+$ 347.0656, found 347.0651.

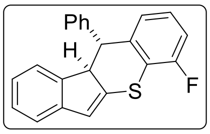

(10*bR,11*R**)-4-Fluoro-11-phenyl-10*b*,11-dihydroindeno[2,1-*b*]thiochromene (4m):** red solid; mp = 144–146 ºC; R_f = 0.23 (hex/EtOAc, 100/1); 78% yield (77 mg); obtained and isolated with d.r. > 20/1 starting from (*E*)-**3m**; ^{1}H NMR (300 MHz, $CDCl_3$) δ (ppm) 7.51–7.48 (m, 3H), 7.36–7.20 (m, 4H), 7.00–6.96 (m, 2H), 6.86 (s, 1H), 6.81 (td, *J* = 7.5, 1.3 Hz, 1H), 6.60–6.57 (m, 1H), 5.91 (d, *J* = 7.5 Hz, 1H), 3.81 (d, *J* = 13.4 Hz, 1H), 3.77 (d, *J* = 13.4 Hz, 1H); ^{13}C NMR (75.4 MHz, $CDCl_3$) δ (ppm) 157.9 (d, *J* = 243.1 Hz, C), 144.0 (C), 143.6 (C), 142.3 (C), 138.2 (d, *J* = 1.9 Hz, C), 137.7 (d, *J* = 2.5 Hz, C), 130.3 (CH), 129.1 (2 x CH), 127.9 (CH), 127.4 (CH), 126.1 (d, *J* = 2.8 Hz, CH), 125.43 (d, *J* = 8.3 Hz, CH), 125.41 (CH), 124.5 (CH), 123.8 (CH), 119.8 (CH), 113.3 (d, *J* = 20.7 Hz, CH), 51.5 (CH), 50.1 (d, *J* = 2.4 Hz, CH), one aromatic CH and one aromatic C do not appear due to overlapping; LRMS (EI) *m/z* 330 (M^+, 64), 253 (100); HRMS (ESI) calcd for $C_{22}H_{16}FS$ $[M+H]^+$ 331.0951, found 331.0946.

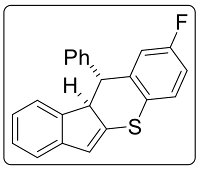

(10*bR,11*R**)-2-Bromo-11-phenyl-10*b*,11-dihydroindeno[2,1-*b*]thiochromene (4n):** colorless solid; mp = 161–163 ºC; R_f = 0.22 (hex/ DCM, 10/1); 79% yield (78 mg); obtained and isolated with d.r. > 20/1 starting from (*E*)-**3n**; ^{1}H NMR (300 MHz, $CDCl_3$) δ (ppm) 7.53–7.50 (m, 3H), 7.35–7.19 (m, 5H), 6.93 (td, *J* = 8.2, 2.5 Hz, 1H), 6.81–6.76 (m, 2H), 6.53 (dd, *J* = 8.2, 2.5 Hz, 1H), 5.89 (d, *J* = 7.6 Hz, 1H), 3.80 (d, *J* = 13.3 Hz, 1H), 3.75 (d, *J* = 13.3 Hz, 1H); ^{13}C NMR (75.4 MHz, $CDCl_3$) δ (ppm) 160.8 (d, *J* = 243.9 Hz, C), 144.3 (C), 143.2 (C), 141.8 (C), 139.5 (C), 138.1 (d, *J* = 6.3 Hz, C), 130.3 (CH), 129.3 (2 × CH), 128.0 (CH), 127.5 (CH), 127.1 (d, *J* = 7.8 Hz, CH), 126.4 (d, *J* = 3.0 Hz, C), 124.5 (CH), 124.0 (CH), 123.6 (CH), 119.6 (CH), 117.7 (d, J = 22.4 Hz, CH), 114.7 (d, *J* = 22.4 Hz, CH), 51.7 (CH), 50.1 (CH), one aromatic CH does not appear due to overlapping; LRMS (EI) *m/z* 330 (M^+, 44), 253 (100); HRMS (APCI) calcd for $C_{22}H_{16}FS$ $[M+H]^+$ 331.0951, found 331.0952.

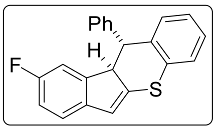

(10*bR,11*S**)-9-Fluoro-11-phenyl-10*b*,11-dihydroindeno[2,1-*b*]thiochromene (4o):** brown solid; mp = 133–135 ºC; R_f = 0.23 (hex/EtOAc, 100/1); 77% yield (76 mg); obtained and isolated with d.r. > 20/1 starting from (*E*)-**3o**; ^{1}H NMR (300 MHz, $CDCl_3$) δ (ppm) 7.52–7.49 (m, 3H), 7.35–7.32 (m, 2H), 7.28–7.11 (m, 3H), 7.04–6.98 (m, 1H), 6.92–6.88 (m, 1H), 6.78 (d, *J* = 7.8 Hz, 1H), 6.70 (s, 1H), 5.57 (dd, *J* = 9.6, 2.3 Hz, 1H), 3.81 (d, *J* = 13.3 Hz, 1H), 3.77 (d, *J* = 13.3 Hz, 1H); ^{13}C NMR (75.4 MHz, $CDCl_3$) δ (ppm) 160.2 (d, *J* = 242.0 Hz, C), 145.5 (d, *J* = 8.6 Hz, C), 142.0 (C), 140.3 (d, *J* = 2.3 Hz, C), 139.4 (d, *J* = 4.0 Hz, C), 135.5 (C), 131.3 (C), 130.7 (CH), 130.3 (CH), 129.2 (2 x CH), 128.0 (CH), 127.4 (CH), 126.1 (CH), 125.4 (CH), 123.0 (CH), 119.8 (d, *J* = 8.5 Hz, CH), 114.0 (d, *J* = 22.9 Hz, CH), 112.7 (d, *J* = 22.9 Hz, CH), 52.2 (d, *J* = 2.4 Hz, CH), 50.0 (CH), one aromatic CH do not appear due to overlapping; LRMS (EI) *m/z* 330 (M^+, 45), 253 (100); HRMS (ESI) calcd for $C_{22}H_{16}FS$ $[M+H]^+$ 331.0951, found 331.0947.

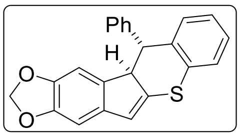

(11*R,11*aR**)-11-Phenyl-11,11*a*-dihydrothiochromeno[3',2':1,2]indeno[5,6-*d*][1,3]dioxole (4p):** brown solid; mp = 210–212 ºC; R_f = 0.21 (hex/EtOAc, 100/1); 80% yield (85 mg); obtained and isolated with d.r. > 20/1 starting from (*E*)-**3p**; ^{1}H NMR (300 MHz, $CDCl_3$) δ (ppm) 7.49–7.43 (m, 3H), 7.31–7.29 (m, 2H), 7.22 (d, *J* = 7.8 Hz, 1H), 7.15 (t, *J* = 7.5 Hz, 1H), 6.96 (t, *J* = 7.5 Hz, 1H), 6.74 (d, *J* = 7.8 Hz, 1H), 6.70 (s, 1H), 6.60 (s, 1H), 5.82 (d, *J* = 13.3 Hz, 2H), 5.35 (s, 1H), 3.73 (d, *J* = 13.0 Hz, 1H), 3.64 (d, *J*

= 13.0 Hz, 1H); ^{13}C NMR (75.4 MHz, $CDCl_3$) δ (ppm) 147.0 (C), 144.4 (C), 142.4 (CH), 138.4 (C), 137.9 (C), 139.7 (C), 135.4 (C), 131.5 (C), 130.6 (CH), 130.4 (C), 129.1 (2 × CH), 127.8 (CH), 127.3 (CH), 126.0 (CH), 125.2 (CH), 123.6 (CH), 106.5 (CH_2), 110.9 (CH), 110.8 (CH), 51.7 (CH), 50.3 (CH), one aromatic CH does not appear due to overlapping; LRMS (EI) *m/z* 356 (M^+, 97), 279 (100); HRMS (ESI) calcd for $C_{23}H_{16}O_2S$ $[M]^+$ 356.0866, found 356.0869.

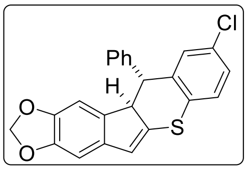

(11*R,11*aR**)-9-Chloro-11-phenyl-11,11*a*-dihydrothiochromeno[3',2':1,2]indeno[5,6-*d*][1,3]dioxole (4q):** brown solid; mp = 173–175 ºC; R_f = 0.20 (hex/EtOAc, 100/1); 76% yield (89 mg); obtained and isolated with d.r. > 20/1 starting from (*E*)-**3q**; ^{1}H NMR (300 MHz, $CDCl_3$) δ (ppm) 7.49–7.47 (m, 3H), 7.36–7.27 (m, 2H), 7.22–7.10 (m, 2H), 6.71–6.70 (m, 2H), 6.60 (s, 1H), 5.83 (d, *J* = 9.3 Hz, 2H), 5.30 (s, 1H), 3.72 (d, *J* = 13.0 Hz, 1H), 3.64 (d, *J* = 13.0 Hz, 1H); ^{13}C NMR (75.4 MHz, $CDCl_3$) δ (ppm) 147.1 (C), 144.6 (C), 141.6 (CH), 138.3 (C), 137.44 (CH), 137.41 (C), 137.0 (C), 130.8 (C), 130.5 (CH), 130.3 (C), 130.2 (C), 129.4 (2 × CH), 129.2 (C), 128.3 (CH), 127.5 (CH), 127.1 (CH), 124.2 (CH), 106.5 (CH_2), 101.02 (CH), 100.98 (CH), 51.3 (CH), 50.3 (CH); LRMS (EI) *m/z* 392 (M^++2, 12), 390 (M^+, 38), 217 (100); HRMS (ESI) calcd for $C_{23}H_{16}ClO_2S$ $[M+H]^+$ 391.0554, found 391.0556.

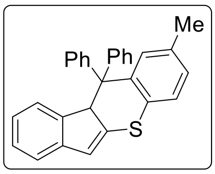

2-Methyl-11,11-diphenyl-10*b*,11-dihydroindeno[2,1-*b*]thiochromene (4r): colorless solid; mp = 254–256 ºC; R_f = 0.12 (hexane); 84% yield (101 mg); obtained and isolated as a single isomer; ^{1}H NMR (300 MHz, $CDCl_3$) δ (ppm) 7.44–7.03 (m, 12H), 6.80 (td, *J* = 7.5, 1.5 Hz, 1H), 6.64 (d, *J* = 1.0 Hz, 1H), 6.49–6.44 (m, 3H), 6.09 (d, *J* = 7.6 Hz, 1H), 4.58 (s, 1H), 2.24 (s, 3H); ^{13}C NMR (75.4 MHz, $CDCl_3$) δ (ppm) 145.8 (C), 145.2 (C), 142.7 (C), 141.8 (C), 141.1 (C), 139.5 (C), 135.2 (C), 132.2 (CH), 130.6 (CH), 129.1 (CH), 128.8 (C), 128.5 (CH), 128.3 (2 × CH), 127.7 (2 × CH), 127.4 (CH), 127.0 (CH), 126.8 (CH), 124.0 (CH), 123.4 (CH), 122.0 (CH), 119.5 (CH), 58.5 (CH), 57.2 (C), 21.4 (CH_3), three aromatic CH do not appear due to overlapping; LRMS (EI) *m/z* 402 (M^+, 28), 325 (100); HRMS (ESI) calcd for $C_{29}H_{22}NaS$ $[M+Na]^+$ 425.1334, found 425.1330.

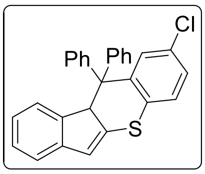

2-Chloro-11,11-diphenyl-10*b*,11-dihydroindeno[2,1-*b*]thiochromene (4s): orange solid; mp = 167–169 ºC; R_f = 0.18 (hexane); 82% yield (104 mg); obtained and isolated as a single isomer; ^{1}H NMR (300 MHz, $CDCl_3$) δ (ppm) 7.46–7.45 (m, 3H), 7.26–7.17 (m, 7H), 7.04 (t, *J* = 7.4 Hz, 2H), 6.81–6.80 (m, 2H), 6.47–6.43 (m, 3H), 6.10 (d, *J* = 7.6 Hz, 1H), 4.56 (s, 1H); ^{13}C NMR (75.4 MHz, $CDCl_3$) δ (ppm) 145.6 (C), 144.4 (C), 143.9 (C), 142.4 (C), 139.7 (C), 138.8 (C), 131.4 (CH), 131.2 (C), 131.0 (C), 130.6 (CH), 128.9 (CH), 128.7 (2 × CH), 128.0 (CH), 127.9 (4 × CH), 127.8 (CH), 127.6 (CH), 127.3 (CH), 124.0 (CH), 123.8 (CH), 122.8 (CH), 119.8 (CH), 57.9 (CH), 57.3 (C), one aromatic CH does not appear due to overlapping; LRMS (EI) *m/z* 424 (M^++2, 8), 422 (M^+, 28), 345 (100), 310 (86); HRMS (ESI) calcd for $C_{28}H_{20}ClS$ $[M+H]^+$ 423.0969, found 423.0962.

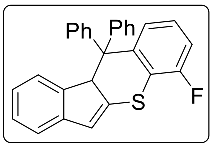

4-Fluoro-11,11-diphenyl-10*b*,11-dihydroindeno[2,1-*b*]thiochromene (4t): yellow solid; mp = 224–226 ºC; R_f = 0.15 (hex/EtOAc, 100/1); 70% yield (85 mg); obtained and isolated along with trace amounts of regioisomer **4't** (from isomerization of double bond); data for **4t**: ^{1}H NMR (300 MHz, $CDCl_3$) δ 7.45–7.06 (m, 12H), 6.85–6.82 (m, 1H), 6.54–6.53 (m, 1H), 6.47–6.43 (m, 3H), 6.18 (d, *J* = 7.6 Hz, 1H), 4.61

(s, 1H); ^{13}C NMR (75.4 MHz, $CDCl_3$) δ (ppm) 158.3 (d, J = 243.6 Hz, C), 145.5 (C), 144.9 (C), 142.5 (C), 139.0 (C), 131.4 (CH), 130.5 (2 × CH), 129.8 (d, J = 8.4 Hz, C), 128.7 (CH), 128.5 (2 × CH), 128.2 (CH), 127.8 (2 × CH), 127.6 (2 × CH), 127.2 (CH), 127.1 (d, J = 2.3 Hz, CH), 125.5 (d, J = 8.4 Hz, CH), 124.0 (CH), 123.9 (CH), 123.7 (CH), 119.8 (CH), 115.9 (d, J = 21.1 Hz, C), 114.0 (d, J = 21.1 Hz, CH), 57.6 (CH), 57.3 (d, J = 2.3 Hz, C); LRMS (EI) *m/z* 406 (M^+, 27), 329 (100); HRMS (ESI) calcd for $C_{28}H_{19}FNaS$ $[M+Na]^+$ 429.1084, found 429.1080.

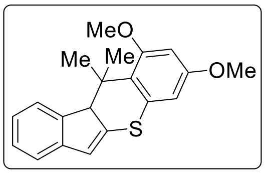

1,3-Dimethoxy-11,11-dimethyl-10*b*,11-dihydroindeno[2,1-*b*]thiochromene (4v): orange oil; R_f = 0.10 (hex/EtOAc, 50/1); 52% yield (50 mg); obtained and isolated as a mixture of products **4v/7v** = ~2/1 (77% overall yield); data for product **4v**: ^{1}H NMR (300 MHz, $CDCl_3$) δ (ppm) 7.75–7.72 (m, 1H), 7.29–7.16 (m, 3H), 6.72 (dd, J = 1.5, 0.6 Hz, 1H), 6.37–6.35 (m, 2H), 3.86 (s, 3H), 3.83 (s, 3H), 3.58 (s, 1H), 2.15 (s, 3H), 0.89 (s, 3H); ^{13}C NMR (75.4 MHz, $CDCl_3$) δ (ppm) 161.1 (C), 160.6 (C), 158.7 (C), 145.7 (C), 144.3 (C), 138.5 (C), 132.6 (C), 127.2 (CH), 125.2 (CH), 123.4 (CH), 119.8 (CH), 110.7 (CH), 102.0 (CH), 98.1 (CH), 61.0 (C), 59.5 (CH), 55.6 (CH_3), 55.3 (CH_3), 20.2 (CH_3), 16.8 (CH_3); LRMS (EI) *m/z* 324 (M^+, 68), 309 (100), 294 (40); HRMS (ESI) calcd for $C_{20}H_{21}O_2S$ $[M+H]^+$ 325.1257, found 325.1260.

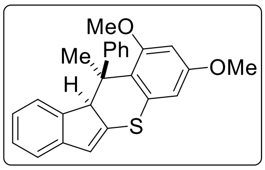

1,3-Dimethoxy-11-methyl-11-phenyl-10b,11-dihydroindeno[2,1-*b*]thiochromene (4w): yellow solid; mp = 143–145 ºC; R_f = 0.10 (hex/EtOAc, 100/1); 79% yield (91 mg); obtained and isolated as a single regio and diastereoisomer starting from a 2/1 mixture of (*E*/*Z*)-**3w**; ^{1}H NMR (300 MHz, $CDCl_3$) δ (ppm) 7.71 (d, J = 7.5 Hz, 1H), 7.09–6.98 (m, 3H), 6.93–6.88 (m, 5H), 6.60 (d, J = 1.5 Hz, 1H), 6.44 (d, J = 2.5 Hz, 1H), 6.28 (d, J = 2.5 Hz, 1H), 3.84 (s, 3H), 3.69 (s, 1H), 3.47 (s, 3H), 2.50 (s, 3H); ^{13}C NMR (75.4 MHz, $CDCl_3$) δ (ppm) 160.5 (C), 159.1 (C), 144.9 (C), 144.1 (C), 143.6 (C), 136.7 (C), 133.0 (C), 126.9 (CH), 126.4 (4 × CH), 125.4 (CH), 125.1 (CH), 124.9 (CH), 123.2 (CH), 122.7 (C), 119.8 (CH), 102.0 (CH), 99.0 (CH), 59.7 (CH), 55.7 (CH_3), 55.5 (CH_3), 47.0 (C), 26.9 (CH_3); LRMS (EI) *m/z* 386 (M^+, 100), 371 (96), 207 (60); HRMS (ESI) calcd for $C_{25}H_{23}O_2S$ $[M+H]^+$ 387.1413, found 387.1416.

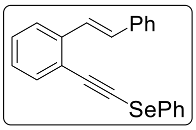

(*E*)-Phenyl((2-styrylphenyl)ethynyl)selane (*E*-5a): orange solid; mp = 52–54 ºC; R_f = 0.21 (hex/EtOAc, 100/1); 82% yield (590 mg); isolated as a > 20:1 mixture of *E*:*Z* isomers; ^{1}H NMR (300 MHz, $CDCl_3$) δ (ppm) 7.78–7.70 (m, 4H), 7.57–7.53 (m, 3H), 7.43–7.22 (m, 9H); ^{13}C NMR (75.4 MHz, $CDCl_3$) δ (ppm) 138.9 (C), 137.3 (C), 132.6 (CH), 130.7 (CH), 129.8 (2 × CH), 129.3 (2 × CH), 129.1 (C), 128.8 (2 × CH), 128.7 (CH), 128.0 (CH), 127.32 (CH), 127.27 (CH), 126.9 (2 × CH), 126.7 (CH), 124.8 (CH), 122.3 (C), 101.7 (C), 74.6 (C); LRMS (EI) *m/z* (%) 360 (M^+, 2), 279 (12), 203 (100); HRMS (ESI) calcd for $C_{22}H_{17}Se$ $[M+H]^+$ 361.0491, found 361.0499.

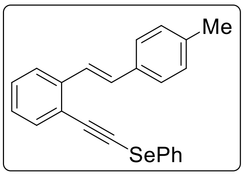

(*E*)-(2-(4-Methylstyryl)phenyl)ethynyl)(phenyl)selane (*E*-5b): yellow oil; R_f = 0.20 (hex/EtOAc, 100/1); 83% yield (621 mg); isolated as a > 20:1 mixture of *E*:*Z* isomers; ^{1}H NMR (300 MHz, $CDCl_3$) δ (ppm) 7.81–7.75 (m, 3H), 7.64 (dd, J = 7.7, 1.3 Hz, 1H), 7.54–7.25 (m, 11H), 2.49 (s, 3H); ^{13}C NMR (75.4 MHz, $CDCl_3$) δ (ppm) 138.9 (C), 137.7 (C), 134.4 (C), 132.5 (CH), 130.5 (CH), 129.7 (2 × CH), 129.4 (2 × CH), 129.11 (2 × CH), 129.05 (C), 128.6 (CH), 127.1 (CH), 127.0 (2 × CH), 126.8

(CH), 125.4 (CH), 124.6 (CH), 122.0 (C), 101.8 (C), 74.4 (C), 21.4 (CH_3); LRMS (EI) *m/z* (%) 374 (M^+, 10), 343 (20), 202 (100); HRMS (ESI) could not be recorded.

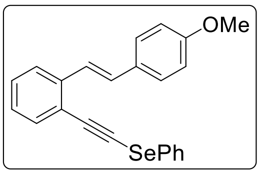

(*E*)-((2-(4-Methoxystyryl)phenyl)ethynyl)(phenyl)selane (*E*-5c): yellow solid; mp = 82–84 ºC; R_f = 0.18 (hex/EtOAc, 50/1); 85% yield (663 mg); isolated as a > 20:1 mixture of *E*:*Z* isomer; ^{1}H NMR (300 MHz, $CDCl_3$) δ (ppm) 7.73–7.67 (m, 3H), 7.57–7.52 (m, 2H), 7.45 (d, *J* = 8.9 Hz, 2H), 7.36–7.30 (m, 4H), 7.24 (td, *J* = 7.5, 1.2 Hz, 1H), 7.17 (d, *J* = 16.3 Hz, 1H), 6.91 (d, *J* = 8.9 Hz, 2H), 3.87 (s, 3H); ^{13}C NMR (75.4 MHz, $CDCl_3$) δ (ppm) 159.6 (C), 139.2 (C), 132.6 (CH), 130.2 (CH), 130.1 (CH), 129.8 (2 × CH), 129.22 (2 × CH), 129.18 (C), 128.7 (CH), 128.2 (2 × CH), 127.2 (CH), 126.9 (C), 124.51 (CH), 124.48 (CH), 121.9 (C), 114.2 (2 × CH), 101.8 (C), 74.3 (C), 55.4 (CH_3); LRMS (EI) *m/z* (%) 390 (M^+, 15), 359 (100), 100 (80); HRMS (ESI) could not be recorded.

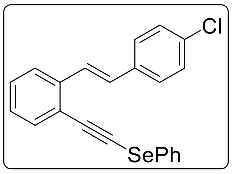

(*E*)-((2-(4-Chlorostyryl)phenyl)ethynyl)(phenyl)selane (*E*-5d): orange solid; mp = 94–96 ºC; R_f= 0.20 (hex/EtOAc, 100/1); 81% yield (638 mg); isolated as a > 20:1 mixture of *E*:*Z* isomers; ^{1}H NMR (300 MHz, $CDCl_3$) δ (ppm) 7.70–7.52 (m, 5H), 7.37–7.26 (m, 9H), 7.12 (d, *J* = 16.3 Hz, 1H); ^{13}C NMR (75.4 MHz, $CDCl_3$) δ (ppm) 138.5 (C), 135.8 (C), 133.5 (C), 132.6 (CH), 129.8 (2 × CH), 129.4 (2 × CH), 129.3 (CH), 129.0 (C), 128.9 (2 × CH), 128.7 (CH), 128.0 (2 × CH), 127.6 (CH), 127.4 (CH), 127.2 (CH), 124.8 (CH), 122.3 (C), 101.5 (C), 74.9 (C); LRMS (EI) *m/z* (%) 396 (M^++2, 2), 394 (M^+, 4), 279 (24), 202 (100); HRMS (ESI) could not be recorded.

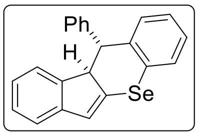

(10*bR,11*R**)-11-Phenyl-10*b*,11-dihydroindeno[2,1-*b*]selenochromene (6a):** yellow solid; mp = 170–172 ºC; R_f= 0.17 (hex/EtOAc, 100/1); 86% yield (93 mg mg); obtained and isolated with d.r. > 20/1 starting from (*E*)-**5a**; ^{1}H NMR (300 MHz, $CDCl_3$) δ(ppm) 7.50–7.15 (m, 9H), 7.06–7.03 (m, 1H), 6.89 (s, 1H), 6.81 (d, *J* = 7.9 Hz, 1H), 6.74 (td, *J* = 7.4, 1.1 Hz, 1H), 5.79 (d, *J* = 7.4 Hz, 1H), 3.86 (d, *J* = 12.7 Hz, 1H), 3.77 (d, *J* = 12.7 Hz, 1H); ^{13}C NMR (75.4 MHz, $CDCl_3$) δ(ppm) 144.8 (C), 144.5 (C), 143.0 (C), 138.3 (C), 136.7 (C), 131.4 (CH), 130.6 (2 × CH), 129.0 (2 × CH), 128.6 (CH), 127.7 (CH), 127.5 (CH), 127.43 (CH), 127.39 (C), 127.2 (CH), 125.9 (CH), 124.8 (CH), 123.3 (CH), 119.4 (CH), 53.5 (CH), 51.5 (CH); LRMS (EI) *m/z* 360 (M^+, 20), 282 (100); HRMS (ESI) could not be recorded.

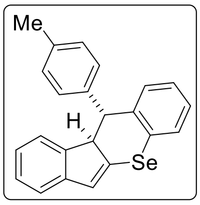

(10*bR,11*R**)11-(*p*-Tolyl)-10b,11-dihydroindeno[2,1-*b*]selenochromene (6b):** yellow solid; mp = 202–204 ºC; R_f= 0.19 (hex/EtOAc, 100/1); 79% yield (89 mg); obtained and isolated with d.r. > 20/1 starting from (*E*)-**5b**; ^{1}H NMR (300 MHz, $CDCl_3$) δ(ppm) 7.35 (dd, *J* = 7.7, 1.3 Hz, 1H), 7.28–7.12 (m, 7H), 7.00 (td, *J* = 7.7, 1.3 Hz, 1H), 6.86 (s, 1H), 6.81 (d, *J* = 7.7 Hz, 1H), 6.75 (td, *J* = 7.7, 1.3 Hz, 1H), 5.86 (d, *J* = 7.7 Hz, 1H), 3.81 (d, *J* = 12.2 Hz, 1H), 3.71 (d, *J* = 12.7 Hz, 1H), 2.48 (s, 3H); ^{13}C NMR (75.4 MHz, $CDCl_3$) δ(ppm) 144.72 (C), 144.67 (C), 139.8 (C), 138.6 (C), 137.2 (C), 136.8 (C), 131.4 (CH), 130.4 (CH), 129.6 (2 × CH), 128.5 (CH), 127.4 (CH), 127.3 (CH), 127.1 (CH), 125.8 (CH), 124.9 (CH), 123.3 (CH), 119.3 (CH), 53.4 (CH), 51.0 (CH), 21.4 (CH_3), one aromatic C and one aromatic CH do not appear due to overlapping; LRMS (EI) *m/z* 374 (M^+, 75), 283 (100), 202 (68); HRMS (APCI) calcd for $C_{23}H_{19}Se$ $[M+H]^+$ 375.0648, found 375.0650.

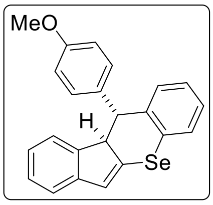

(10*bR,11*R**)-11-(4-Methoxyphenyl)-10*b*,11-dihydroindeno[2,1-*b*]selenochromene (6c):** yellow solid; mp = 175–177 ºC; R_f = 0.11 (hexane); 88% yield (103 mg); obtained and isolated with d.r. = 20/1 starting from (*E*)-**6c**; ^{1}H NMR (300 MHz, $CDCl_3$) δ (ppm) 7.37 (d, *J* = 7.7, 1.3 Hz, 1H), 7.25–7.12 (m, 5H), 7.06–7.01 (m, 3H), 6.88–6.76 (m, 3H), 5.92 (d, *J* = 7.7 Hz, 1H), 3.94 (s, 3H), 3.81 (d, *J* = 12.6 Hz, 1H), 3.72 (d, *J* = 12.6 Hz, 1H); ^{13}C NMR (75.4 MHz, $CDCl_3$) δ (ppm) 159.0 (C), 144.7 (C), 144.6 (C), 138.7 (C), 136.8 (C), 134.9 (CH), 131.4 (C), 131.3 (CH), 128.5 (2 × CH), 127.41 (CH), 127.36 (CH), 127.1 (CH), 125.8 (CH), 124.9 (CH), 123.3 (CH), 119.3 (CH), 114.3 (2 × CH), 55.5 (CH_3), 53.5 (CH), 50.5 (CH), one aromatic C does not appear due to overlapping; LRMS (EI) *m/z* 390 (M^+, 85), 283 (100), 202 (72); HRMS (ESI) could not be recorded.

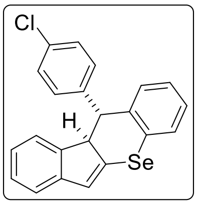

(10*bR,11*R**)-11-(4-Chlorophenyl)-10*b*,11-dihydroindeno[2,1-*b*]selenochromene (6d):** orange solid; mp = 198–200 ºC; R_f = 0.15 (hexane); 70% yield (83 mg); obtained and isolated with d.r. > 20/1 starting from (*E*)-**5d**; ^{1}H NMR (300 MHz, $CDCl_3$) δ (ppm) 7.49 (d, *J* = 8.4 Hz, 2H), 7.40 (dd, *J* = 7.7, 1.4 Hz, 1H), 7.30–7.17 (m, 5H), 7.05 (td, *J* = 7.6, 1.4 Hz, 1H), 6.91 (s, 1H), 6.82 (td, *J* = 7.7, 1.4 Hz, 1H), 6.76 (d, *J* = 8.0 Hz, 1H), 5.91 (d, *J* = 7.6 Hz, 1H), 3.81 (d, *J* =13.1 Hz, 1H), 3.76 (d, *J* =13.1 Hz, 1H); ^{13}C NMR (75.4 MHz, $CDCl_3$) δ (ppm) 144.7 (C), 144.2 (C), 141.6 (2 × C), 137.7 (C), 136.4 (C), 133.4 (C), 131.9 (2 × CH), 131.2 (CH), 129.2 (2 × CH), 128.7 (CH), 127.7 (CH), 127.6 (CH), 127.4 (CH), 125.9 (CH), 124.6 (CH), 123.5 (CH), 119.5 (CH), 53.2 (CH), 50.9 (CH); LRMS (EI) *m/z* 396 (M^++2, 25), 394 (M^+, 68), 283 (100), 202 (60); HRMS (ESI) could not be recorded.

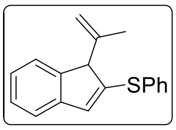

Phenyl-(1-(prop-1-en-2-yl)-1*H*-inden-2-yl)sulfane (7u): the spectroscopic data matches those reported in our previous work.[2] Dark yellow oil, R_f = 0.25 (hex/EtOAc, 100/1), 71% yield (56 mg), obtained and isolated as a single product. ^{1}H NMR (300 MHz, $CDCl_3$) δ (ppm) 7.59–7.56 (m, 2H), 7.41–7.38 (m, 3H), 7.28–7.13 (m, 4H), 6.46 (dd, *J* = 1.5, 0.6 Hz, 1H), 5.09 (d, *J* = 5.6, 1H), 5.08 (d, *J* = 5.6, 1H), 4.19 (s, 1H), 1.34 (s, 3H). ^{13}C NMR (75.4 MHz, $CDCl_3$) δ (ppm) 146.7 (C), 145.7 (C), 144.1 (C), 143.0 (C), 133.5 (2 × CH), 133.1 (C), 129.4 (2 × CH), 128.8 (CH), 128.3 (CH), 127.2 (CH), 124.5 (CH), 123.2 (CH), 119.6 (CH), 116.2 (CH_2), 60.9 (CH), 16.8 (CH_3). LRMS (EI) *m/z* 264 (M^+, 43), 155 (100). HRMS (ESI) calcd for $C_{18}H_{17}S$ $[M+H]^+$ 265.1045, found 265.1041.

Enantioselective Synthesis of Selected Dihydroindeno[2,1-*b*]thiochromenes 4

Optimization of the reaction conditions for the enantioselective synthesis of 4a:

Table S.1. Optimization of the reaction conditions for the enantioselective synthesis of **4a**[a]

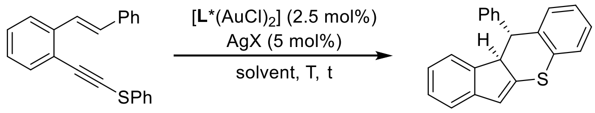

(E)-3a **4a**

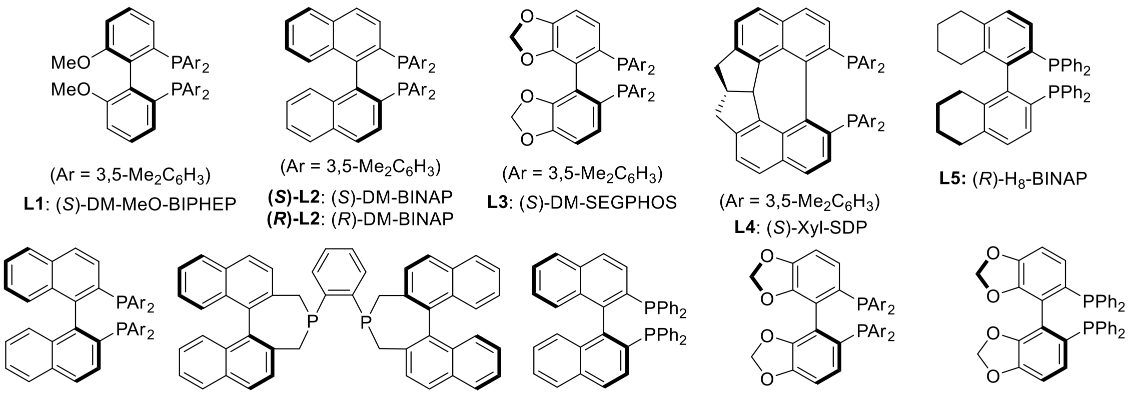

(Ar = 3,5-Me$_2$C$_6$H$_3$) **L1**: (*S*)-DM-MeO-BIPHEP

(Ar = 3,5-Me$_2$C$_6$H$_3$) **(*S*)-L2**: (*S*)-DM-BINAP **(*R*)-L2**: (*R*)-DM-BINAP

(Ar = 3,5-Me$_2$C$_6$H$_3$) **L3**: (*S*)-DM-SEGPHOS

(Ar = 3,5-Me$_2$C$_6$H$_3$) **L4**: (*S*)-Xyl-SDP

L5: (*R*)-H$_8$-BINAP

(Ar = 4-MeC$_6$H$_4$) **L6**: (*R*)-Tol-BINAP

L7: (*R*)-binaphane

L8: (*R*)-BINAP

(Ar = 3,5-(*t*Bu)$_2$-4-MeOC$_6$H$_2$) **L9**: (*S*)-DTMB-MeO-SEGPHOS

L10: (*S*)-SEGPHOS

Entry	L*/AgX	solvent	T (°C)	t (h)	*e.r.*[b]	yield (%)
1	**L1**/AgOTs	DCM	25	0.5	67:33	82
2	**(*S*)-L2**/AgOTs	DCM	25	0.5	85:15	87
3	**L3**/AgOTs	DCM	25	0.5	73:27	90
4	**L4**/AgOTs	DCM	25	0.5	46:53	90
5	**L5**/AgOTs	DCM	25	0.5	52:47	83
6	**L6**/AgOTs	DCM	25	0.5	47:52	87
7	**L7**/AgOTs	DCM	25	0.5	53:46	69
8	**L8**/AgOTs	DCM	25	0.5	49:51	70
9	**(*S*)-L2**/AgSbF$_6$	DCM	25	0.5	55:45	72
10	**(*S*)-L2**/NaBArF	DCM	25	0.5	57:42	90
11	**(*S*)-L2**/AgNTf$_2$	DCM	25	0.5	54:45	89
12	**(*S*)-L2**/AgOTs	DCM	0	0.5	85:15	90
13	**(*S*)-L2**/AgOTs	DCM	–20	72	85:15	74[c]
14	**(*S*)-L2**/AgOTs[d]	DCE	–10	16	85:15	90
15	**L3**/AgOTs	DCE	–10	16	90:10	91
16	**L9**/AgOTs	DCE	–10	16	72:28	81
17	**L10**/AgOTs	DCE	–10	16	33:66	80

[a]Reactions conditions: *o*-(alkynyl)styrene (*E*)-**3a** (0.1 mmol), L*(AuCl)$_2$ (2.5 mol%), silver salt (5 mol%) in 0.4 mL of solvent until complete conversion. [b]Determined by HPLC on a chiral stationary phase using a Chiracel ADH column (eluent: *n*-hexane/**i**-PrOH (90:10), flow: 0.5 mL/min). [c]80% conversion as estimated by ^{1}H NMR. [d]*e.r.* = 15:85 when (*R*)-**L2** is employed.

Starting from *o*-alkynylstyrene (*E*)-**3a** we carried out the gold-catalyzed reaction in the presence of chiral biphosphines with biphenyl skeletons as ligands in order to study the enantioselectivity of the process. Firstly, we selected a variety of dinuclear chiral gold(I) catalysts with (*S*)-DM-MeO-Biphep (**L1**), (*S*)-DM-Binap ((*S*)-**L2**), (*R*)-DM-Binap ((*R*)-**L2**), (*S*)-DM-Segphos (**L3**), (*S*)-Xylyl-SDP (**L4**), (*R*)-H_8-Binap (**L5**), (*R*)-Tol-Binap (**L6**), (*R*)-Binaphane (**L7**), (*R*)-Binap (**L8**), (*S*)-DTBM-MeO-Segphos (**L9**) and (*S*)-Segphos (**L10**) as ligands, previously prepared according to known procedures.[10] Some tests were carried out employing different gold(I) complexes with chiral ligands (**L1**–**L8**) along with AgOTs, in dichloromethane as solvent at rt during 30 min obtaining, in all cases, product **4a** with high yields (entries 1–8). The best enantiomeric ratios were obtained using **L2** (*e.r.* = 85:15) and **L3** (*e.r.* = 73:27). Then, different silver salts were evaluated using the gold complex with **L2** as chiral ligand (entries 9–11). However, the enantiomeric ratios did not improve. At this point, we decided to decrease the reaction temperature. We found that the *e.r.* did not improve when carrying out the reaction at 0 ºC (entry 12), whereas when cooling to −20 ºC the conversion was not complete, even increasing the reaction time up to 72 h (entry 13). When the reaction was performed in DCE at −10 ºC for 16 h the conversion was complete but the *e.r.* did not improve (entry 14). Surprisingly, when we changed the chiral ligand from **L2** to **L3** and carried out the reaction in DCE at −10 ºC (entry 15), we found the best *e.r.* using this (*S*)-DM-Segphos (**L3**) as chiral ligand (*e.r.* = 90:10). No further improvement was found using other chiral ligands such as **L9** or **L10** under these conditions (entries 16 and 17).

Enantioselective synthesis of selected thiochromenes 4:

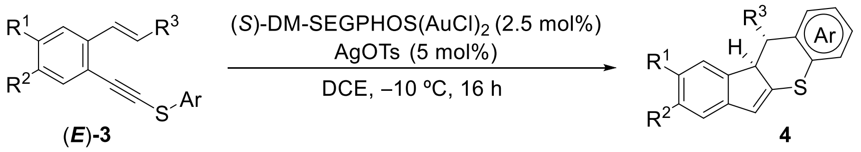

(***E***)**-3** → **4**

AgOTs (3 mg, 0.015 mmol) was added to a solution of (*S*)-DM-SEGPHOS(AuCl)$_2$ (7 mg, 0.008 mmol) in dry DCE (1.2 mL) and the resulting mixture was stirred for 10–15 min under N_2. Then, the mixture was cooled to −10 ºC and the corresponding starting *o*-(alkynyl)styrene (***E***)**-3** (0.3 mmol) was added. The resulting reaction mixture was stirred at −10 ºC until total consumption of the styrene derivative (16 h). The mixture was filtered through a short pad of silica gel using a 100:1 mixture of hexane/EtOAc as eluent. The solvent was removed under reduced pressure, and the crude mixture was purified by flash column chromatography on silica gel using mixtures of hexane/EtOAc as eluents to obtain the corresponding dihydroindeno[2,1-*b*]thiochromene **4** in the yields and enantiomeric ratios reported in Table 4.

Chiral HPLC-traces:

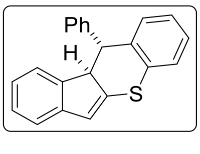

4a (89%, er = 90/10)
HPLC (Chiralpak AD-H, *i*-propanol/*n*-hexane 2/98, flow rate = 0.5 mL/min, l = 230 nm) tR = 9.8 min (major), 11.7 min (minor)

[10] Muñoz, M. P.; Adrio, J.; Carretero, J. C.; Echavarren, A. M. *Organometallics* **2005**, *24*, 1293–1300.

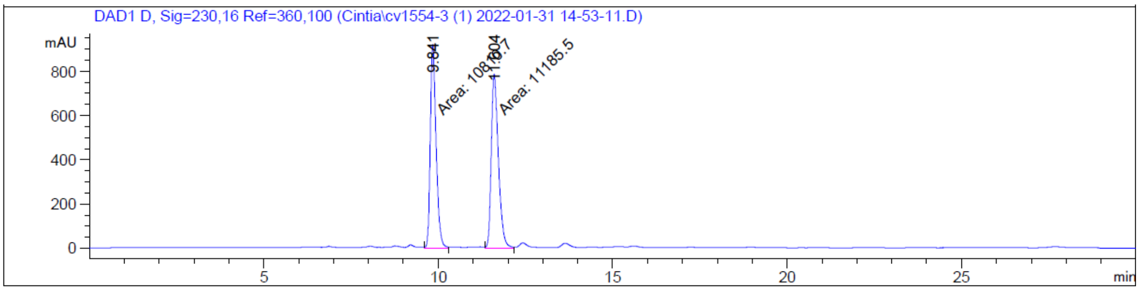

Peak #	RetTime [min]	Type	Width [min]	Area [mAU*s]	Height [mAU]	Area %
1	9.841	MM	0.1952	1.08107e4	922.81201	49.1480
2	11.604	MM	0.2364	1.11855e4	788.69110	50.8520

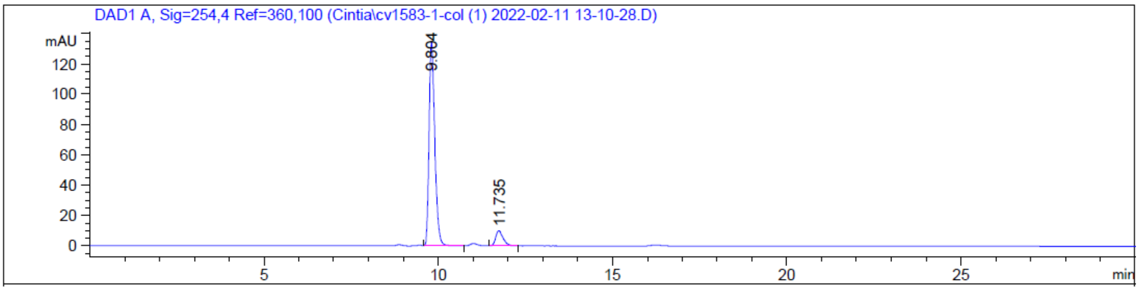

Peak #	RetTime [min]	Type	Width [min]	Area [mAU*s]	Height [mAU]	Area %
1	9.804	VB	0.1945	1.80206e4	1434.84473	90.6337
2	11.732	MM T	0.2350	1862.28259	132.09766	9.3663

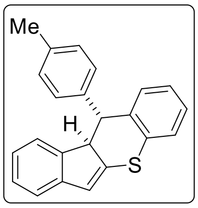

4b (84%, er = 86/14)

HPLC (Chiralpak AD-H, *i*-propanol/*n*-hexane 2/98, flow rate = 0.5 mL/min, l = 230 nm) tR = 9.2 min (major), 10.4 min (minor)

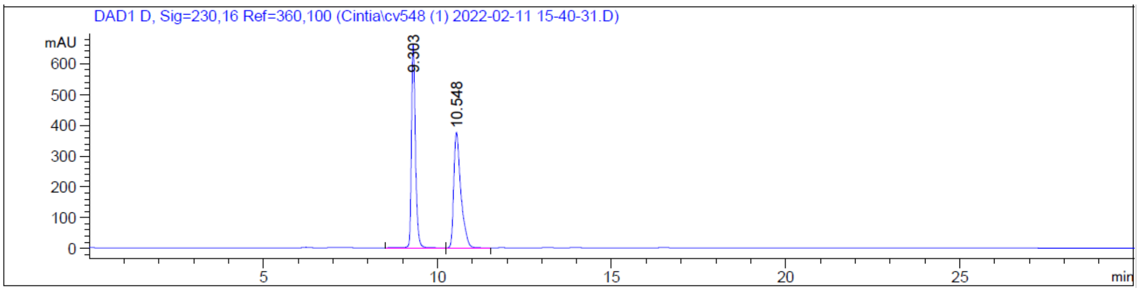

Peak #	RetTime [min]	Type	Width [min]	Area [mAU*s]	Height [mAU]	Area %
1	9.303	BB	0.1189	5274.70068	662.14709	49.8421
2	10.548	BB	0.2072	5308.11865	374.93533	50.1579

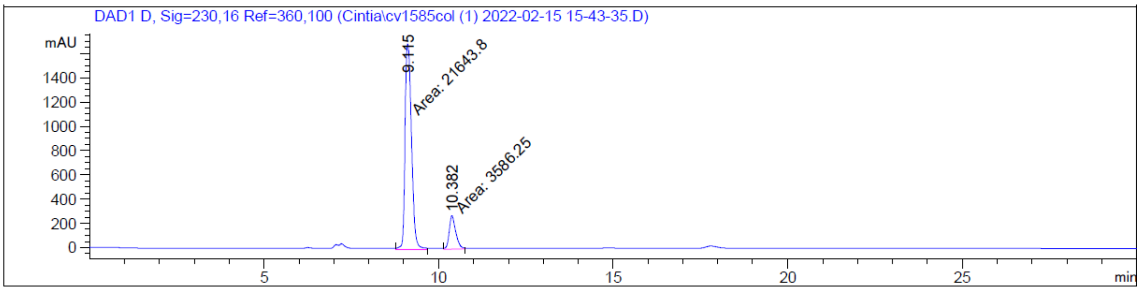

Peak #	RetTime [min]	Type	Width [min]	Area [mAU*s]	Height [mAU]	Area %
1	9.115	MM	0.2132	2.16438e4	1691.73035	85.7858
2	10.382	MM	0.2182	3586.24609	273.94598	14.2142

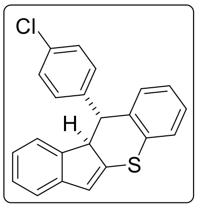

4d (85%, er = 78/22)

HPLC (Chiralpak AD-H, *i*-propanol/*n*-hexane 2/98, flow rate = 0.5 mL/min, l = 230 nm) tR = 10.8 min (major), 12.0 min (minor)

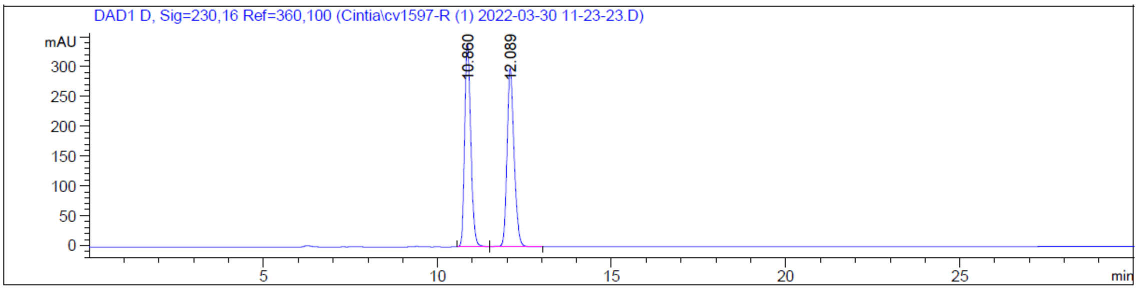

Peak #	RetTime [min]	Type	Width [min]	Area [mAU*s]	Height [mAU]	Area %
1	10.860	BB	0.1865	4163.16846	340.78809	49.5984
2	12.089	BB	0.2151	4230.59229	298.99417	50.4016

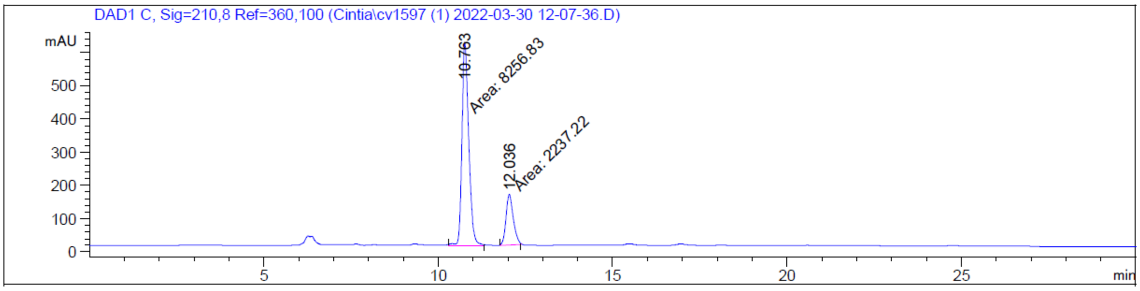

Peak #	RetTime [min]	Type	Width [min]	Area [mAU*s]	Height [mAU]	Area %
1	10.763	MM	0.2251	8256.82910	611.44525	78.6810
2	12.036	MM	0.2427	2237.22241	153.63199	21.3190

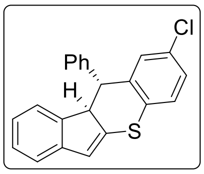

4j (87%, er = 88/12)
HPLC (Chiralpak AD-H, *i*-propanol/*n*-hexane 2/98, flow rate = 0.5 mL/min, l = 230 nm) tR = 10.1 min (major), 13.4 min (minor)

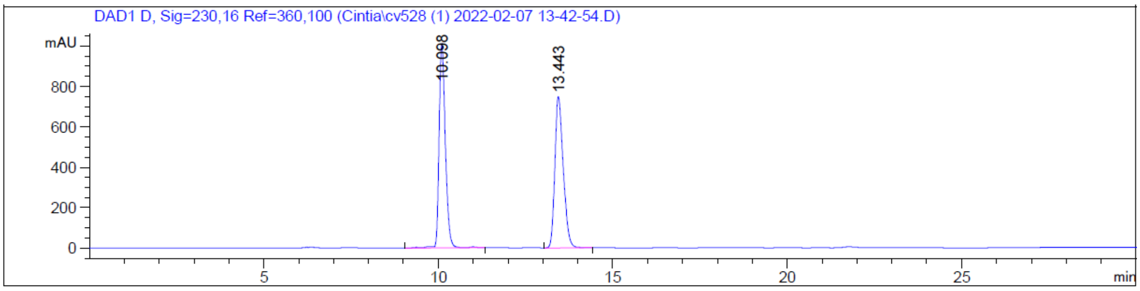

Peak #	RetTime [min]	Type	Width [min]	Area [mAU*s]	Height [mAU]	Area %
1	10.098	VV R	0.1868	1.25441e4	1009.10828	50.0627
2	13.443	BB	0.2529	1.25127e4	749.08551	49.9373

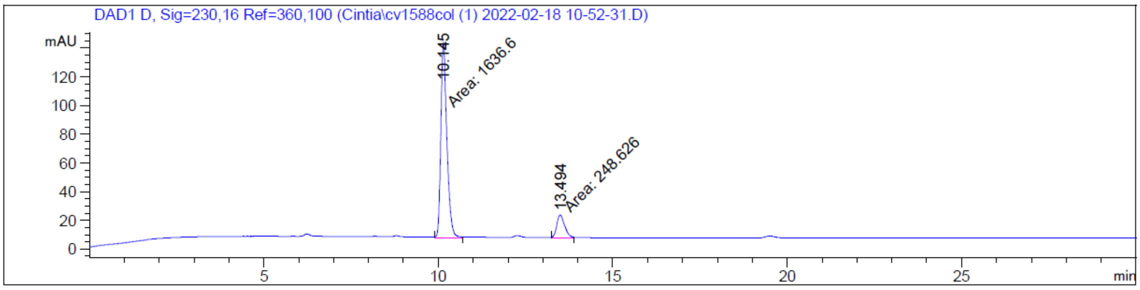

Peak #	RetTime [min]	Type	Width [min]	Area [mAU*s]	Height [mAU]	Area %
1	10.145	MM	0.2012	1636.60229	135.57088	86.8119
2	13.494	MM	0.2617	248.62584	15.83632	13.1881

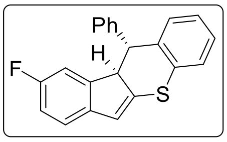

4o (74%, er = 72/28)

HPLC (Chiralpak AD-H, *i*-propanol/*n*-hexane 2/98, flow rate = 0.5 mL/min, l = 230 nm) tR = 10.5 min (major), 11.3 min (minor)

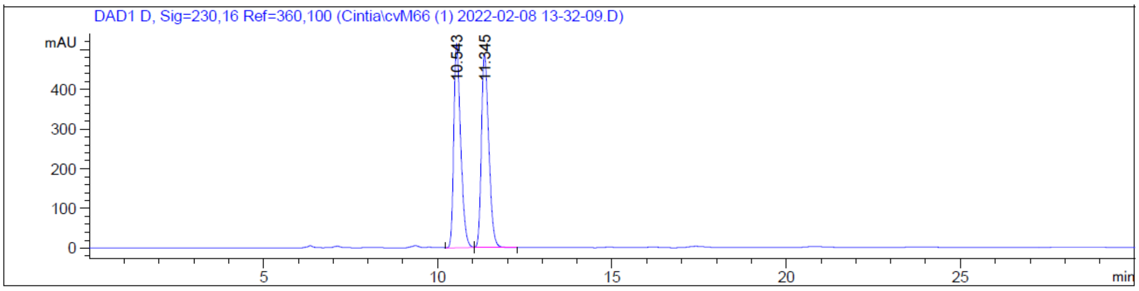

Peak #	RetTime [min]	Type	Width [min]	Area [mAU*s]	Height [mAU]	Area %
1	10.543	BV	0.2095	7107.11768	513.28198	49.7898
2	11.345	VB	0.2261	7167.11719	485.73230	50.2102

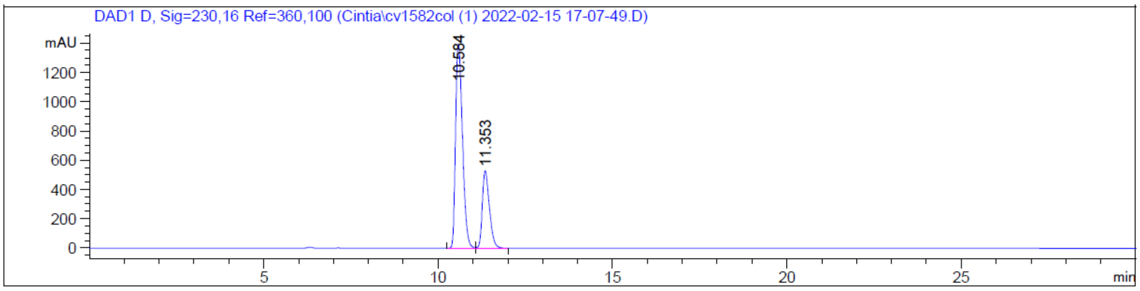

Peak #	RetTime [min]	Type	Width [min]	Area [mAU*s]	Height [mAU]	Area %
1	10.584	BV	0.2099	1.91639e4	1397.87830	72.0638
2	11.353	VB	0.2077	7429.04541	529.29633	27.9362

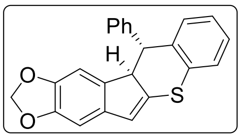

4p (88%, er = 84/16)
HPLC (Chiralpak AD-H, *i*-propanol/*n*-hexane 2/98, flow rate = 0.5 mL/min, l = 230 nm) tR = 19.9 min (major), 24.0 min (minor)

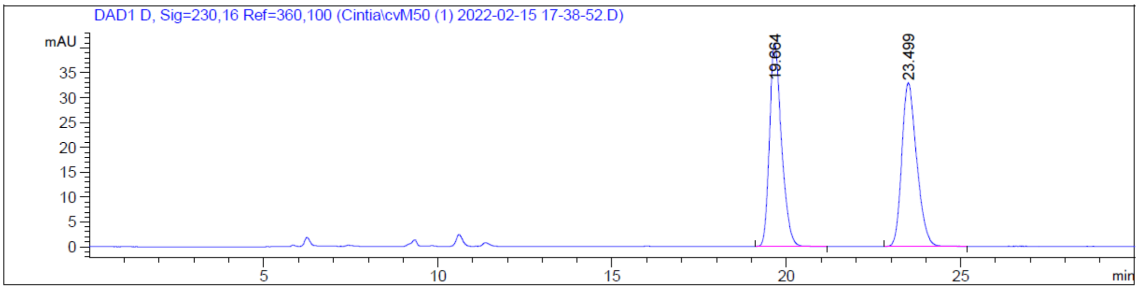

Peak #	RetTime [min]	Type	Width [min]	Area [mAU*s]	Height [mAU]	Area %
1	19.664	BB	0.3574	979.57288	40.90039	50.0243
2	23.499	BB	0.4513	978.62244	32.87201	49.9757

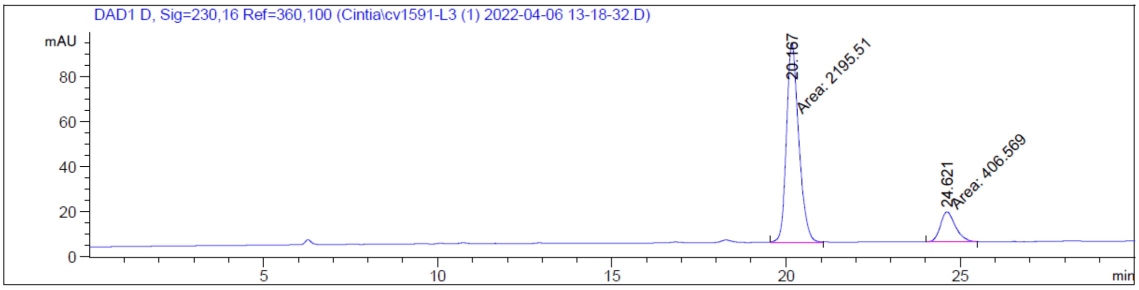

Peak #	RetTime [min]	Type	Width [min]	Area [mAU*s]	Height [mAU]	Area %
1	20.167	MM	0.4105	2195.51489	89.14542	84.3753
2	24.621	MM	0.5054	406.56860	13.40846	15.6247

X-Ray Crystallographic Data for 4m

A single crystal of CCDC2168644 (**4m**) suitable for X-Ray crystallography was obtained by crystallization *via* evaporation from its hexane solution. The crystal was kept at 173.15 K during data collection on a Bruker APEX-II CCD diffractometer. The structure was solved with the ShelXT[11] structure solution program using Intrinsic Phasing and refined with the ShelXL[12] refinement package using Least Squares minimization.

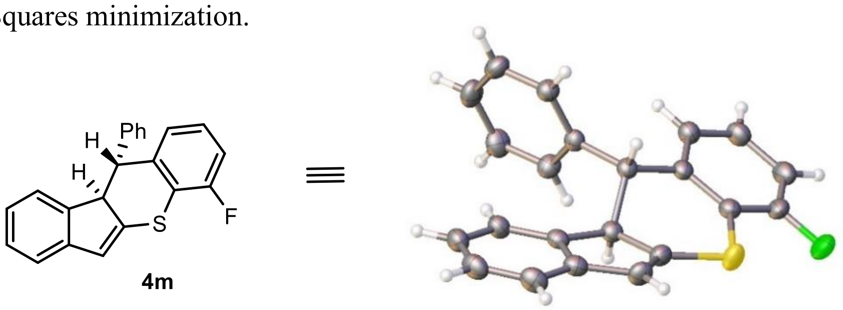

ORTEP drawing of **4m** showing thermal ellipsoids at the 50% probability level.

Table S1: Crystal data and structure refinement for 4m (CCDC2168644)

**Identification code**	**4m**
**Empirical formula**	$C_{22}H_{15}FS$
**Formula weight**	330.40
**Temperature/K**	173.15
**Crystal system**	Monoclinic
**Space group**	$P2_1/c$
**a/Å**	5.543(5)
**b/Å**	18.490(16)
**c/Å**	15.414(14)
**α/°**	90
**β/°**	94.771(15)
**γ/°**	90
**Volume/Å^3**	1574(2)
**Z**	4
**ρ_{calc}g/cm^3**	1.394
**μ/mm^{-1}**	0.215
**F(000)**	688.0
**Crystal size/mm^3**	0.4 × 0.1 × 0.1
**Radiation**	MoKα (λ = 0.71073)
**2Θ range for data collection/°**	3.448 to 52.044
**Index ranges**	$-6 \leq h \leq 6, -22 \leq k \leq 22$ $-18 \leq l \leq 18$
**Reflections collected**	12544
**Independent reflections**	3091 [R_{int} = 0.0492, R_{sigma} = 0.0403]
**Data/restraints/parameters**	3091/0/217
**Goodness-of-fit on F^2**	1.049
**Final R indexes [I>=2σ (I)]**	R_1 = 0.0429, wR_2 = 0.1128
**Final R indexes [all data]**	R_1 = 0.0502, wR_2 = 0.1176
**Largest diff. peak/hole / e Å^{-3}**	0.33/–0.24

[11] Sheldrick, G. M. *Acta Cryst*. **2015**, *A71*, 3-8.
[12] Sheldrick, G. M. *Acta Cryst*. **2015**, *C71*, 3-8.

X-Ray Crystallographic Data for 4w

A single crystal of CCDC2168642 **(4w)** suitable for X-Ray crystallography was obtained by crystallization *via* evaporation from its hexane solution. The crystal was kept at 240.0 K during data collection on a Bruker APEX-II CCD diffractometer. The structure was solved with the ShelXT[11] structure solution program using Intrinsic Phasing and refined with the ShelXL[12] refinement package using Least Squares minimization.

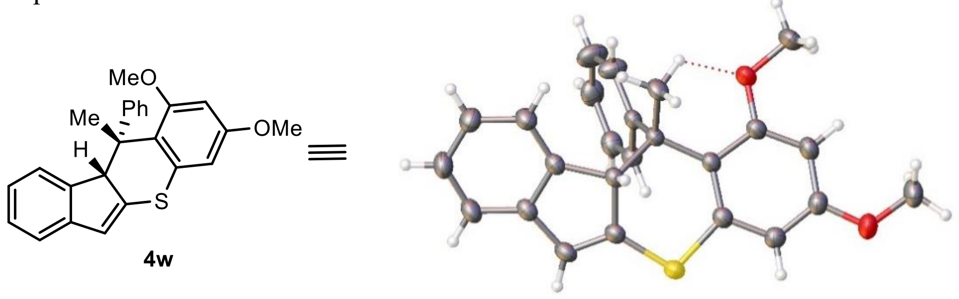

ORTEP drawing of **4w** showing thermal ellipsoids at the 50% probability level.

Table S2: Crystal data and structure refinement for 4w (CCDC2168642)

**Identification code**	**4w**
**Empirical formula**	$C_{25}H_{22}O_2S_1$
**Formula weight**	386.48
**Temperature/K**	240(2)
**Crystal system**	Monoclinic
**Space group**	$P2_1/n$
**a/Å**	8.9677(7)
**b/Å**	13.1732(10)
**c/Å**	16.5100(12)
**α/°**	90
**β/°**	98.051(2)
**γ/°**	90
**Volume/Å^3**	1931.2(3)
**Z**	4
**ρ_{calc}g/cm^3**	1.329
**μ/mm^{-1}**	1.624
**F(000)**	816.0
**Crystal size/mm^3**	0.3 × 0.2 × 0.04
**Radiation**	CuKα (λ = 1.54178)
**2Θ range for data collection/°**	8.62 to 144.64
**Index ranges**	$-11 \le h \le 11, -16 \le k \le 16, -20 \le l \le 20$
**Reflections collected**	35263
**Independent reflections**	3765 [R_{int} = 0.0610, R_{sigma} = 0.0331]
**Data/restraints/parameters**	3765/0/256
**Goodness-of-fit on F^2**	1.029
**Final R indexes [I>=2σ (I)]**	R_1 = 0.0422, wR_2 = 0.1059
**Final R indexes [all data]**	R_1 = 0.0468, wR_2 = 0.1122
**Largest diff. peak/hole / e Å^{-3}**	0.26/–0.30

^{1}H and ^{13}C NMR Spectra of Characterized Compounds

^1H NMR (300 MHz, $CDCl_3$)

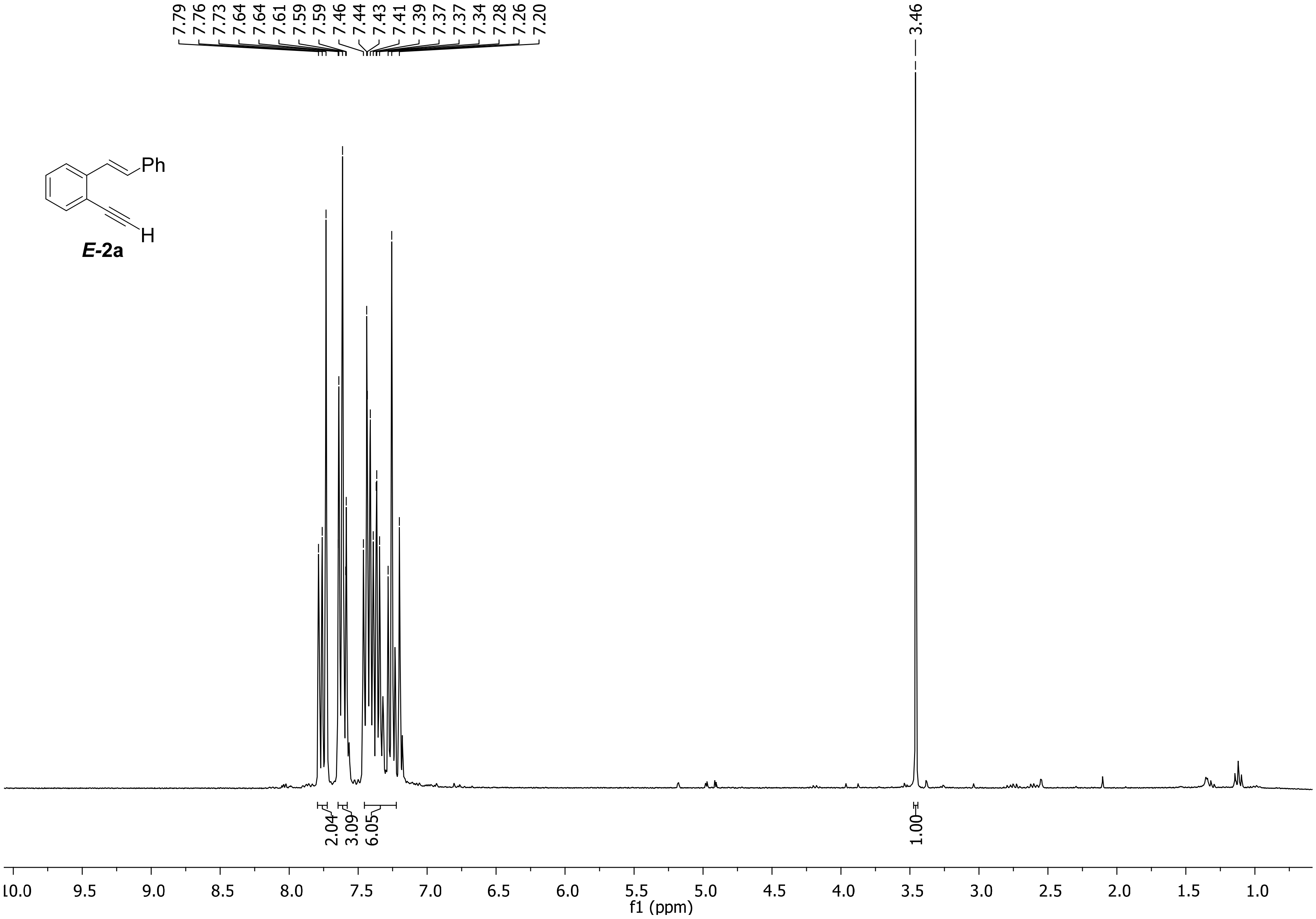

^{13}C NMR (75.4 MHz, $CDCl_3$)

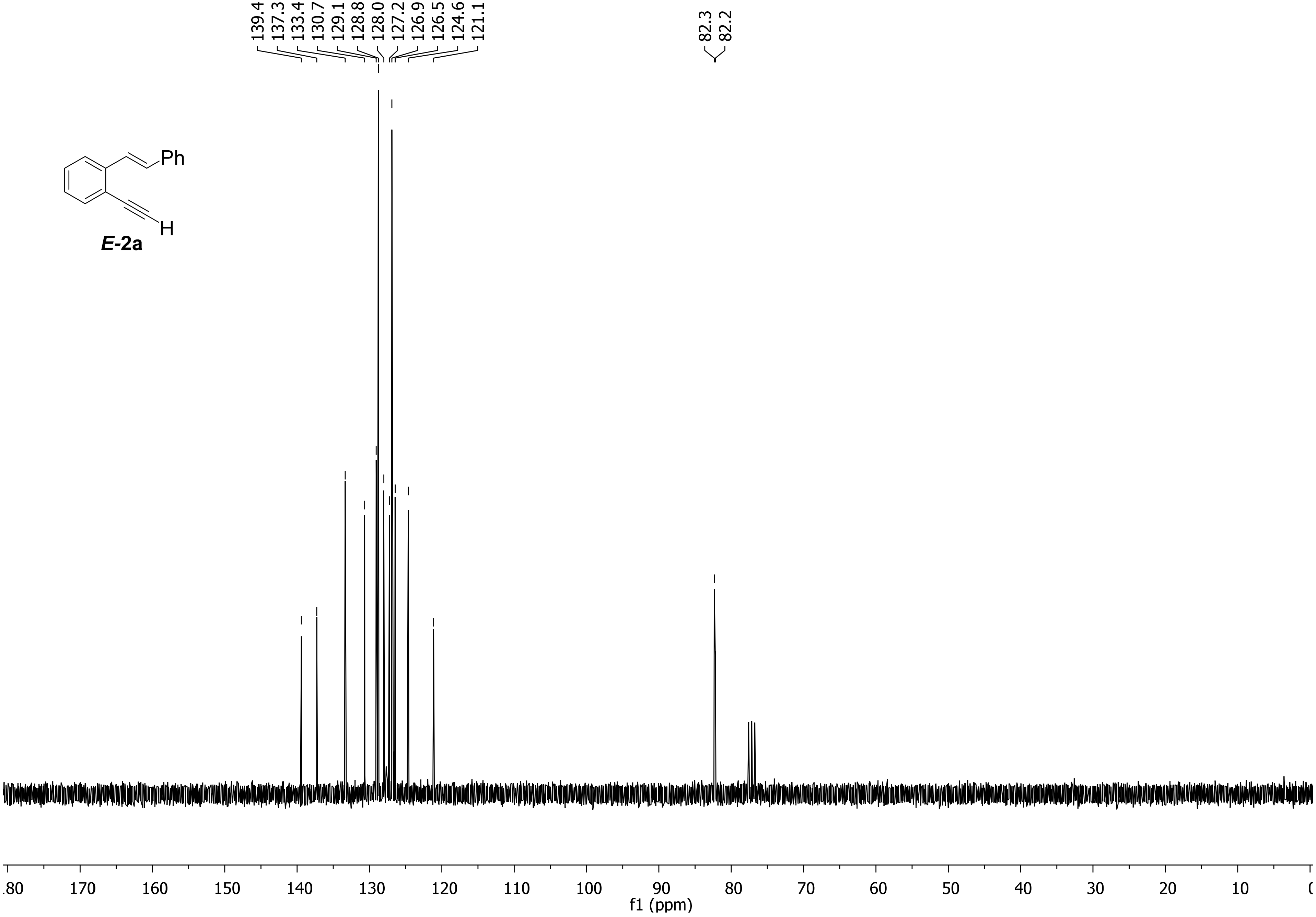

^{1}H NMR (300 MHz, CDCl$_3$)

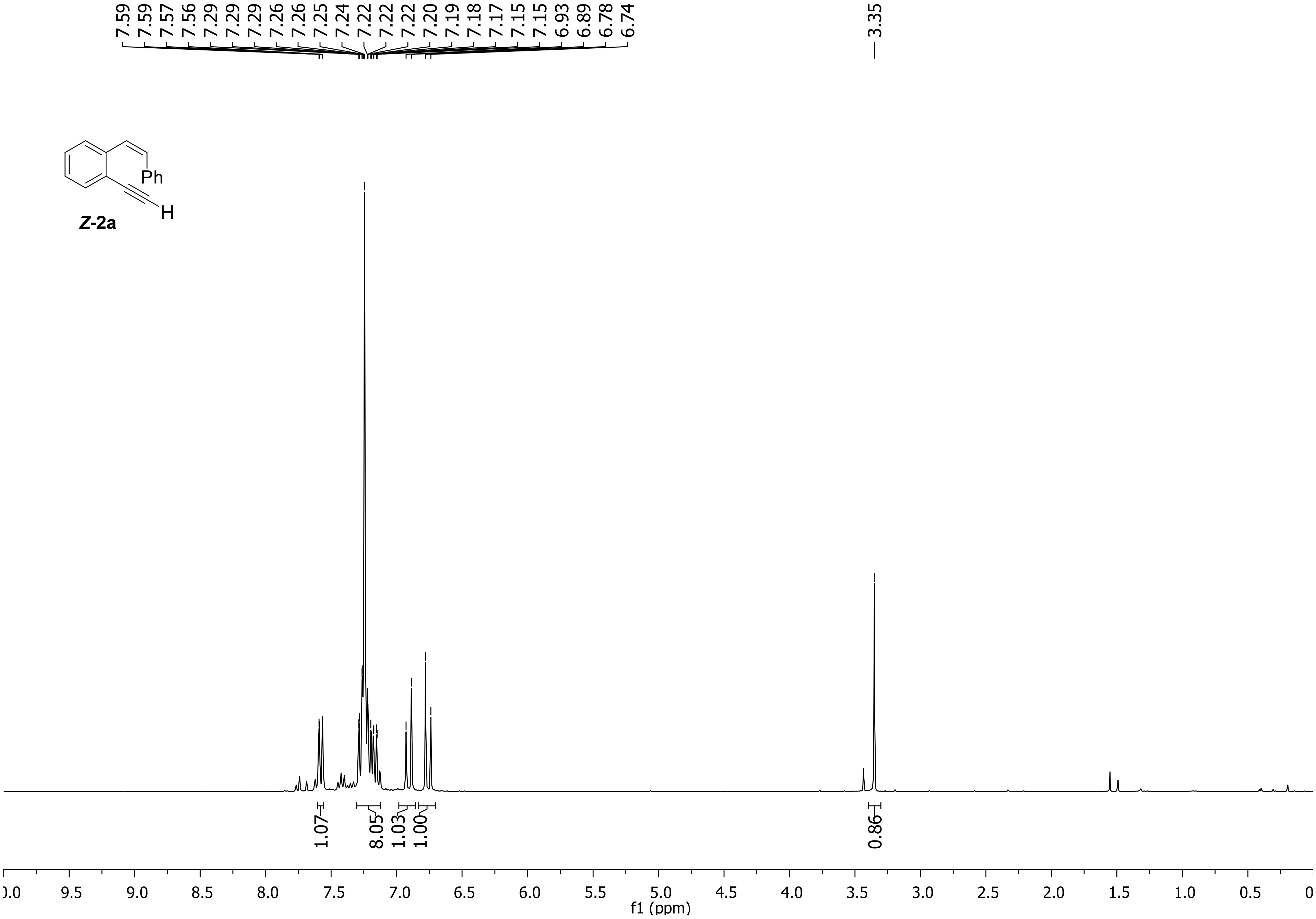

^{13}C NMR (75.4 MHz, $CDCl_3$)

***Z*-2a**

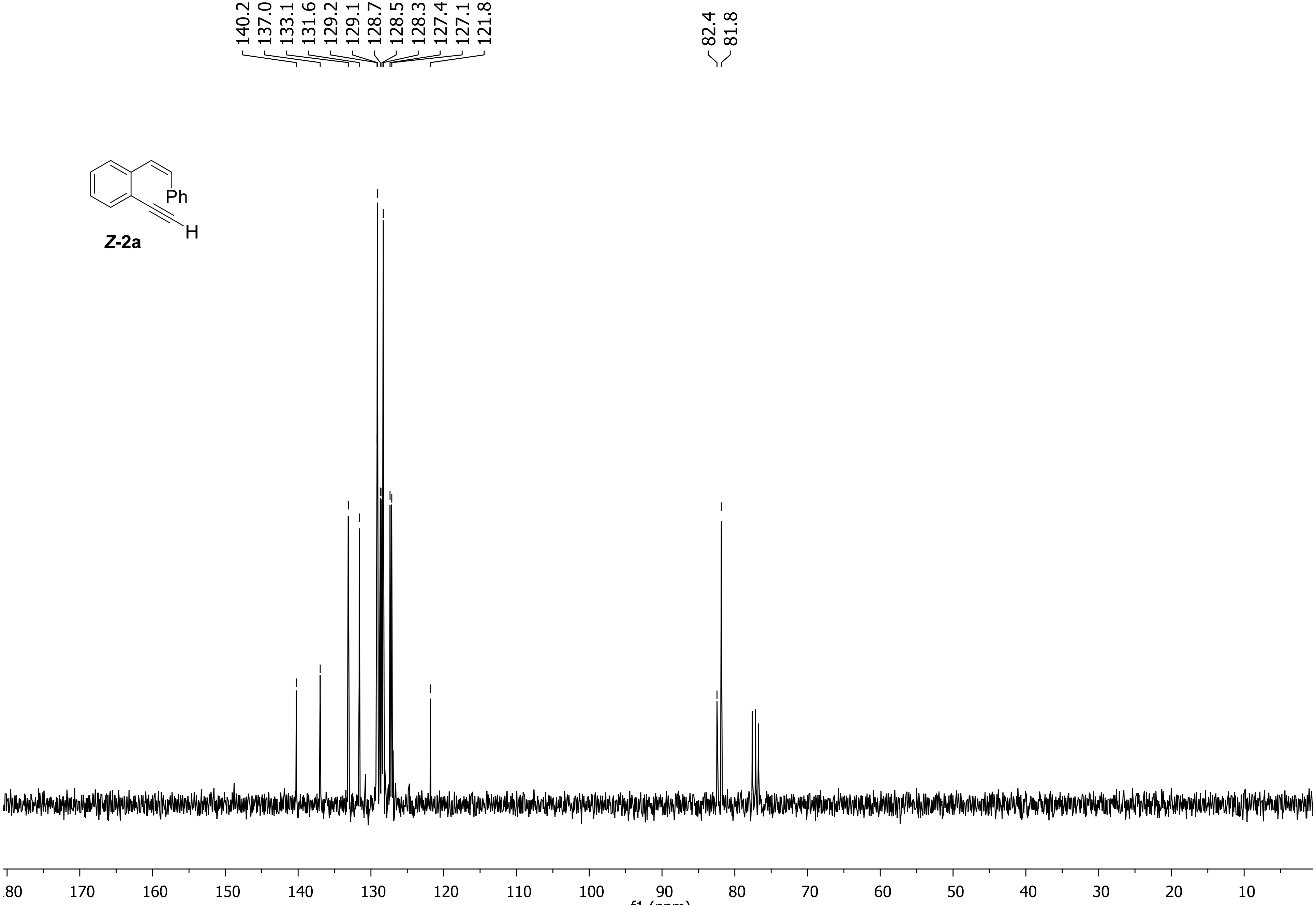

^{1}H NMR (300 MHz, $CDCl_3$)

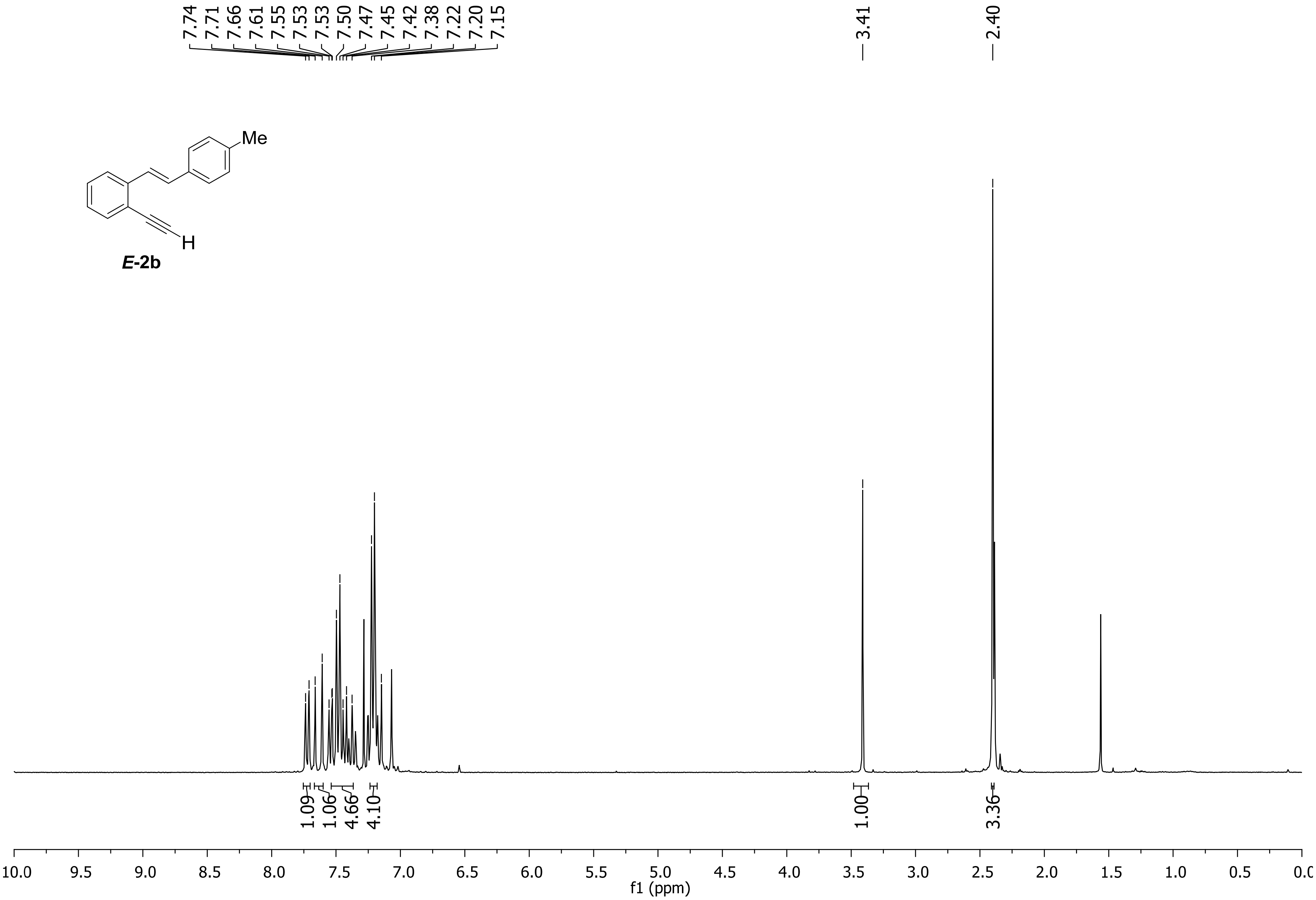

^{13}C NMR (75.4 MHz, $CDCl_3$)

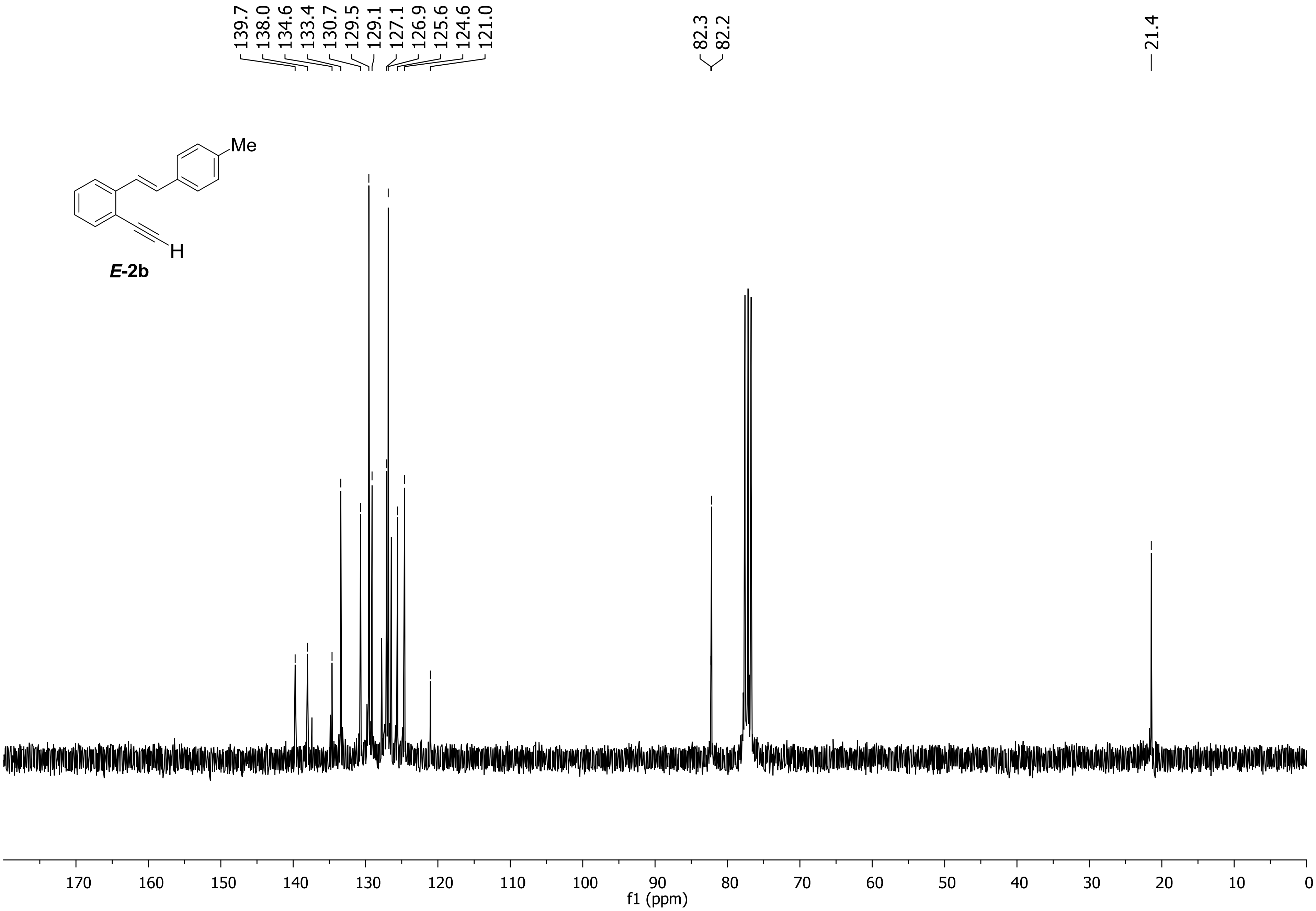

^{1}H NMR (300 MHz, $CDCl_3$)

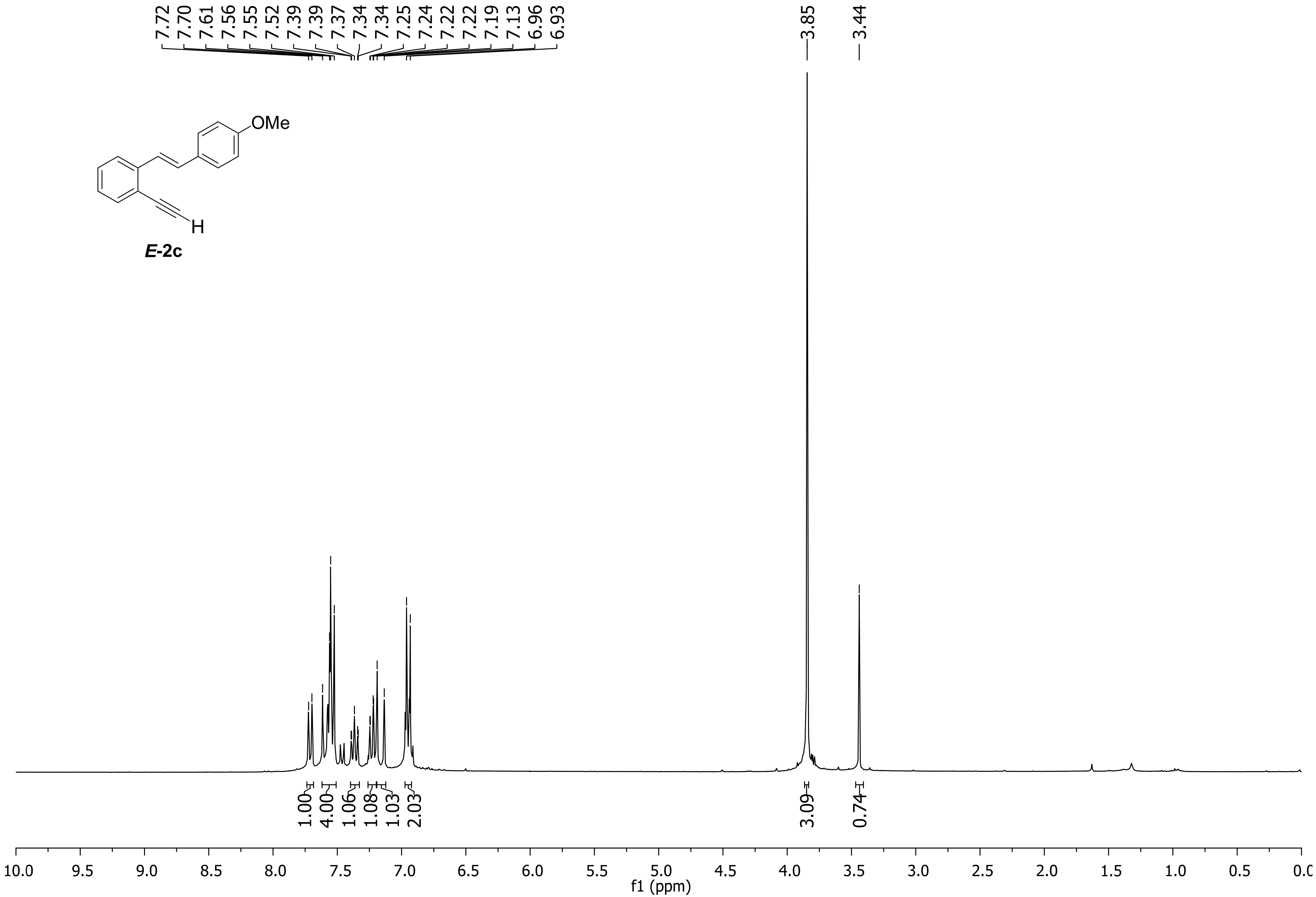

^{13}C NMR (75.4 MHz, $CDCl_3$)

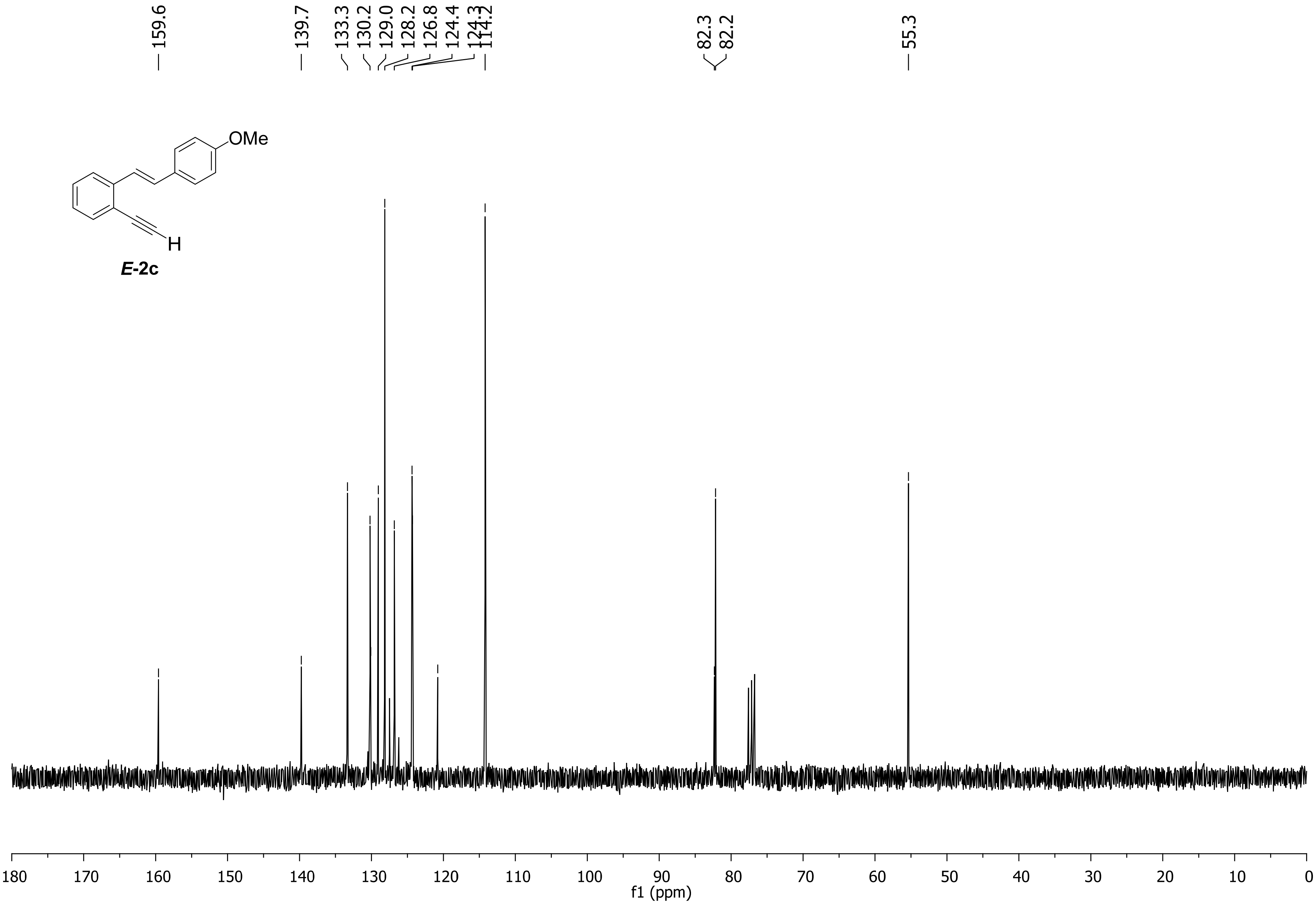

^{1}H NMR (300 MHz, $CDCl_3$)

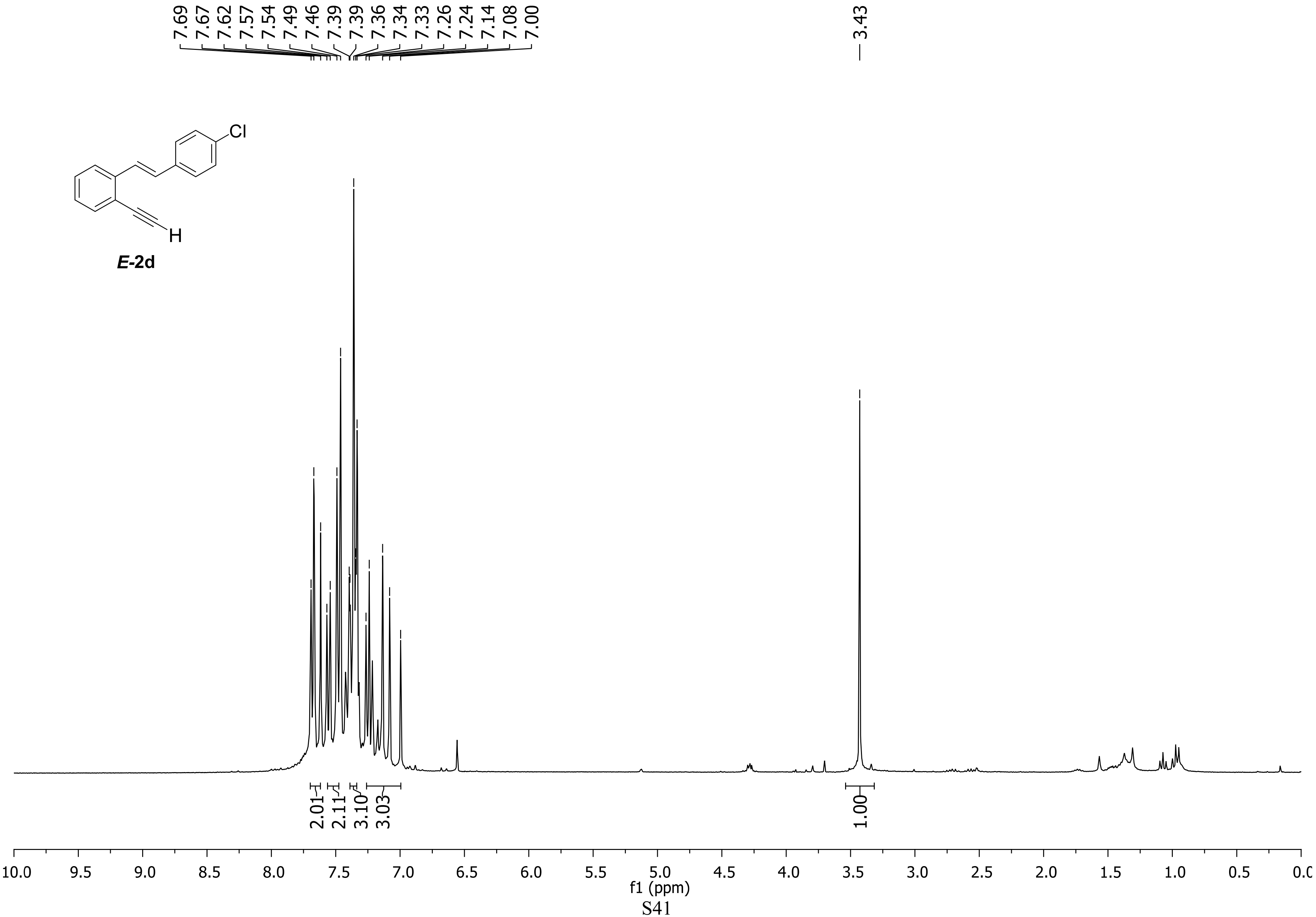

***E*-2d**

^{13}C NMR (75.4 MHz, $CDCl_3$)

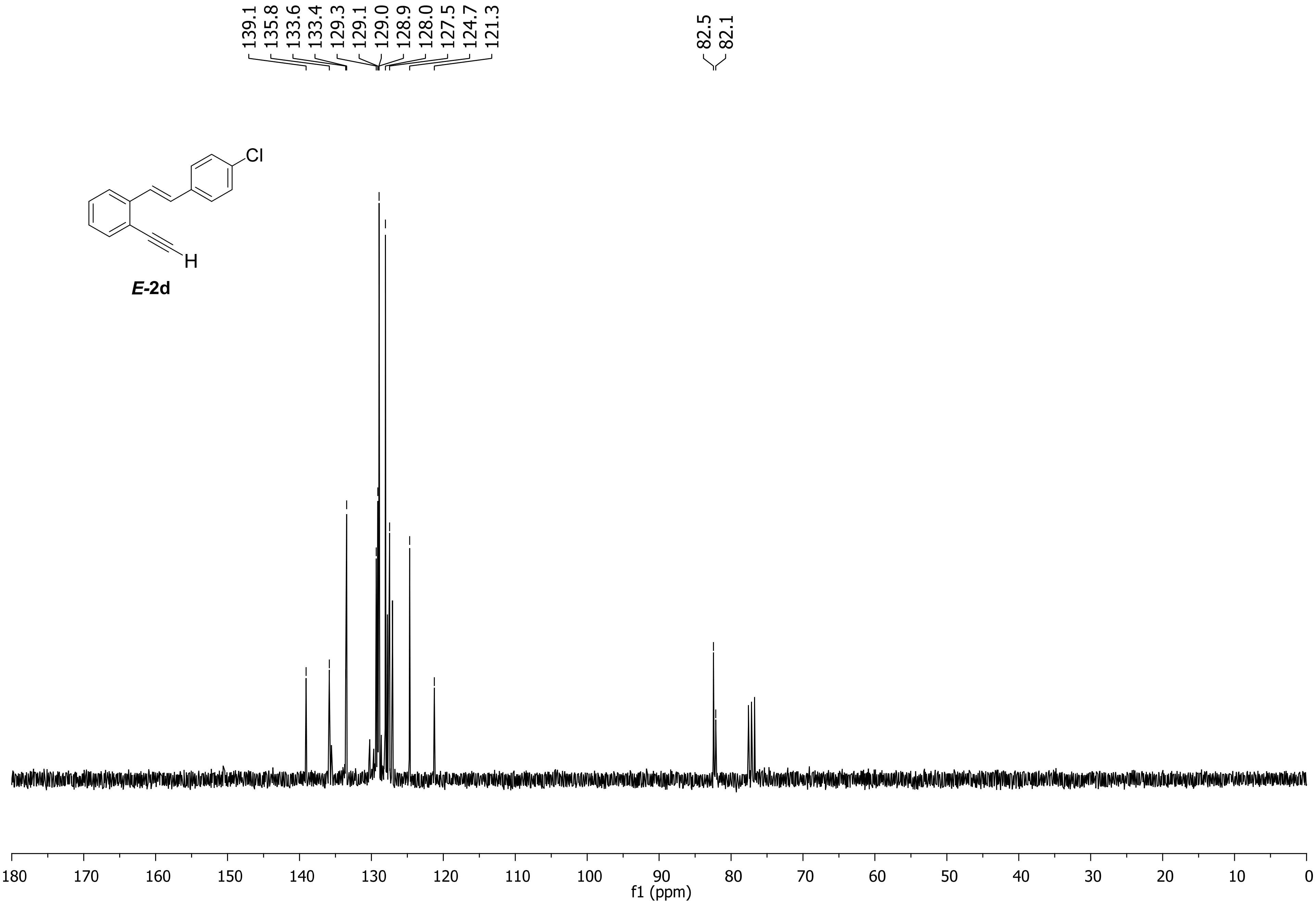

^{1}H NMR (300 MHz, $CDCl_3$)

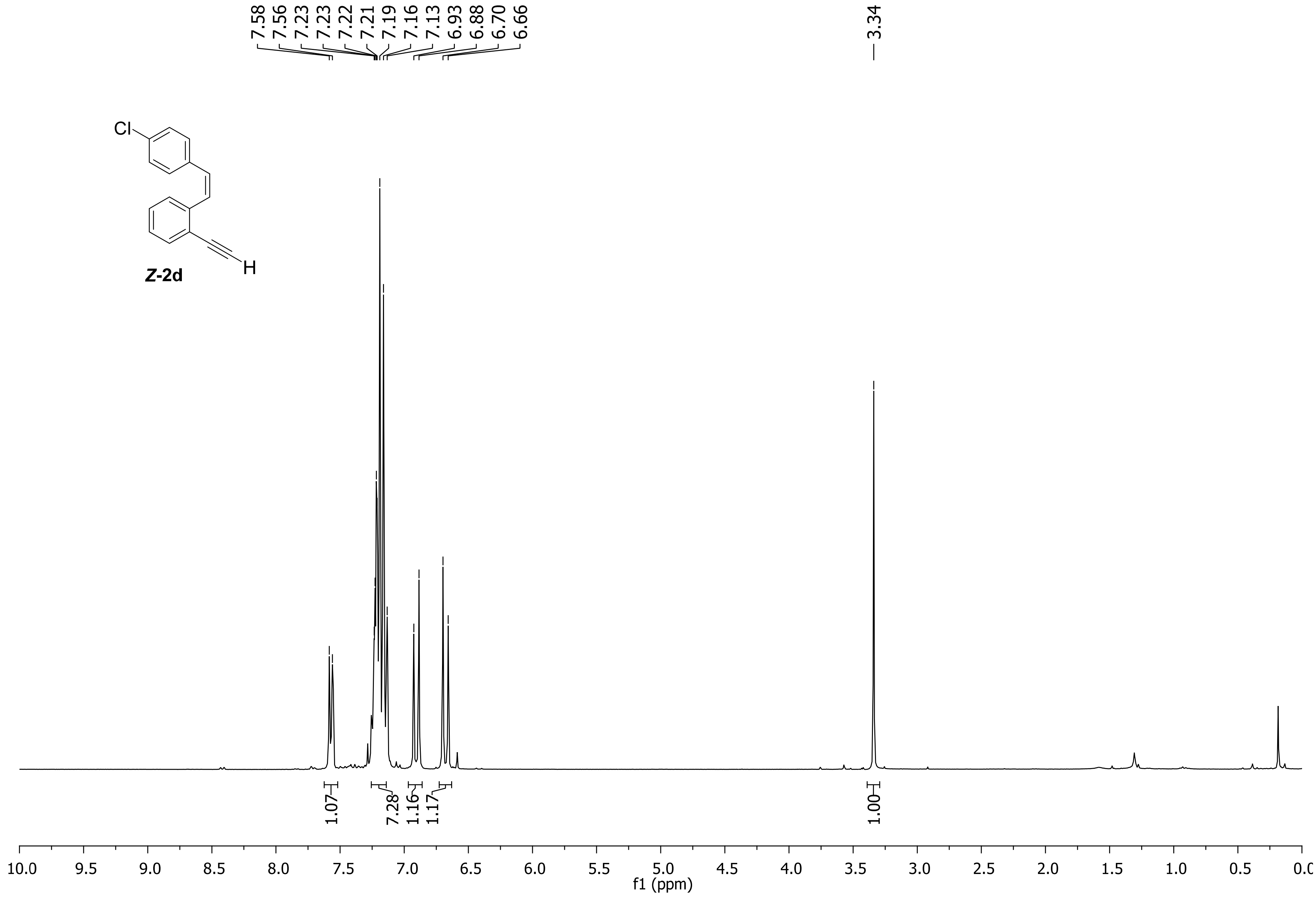

^{13}C NMR (75.4 MHz, $CDCl_3$)

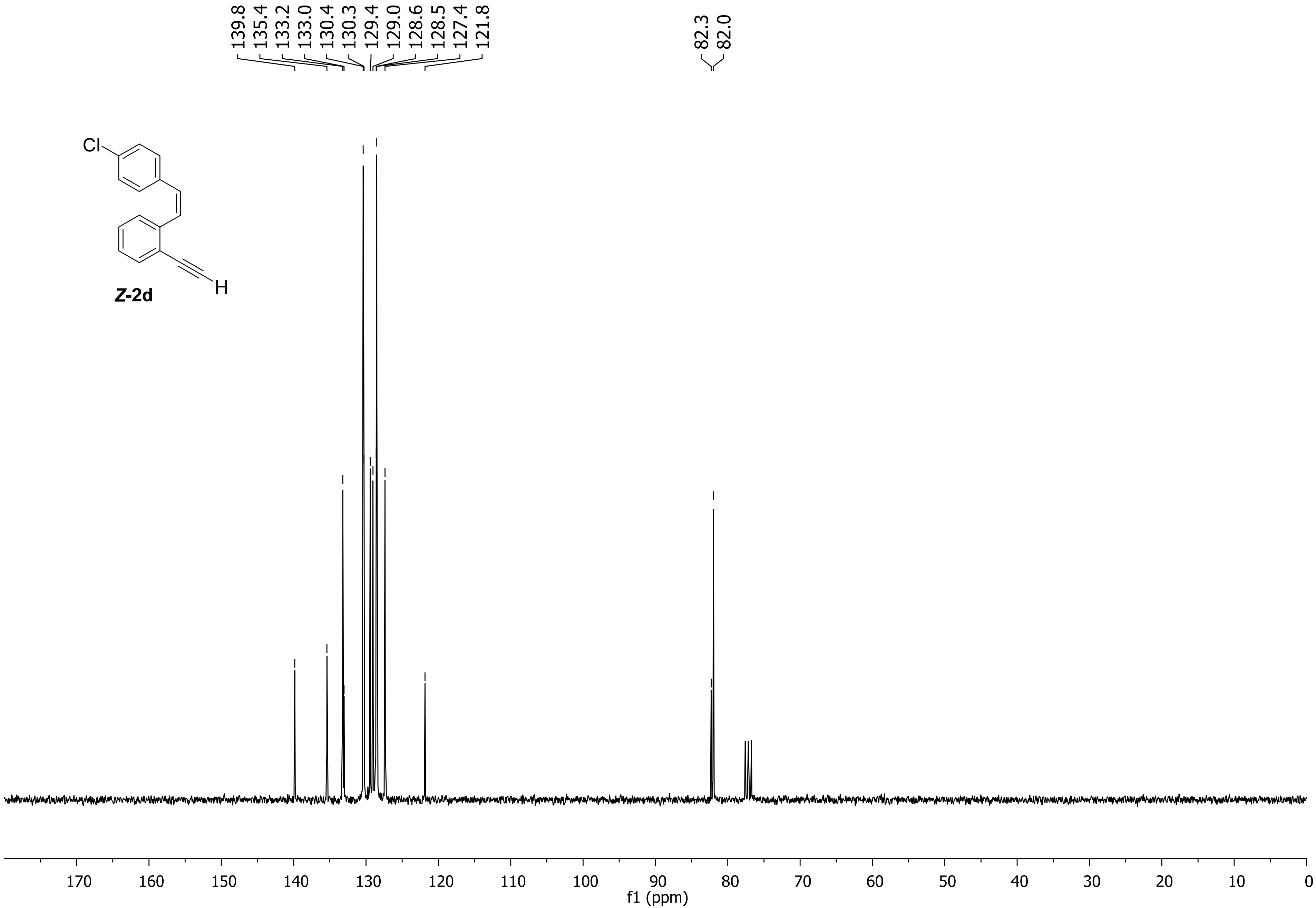

^{1}H NMR (300 MHz, $CDCl_3$)

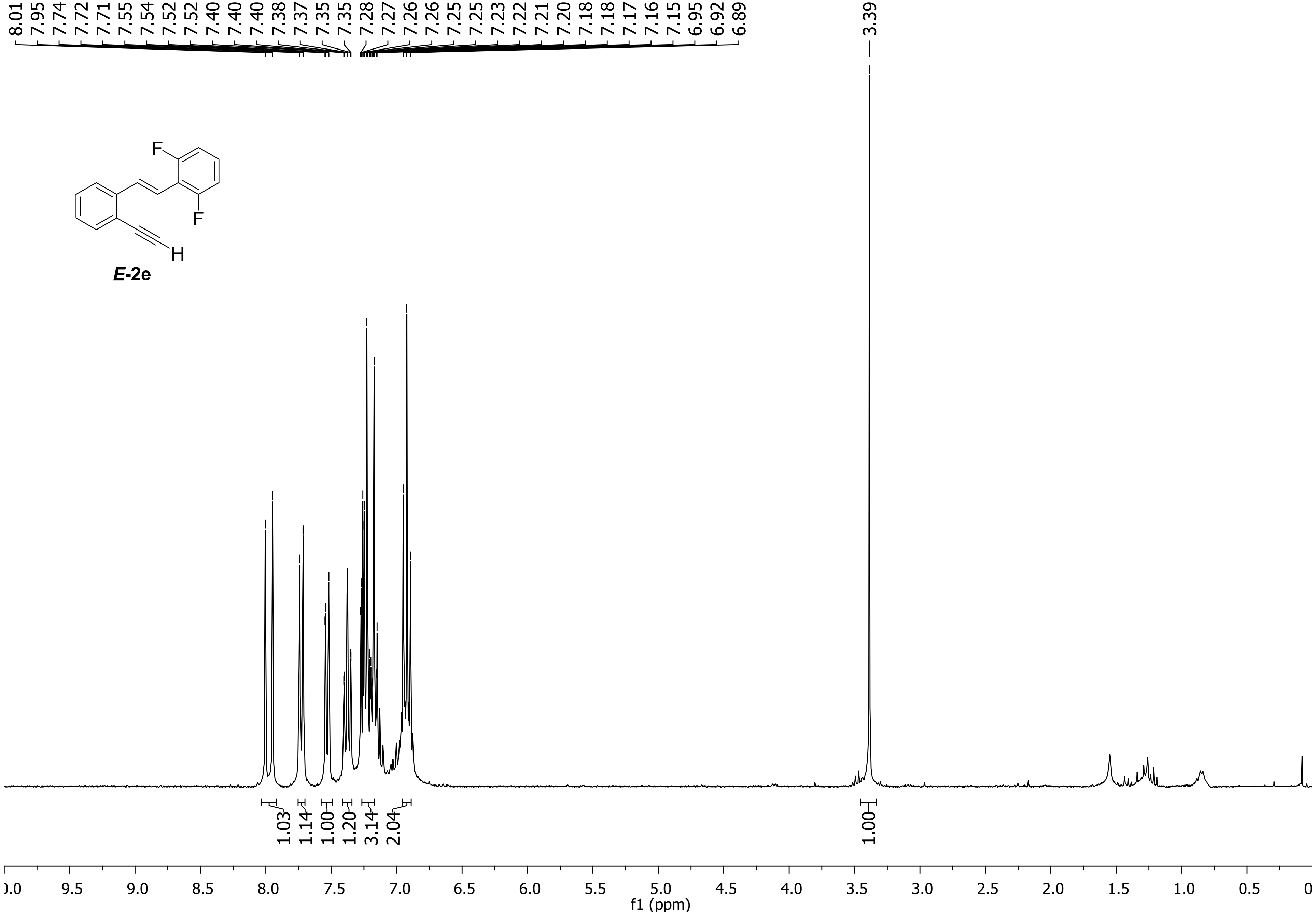

^{13}C NMR (75.4 MHz, $CDCl_3$)

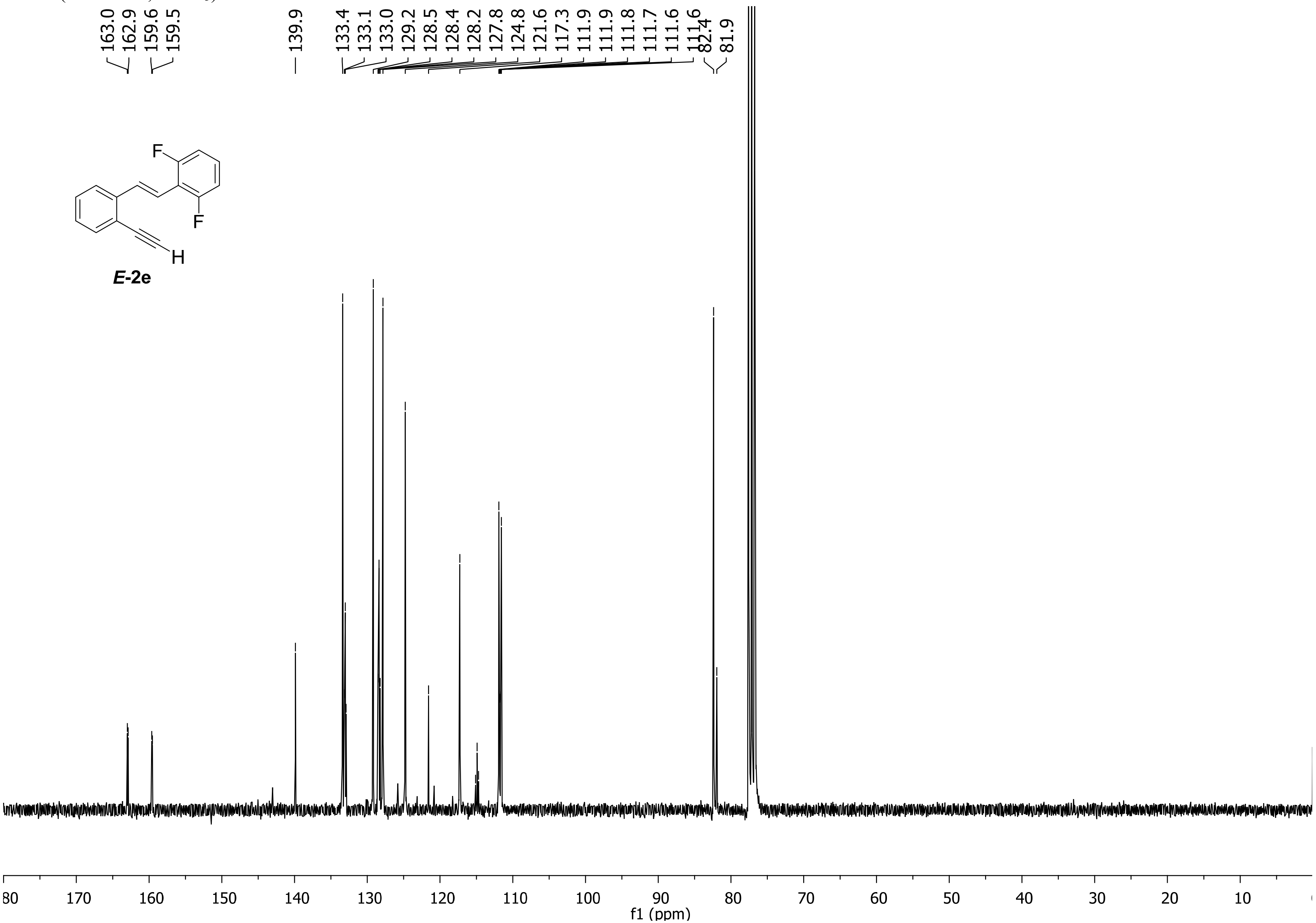

^{1}H NMR (300 MHz, $CDCl_3$)

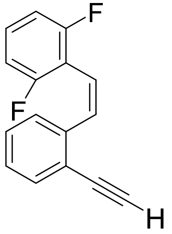

***Z*-2e**

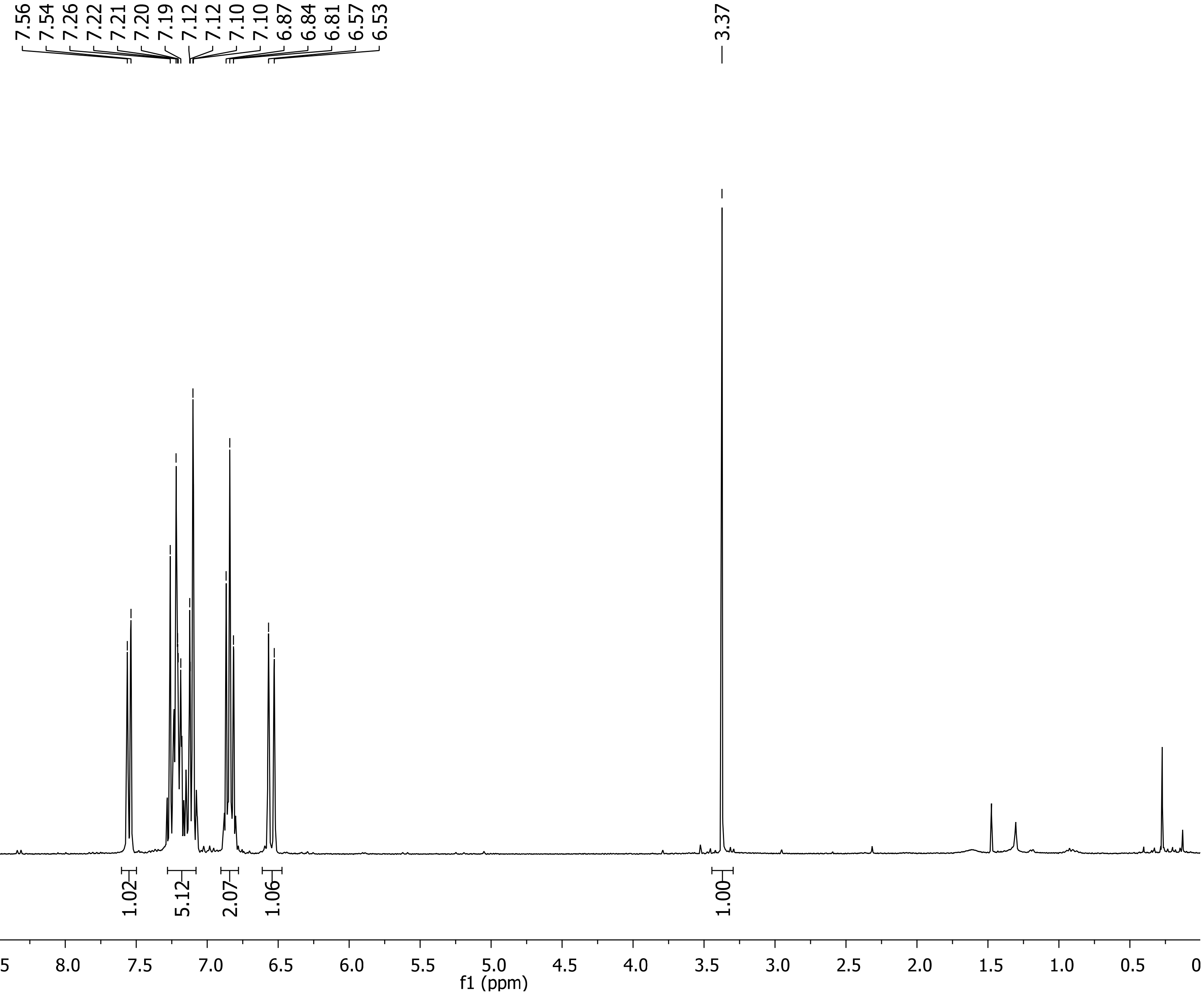

^{13}C NMR (75.4 MHz, $CDCl_3$)

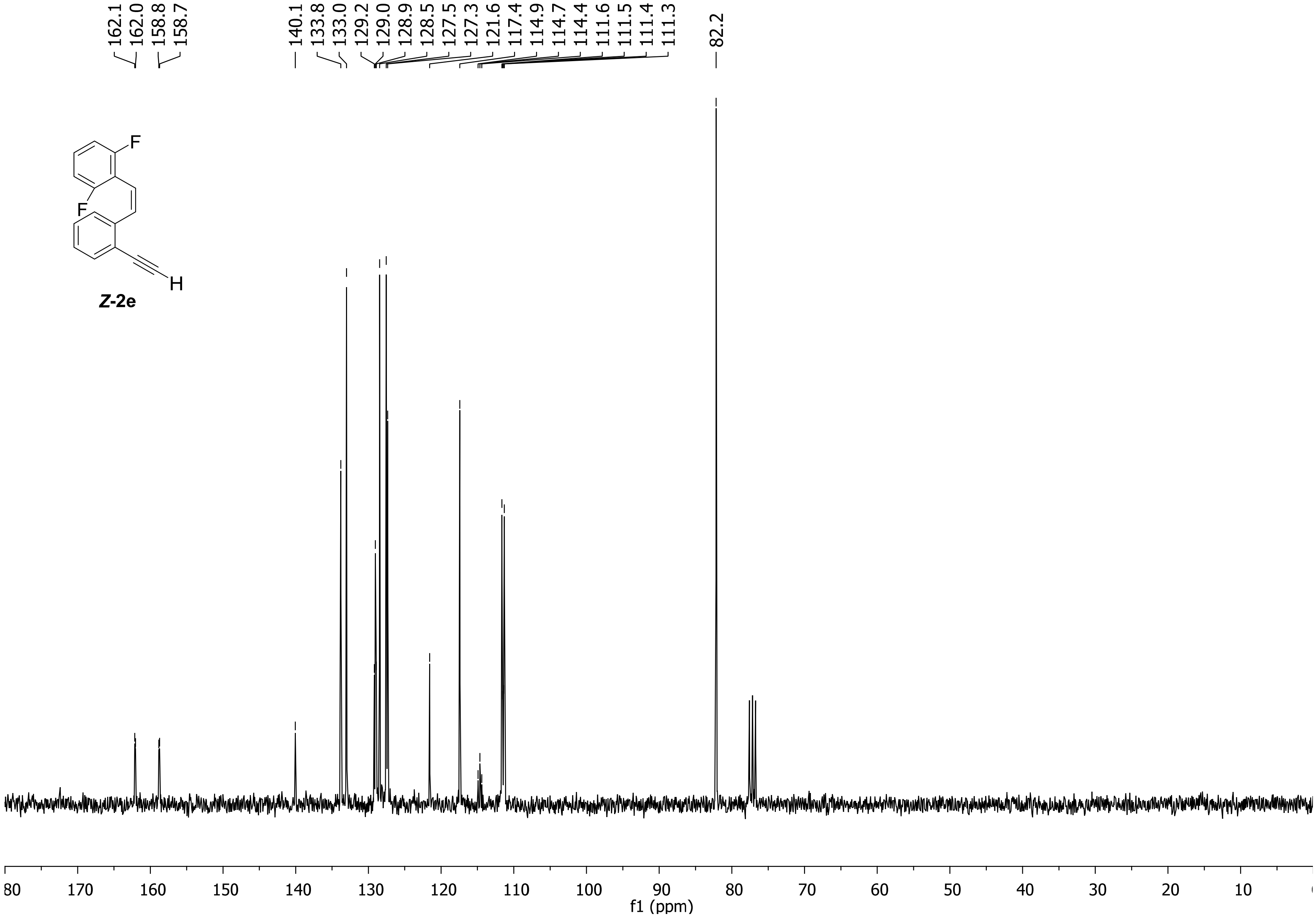

***Z*-2e**

^{1}H NMR (300 MHz, $CDCl_3$)

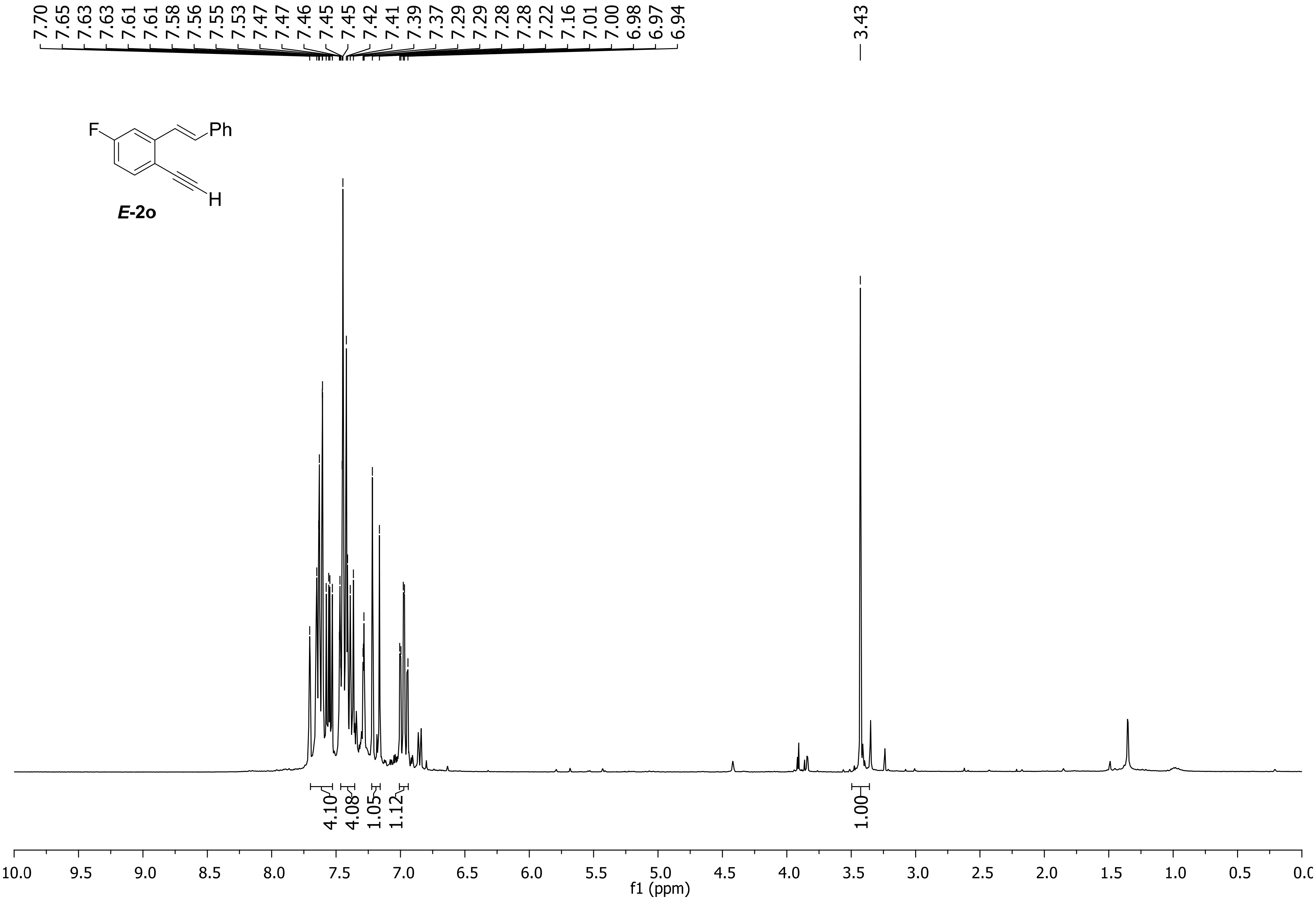

^{13}C NMR (75.4 MHz, $CDCl_3$)

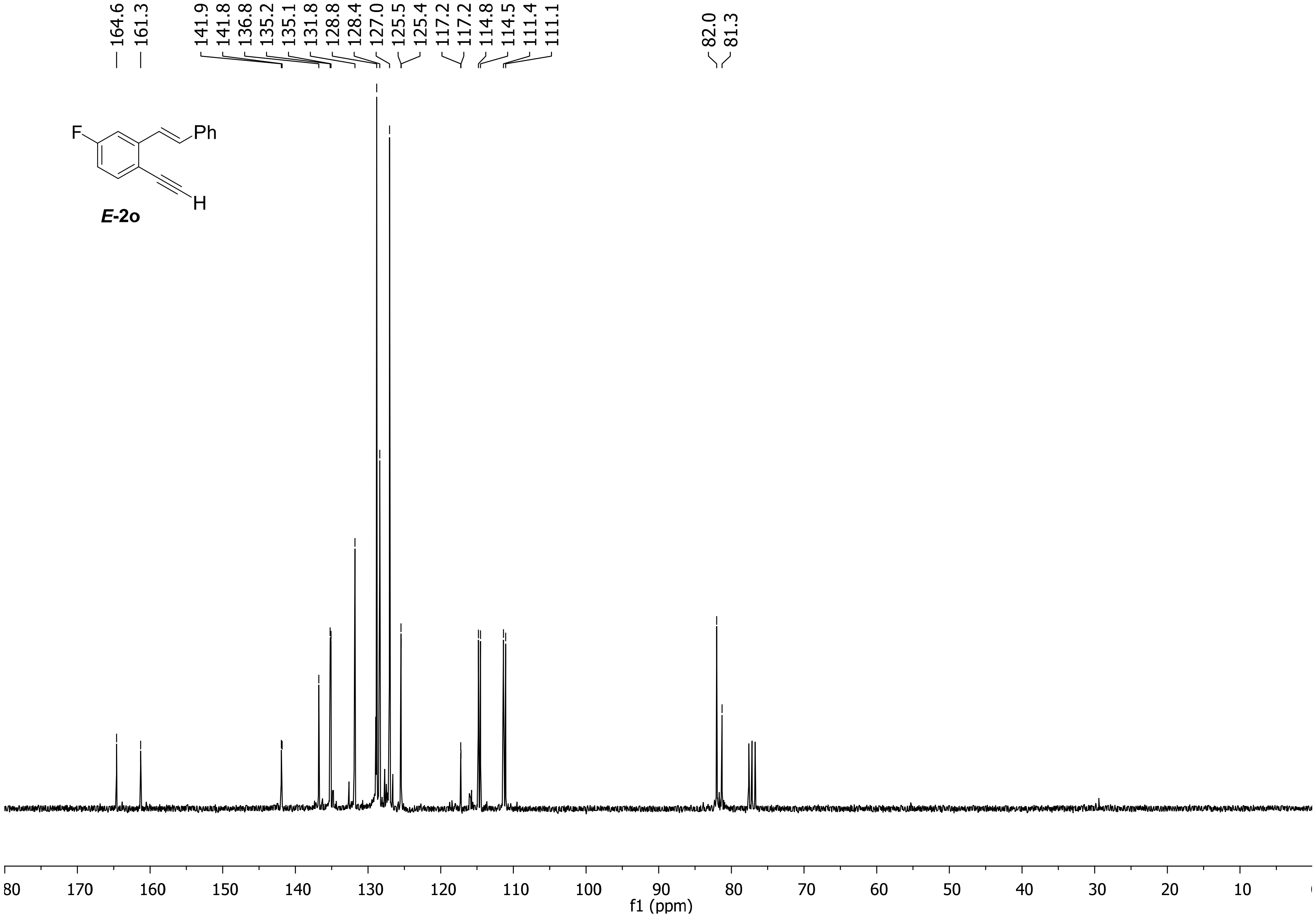

^{1}H NMR (300 MHz, $CDCl_3$)

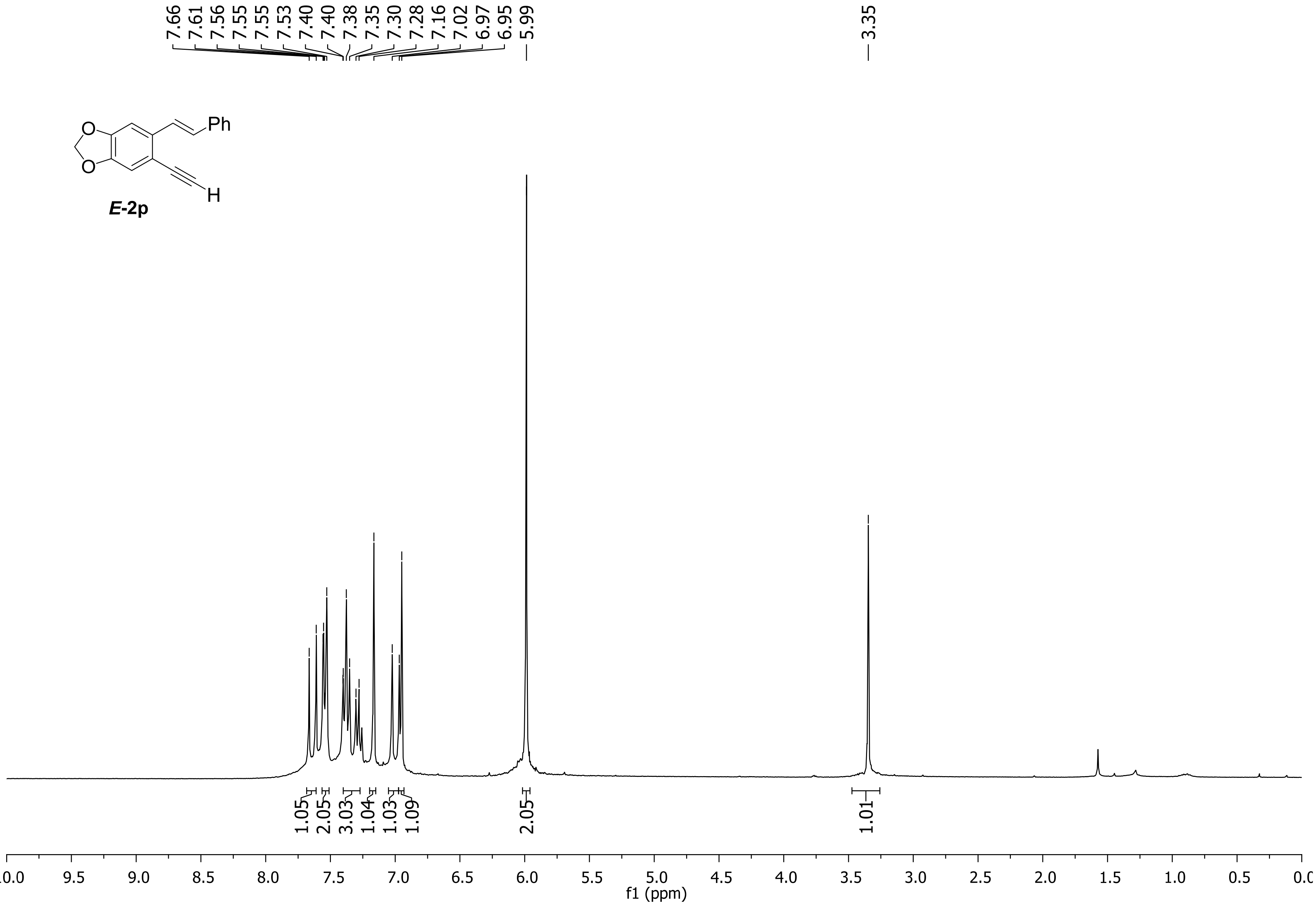

^{13}C NMR (75.4 MHz, $CDCl_3$)

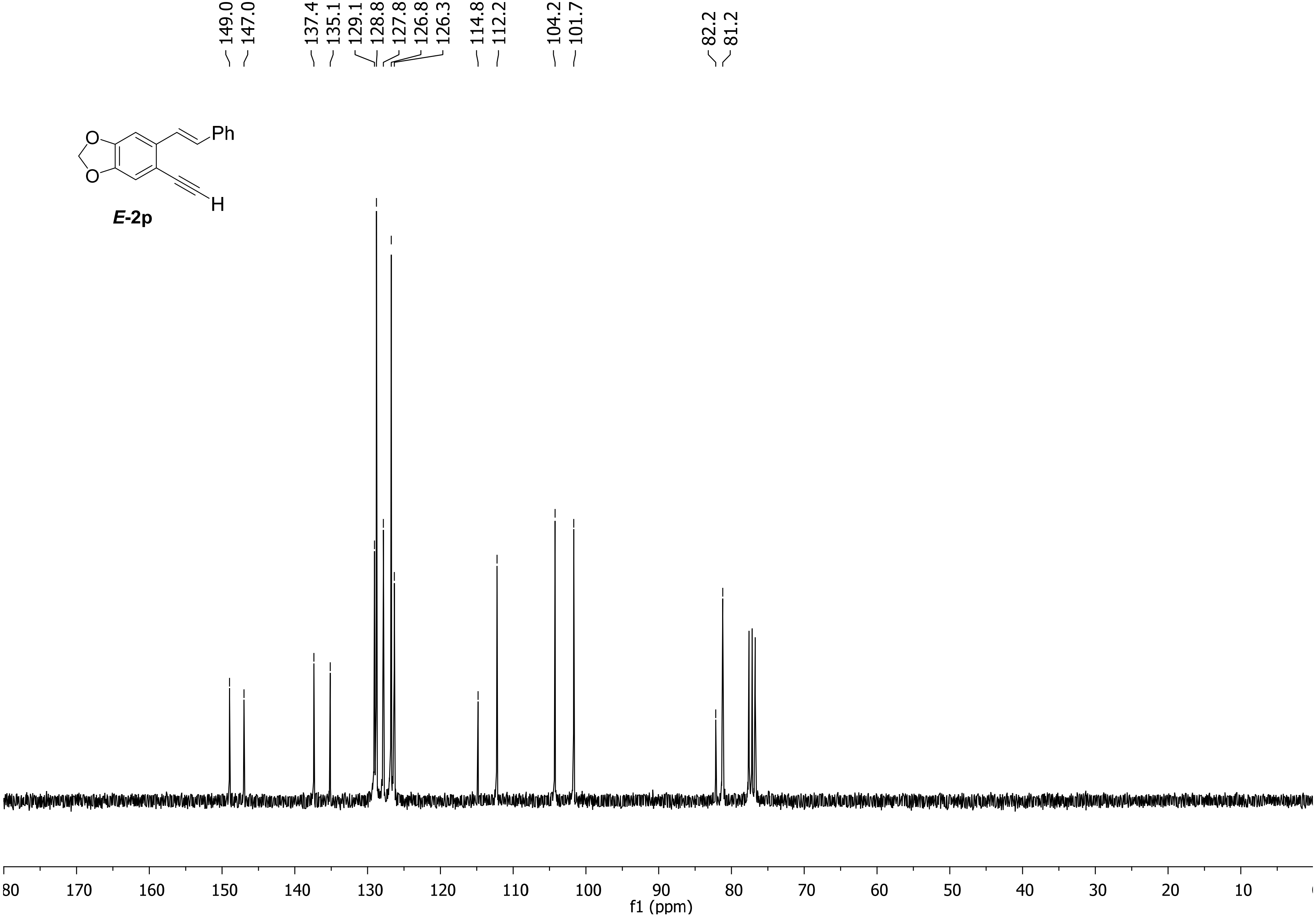

^{1}H NMR (300 MHz, $CDCl_3$)

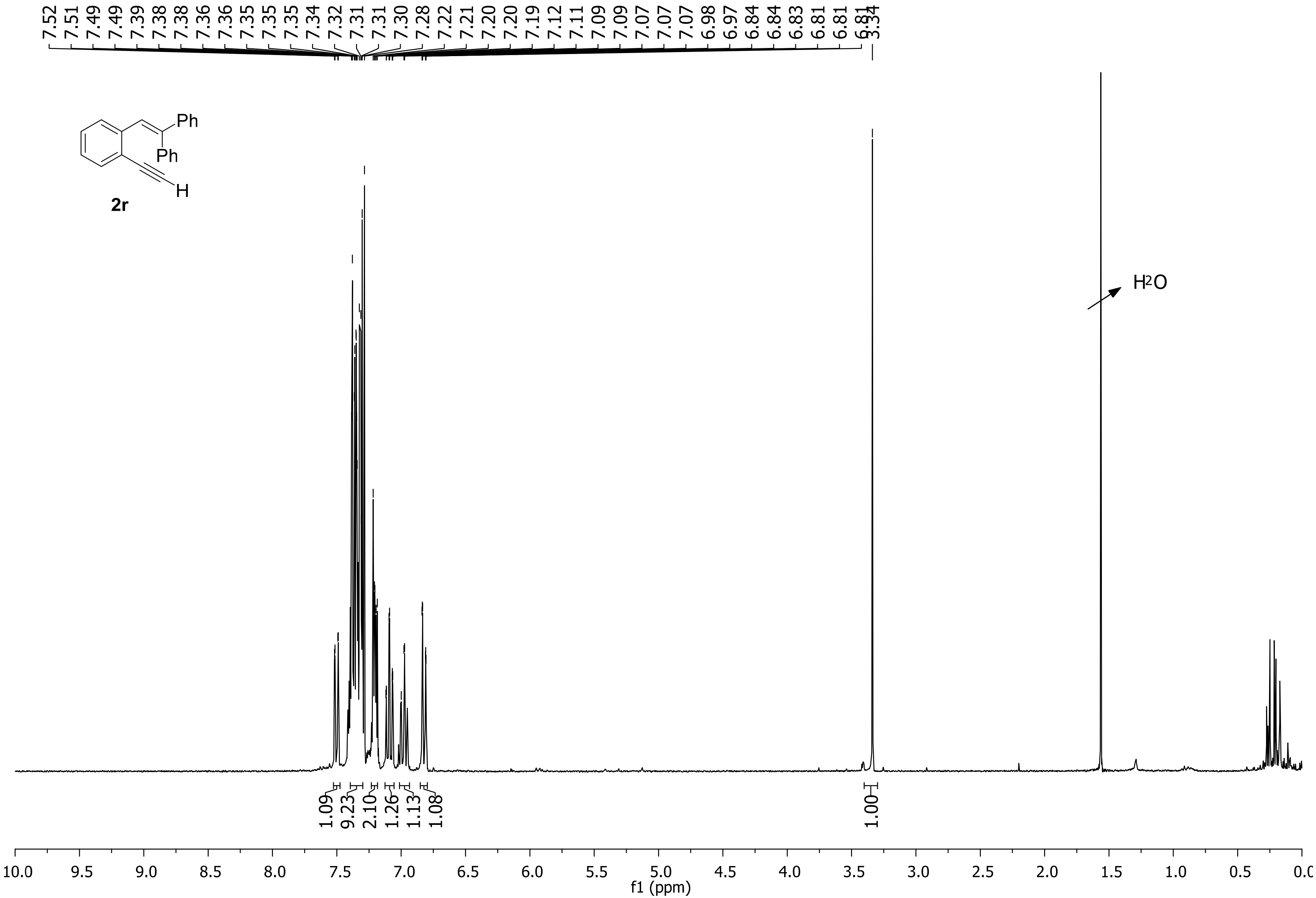

^{13}C NMR (75.4 MHz, $CDCl_3$)

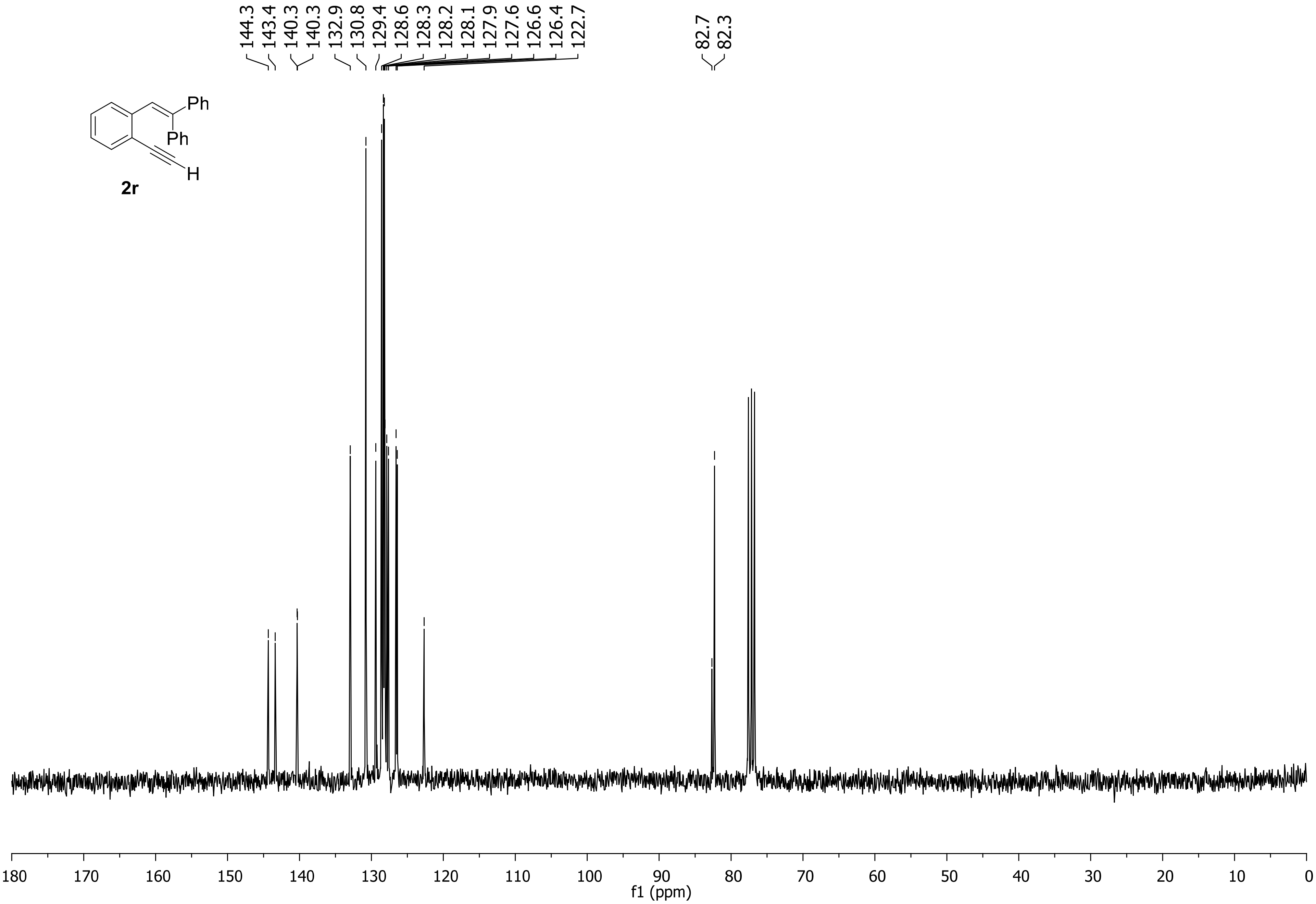

^{1}H NMR (300 MHz, $CDCl_3$)

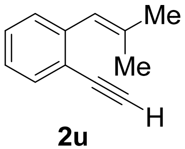

2u

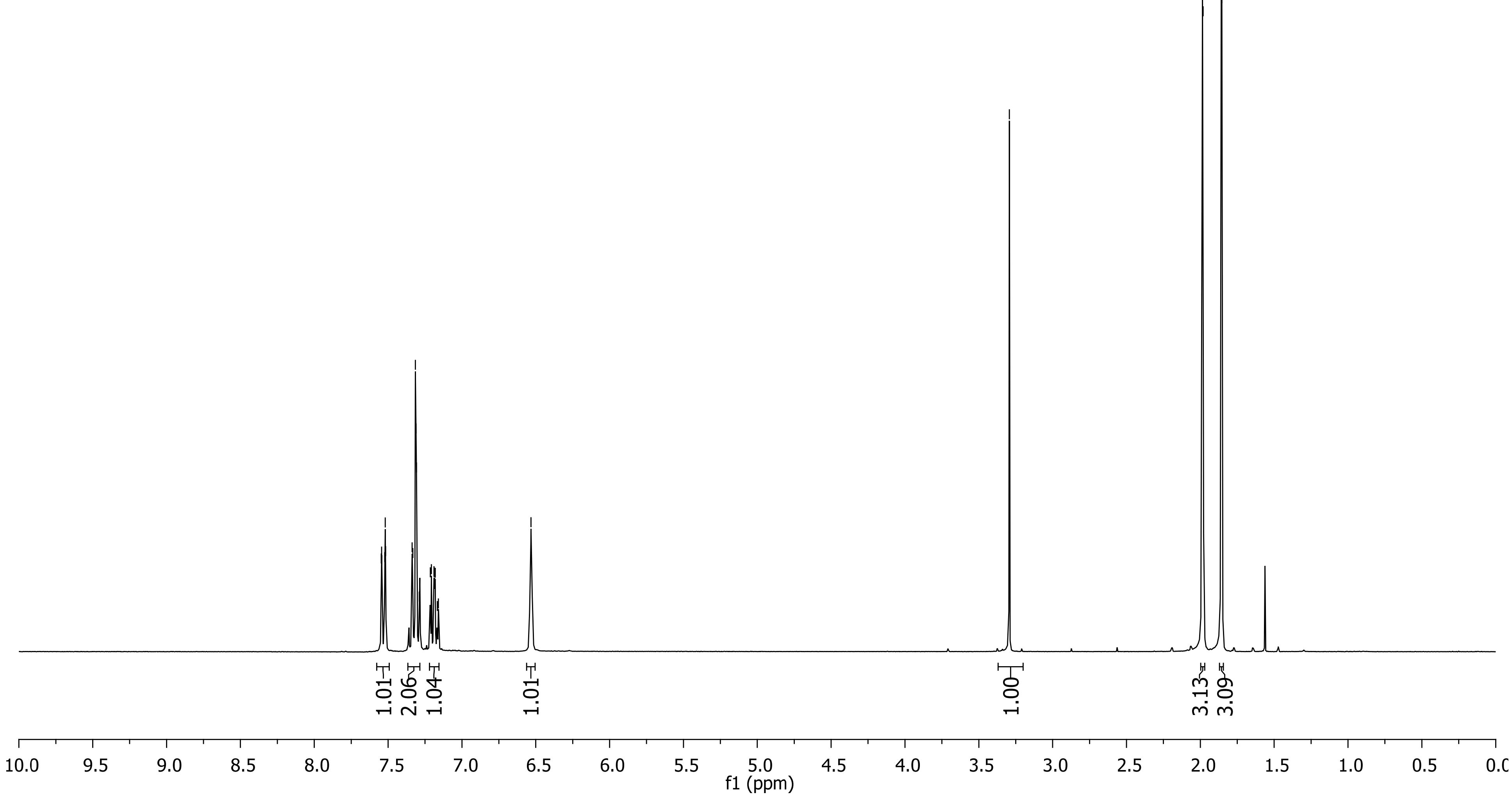

^{13}C NMR (75.4 MHz, $CDCl_3$)

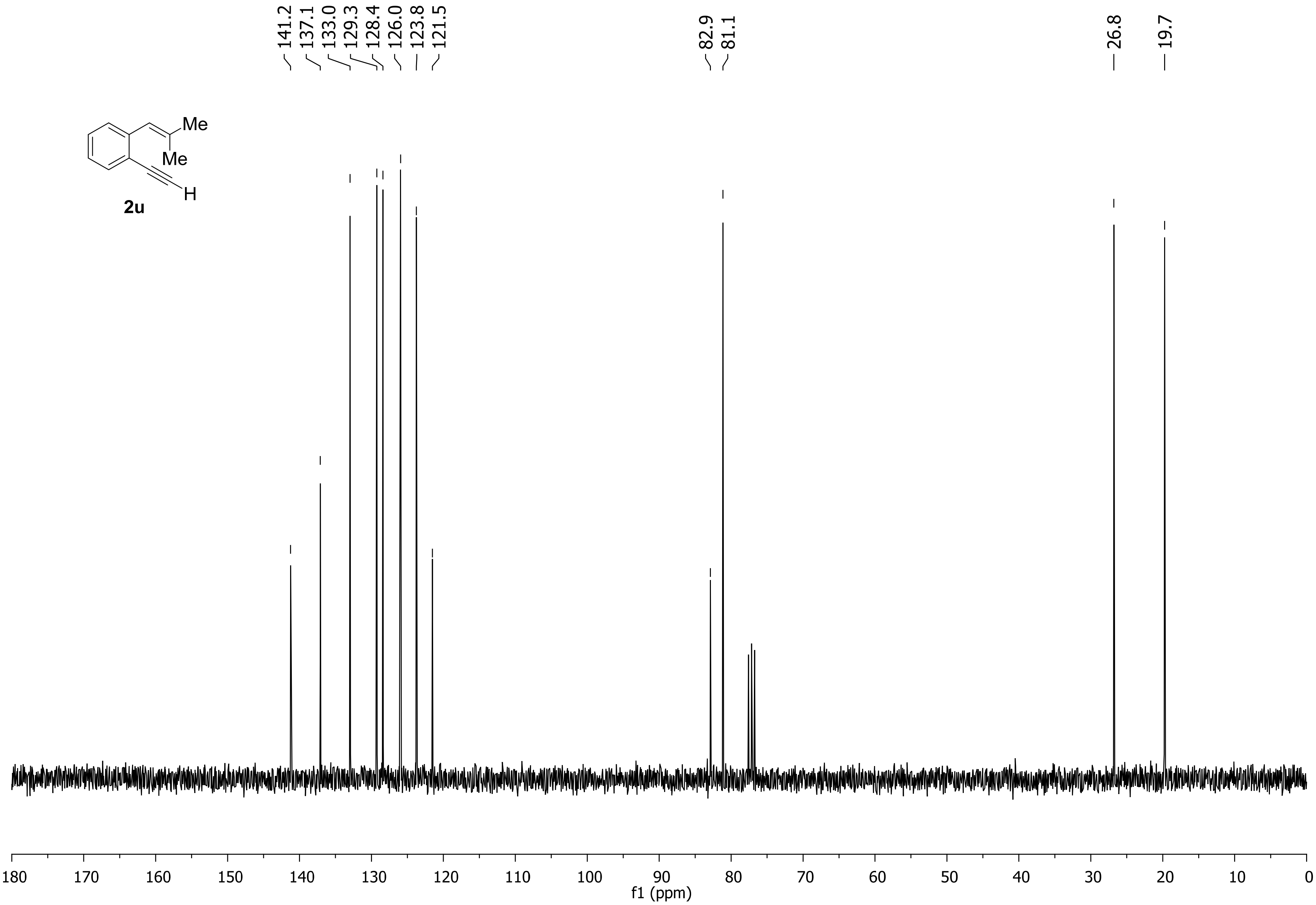

^{1}H NMR (300 MHz, $CDCl_3$)

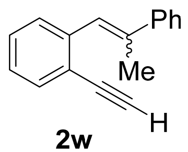

2w
(*E*/*Z* = 2/1)

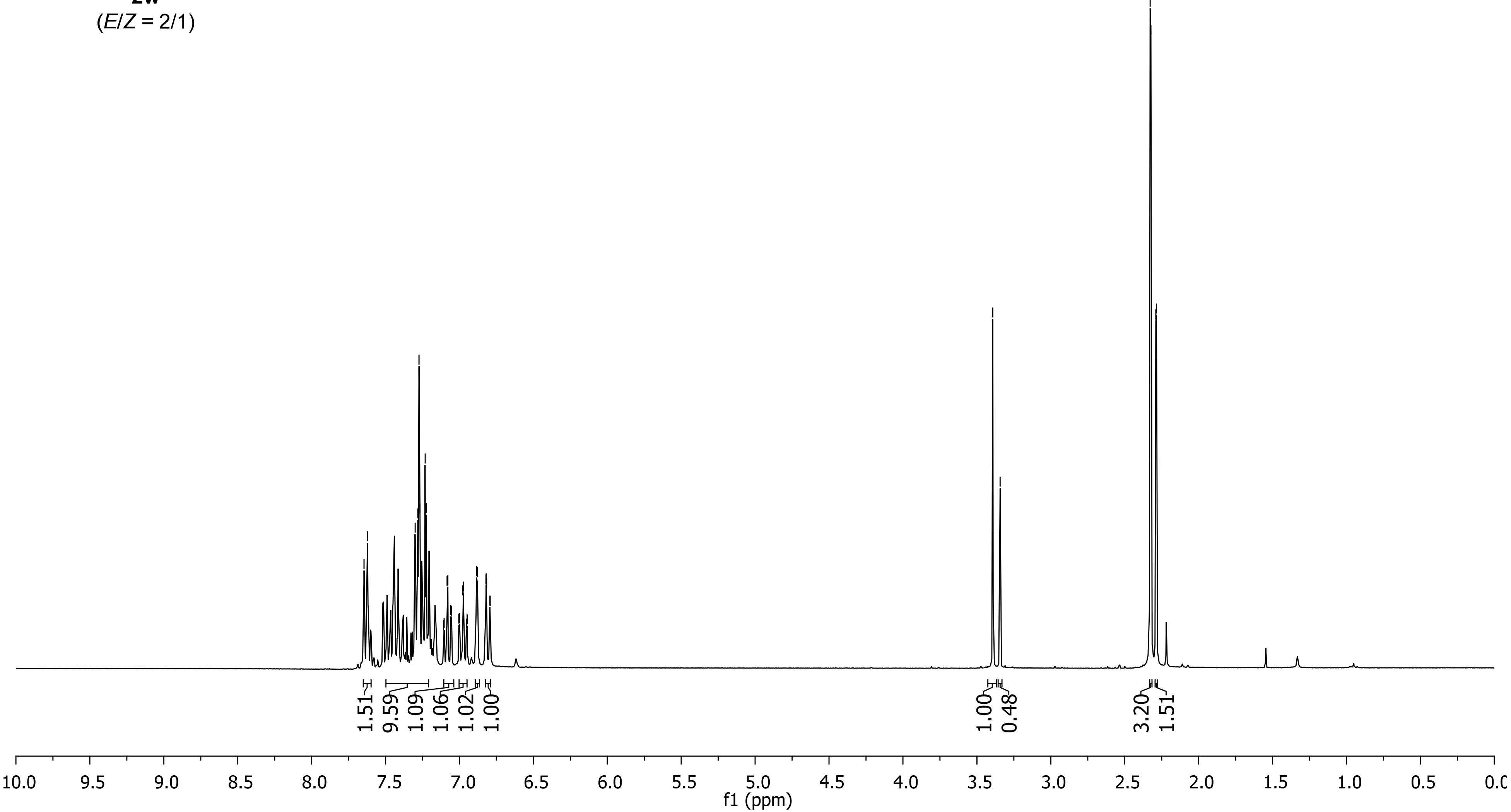

^{13}C NMR (75.4 MHz, $CDCl_3$)

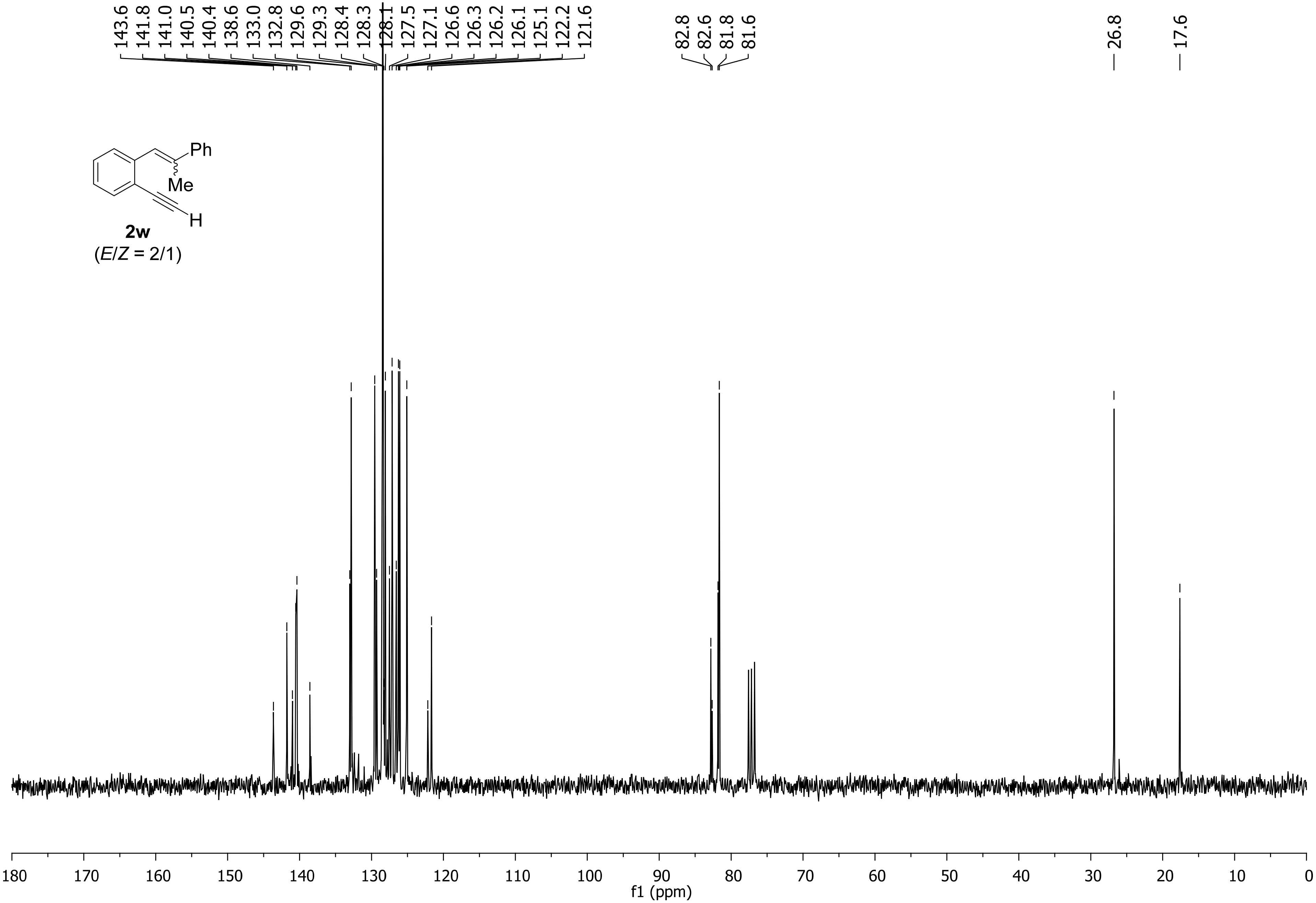

^{1}H NMR (300 MHz, $CDCl_3$)

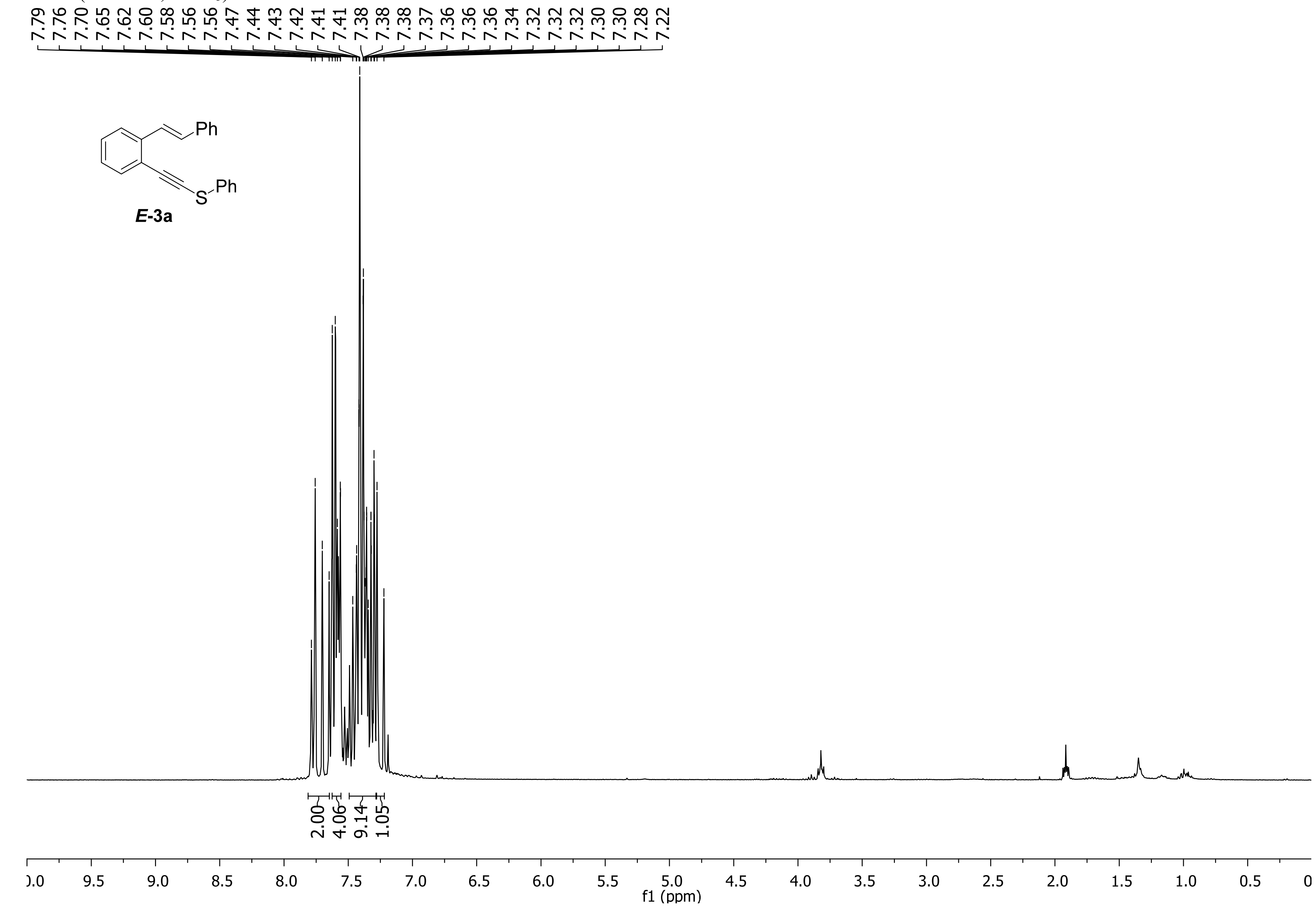

***E*-3a**

^{13}C NMR (75.4 MHz, $CDCl_3$)

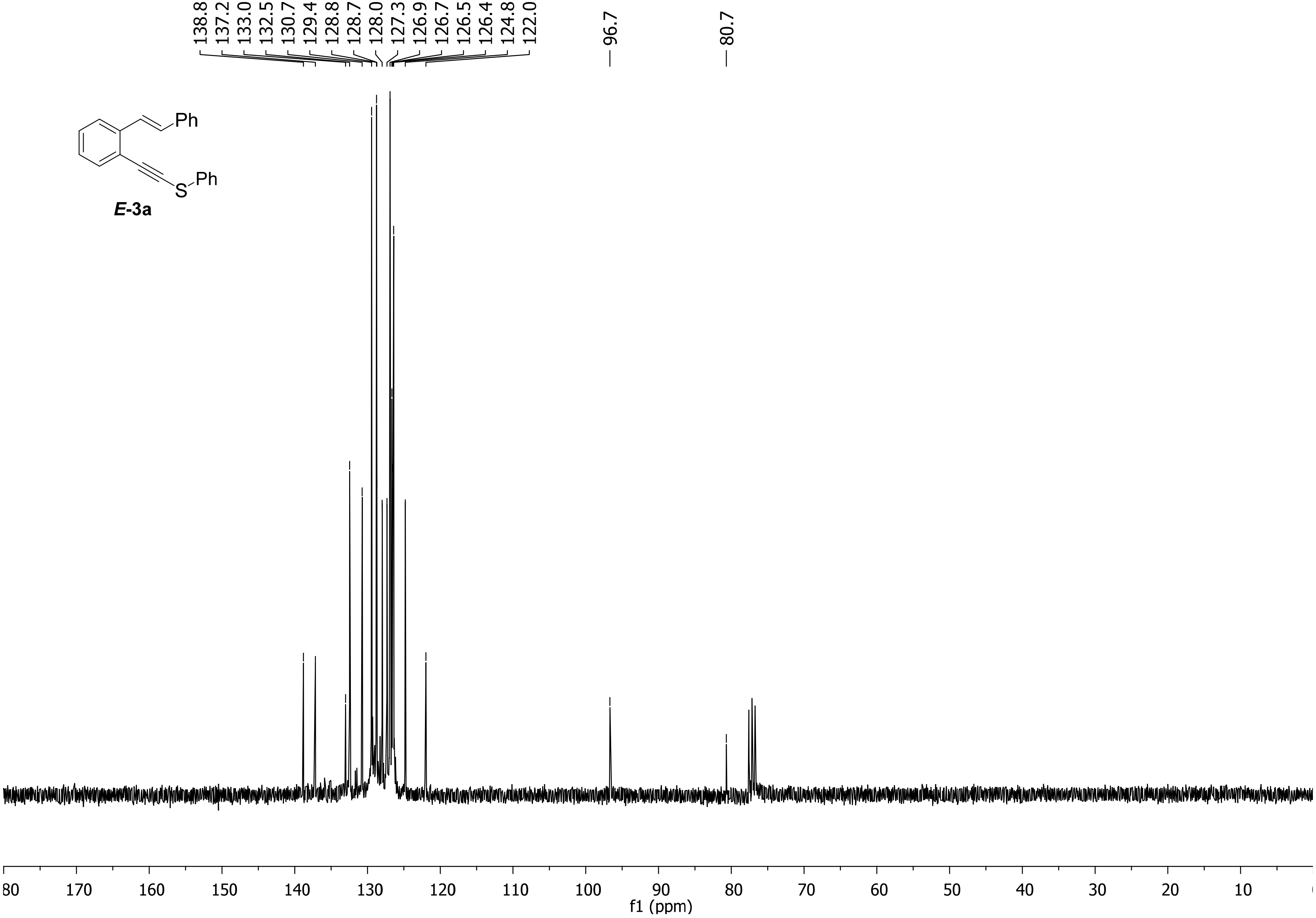

^{1}H NMR (300 MHz, $CDCl_3$)

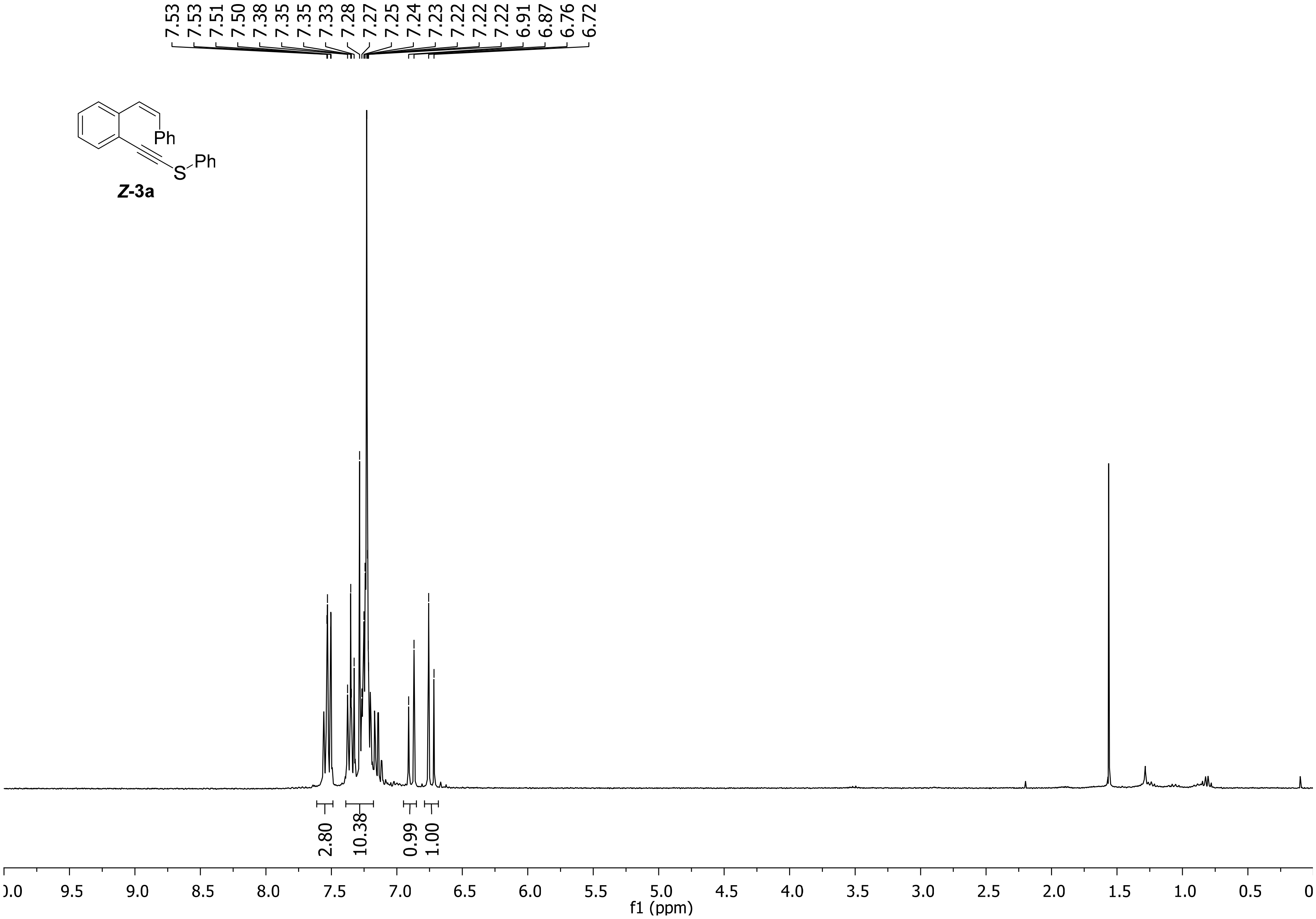

^{13}C NMR (75.4 MHz, $CDCl_3$)

Z-3a

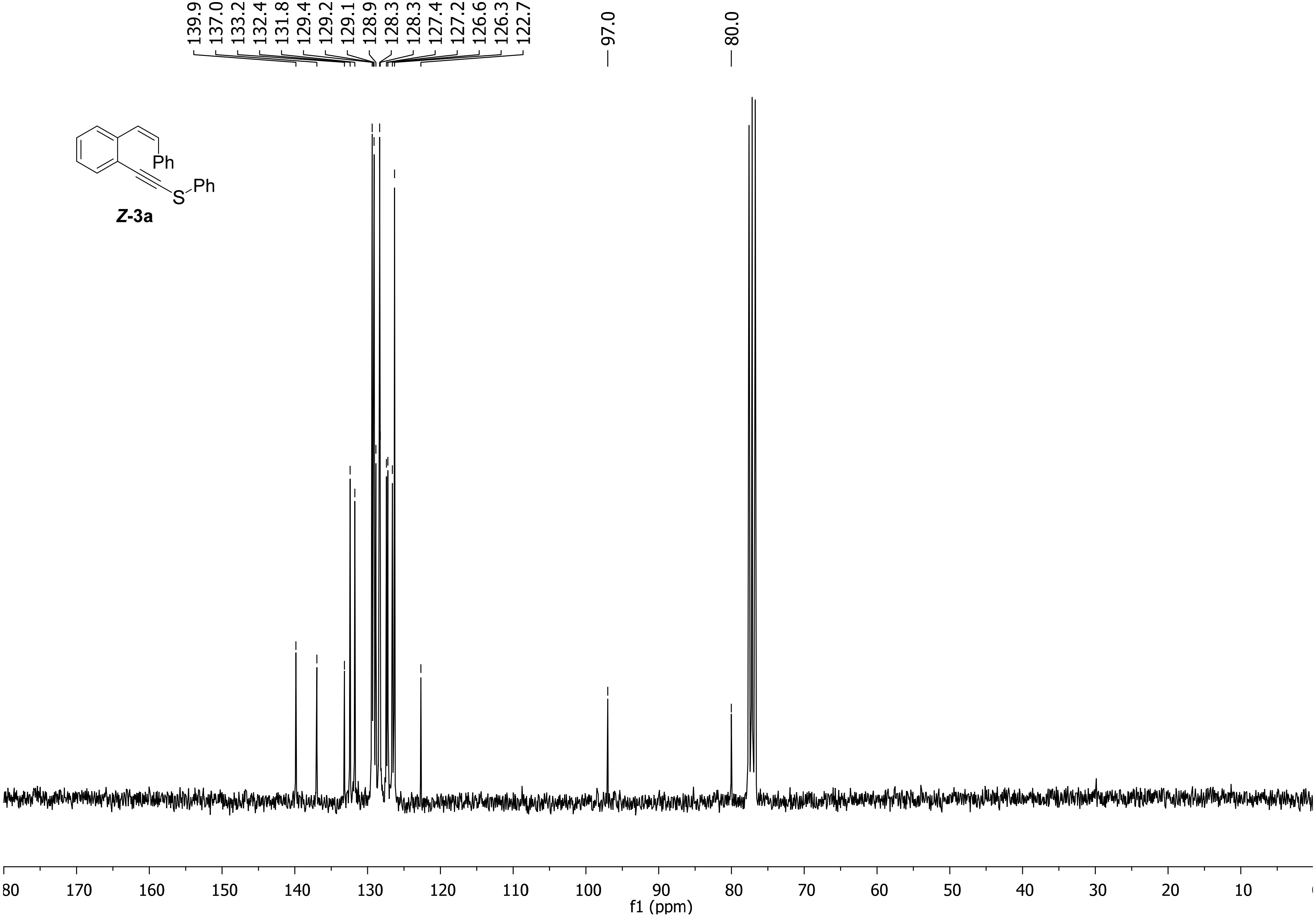

^{1}H NMR (300 MHz, $CDCl_3$)

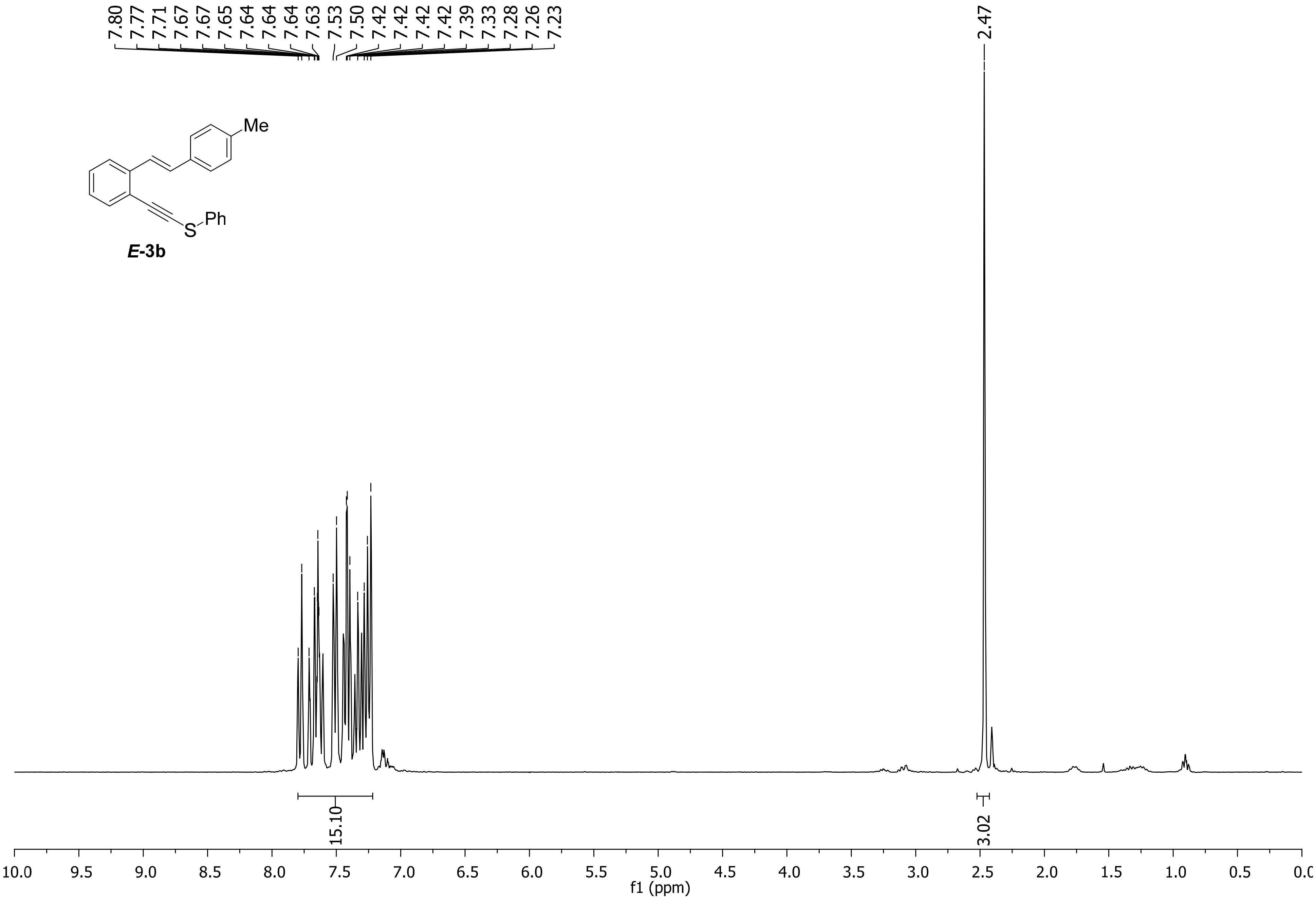

^{13}C NMR (75.4 MHz, $CDCl_3$)

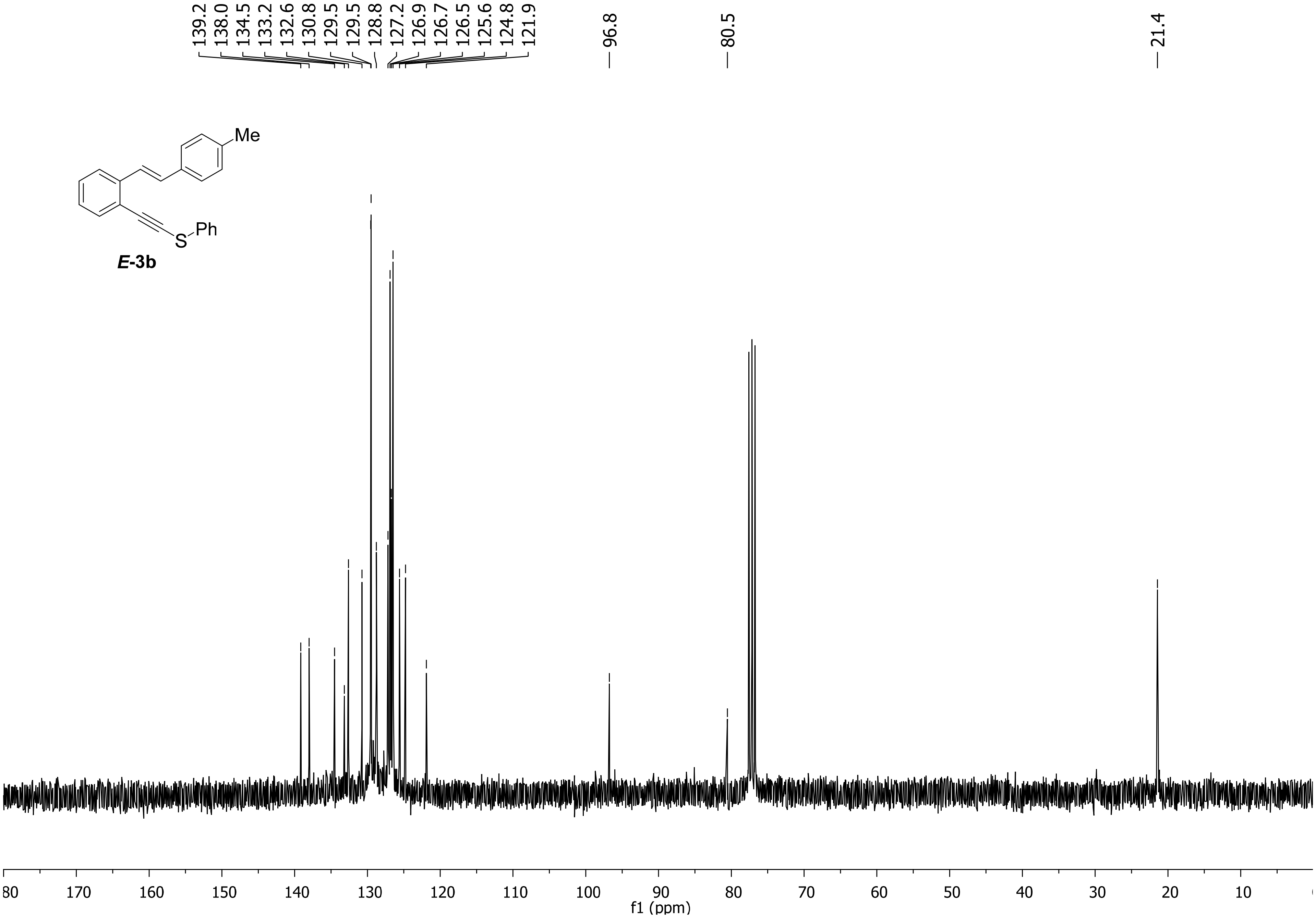

***E*-3b**

^{1}H NMR (300 MHz, $CDCl_3$)

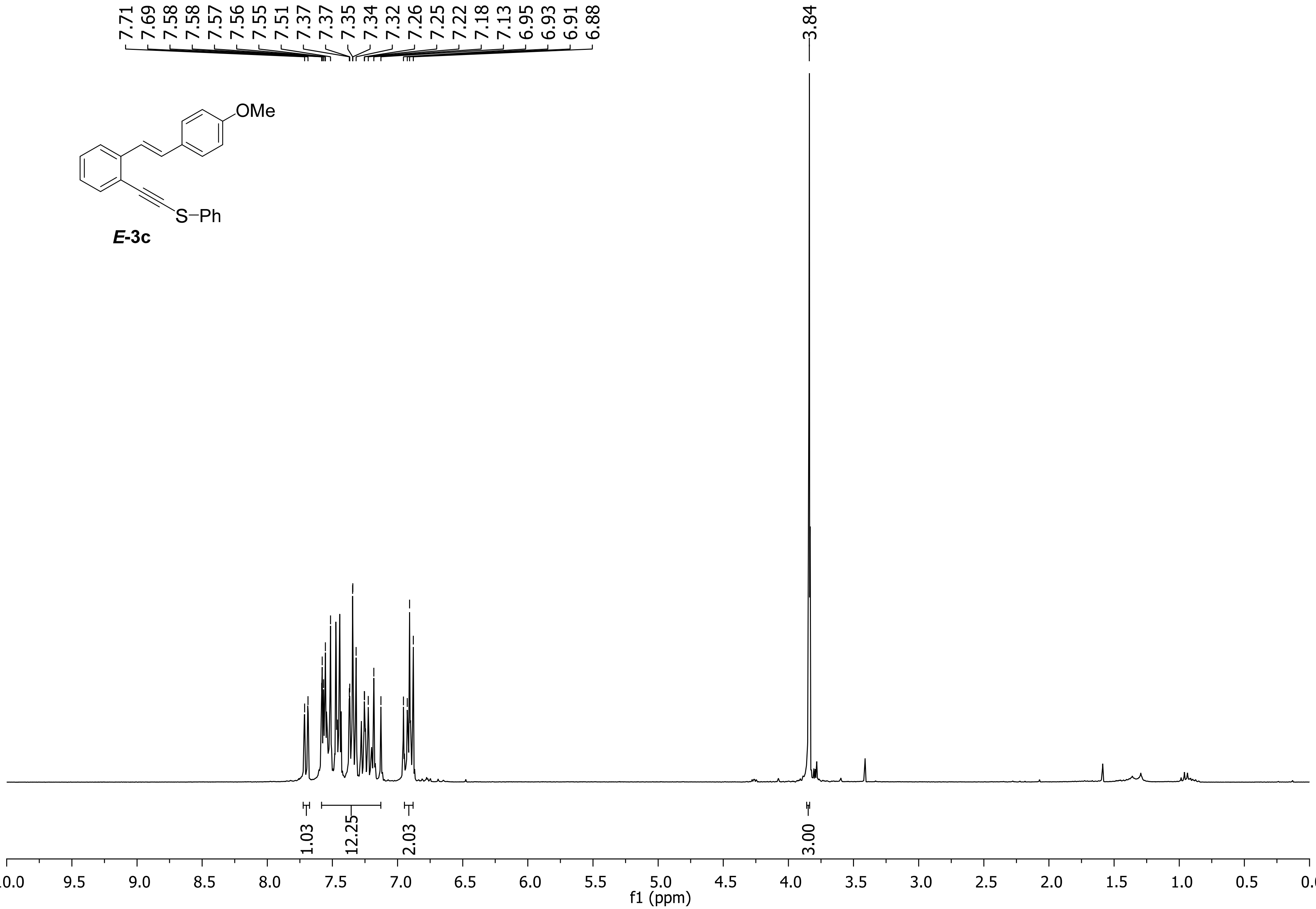

^{13}C NMR (75.4 MHz, $CDCl_3$)

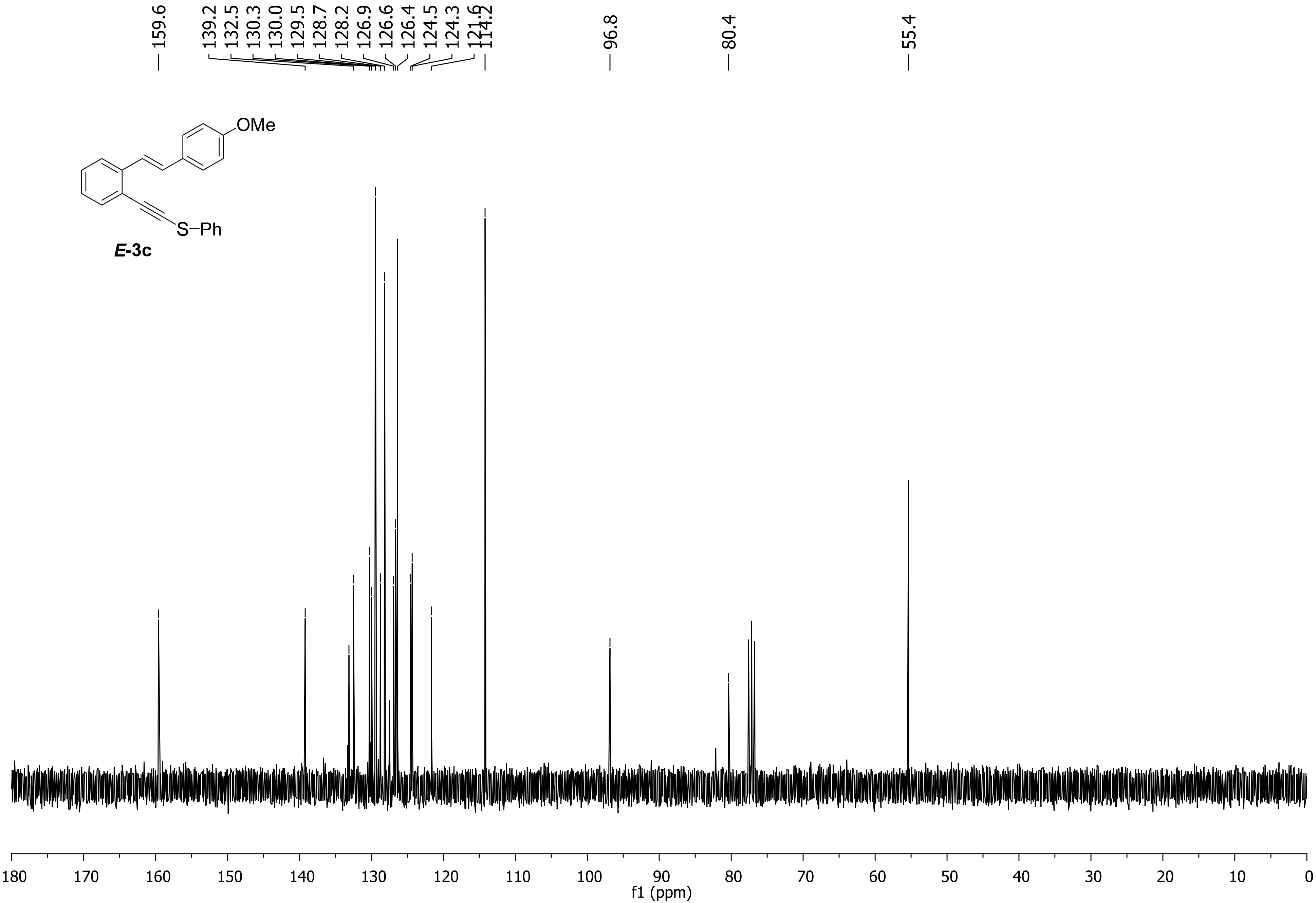

^{1}H NMR (300 MHz, $CDCl_3$)

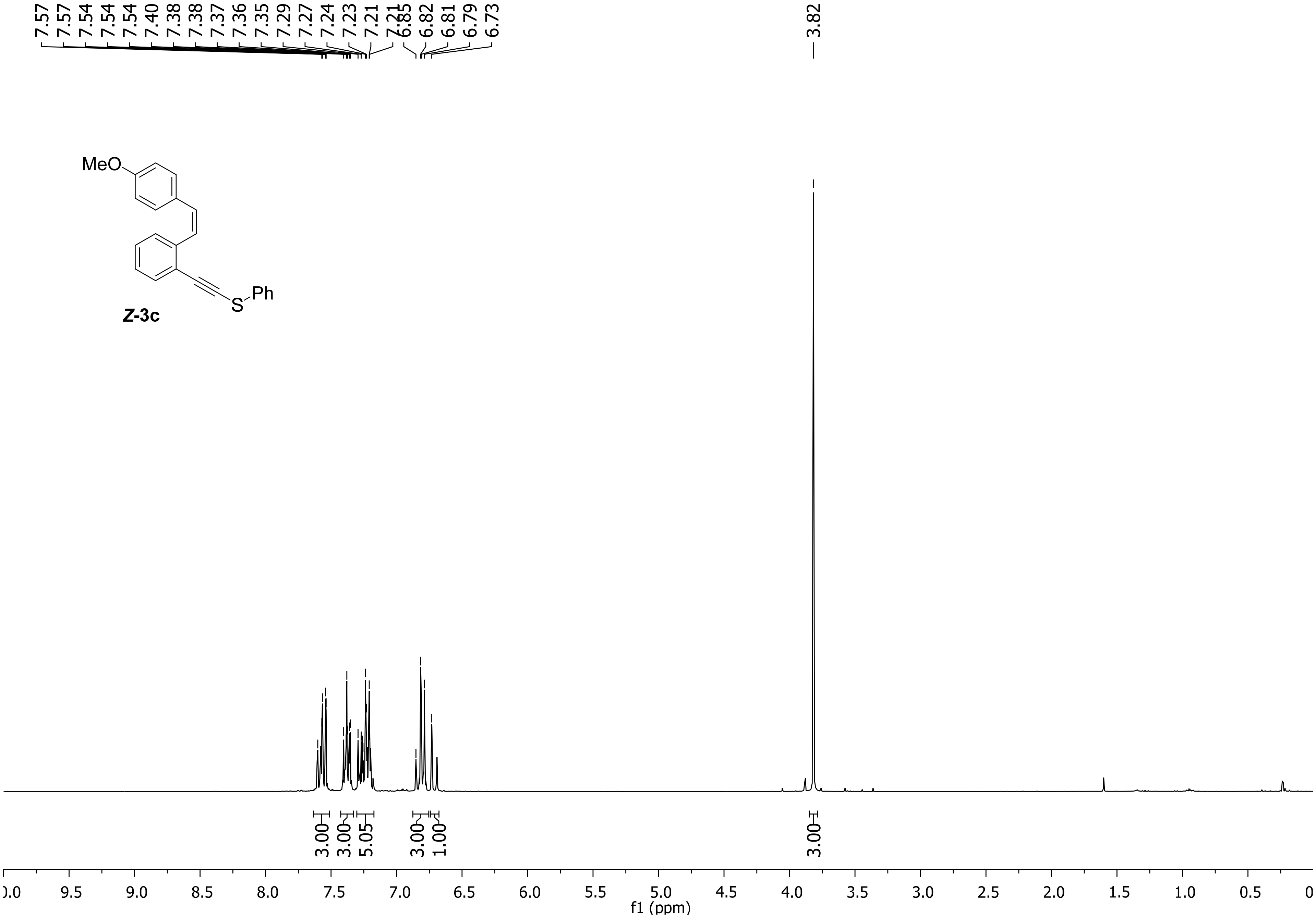

^{13}C NMR (75.4 MHz, $CDCl_3$)

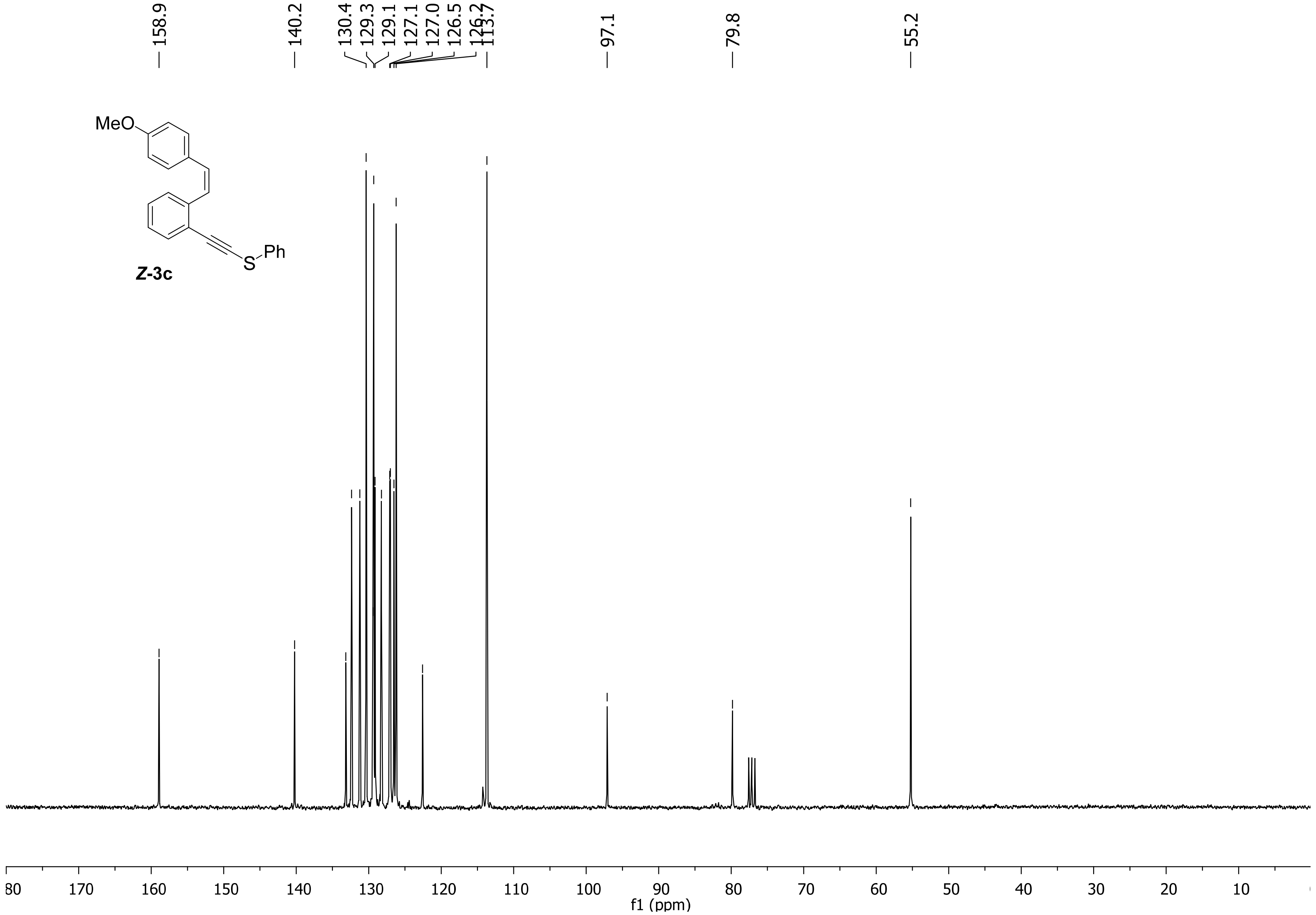

^{1}H NMR (300 MHz, $CDCl_3$)

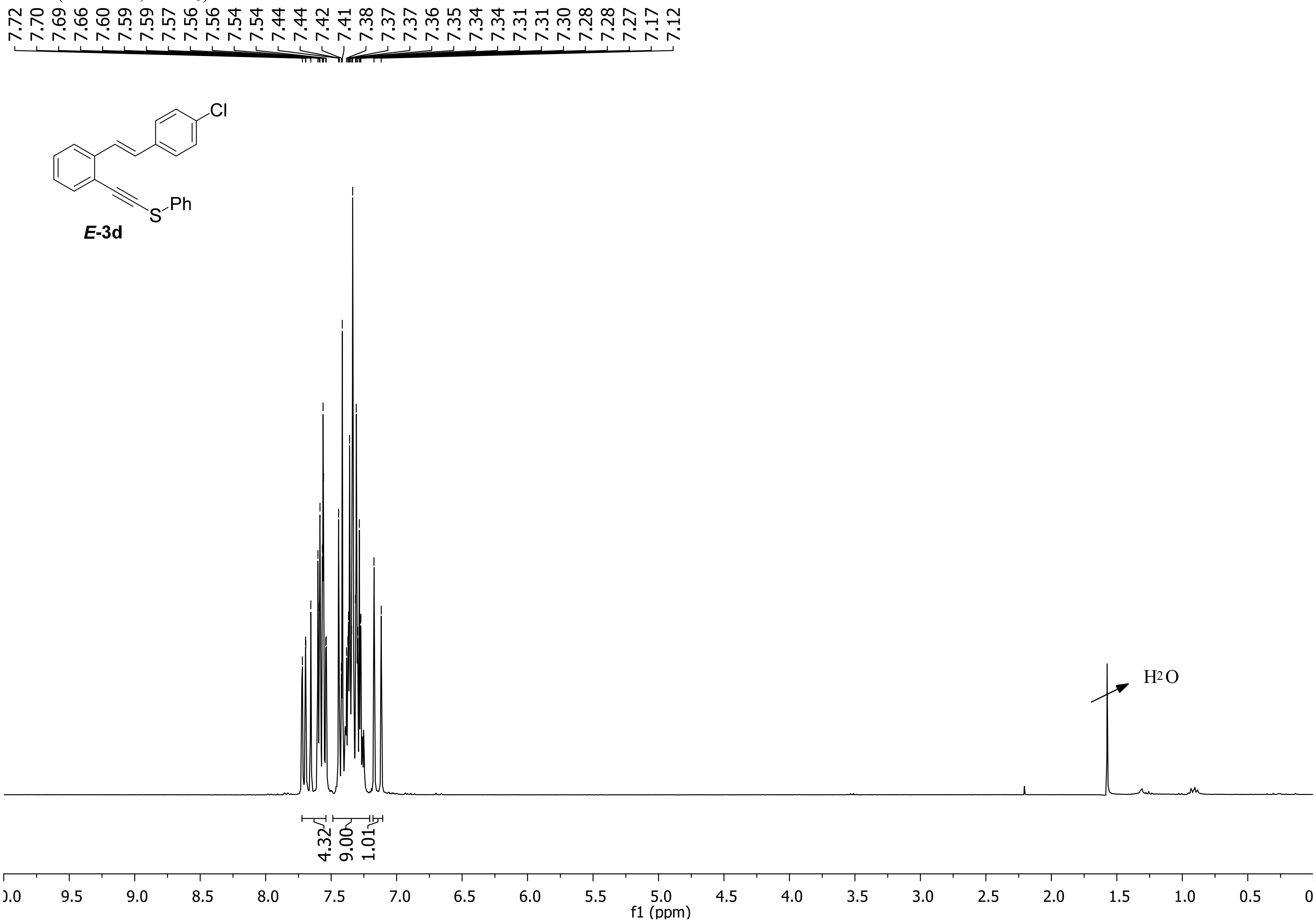

^{13}C NMR (75.4 MHz, $CDCl_3$)

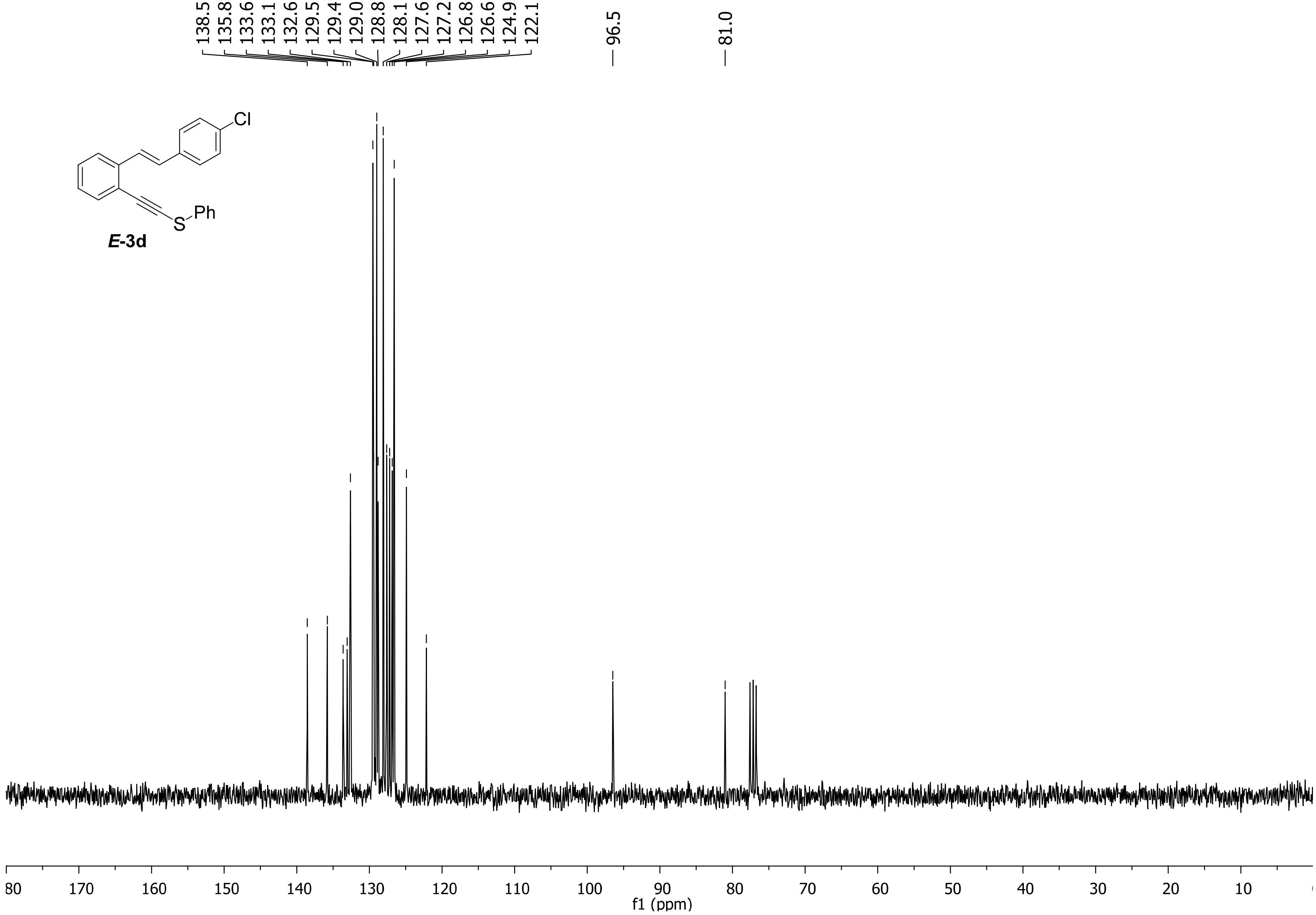

^{1}H NMR (300 MHz, $CDCl_3$)

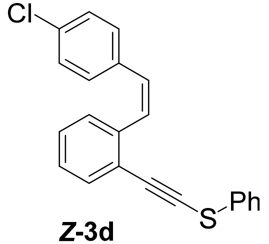

***Z*-3d**

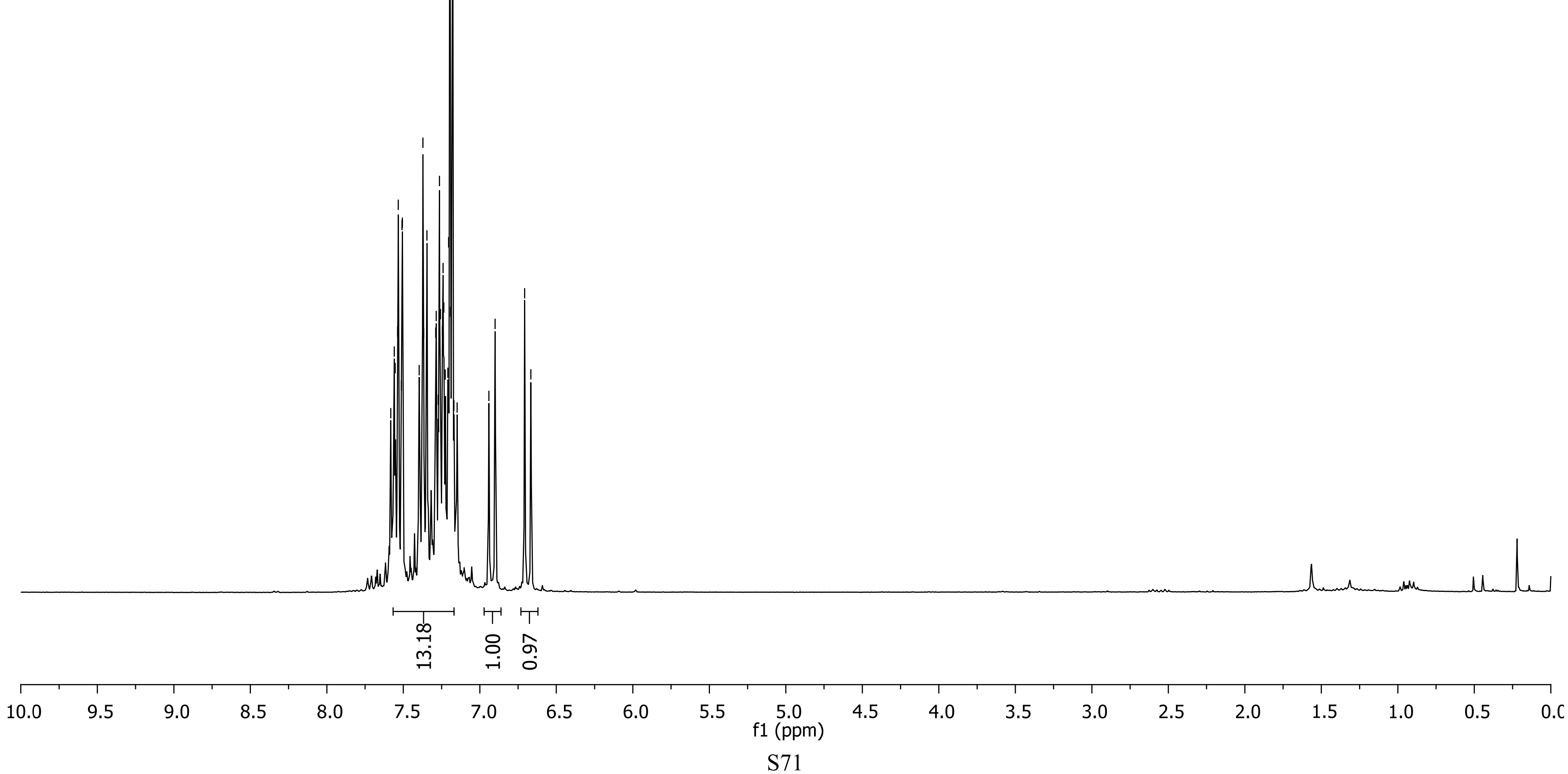

^{13}C NMR (75.4 MHz, $CDCl_3$)

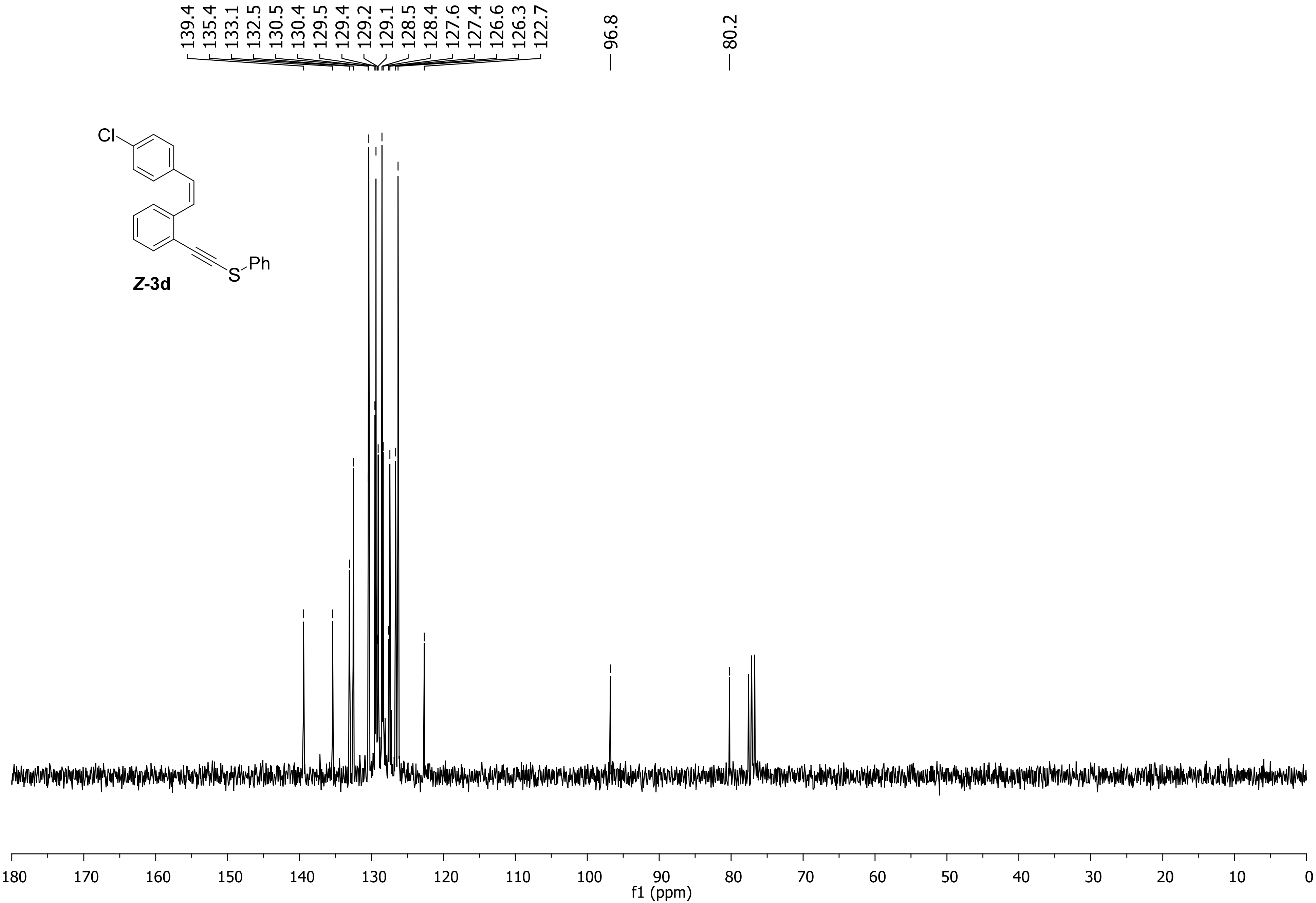

^{1}H NMR (300 MHz, $CDCl_3$)

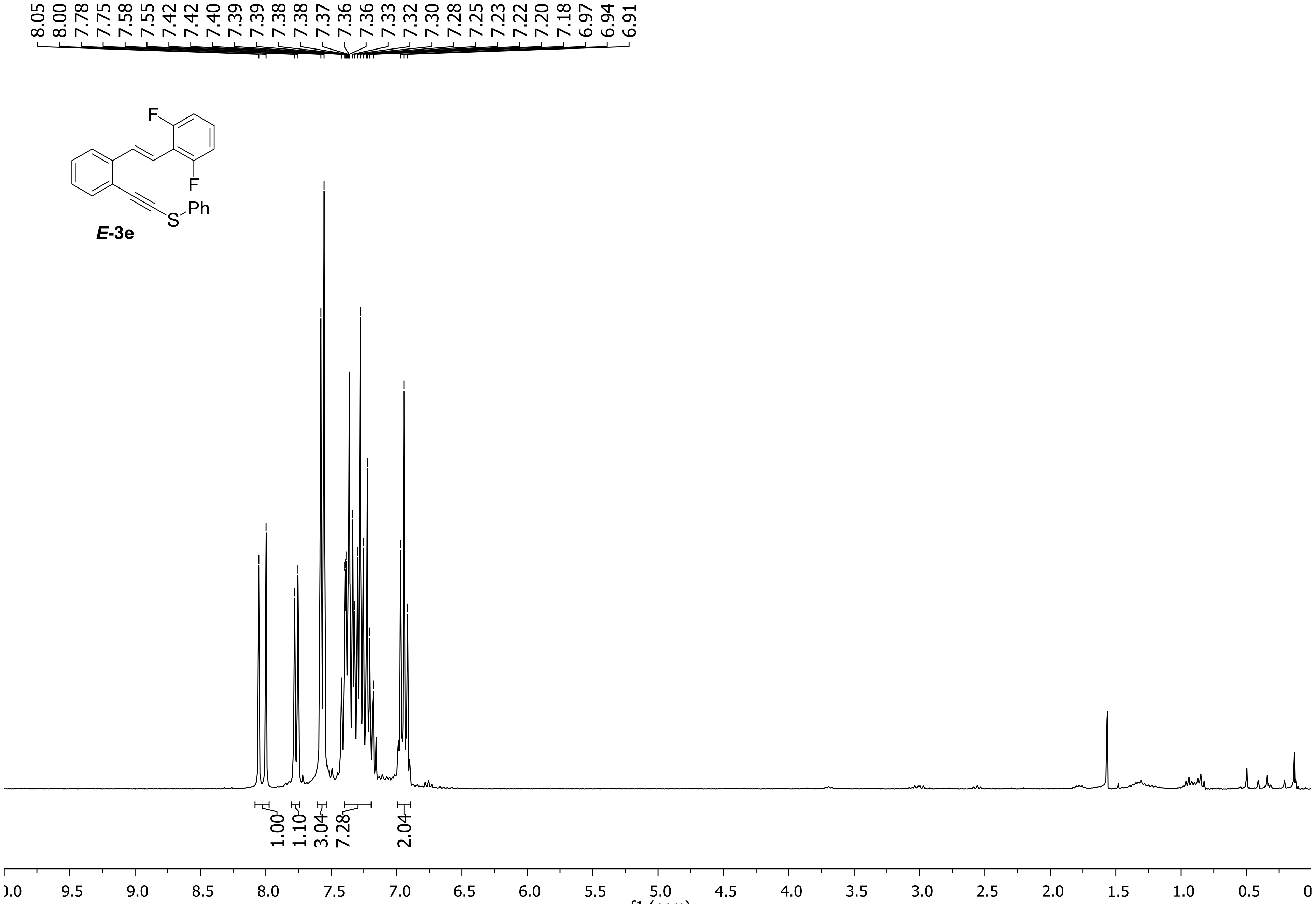

^{13}C NMR (75.4 MHz, $CDCl_3$)

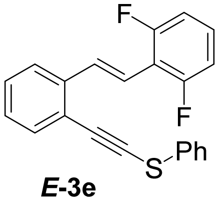

E-**3e**

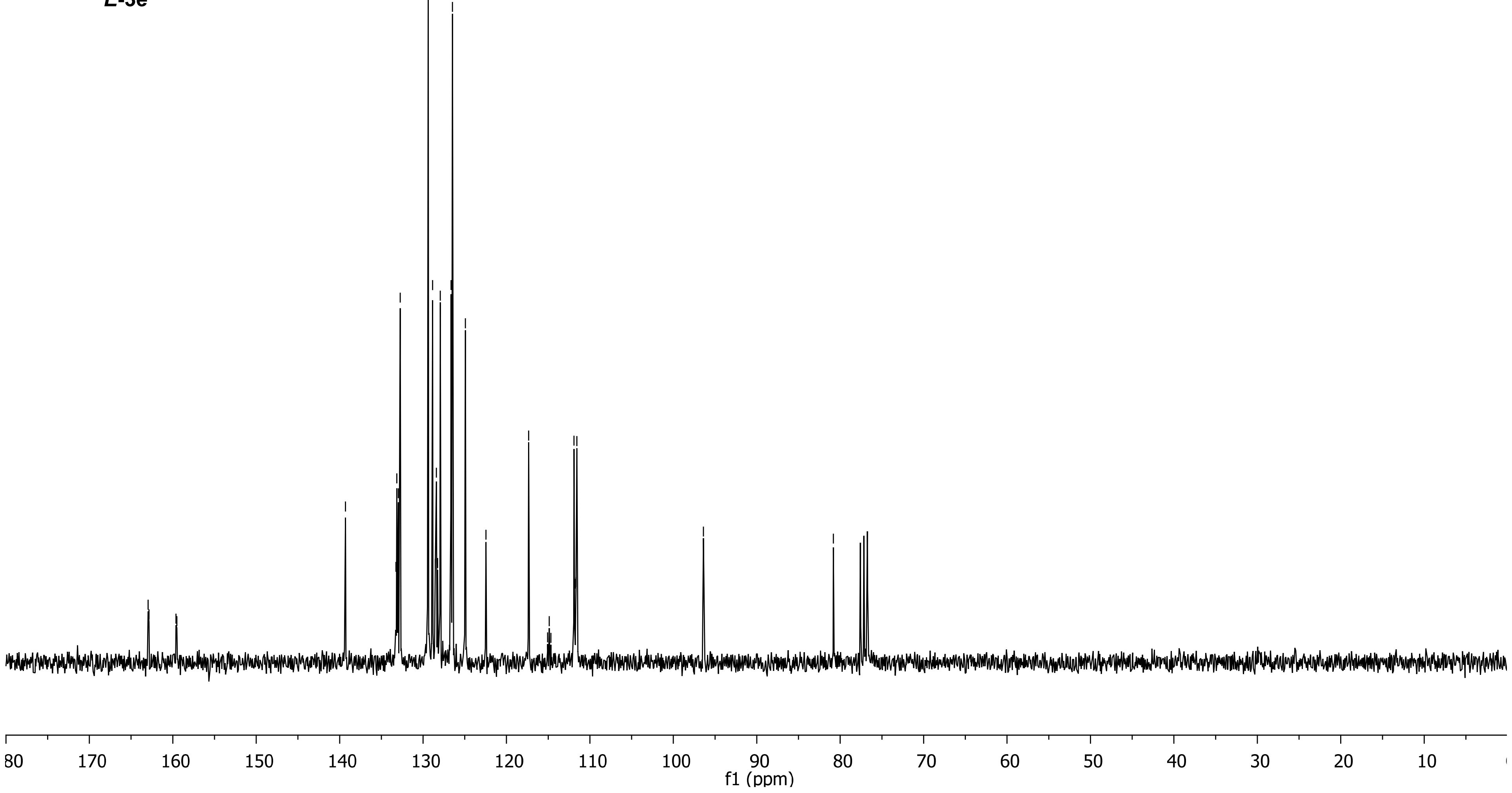

^{1}H NMR (300 MHz, $CDCl_3$)

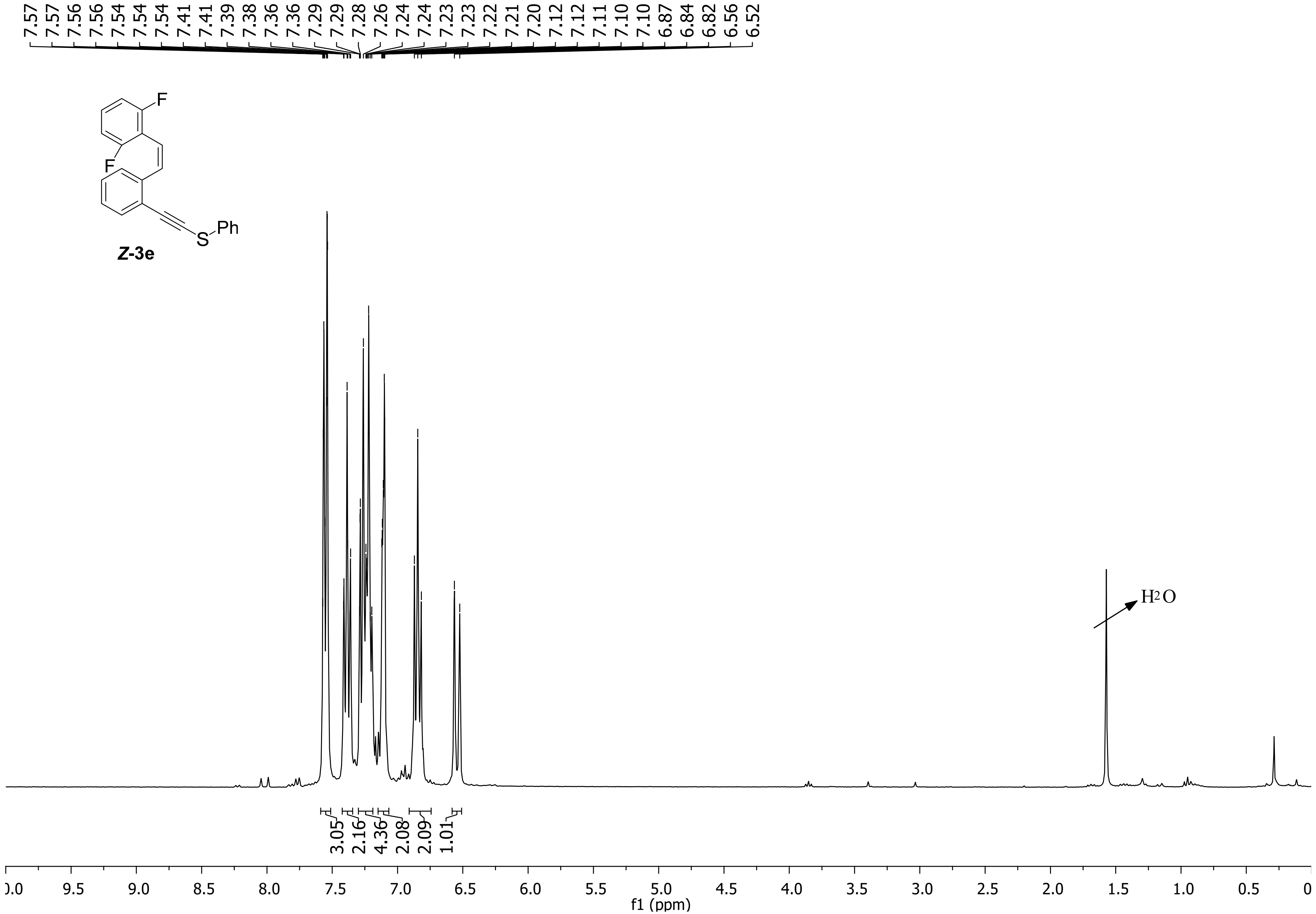

^{13}C NMR (75.4 MHz, $CDCl_3$)

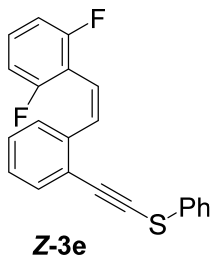

***Z*-3e**

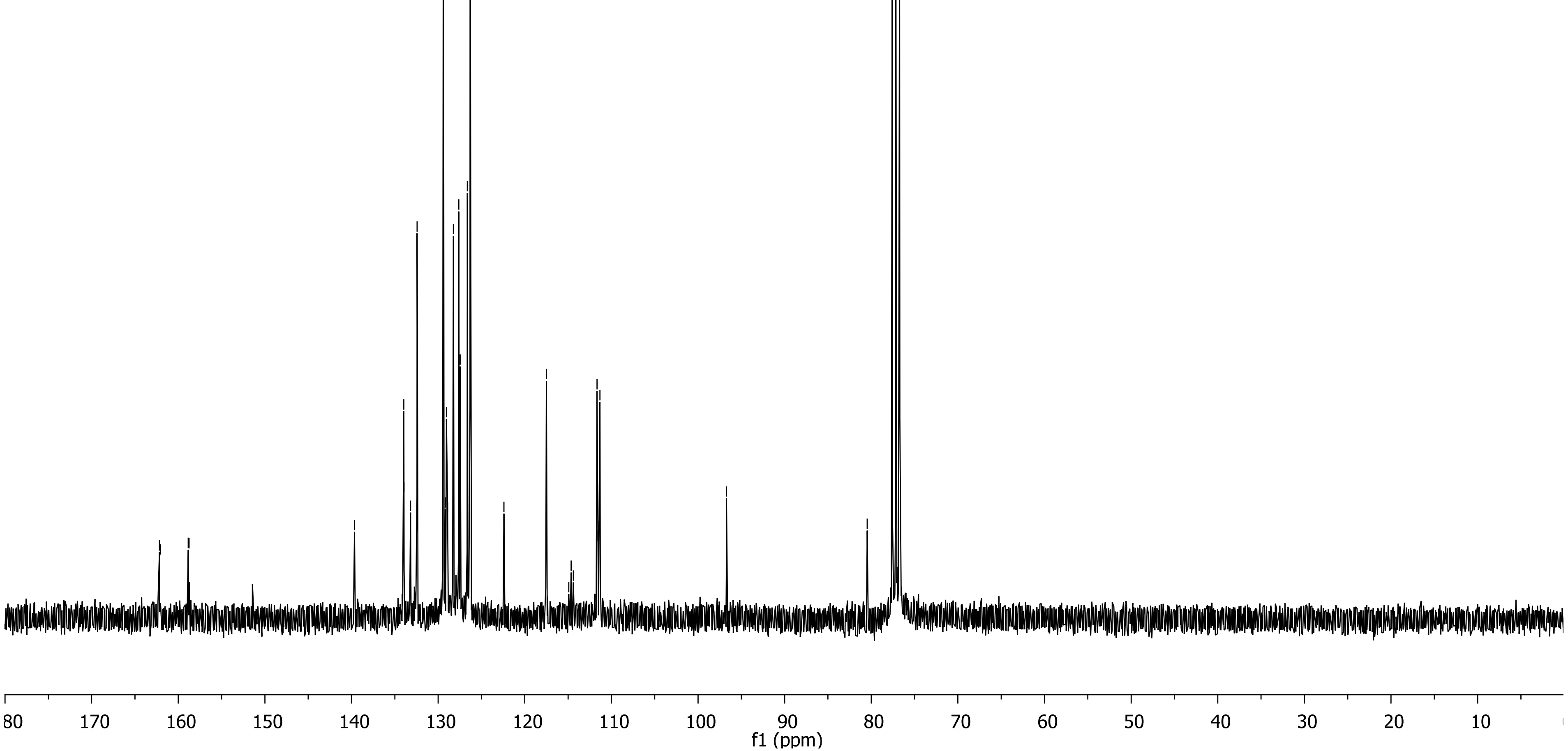

^{1}H NMR (300 MHz, $CDCl_3$)

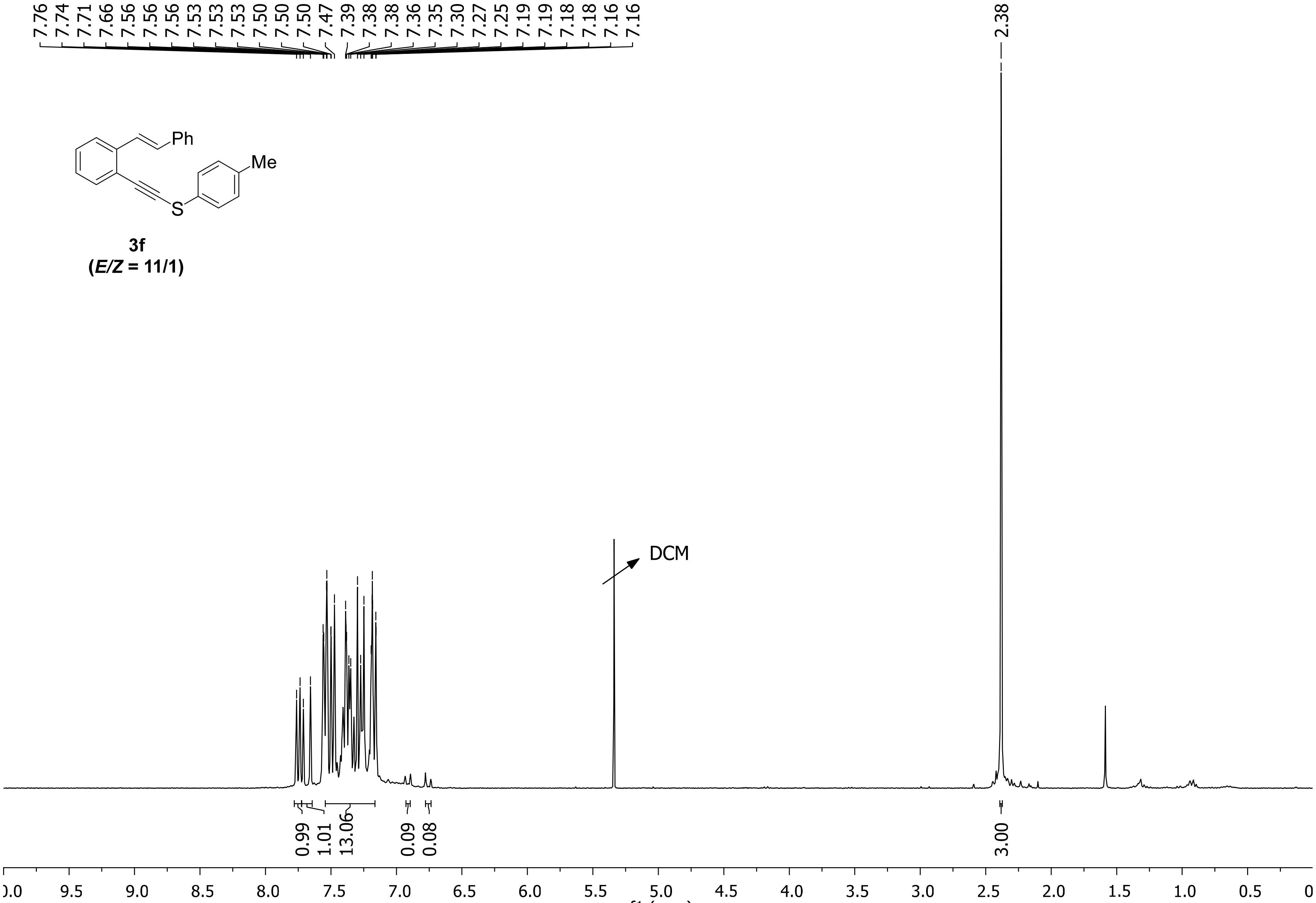

3f
(*E/Z* = 11/1)

^{13}C NMR (75.4 MHz, $CDCl_3$)

3f
(*E/Z* = 11/1)

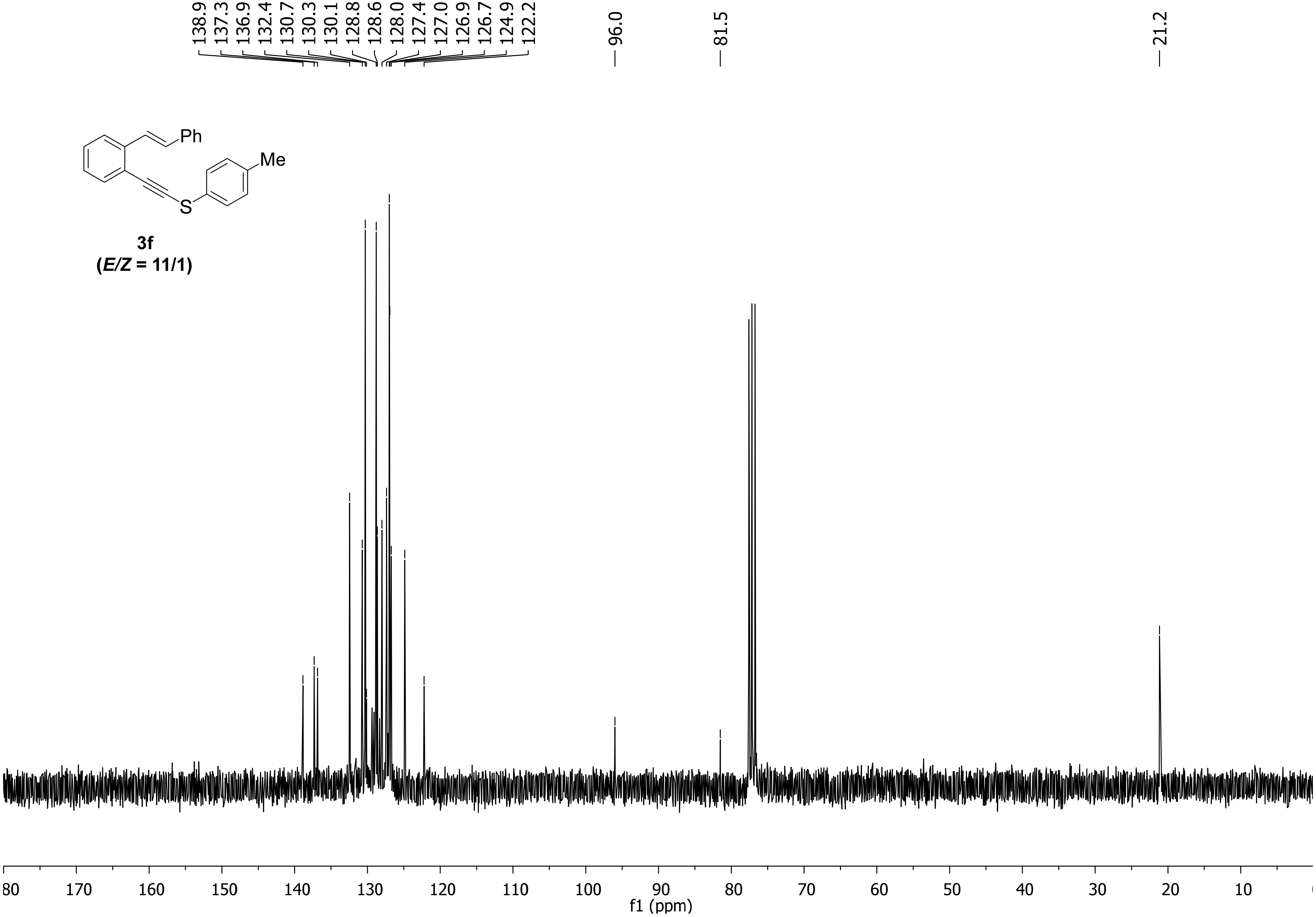

^{1}H NMR (300 MHz, $CDCl_3$)

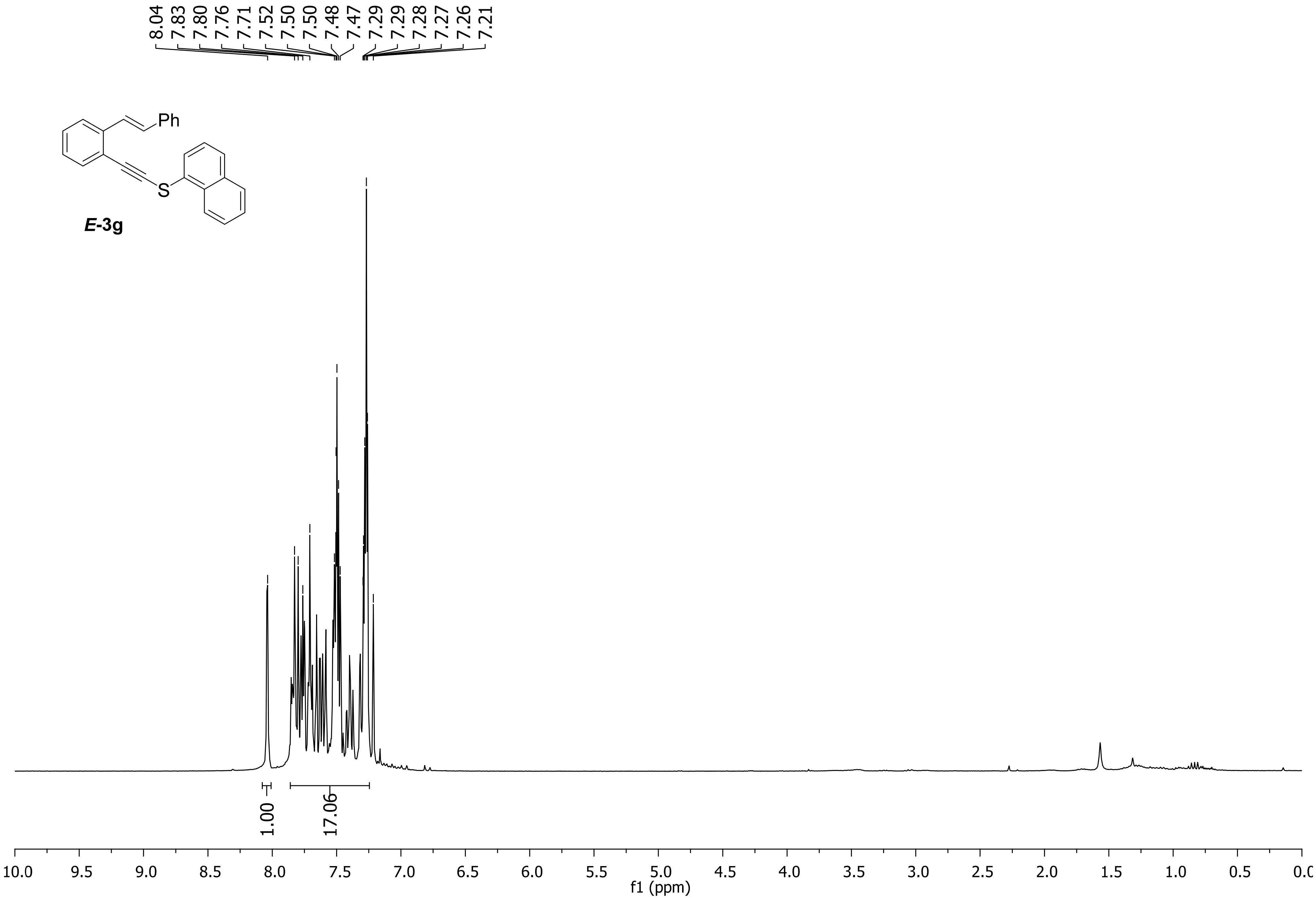

***E*-3g**

^{13}C NMR (75.4 MHz, $CDCl_3$)

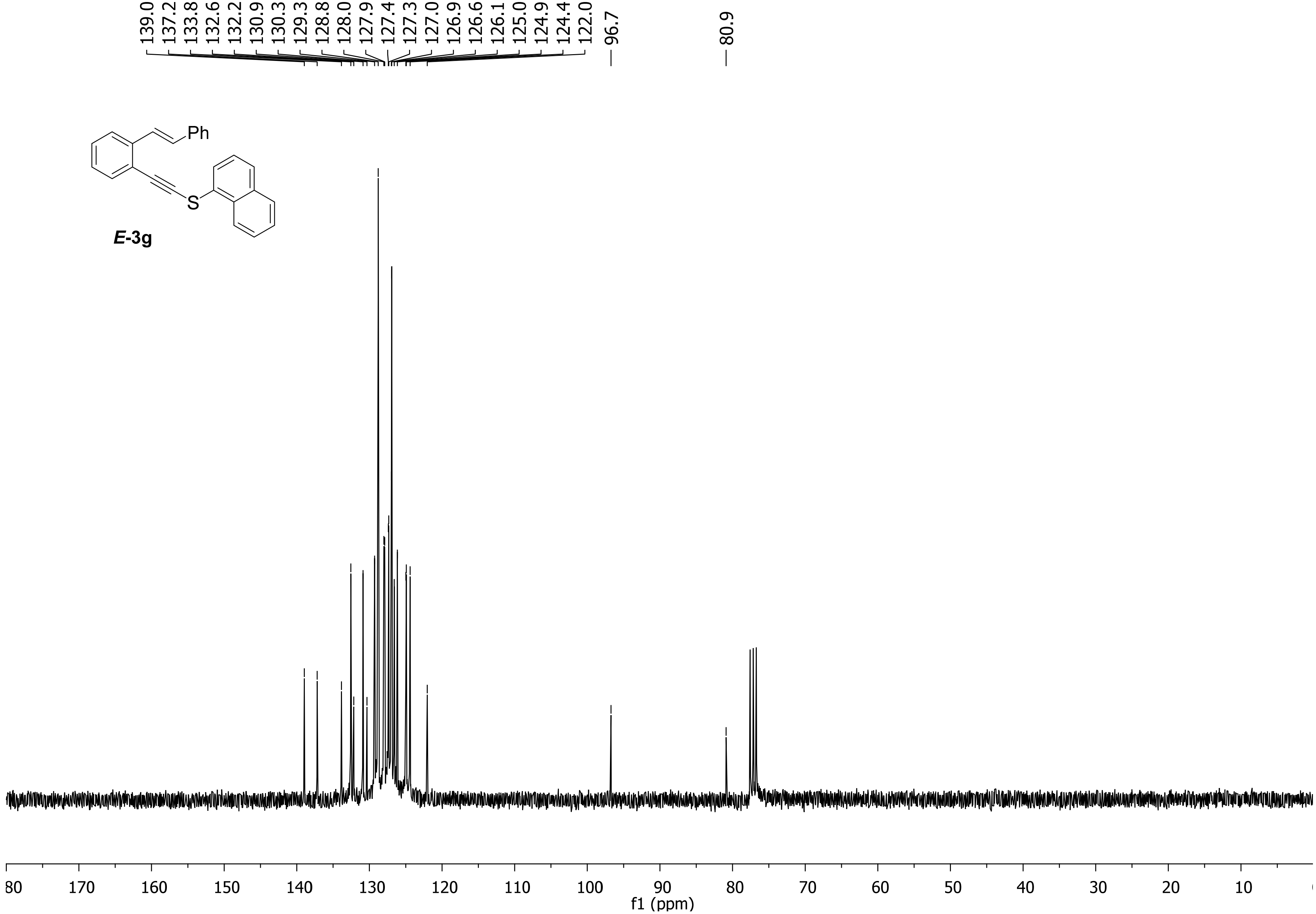

***E*-3g**

^{1}H NMR (300 MHz, $CDCl_3$)

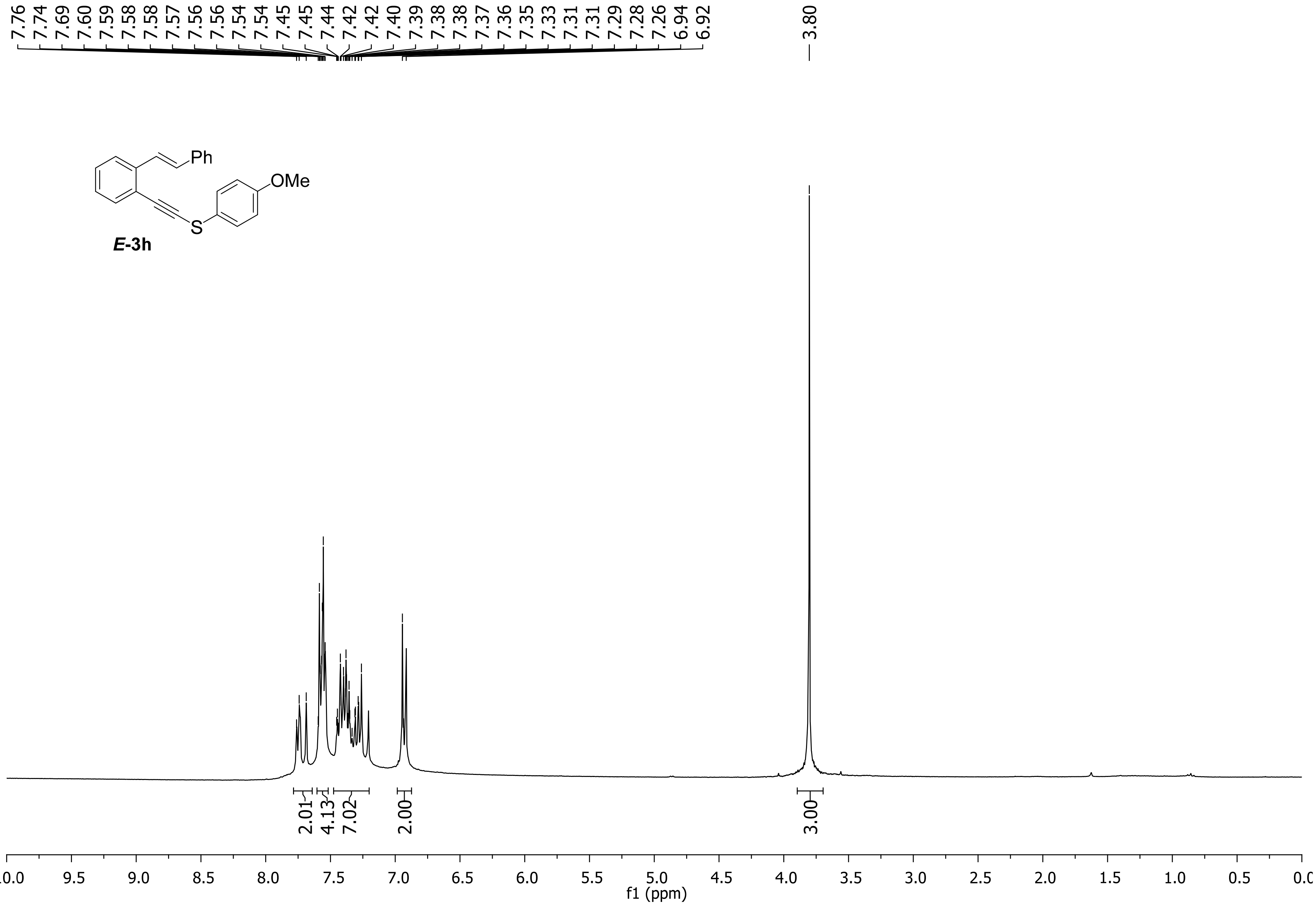

^{13}C NMR (75.4 MHz, $CDCl_3$)

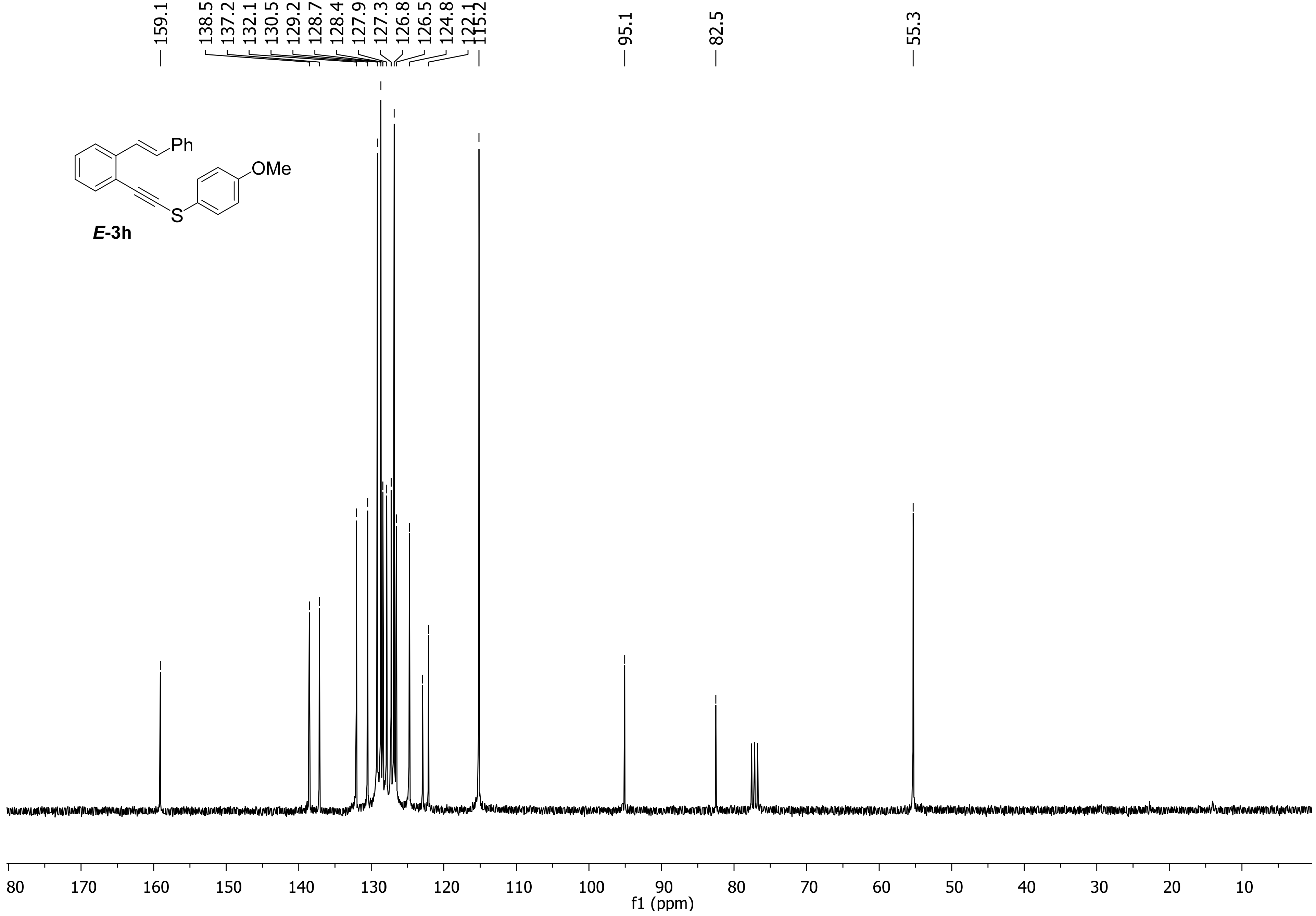

^{1}H NMR (300 MHz, $CDCl_3$)

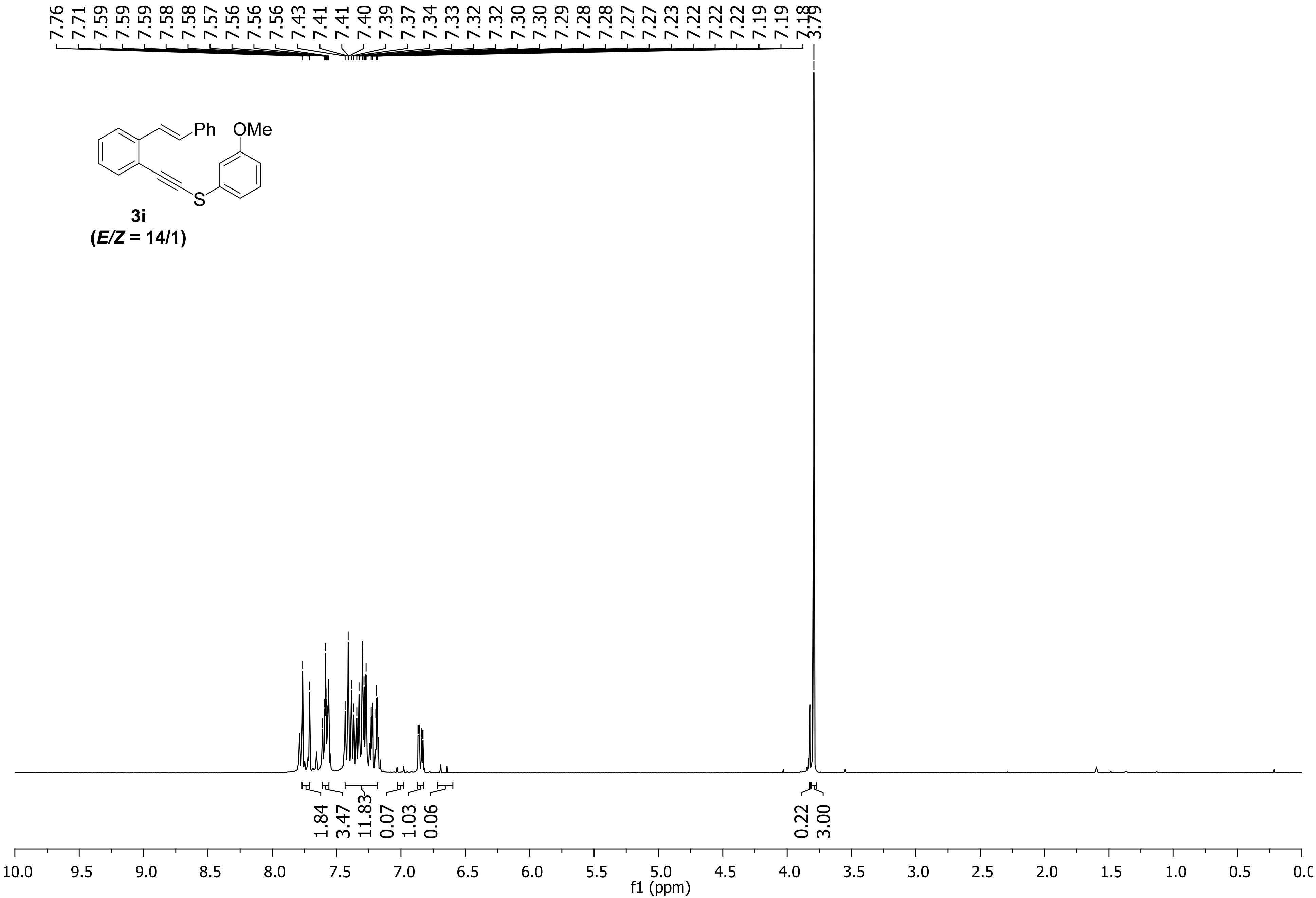

^{13}C NMR (75.4 MHz, $CDCl_3$)

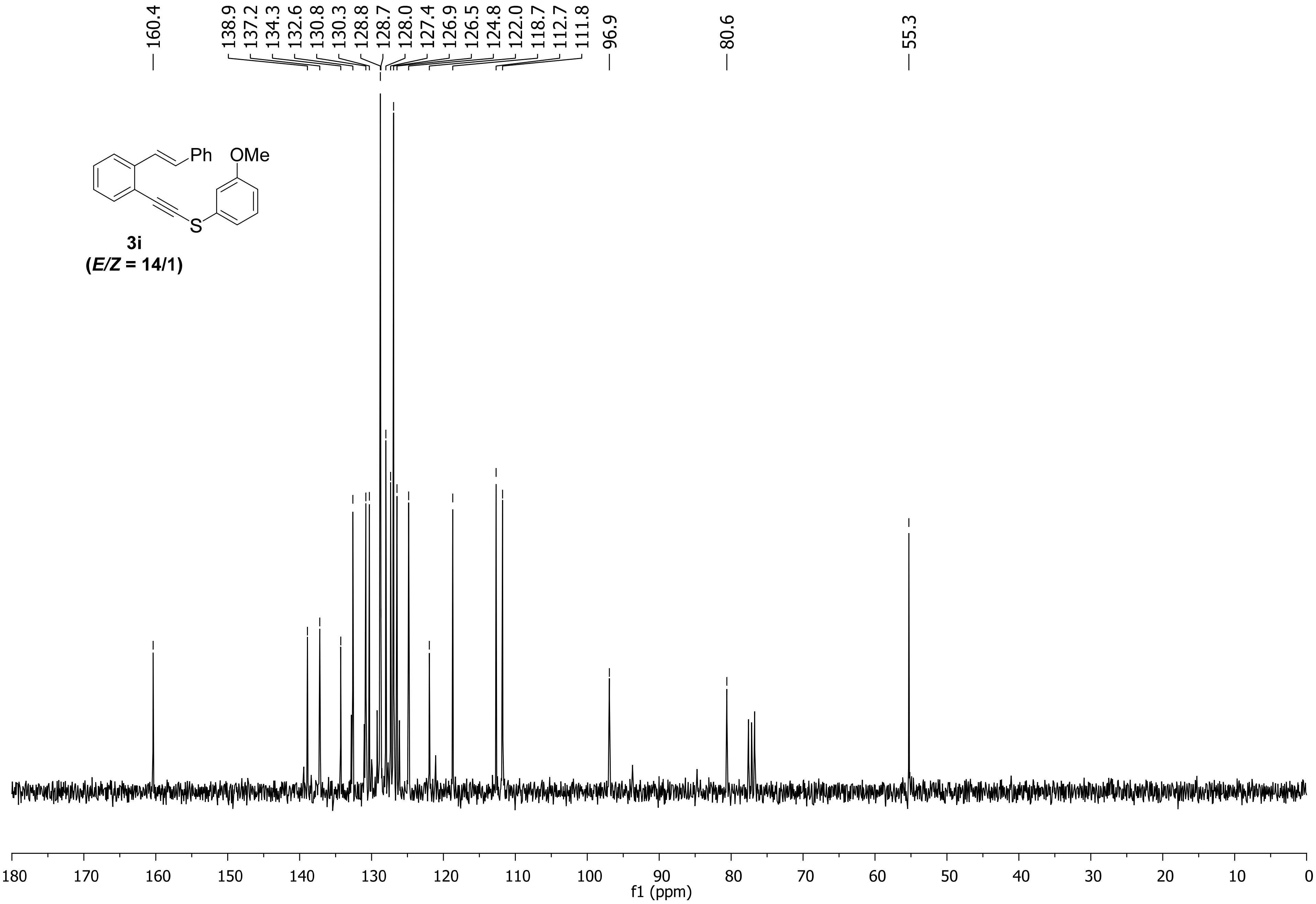

^{1}H NMR (300 MHz, $CDCl_3$)

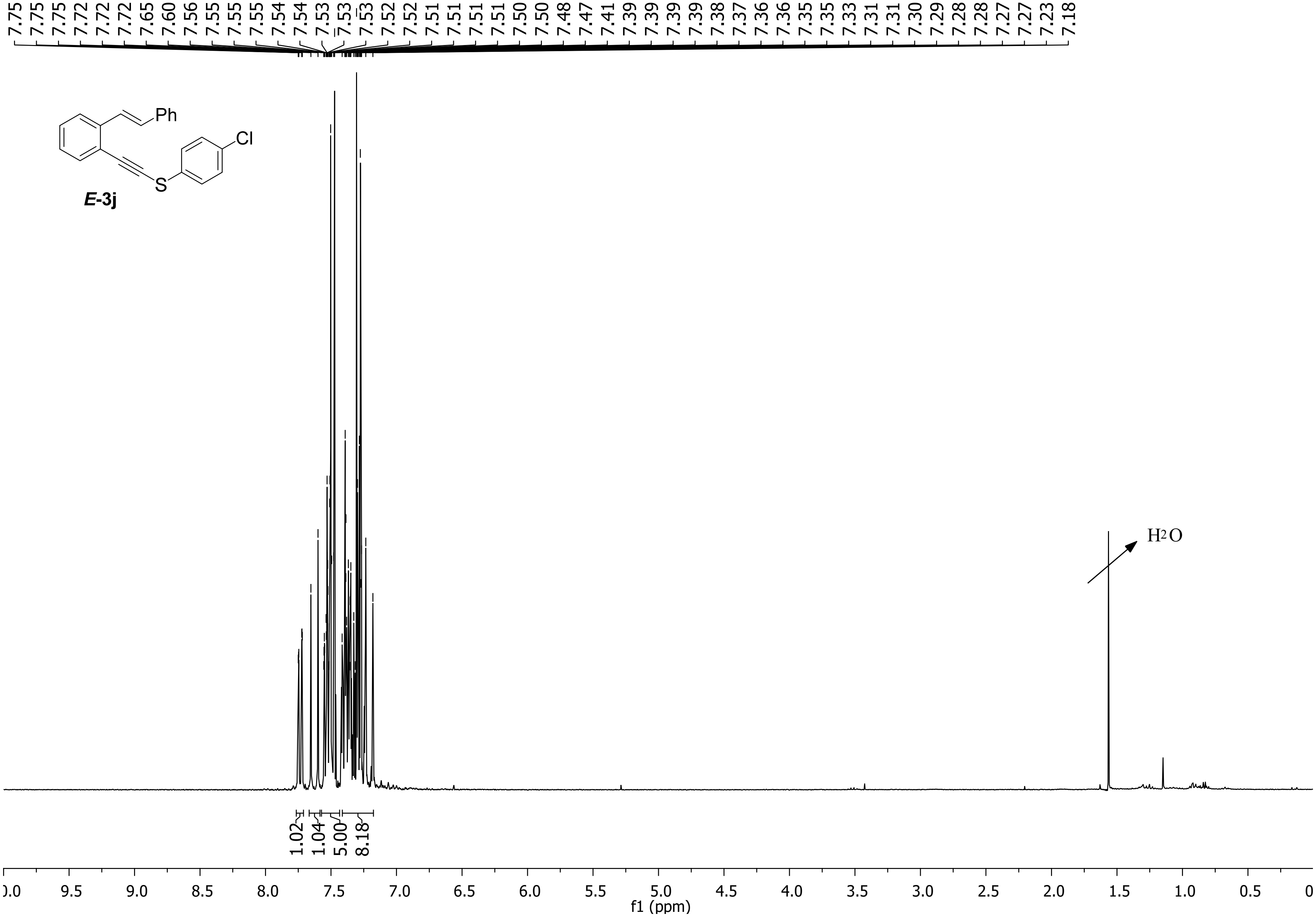

^{13}C NMR (75.4 MHz, $CDCl_3$)

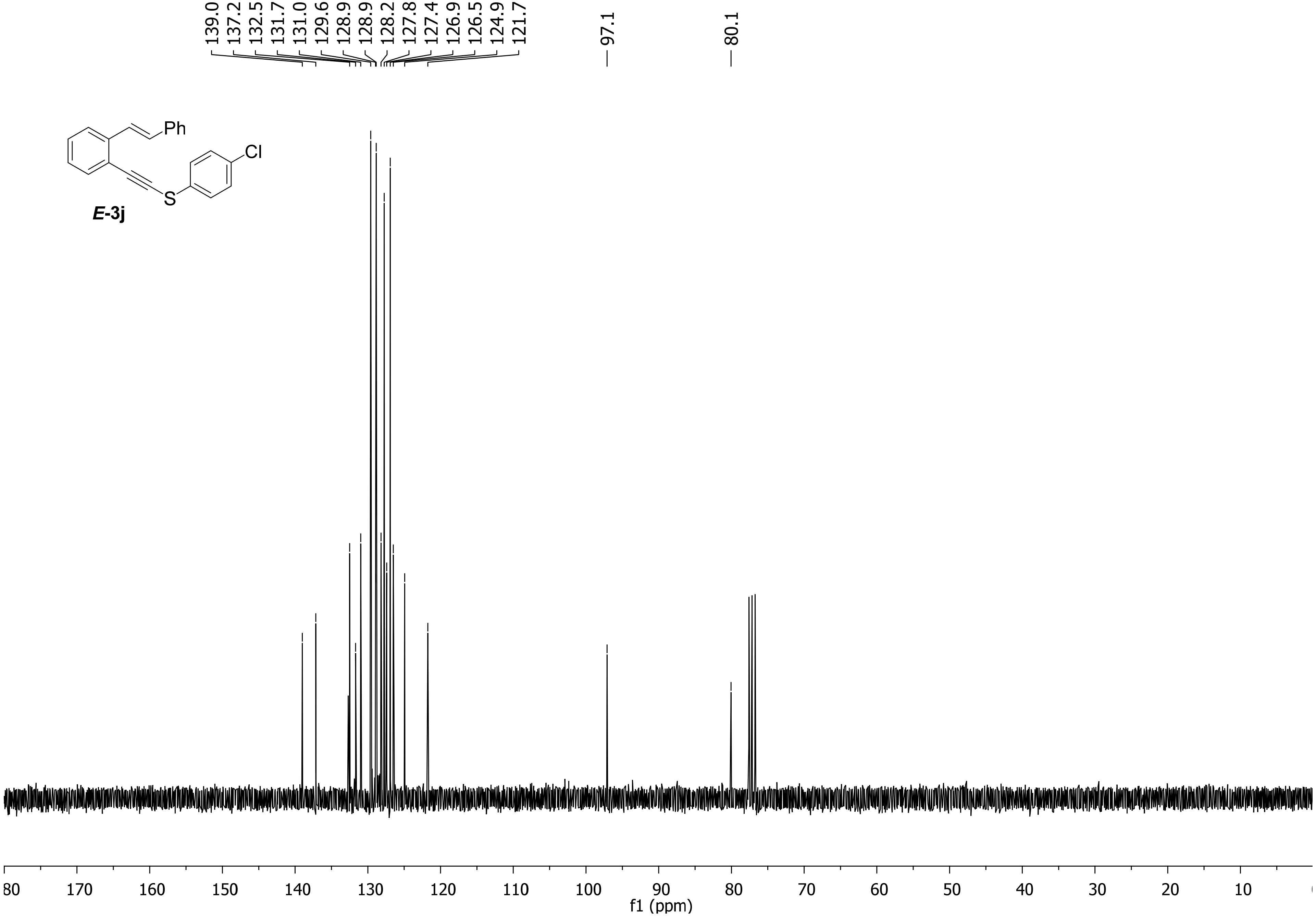

^{1}H NMR (300 MHz, $CDCl_3$)

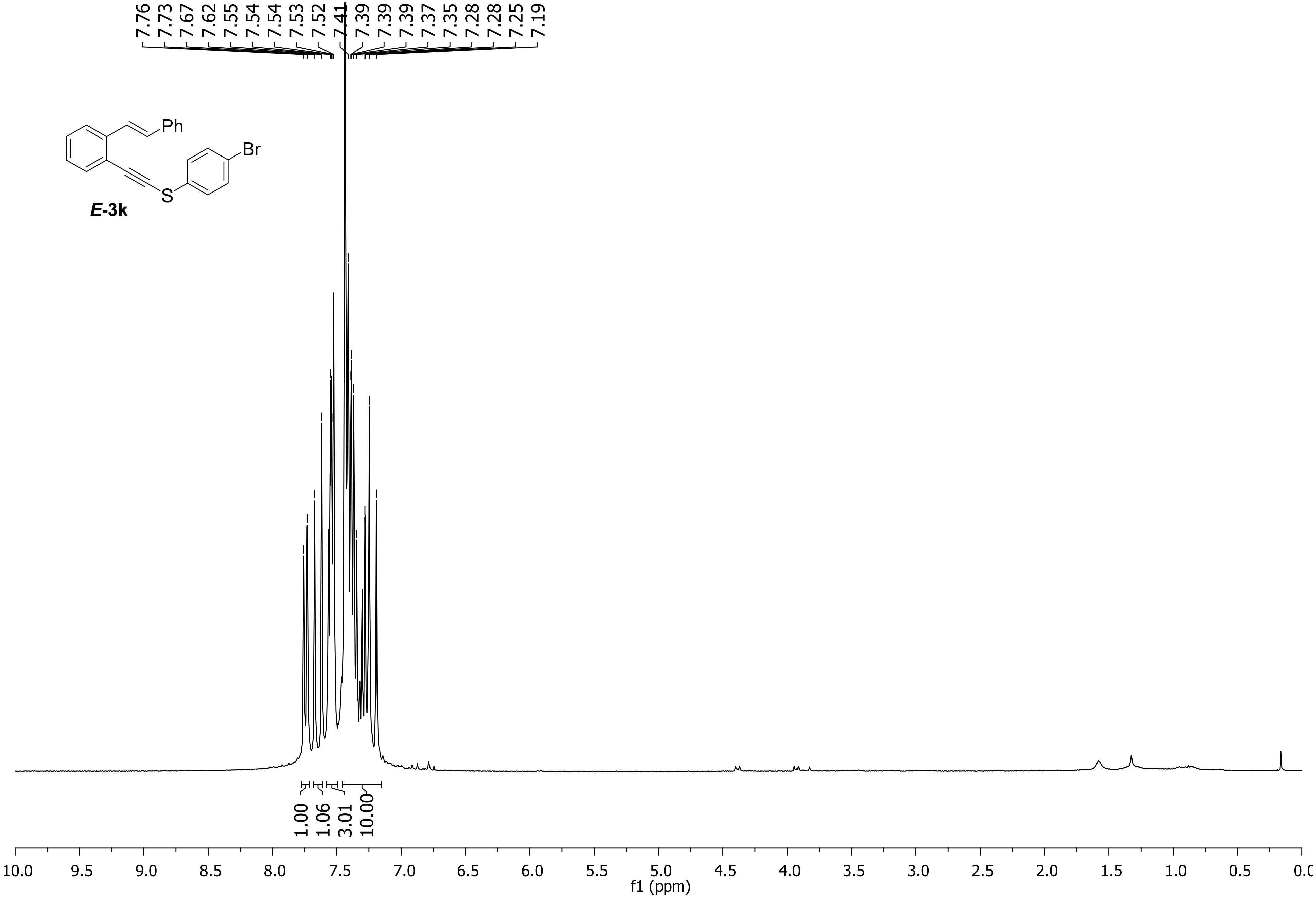

^{13}C NMR (75.4 MHz, $CDCl_3$)

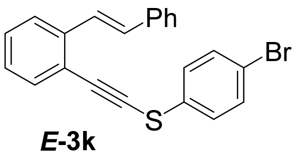

***E*-3k**

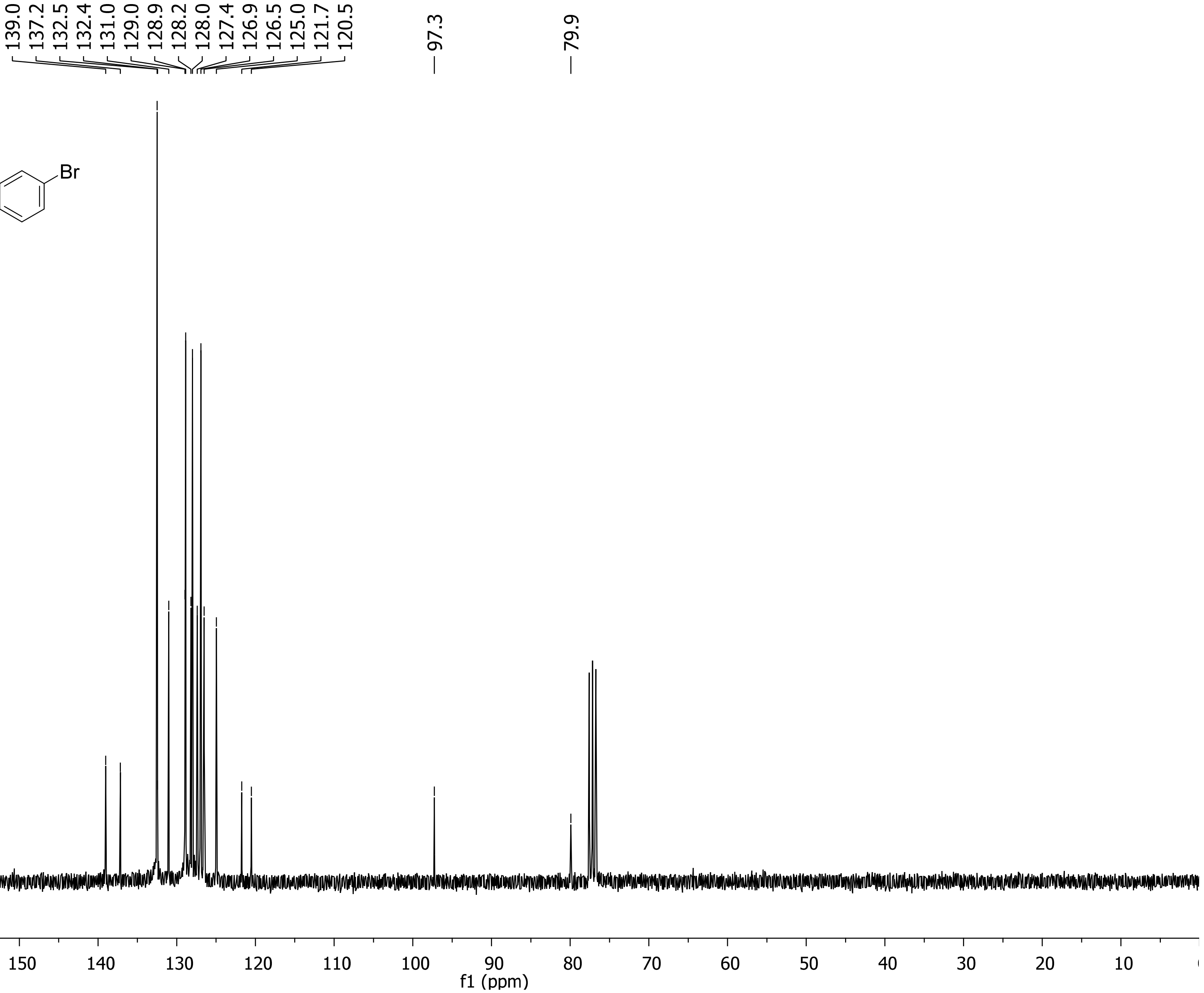

^{1}H NMR (300 MHz, $CDCl_3$)

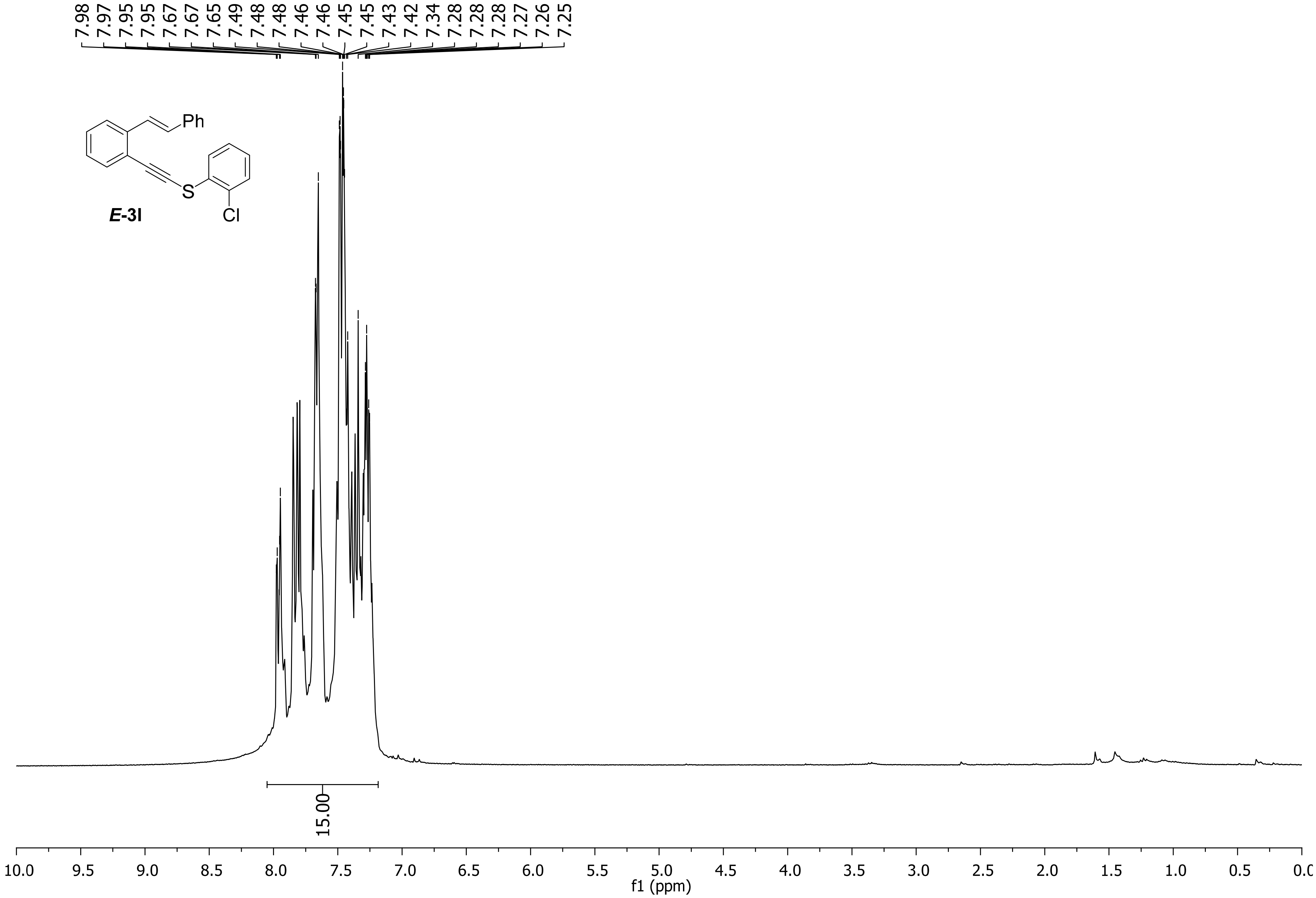

^{13}C NMR (75.4 MHz, $CDCl_3$)

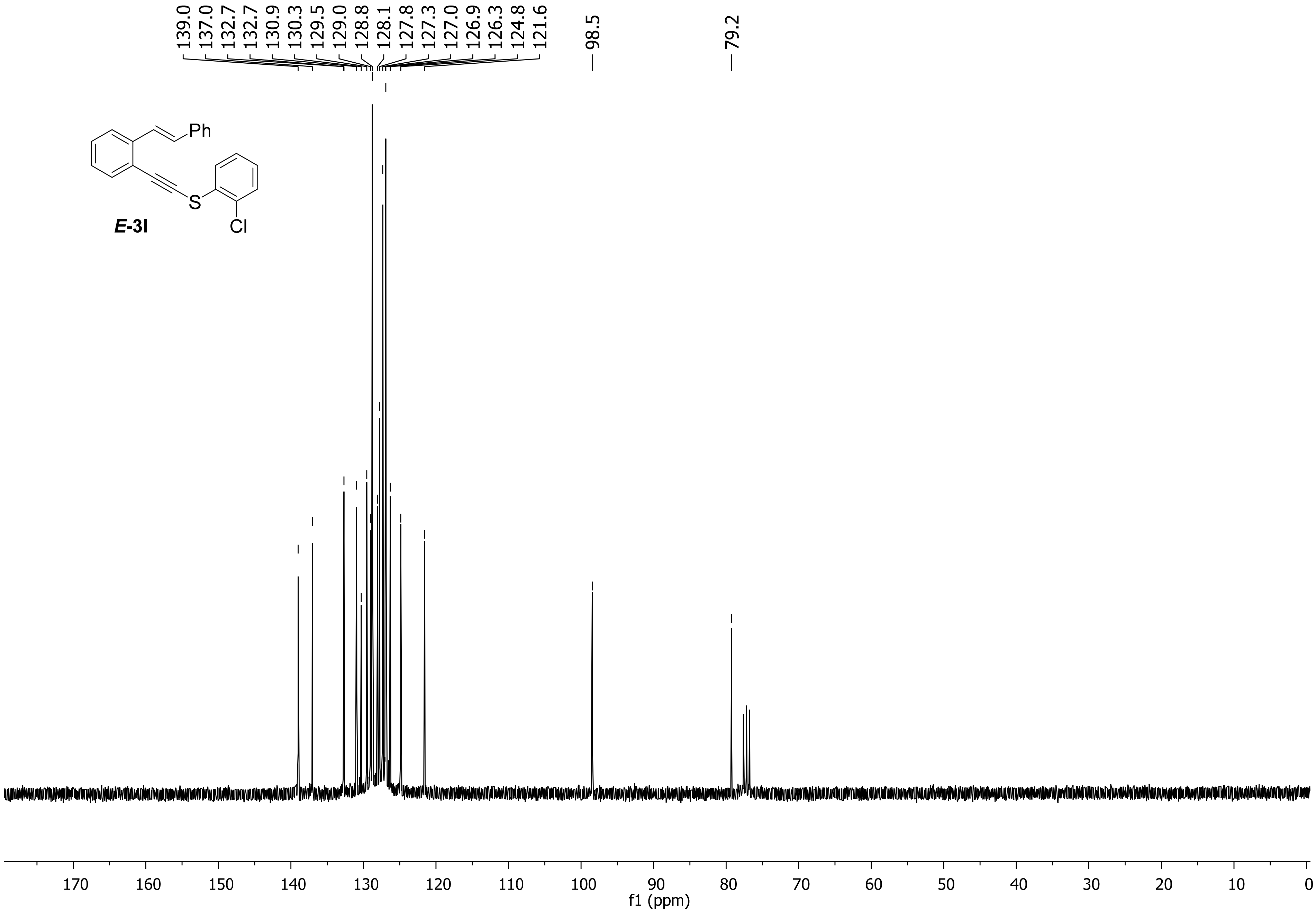

^{1}H NMR (300 MHz, $CDCl_3$)

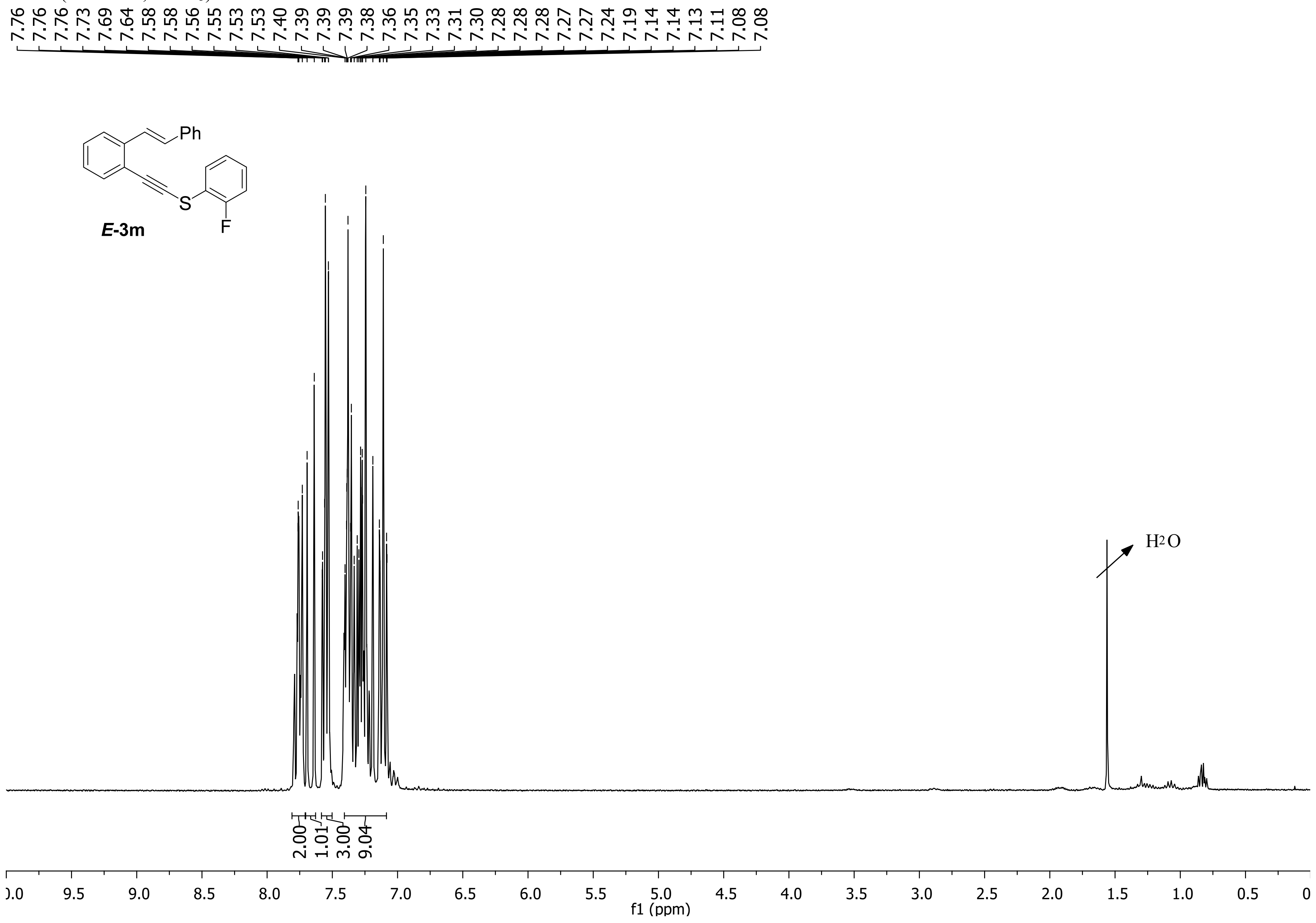

^{13}C NMR (75.4 MHz, $CDCl_3$)

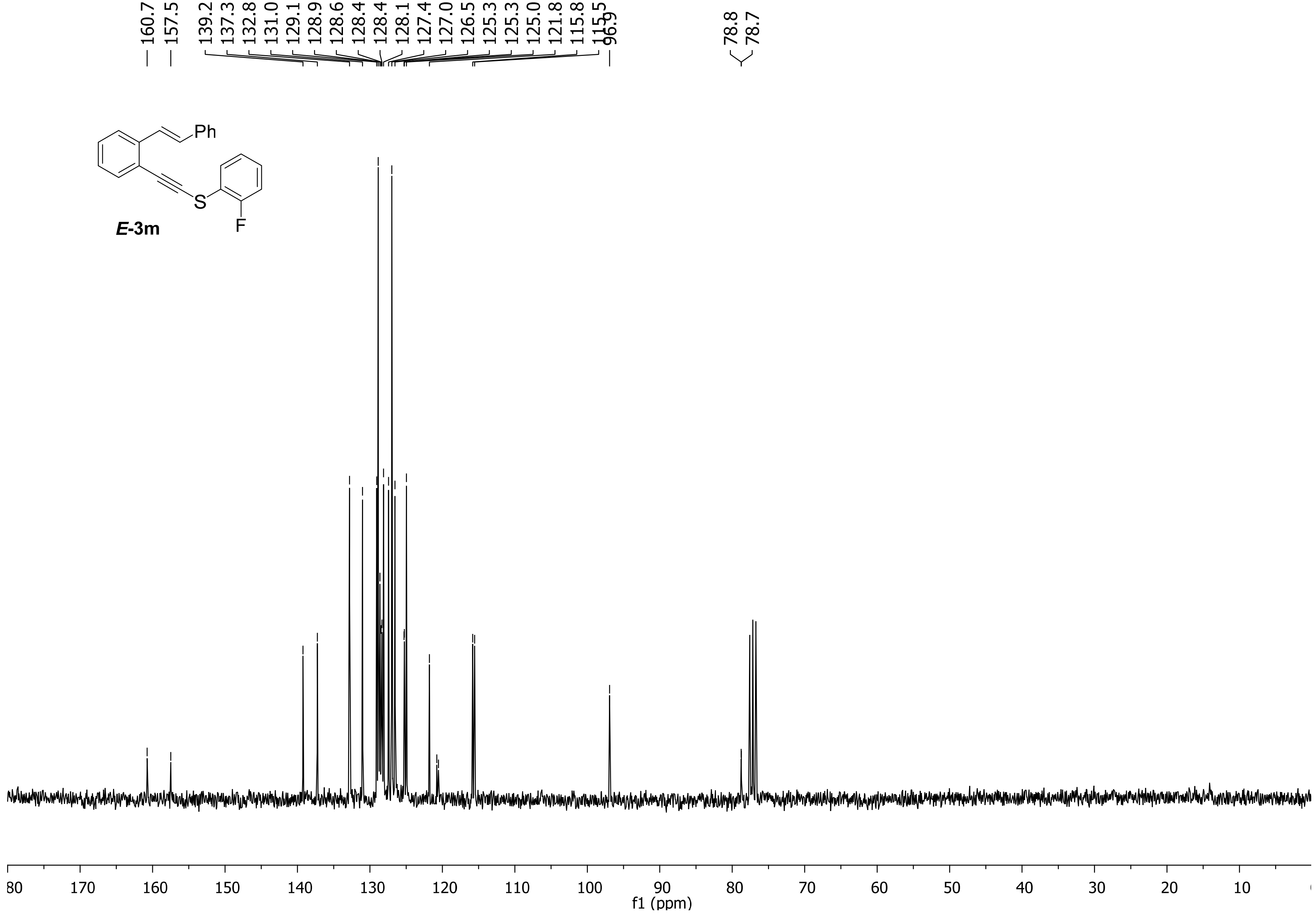

^{1}H NMR (300 MHz, $CDCl_3$)

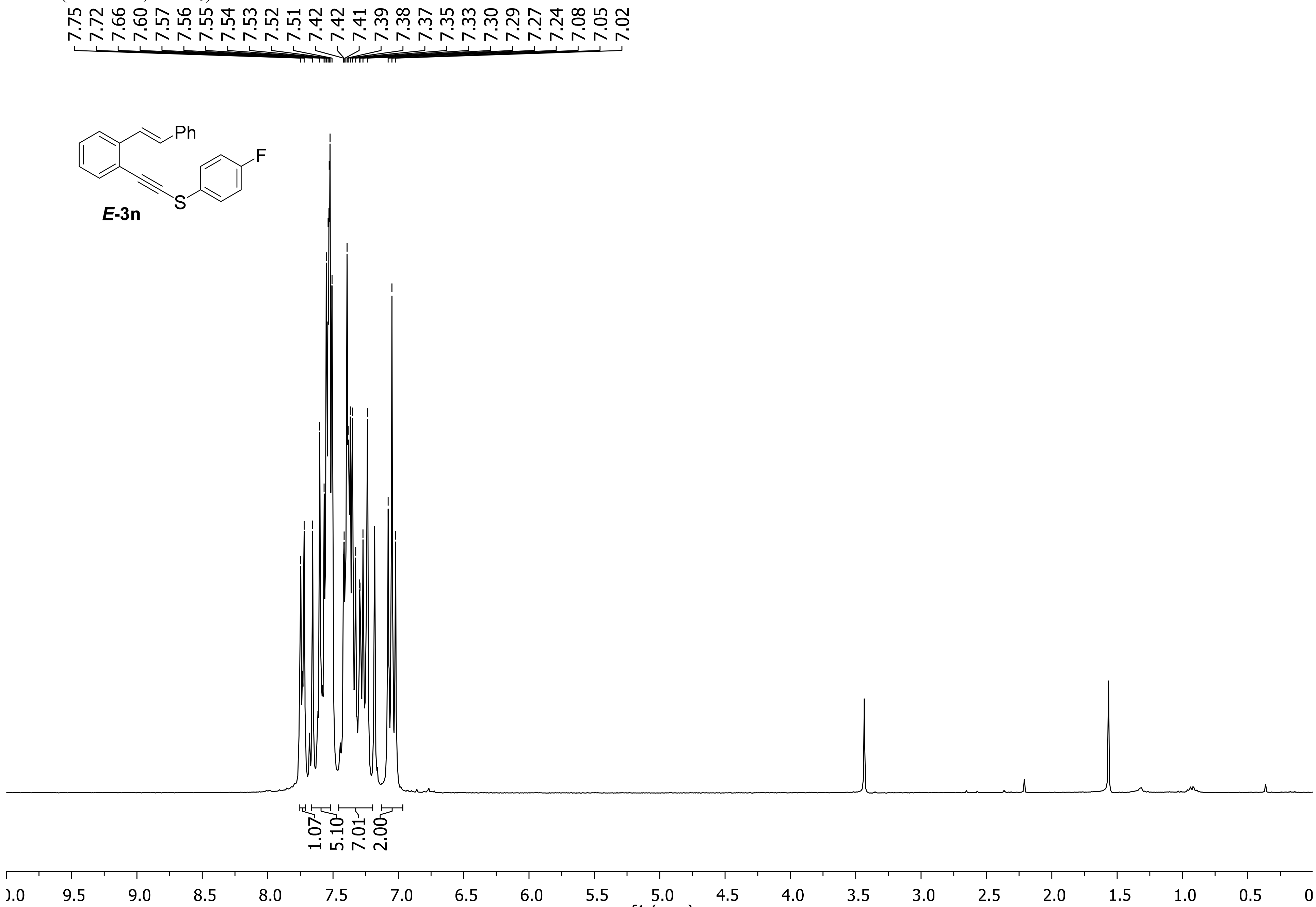

^{13}C NMR (75.4 MHz, $CDCl_3$)

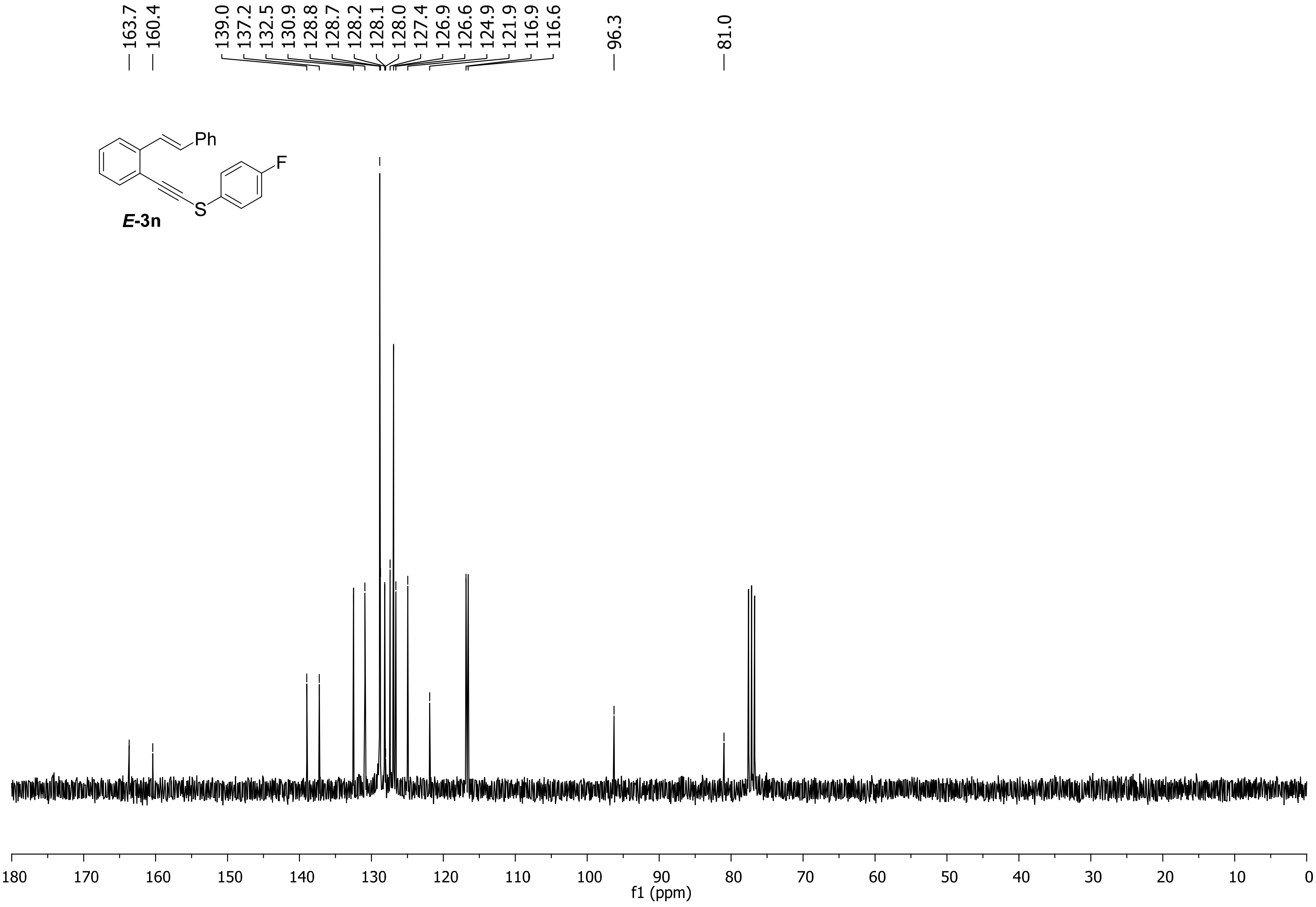

***E*-3n**

^{1}H NMR (300 MHz, $CDCl_3$)

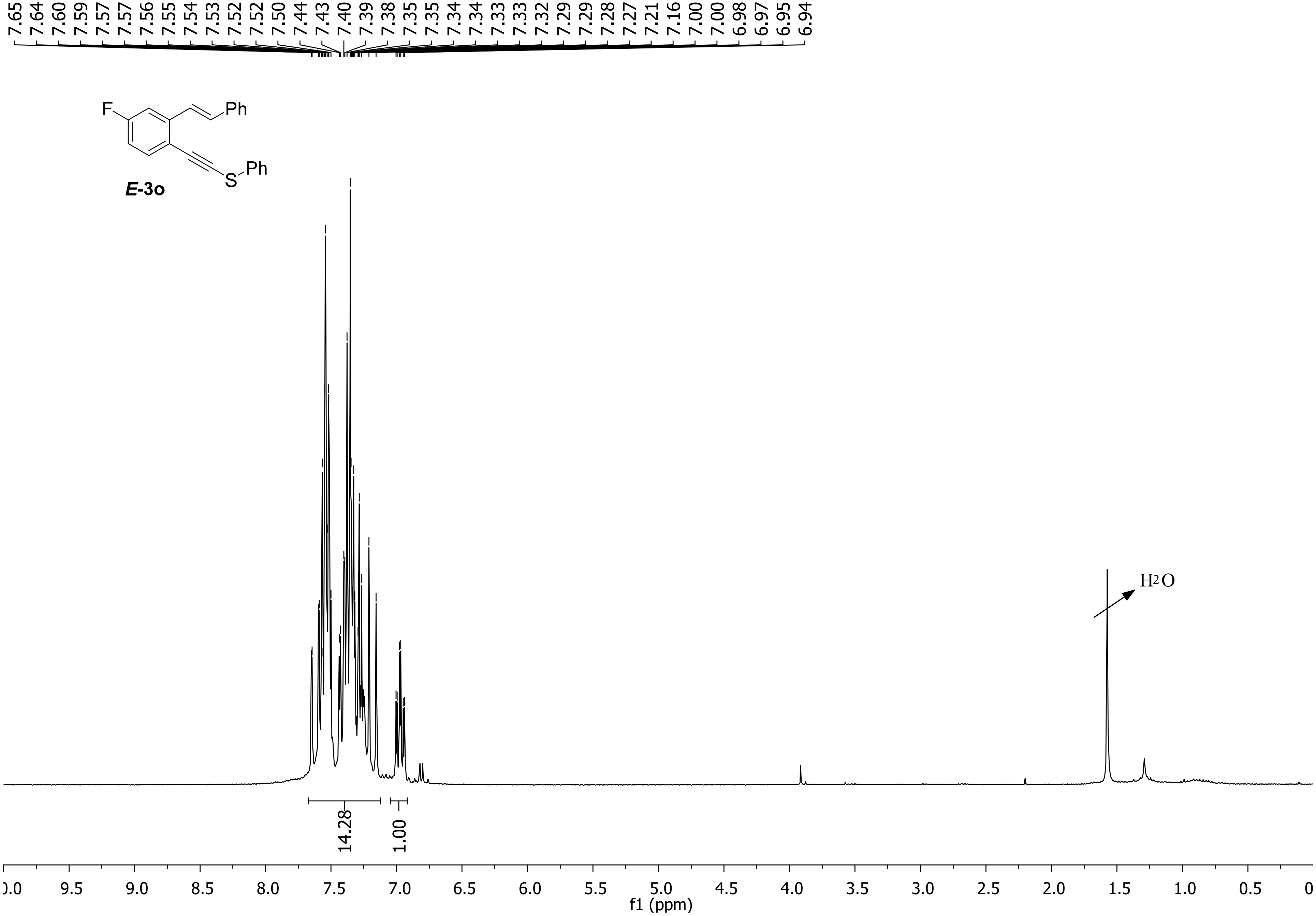

^{13}C NMR (75.4 MHz, $CDCl_3$)

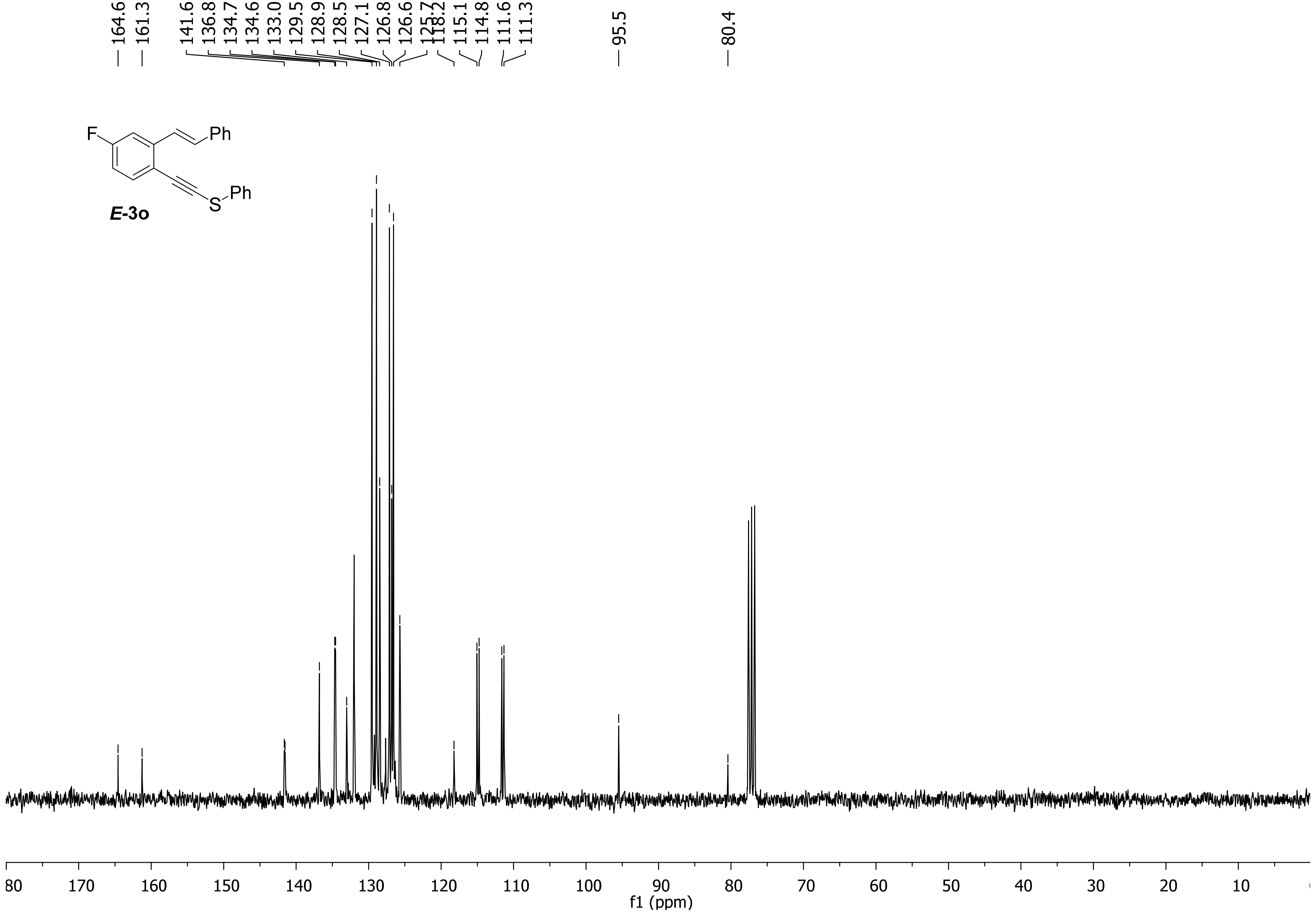

^{1}H NMR (300 MHz, $CDCl_3$)

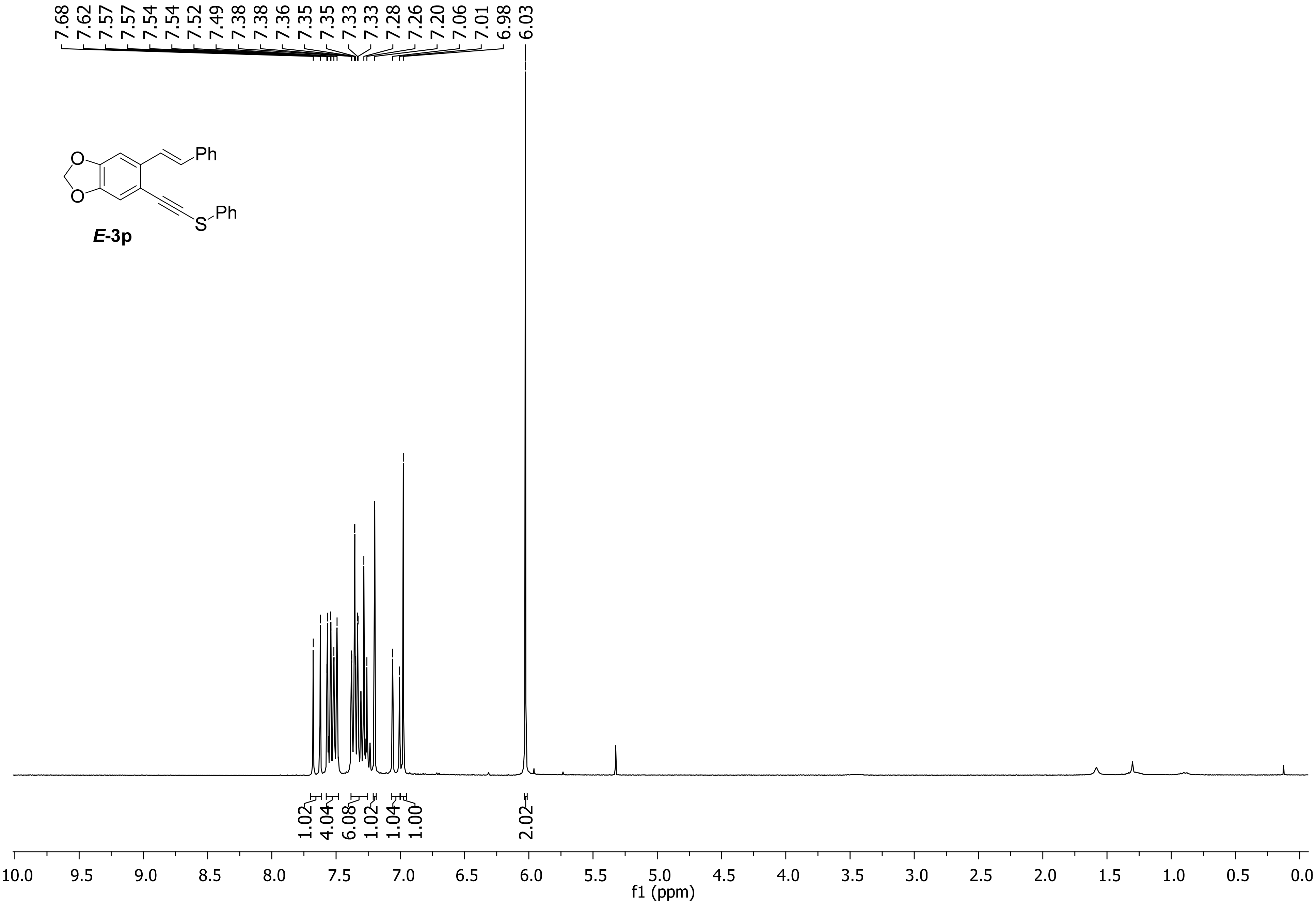

^{13}C NMR (75.4 MHz, $CDCl_3$)

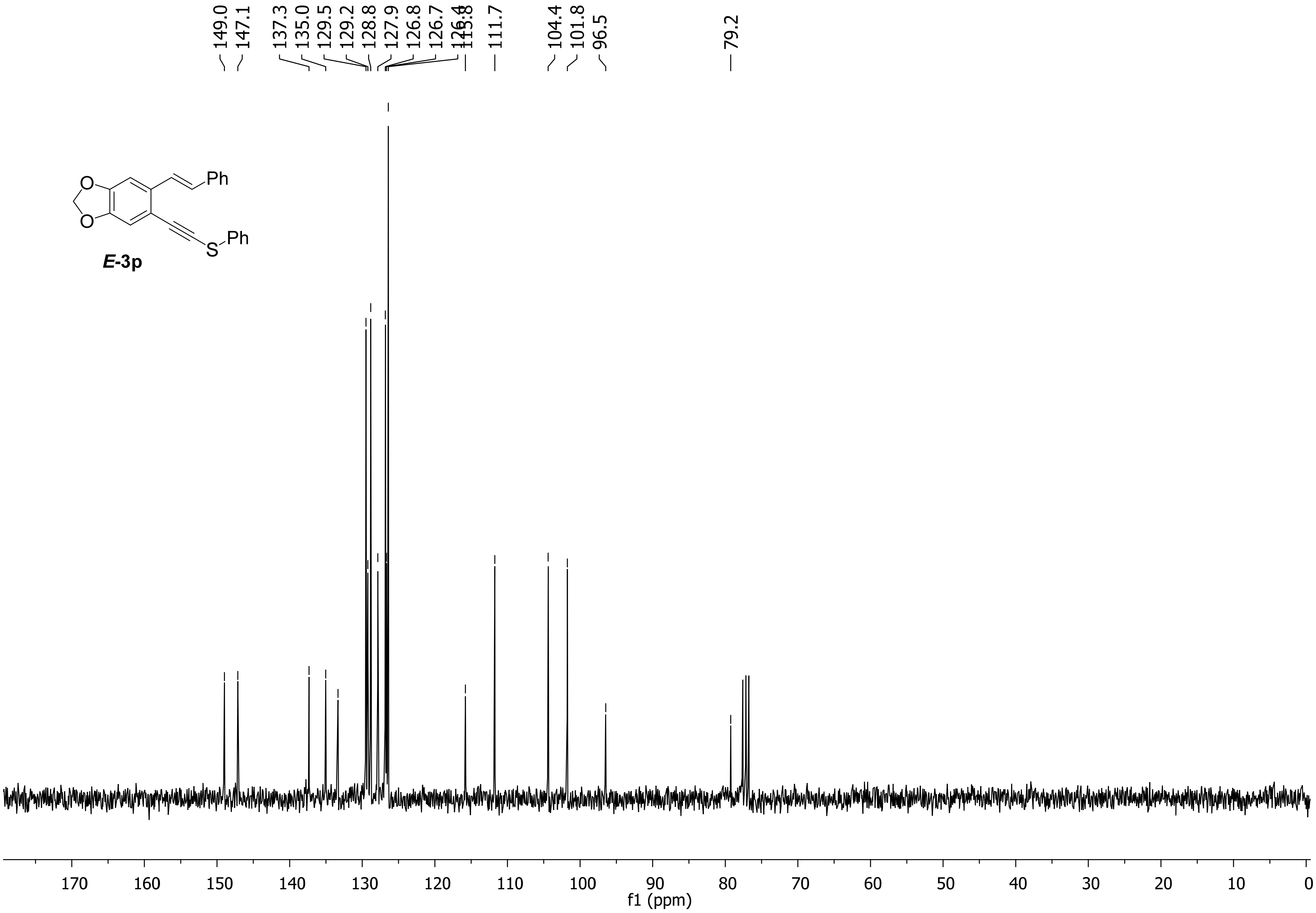

^{1}H NMR (300 MHz, $CDCl_3$)

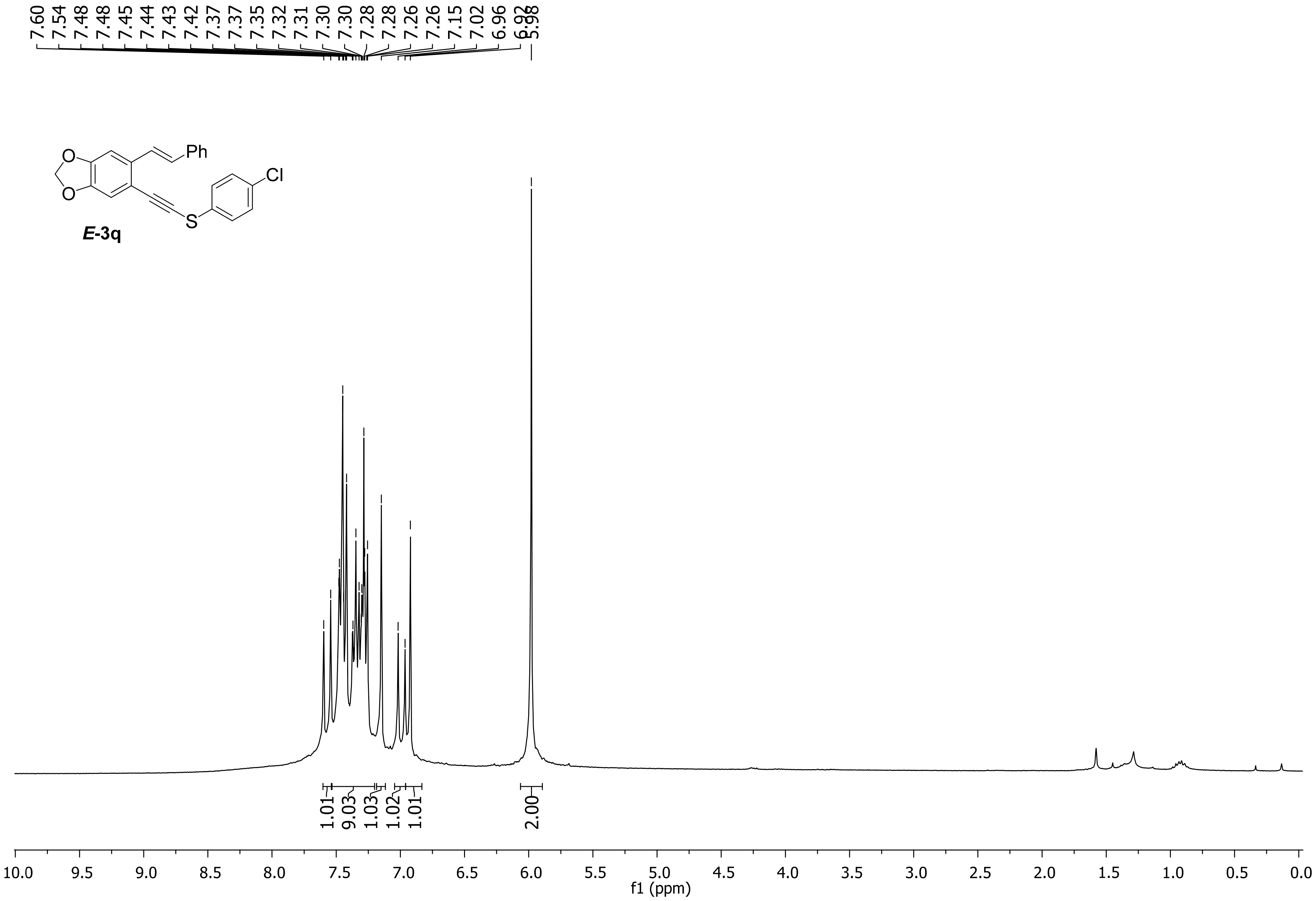

^{13}C NMR (75.4 MHz, $CDCl_3$)

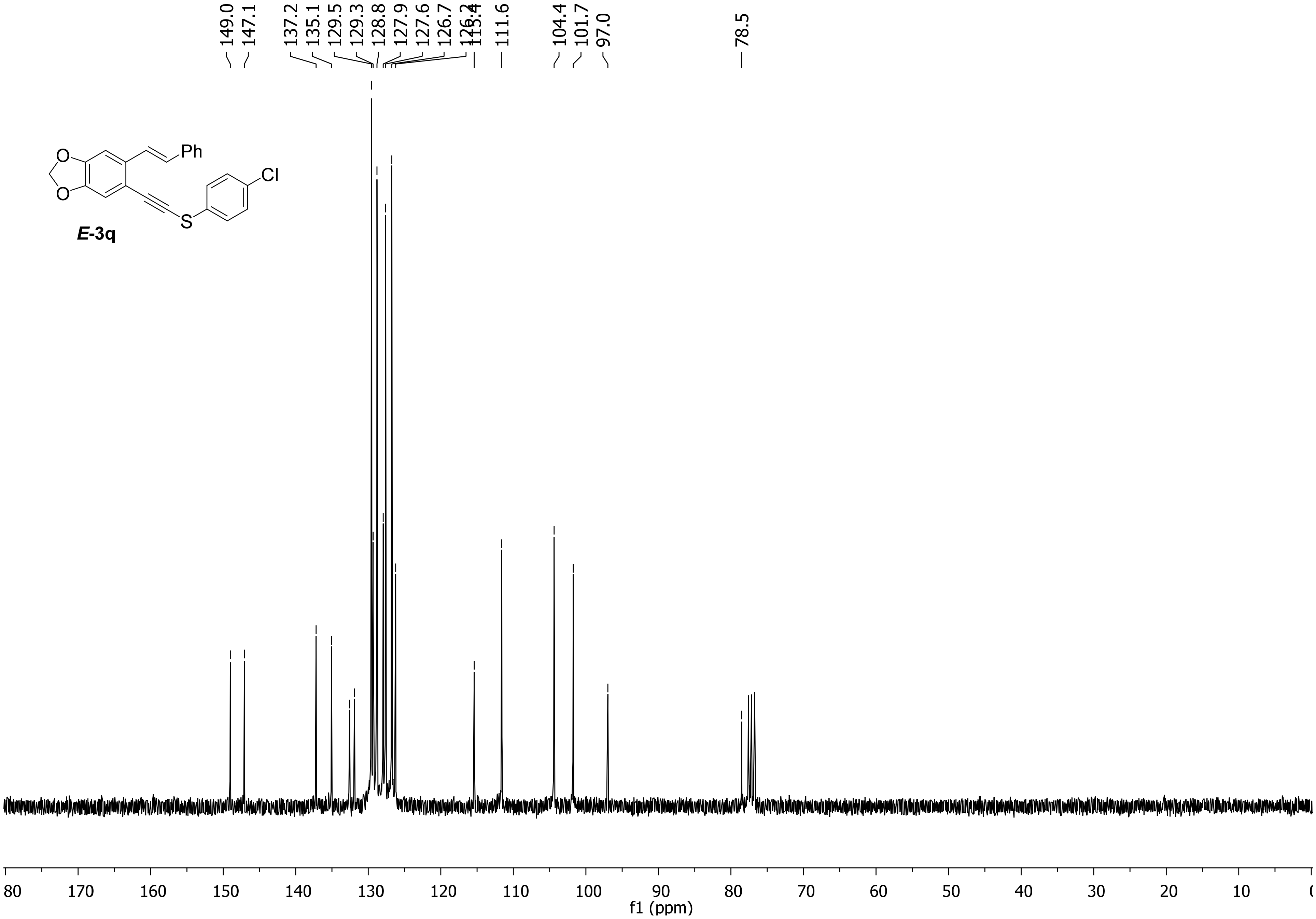

^{1}H NMR (300 MHz, $CDCl_3$)

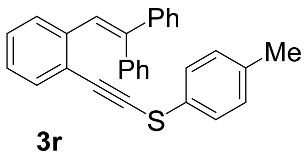

3r

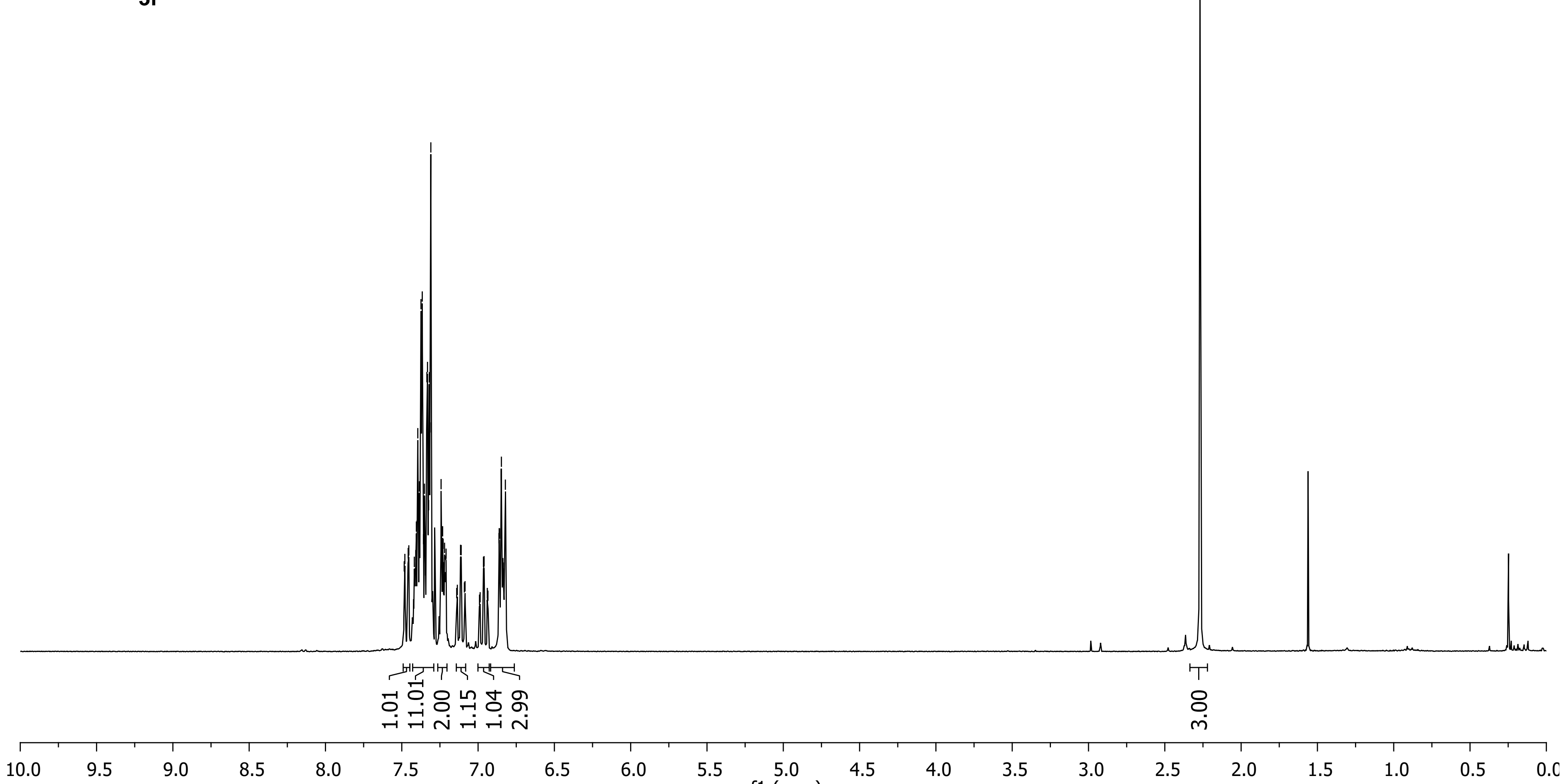

^{13}C NMR (75.4 MHz, $CDCl_3$)

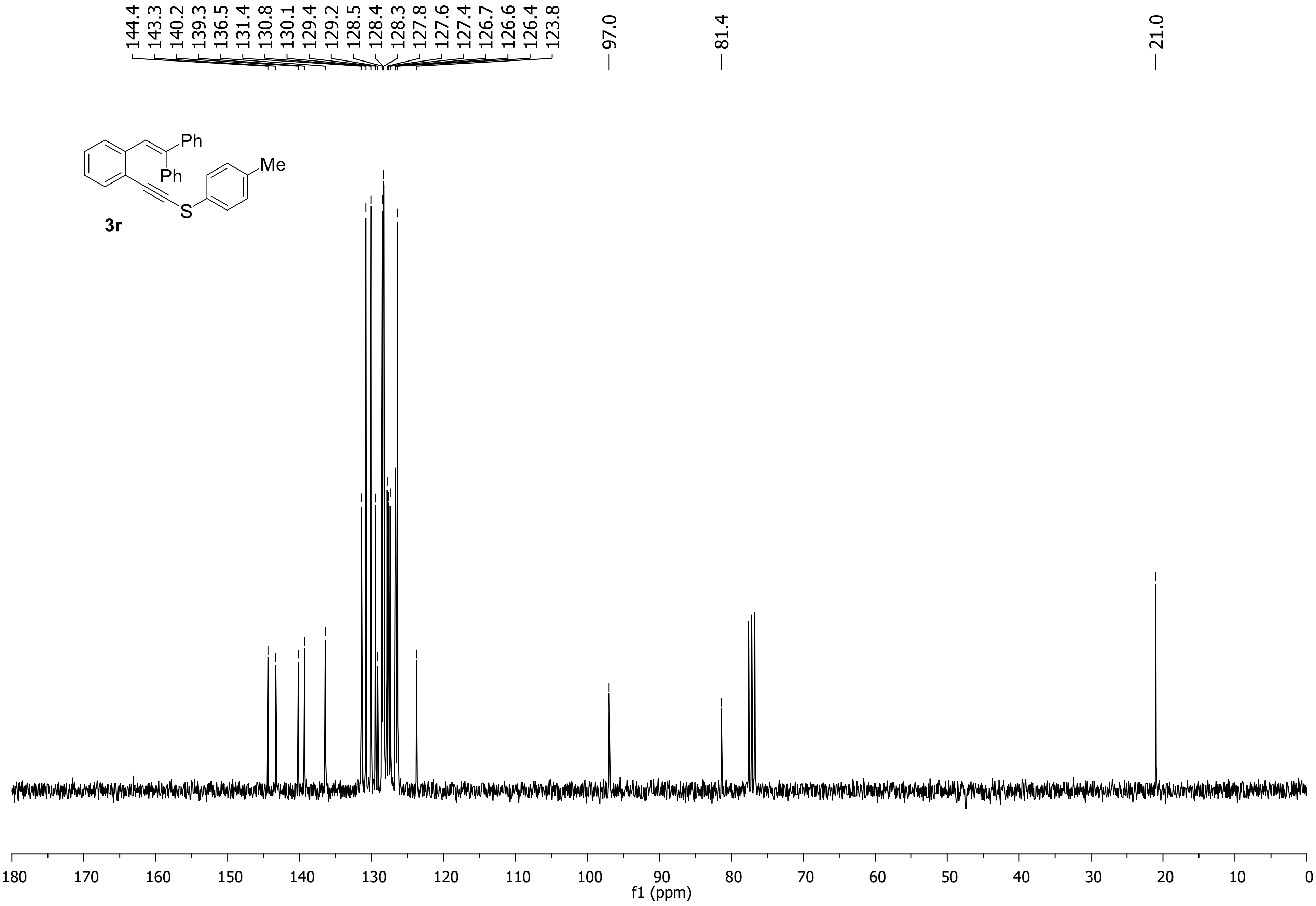

^{1}H NMR (300 MHz, $CDCl_3$)

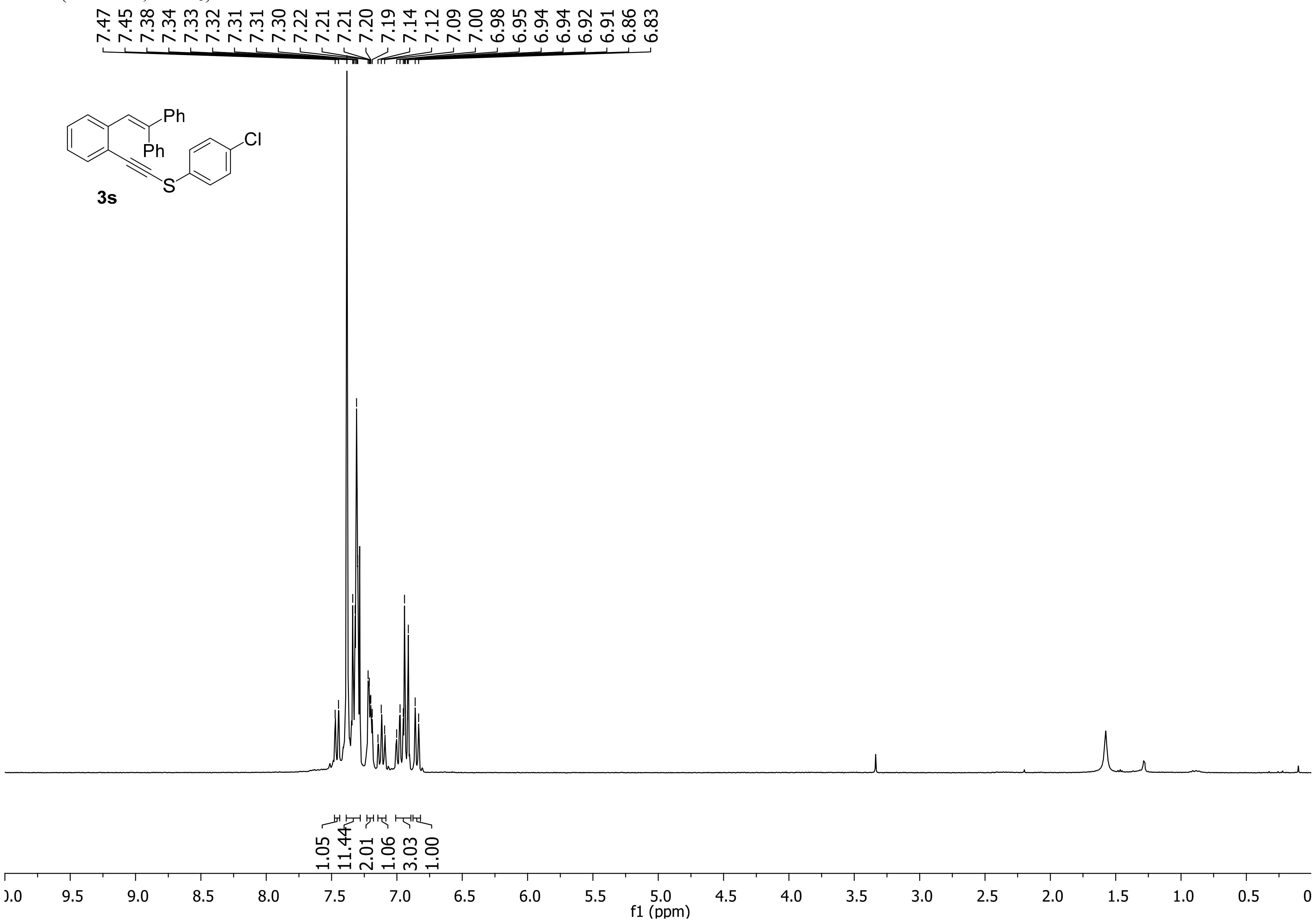

^{13}C NMR (75.4 MHz, $CDCl_3$)

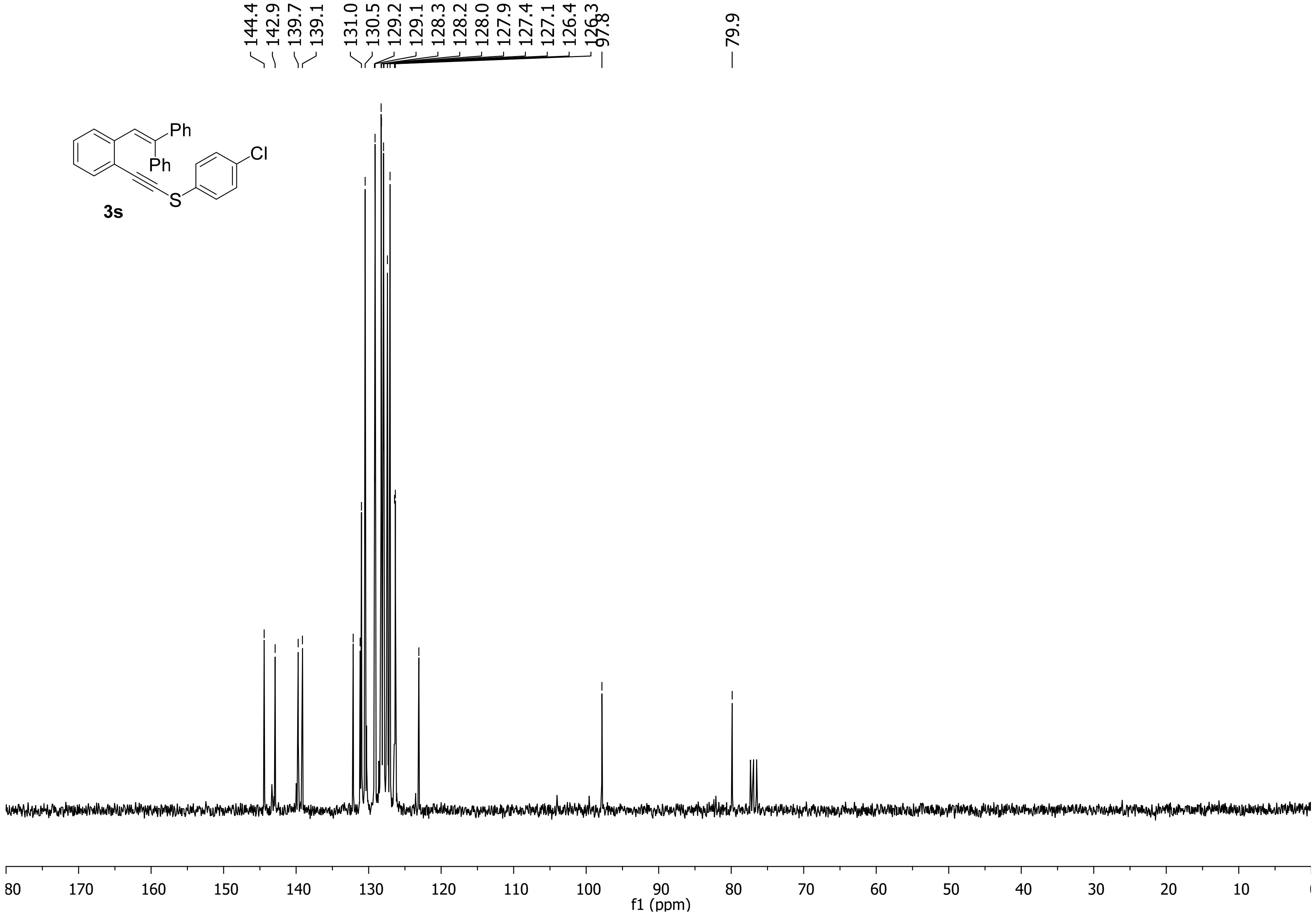

^{1}H NMR (300 MHz, $CDCl_3$)

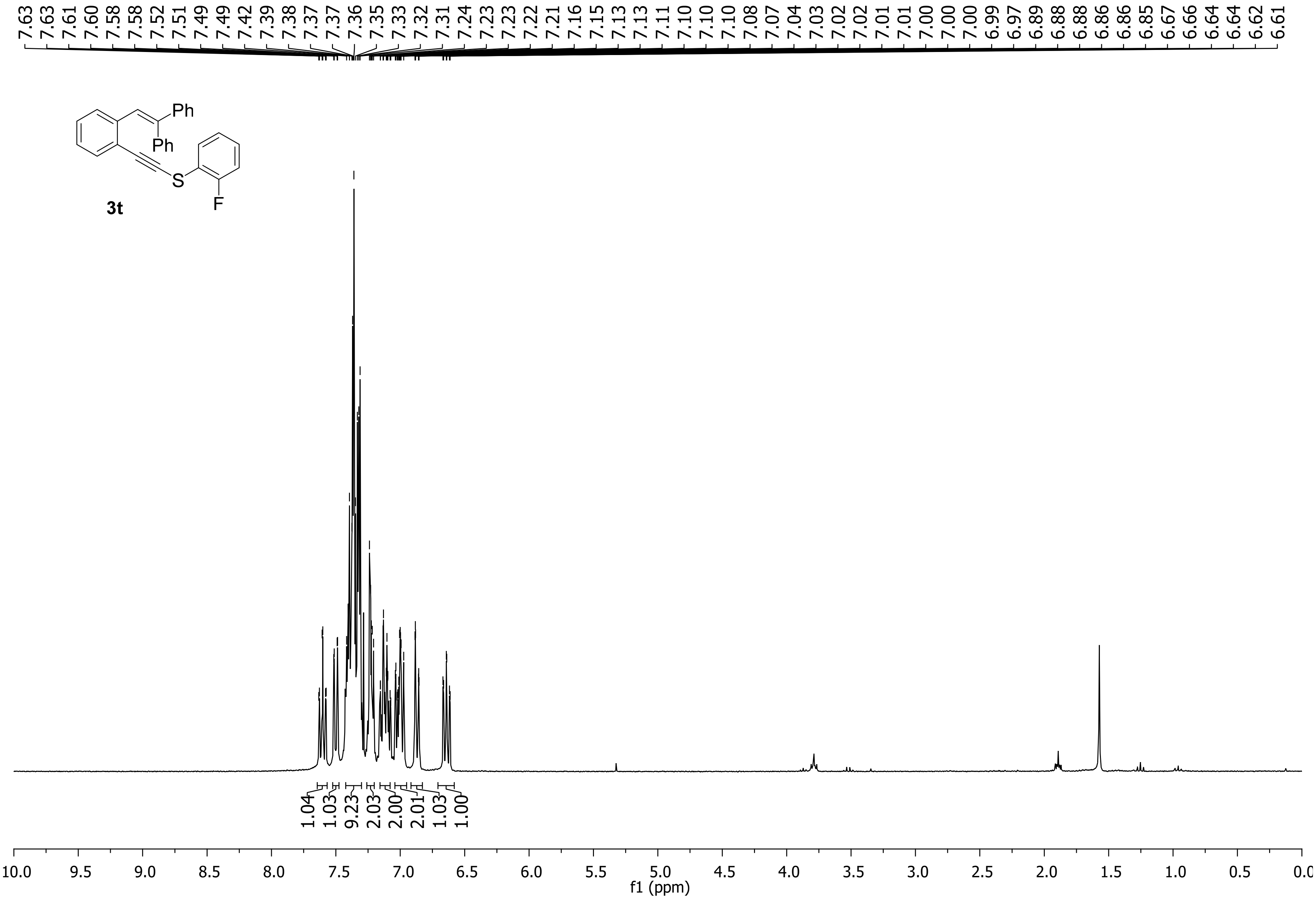

^{13}C NMR (75.4 MHz, $CDCl_3$)

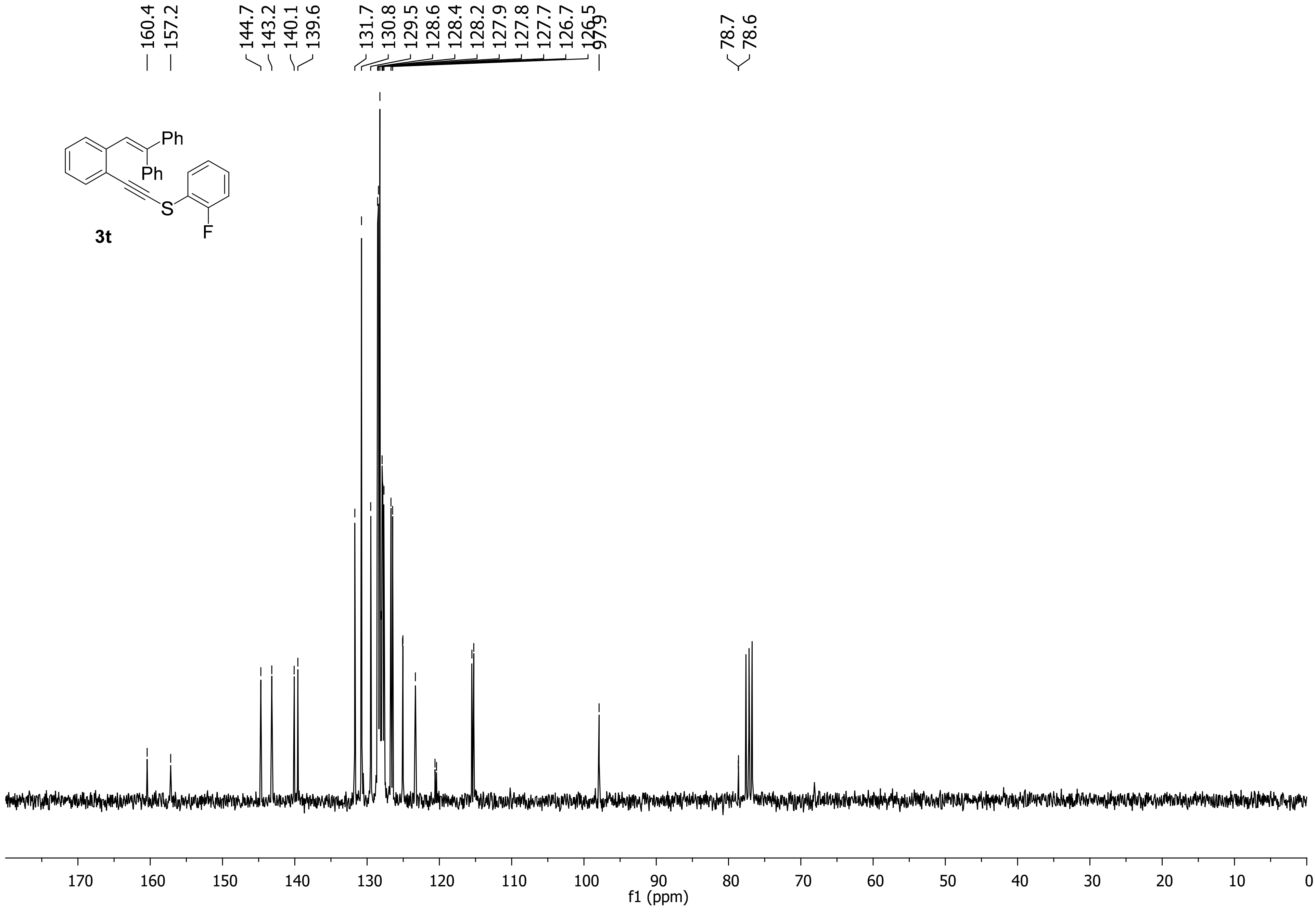

3t

^{1}H NMR (300 MHz, $CDCl_3$)

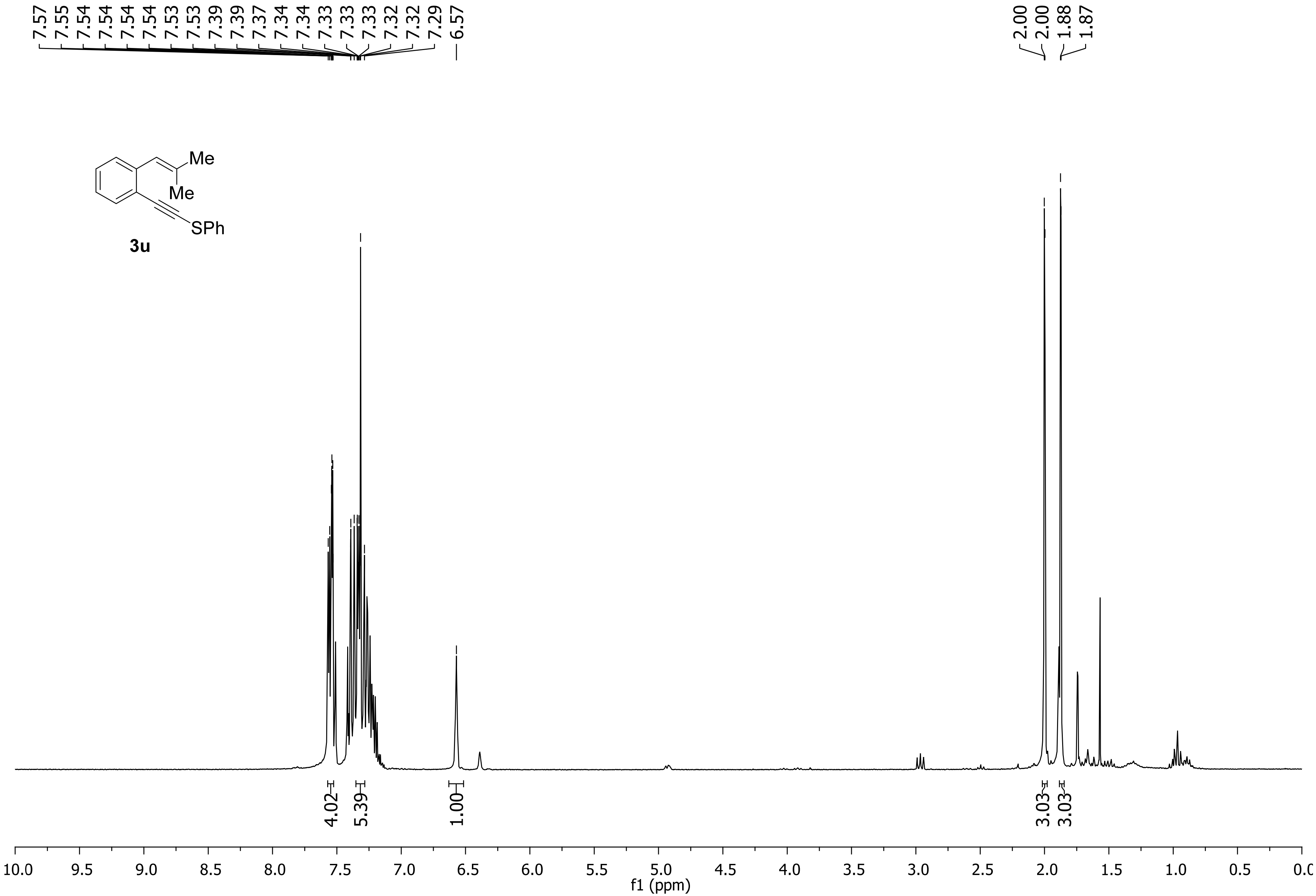

^{13}C NMR (75.4 MHz, $CDCl_3$)

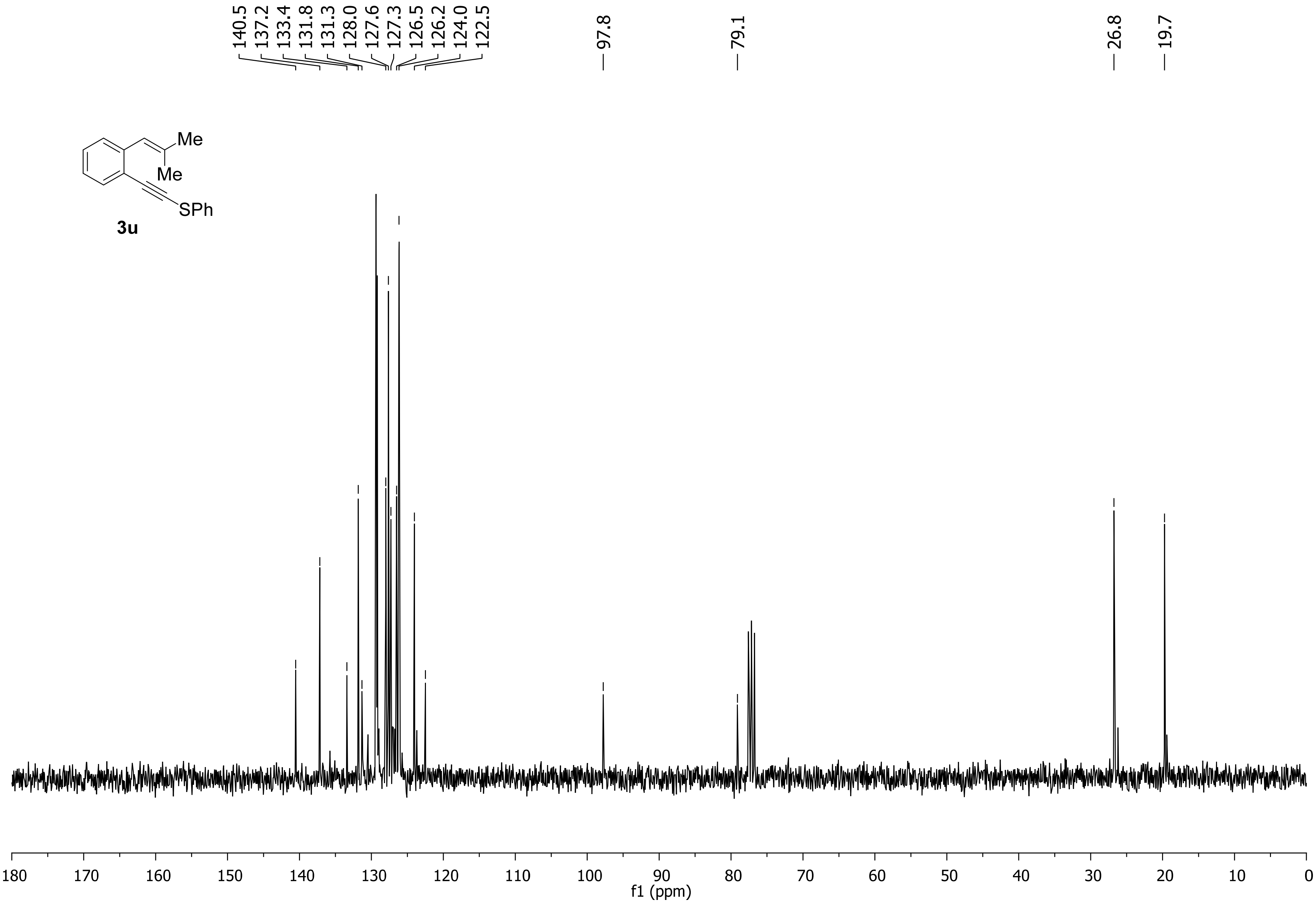

^{1}H NMR (300 MHz, $CDCl_3$)

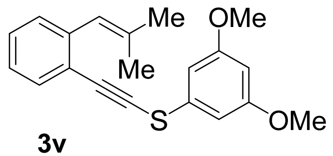

3v

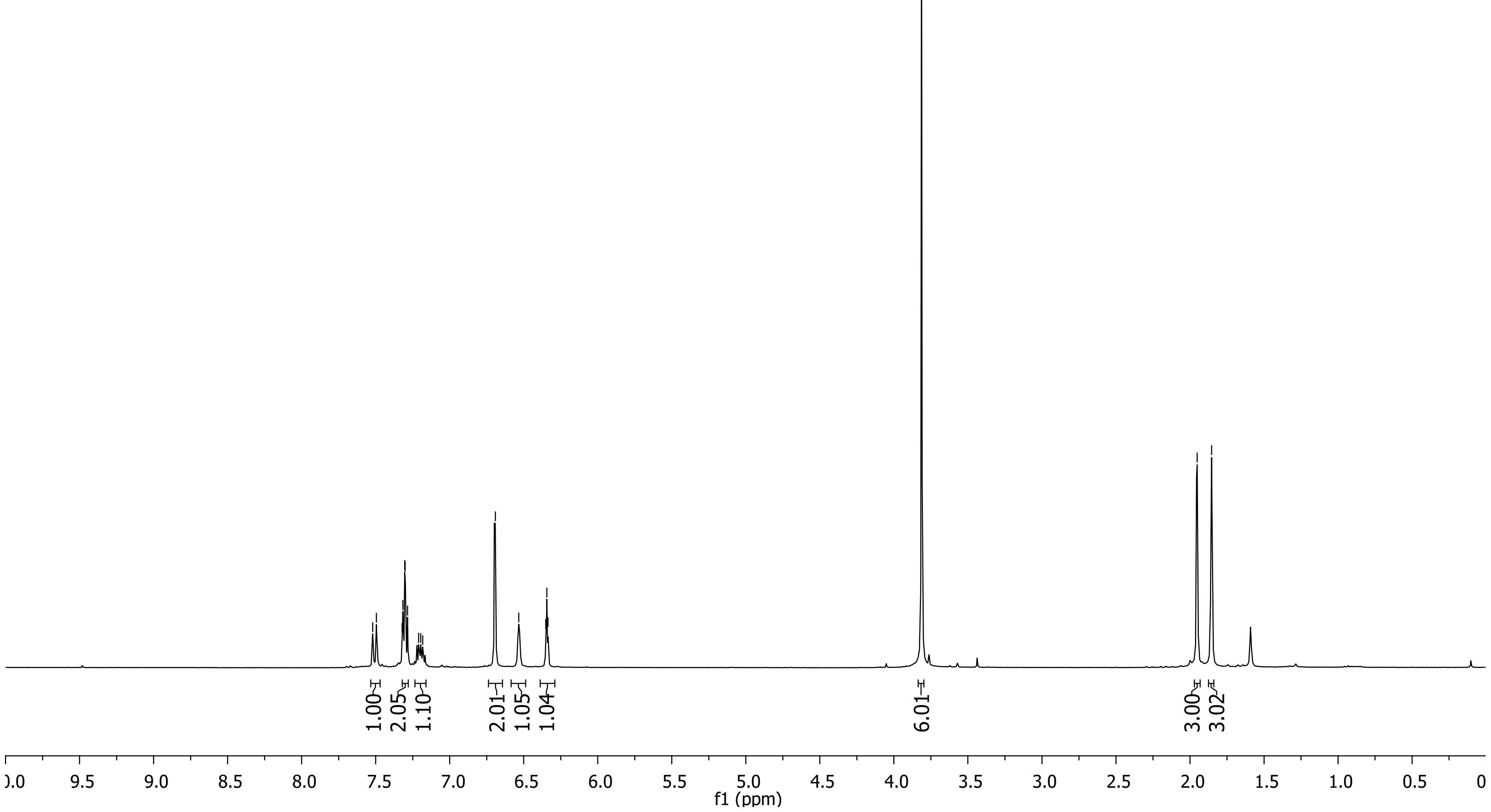

^{13}C NMR (75.4 MHz, $CDCl_3$)

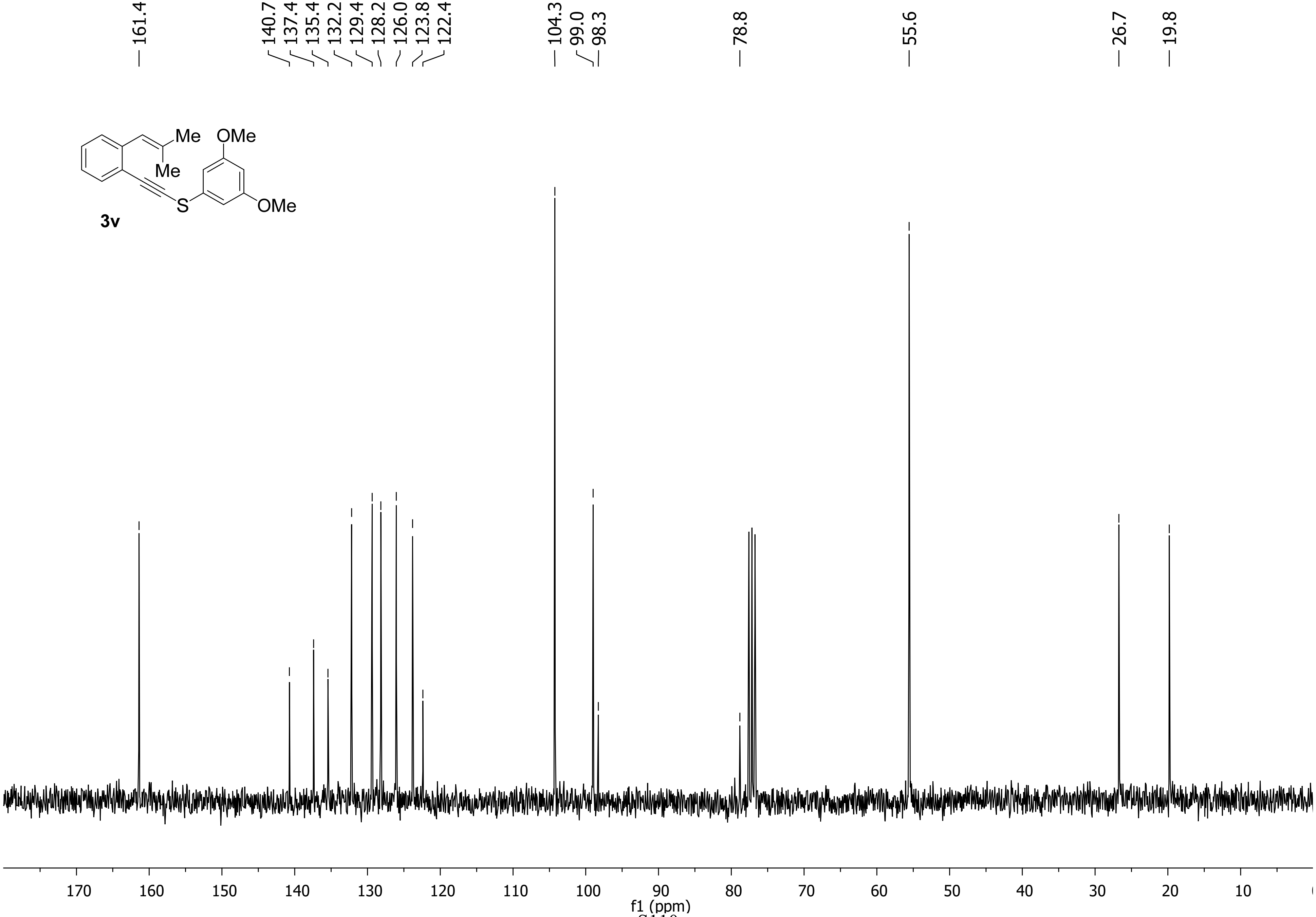

^{1}H NMR (300 MHz, CDCl$_3$)

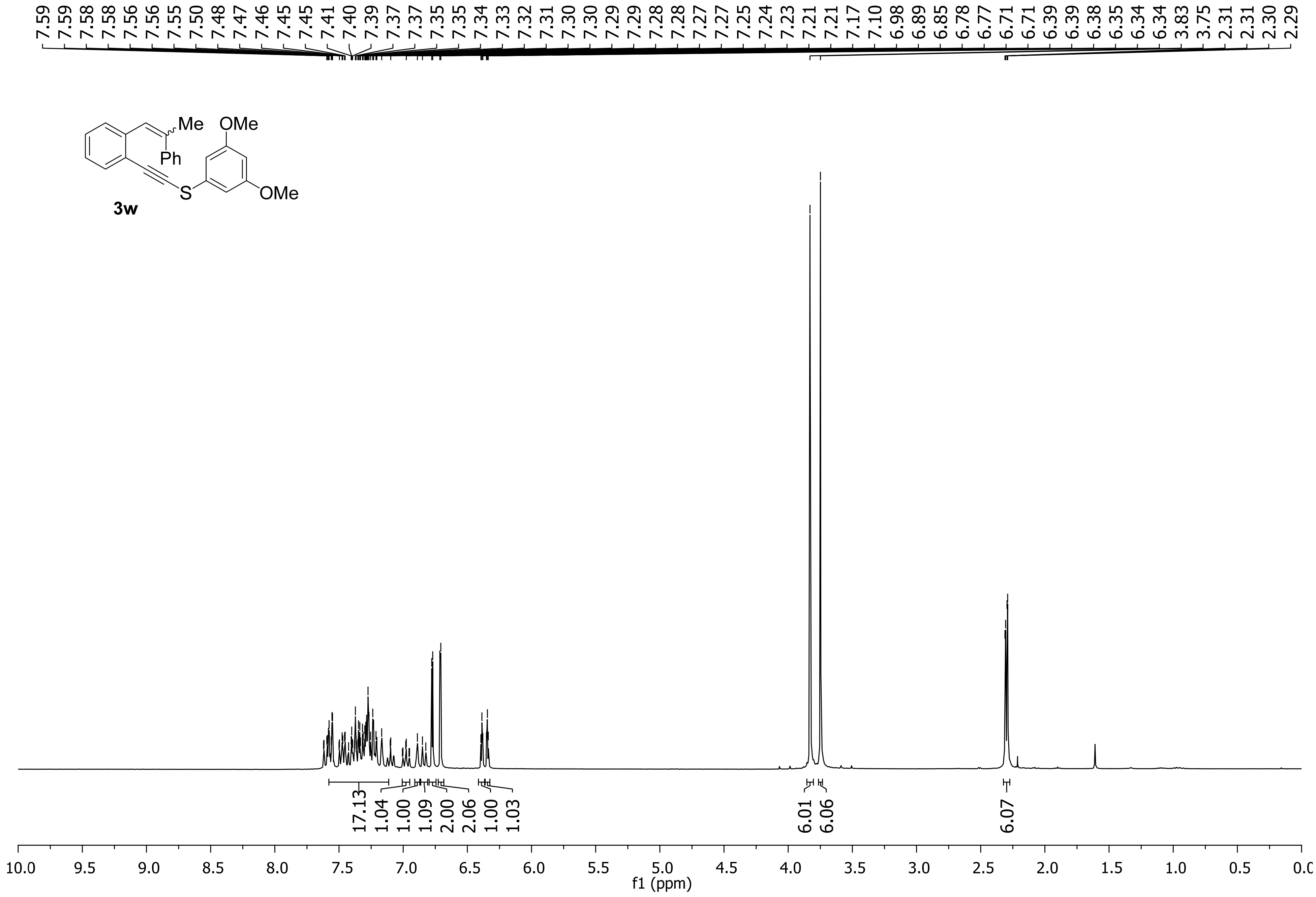

^{13}C NMR (75.4 MHz, $CDCl_3$)

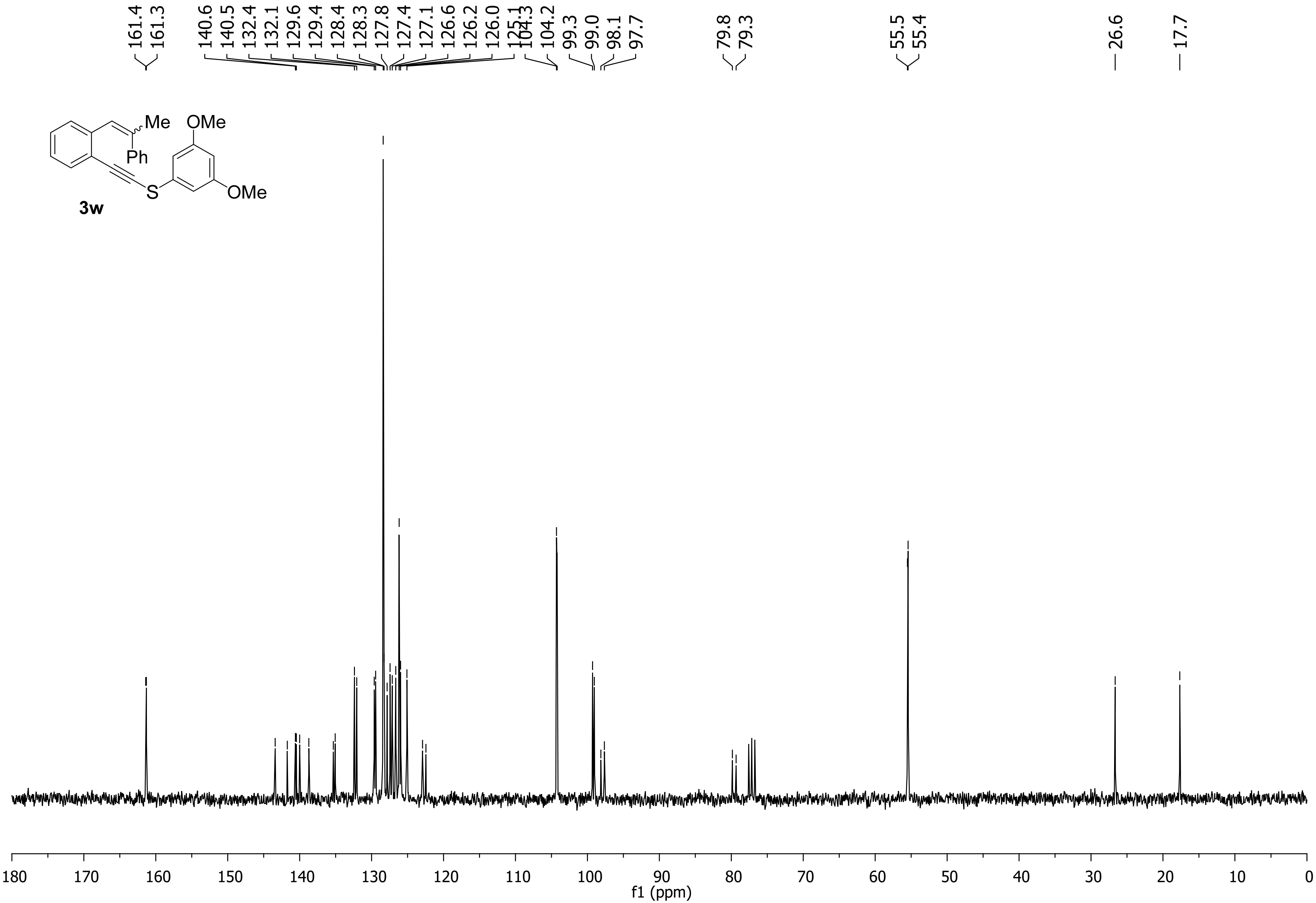

^{1}H NMR (300 MHz, $CDCl_3$)

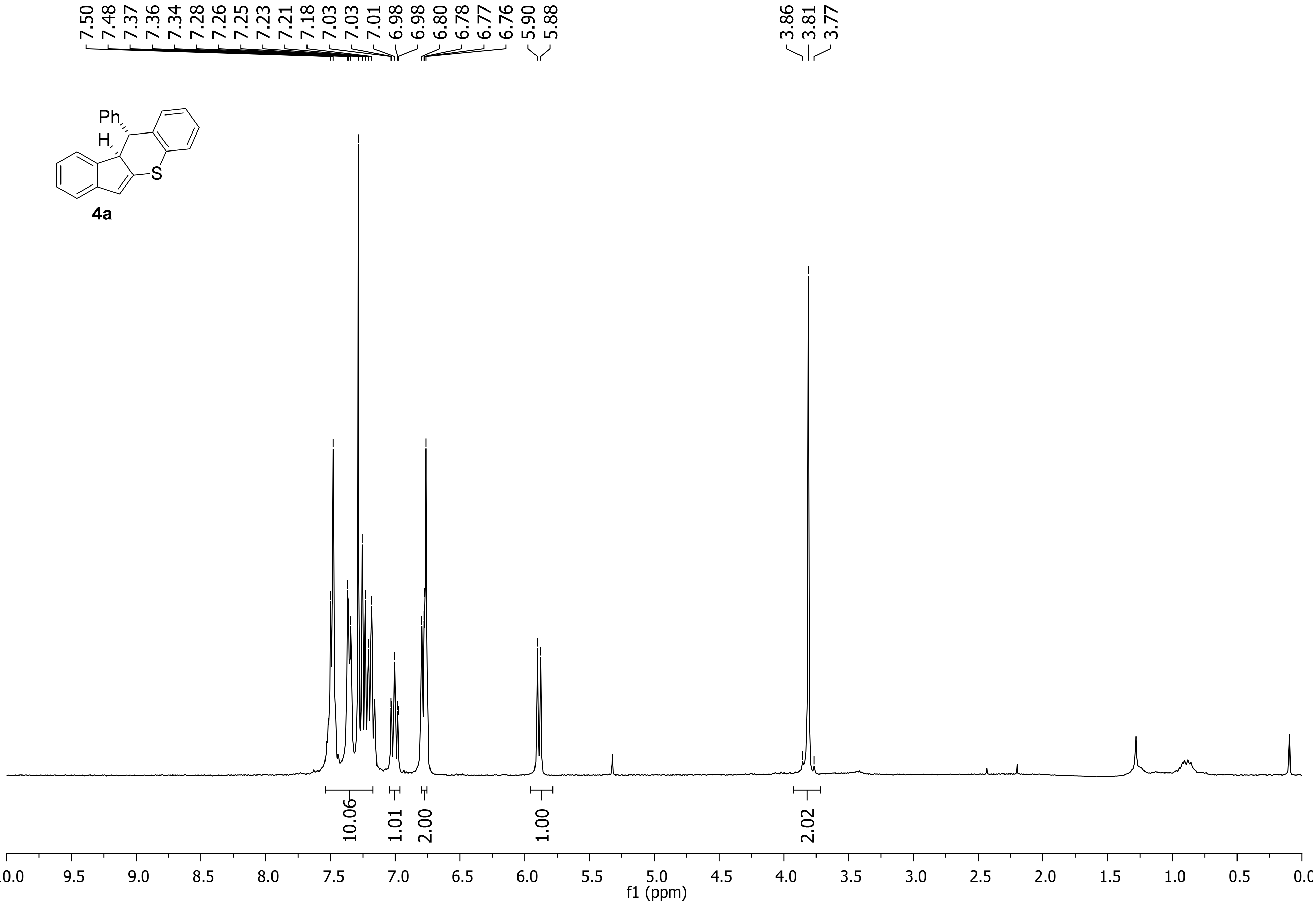

4a

^{13}C NMR (75.4 MHz, $CDCl_3$)

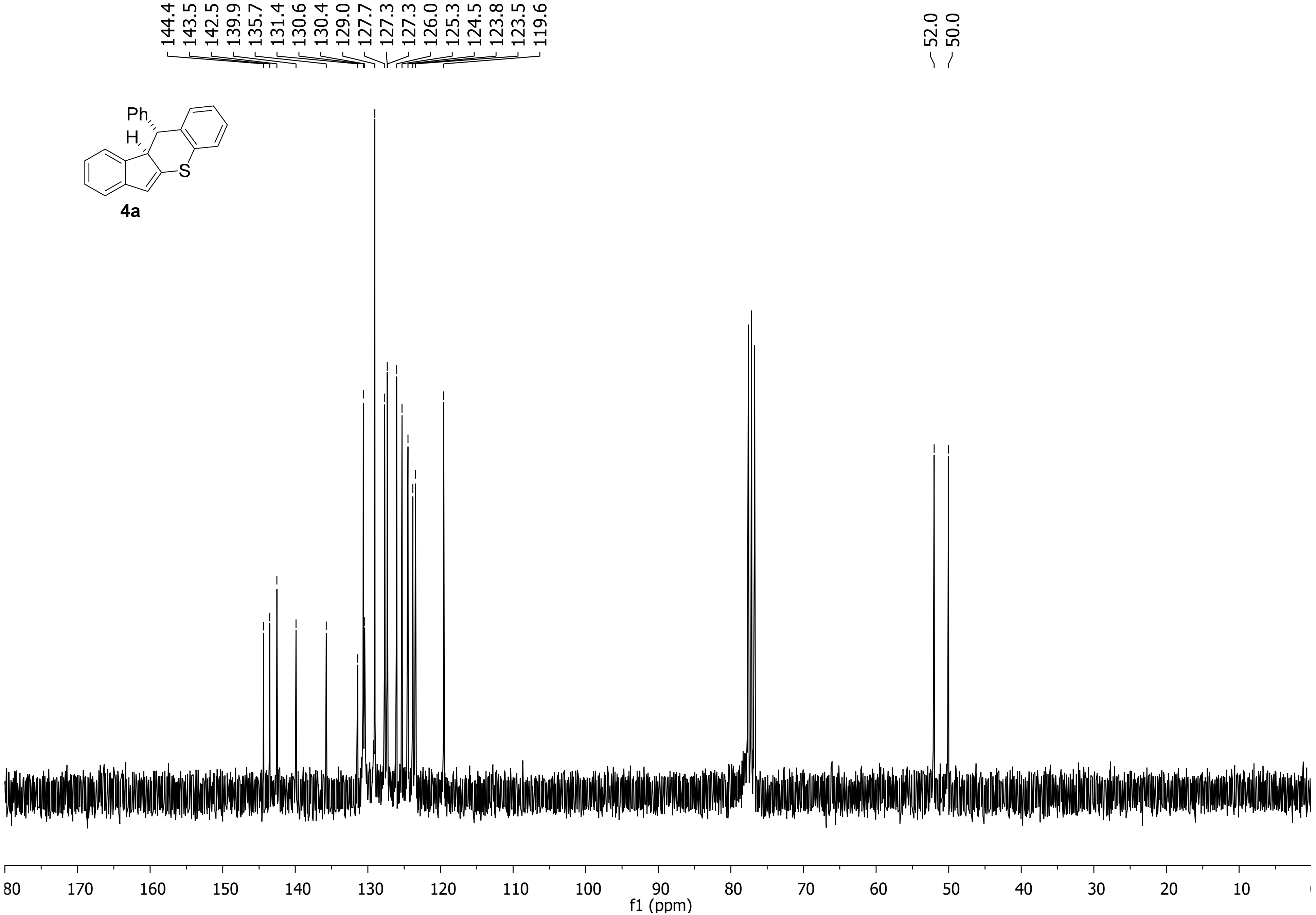

^{1}H NMR (300 MHz, $CDCl_3$)

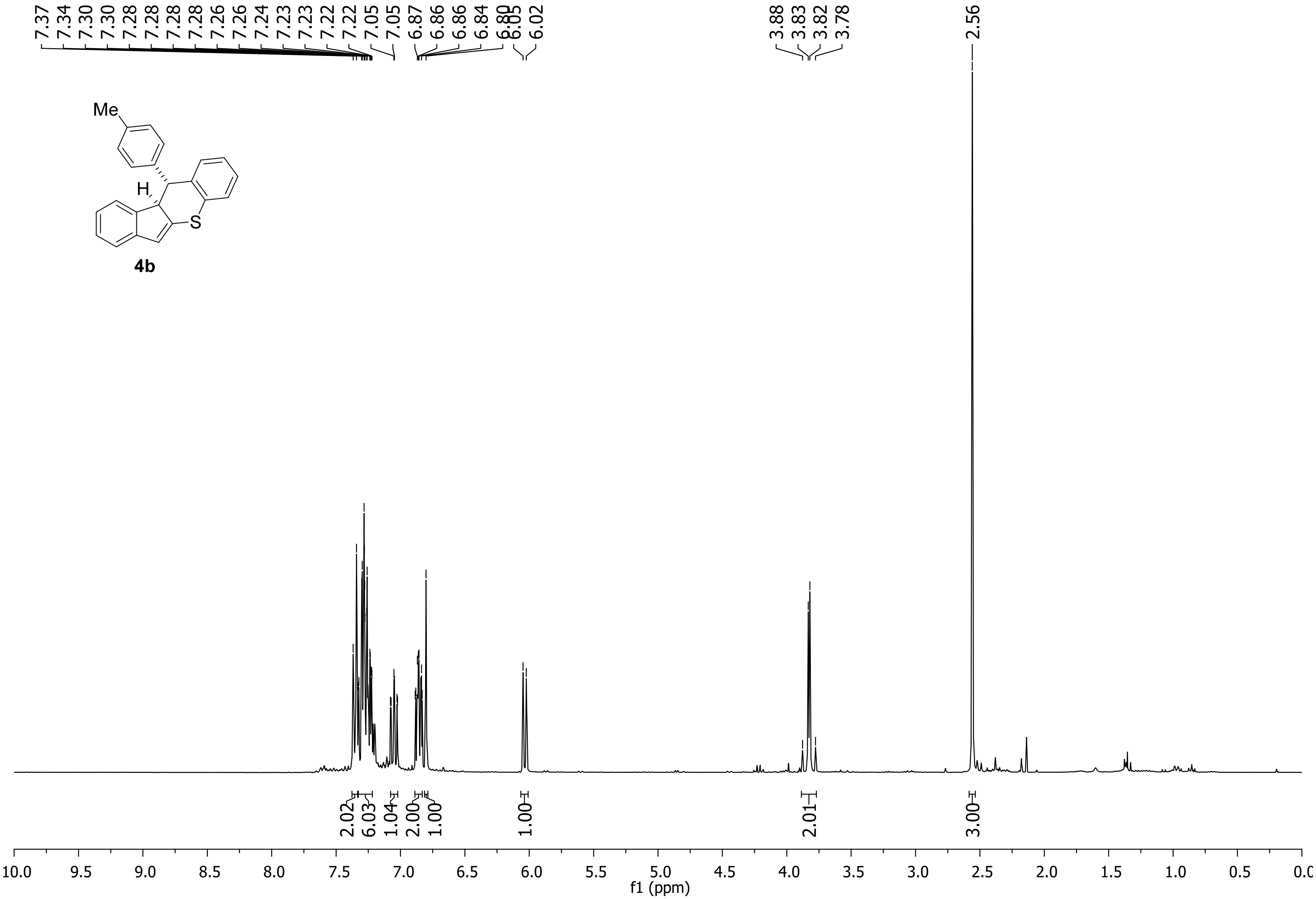

^{13}C NMR (75.4 MHz, $CDCl_3$)

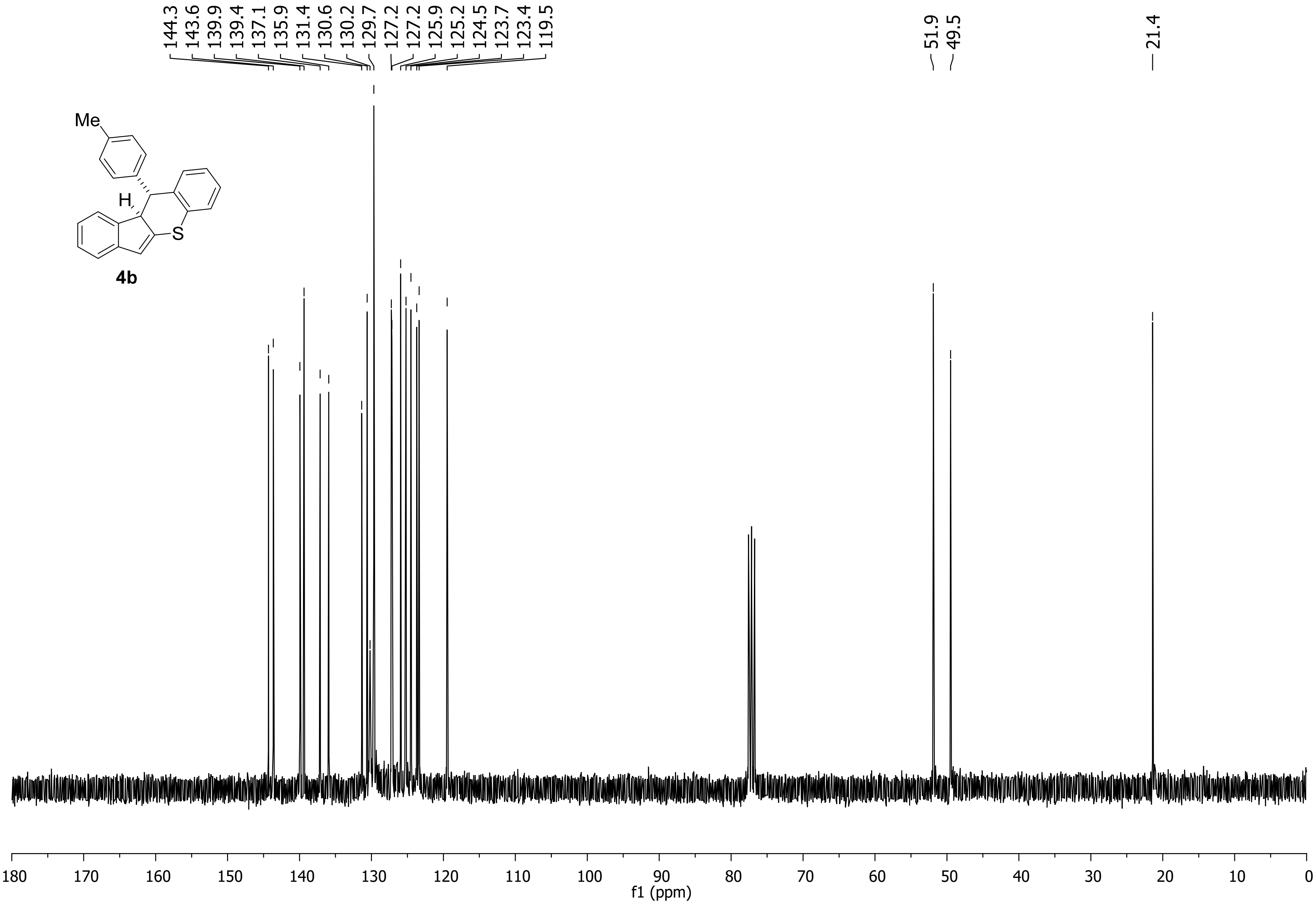

^{1}H NMR (300 MHz, $CDCl_3$)

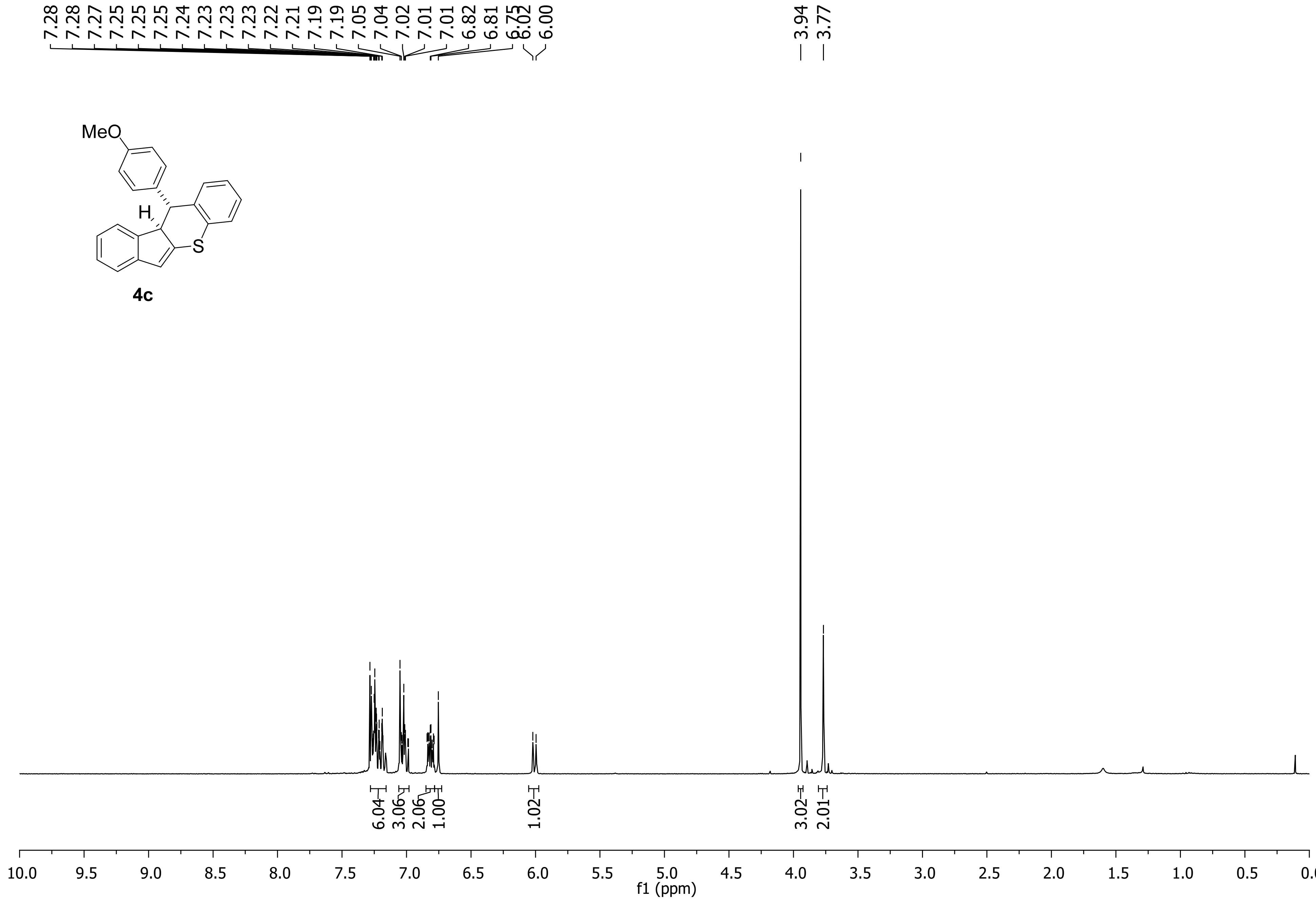

^{13}C NMR (75.4 MHz, $CDCl_3$)

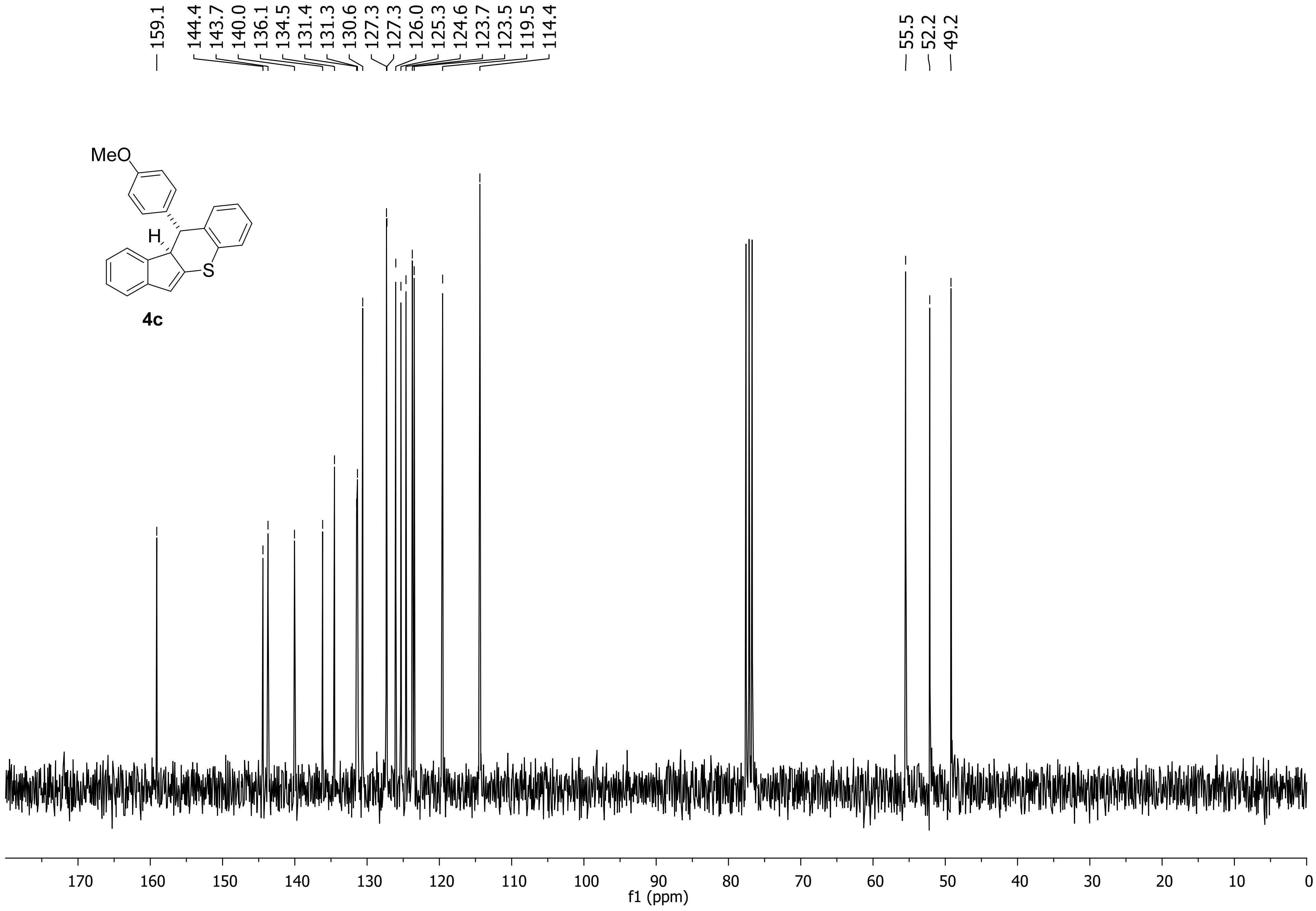

4c

^{1}H NMR (300 MHz, $CDCl_3$)

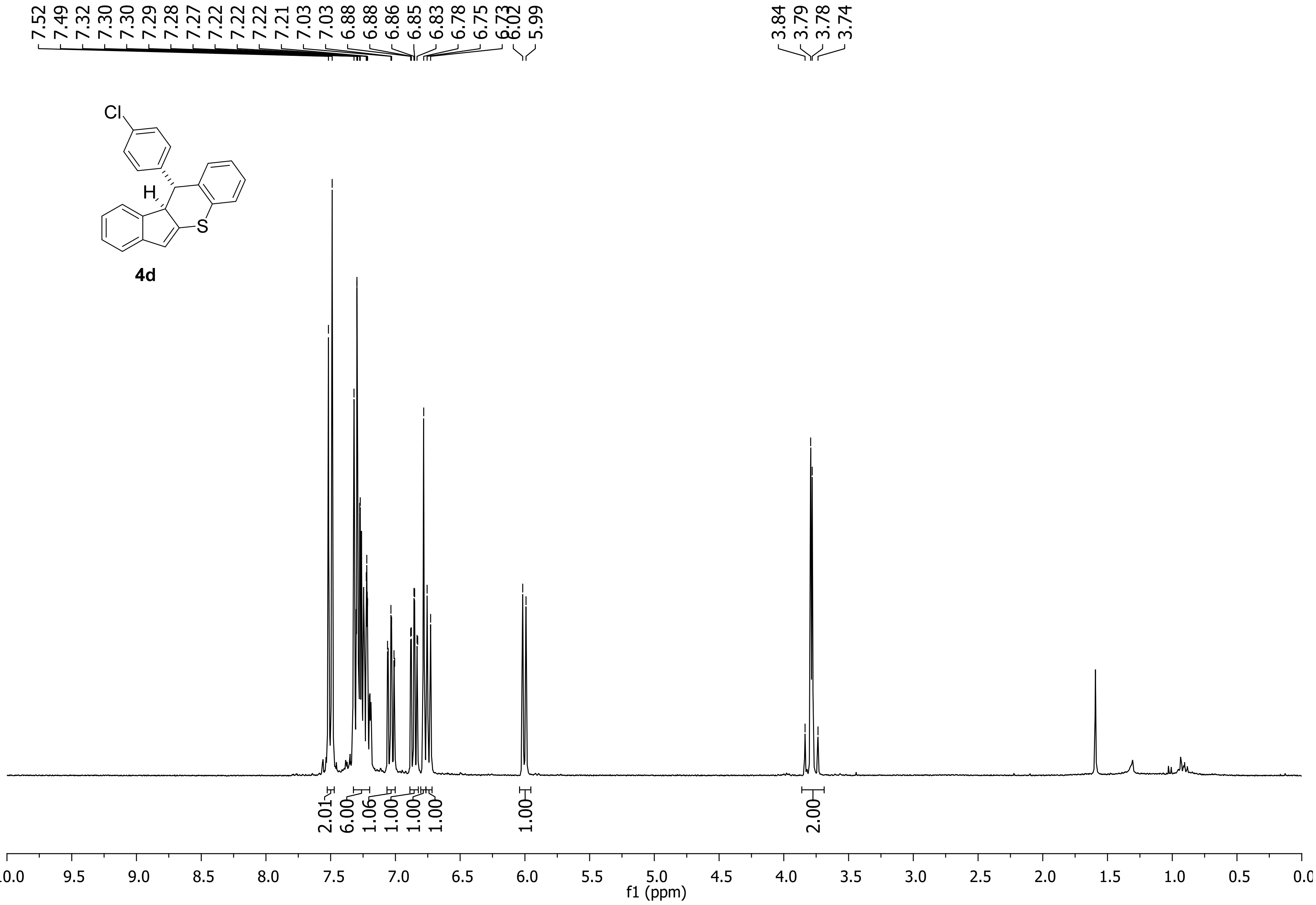

^{13}C NMR (75.4 MHz, $CDCl_3$)

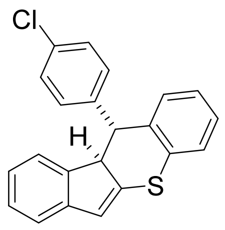

4d

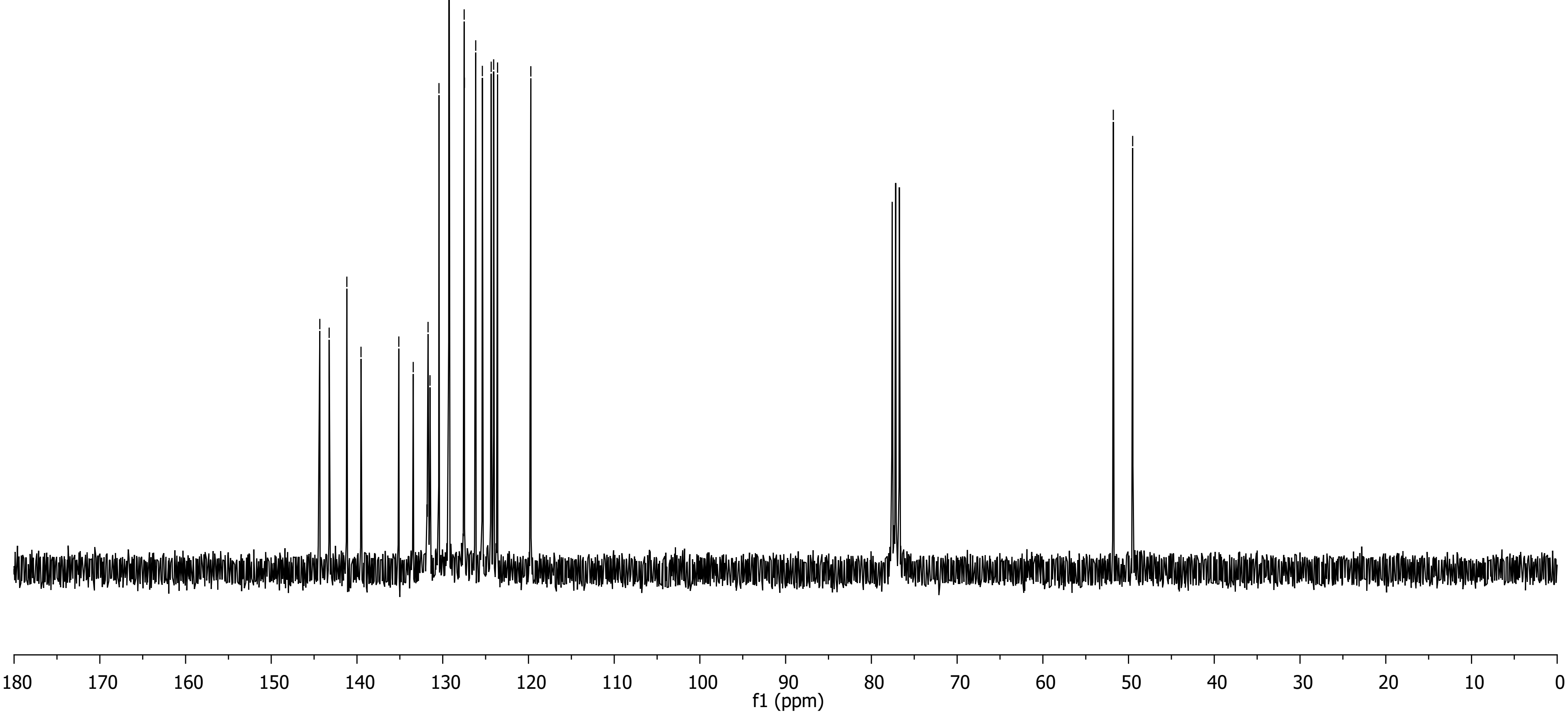

^1H NMR (300 MHz, $CDCl_3$)

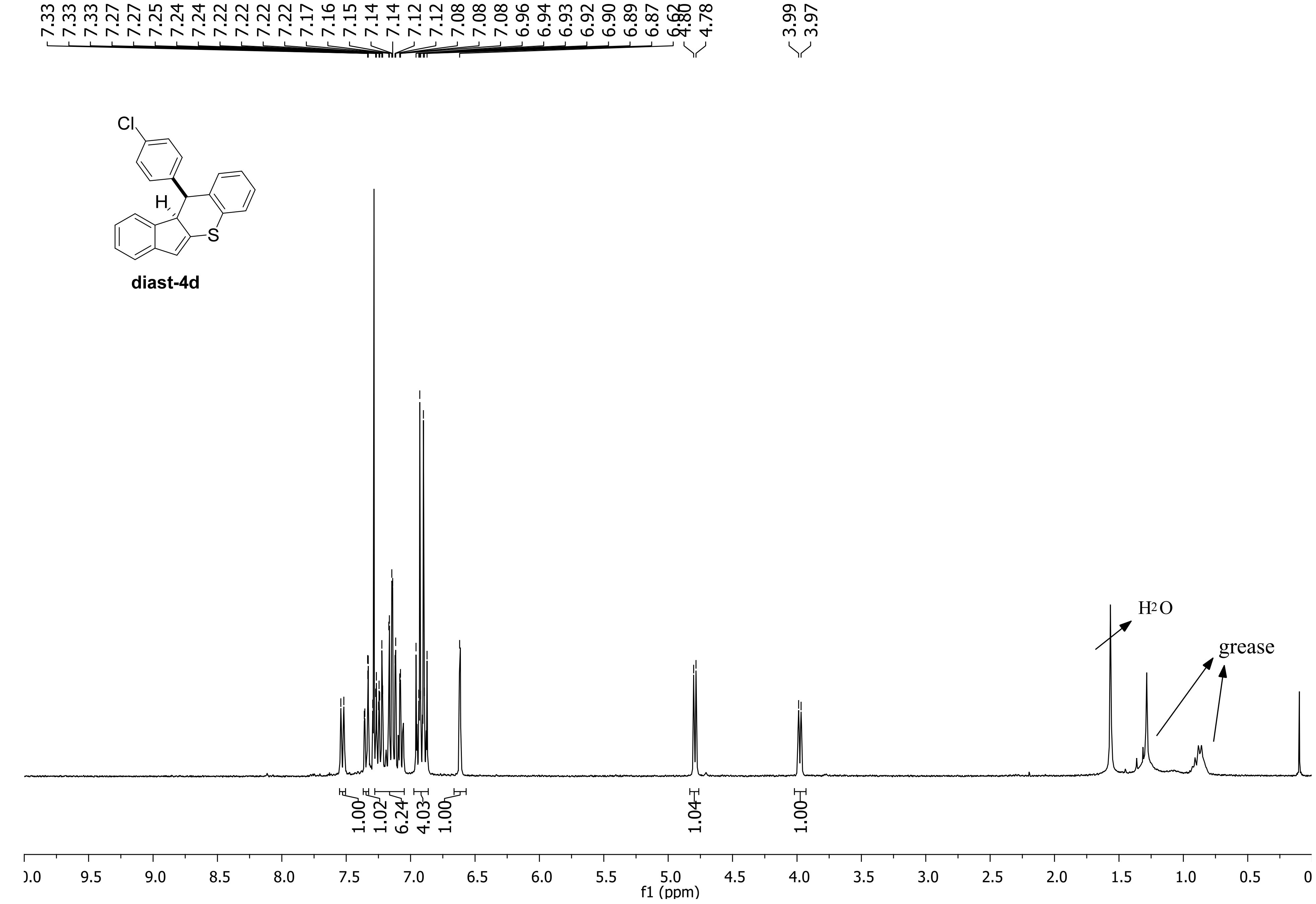

^{13}C NMR (75.4 MHz, $CDCl_3$)

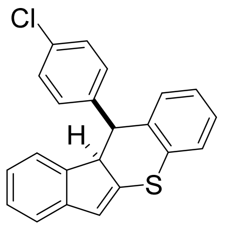

diast-4d

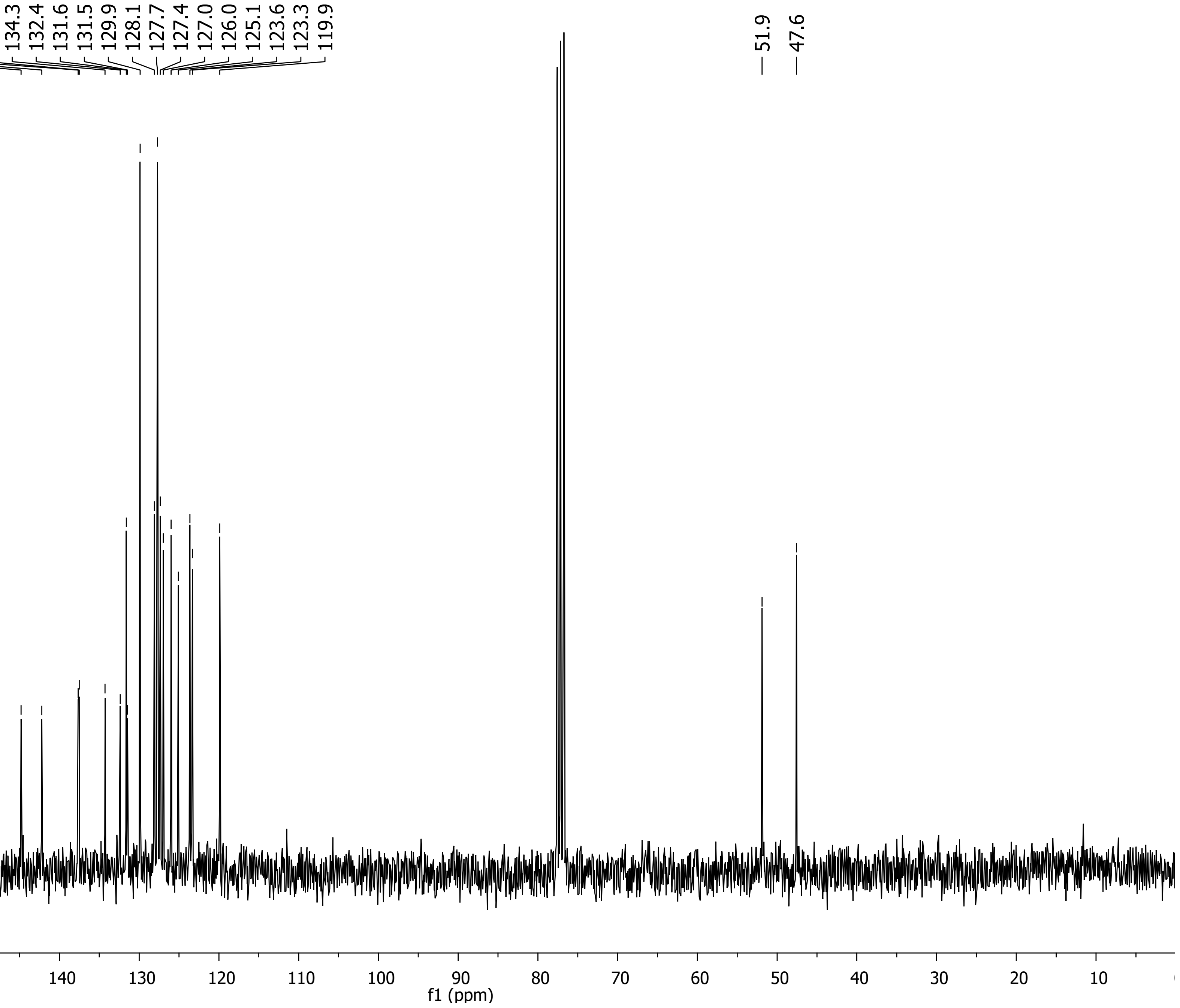

^{1}H NMR (300 MHz, $CDCl_3$)

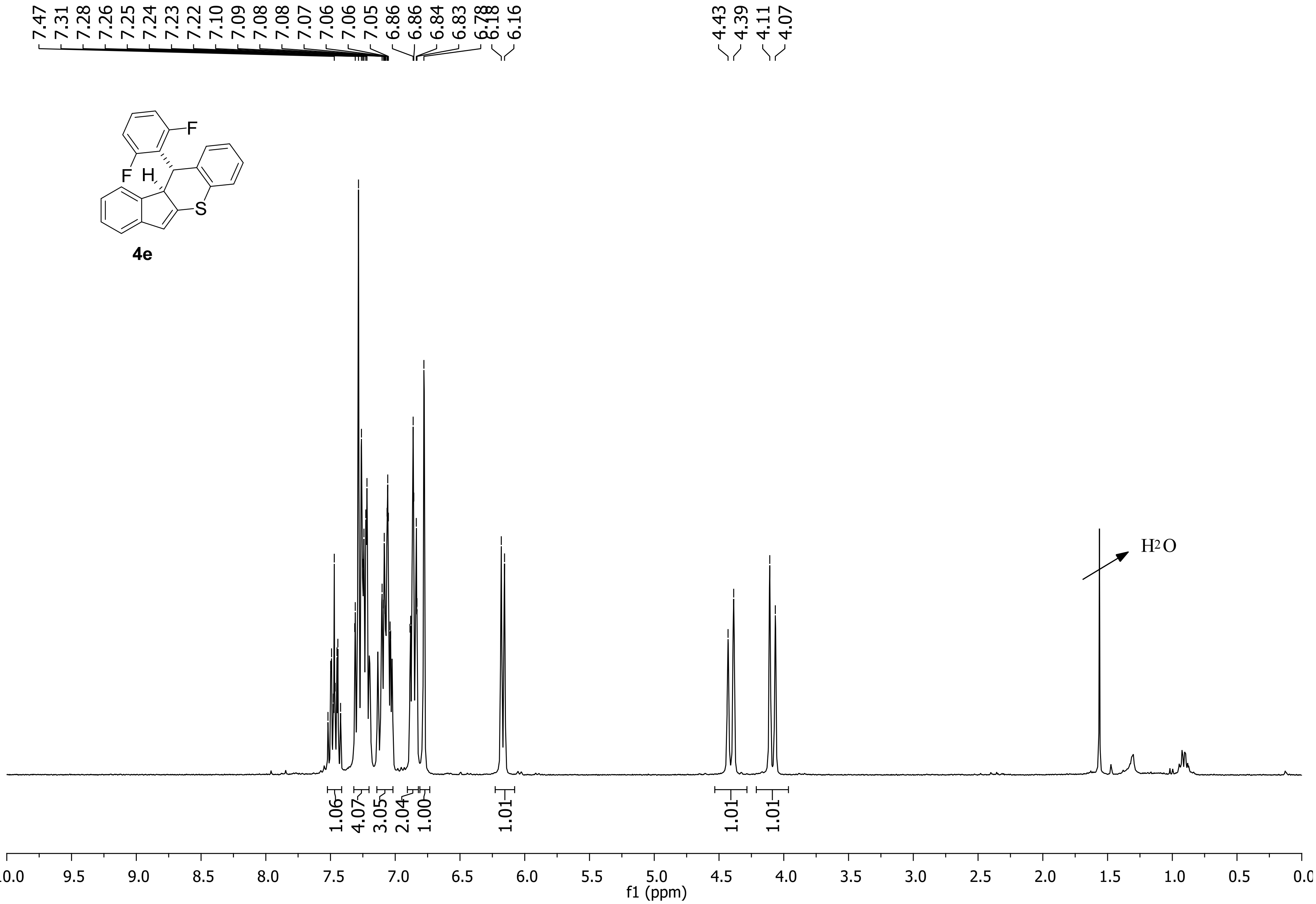

^{13}C NMR (75.4 MHz, $CDCl_3$)

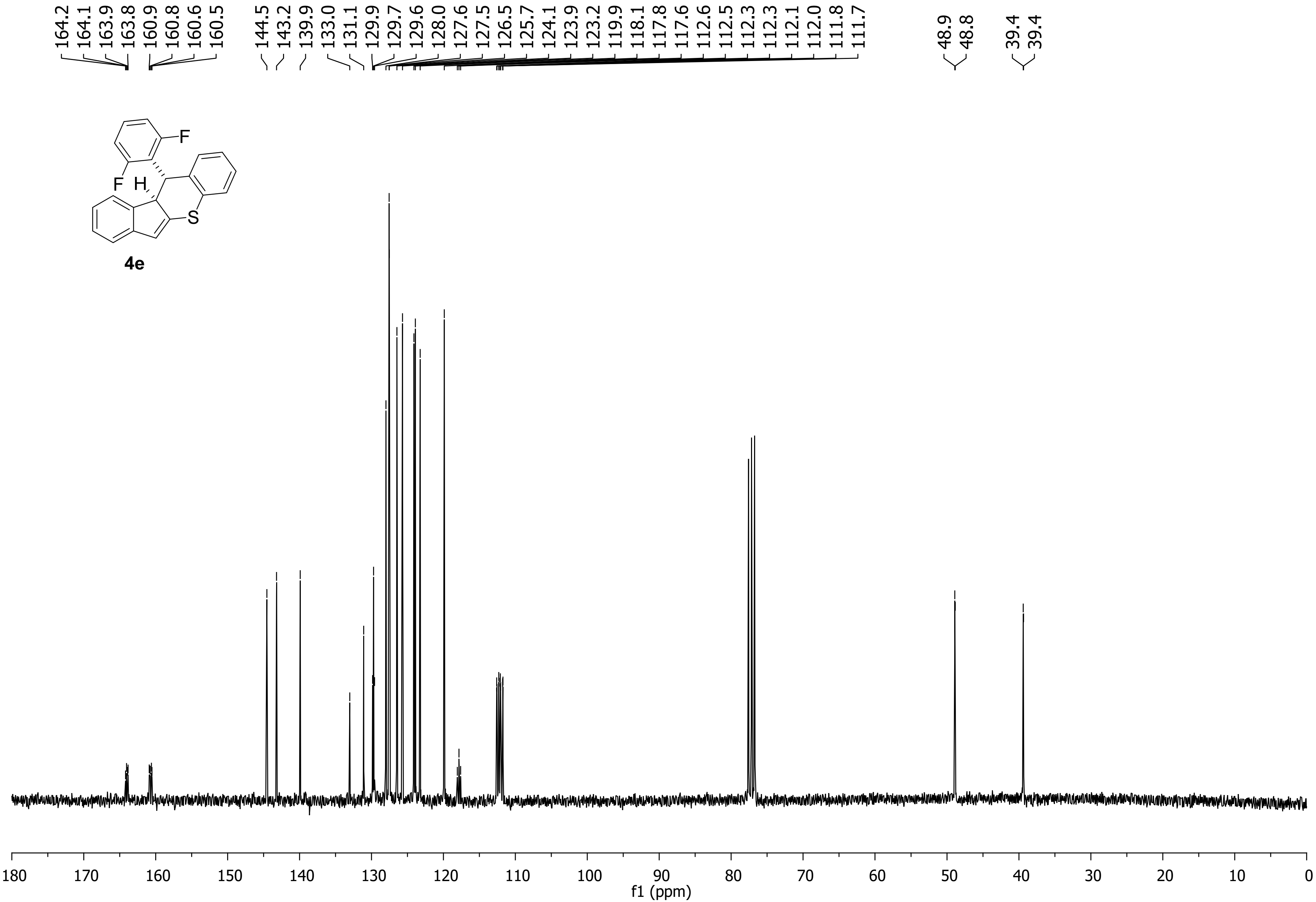

^{1}H NMR (300 MHz, $CDCl_3$)

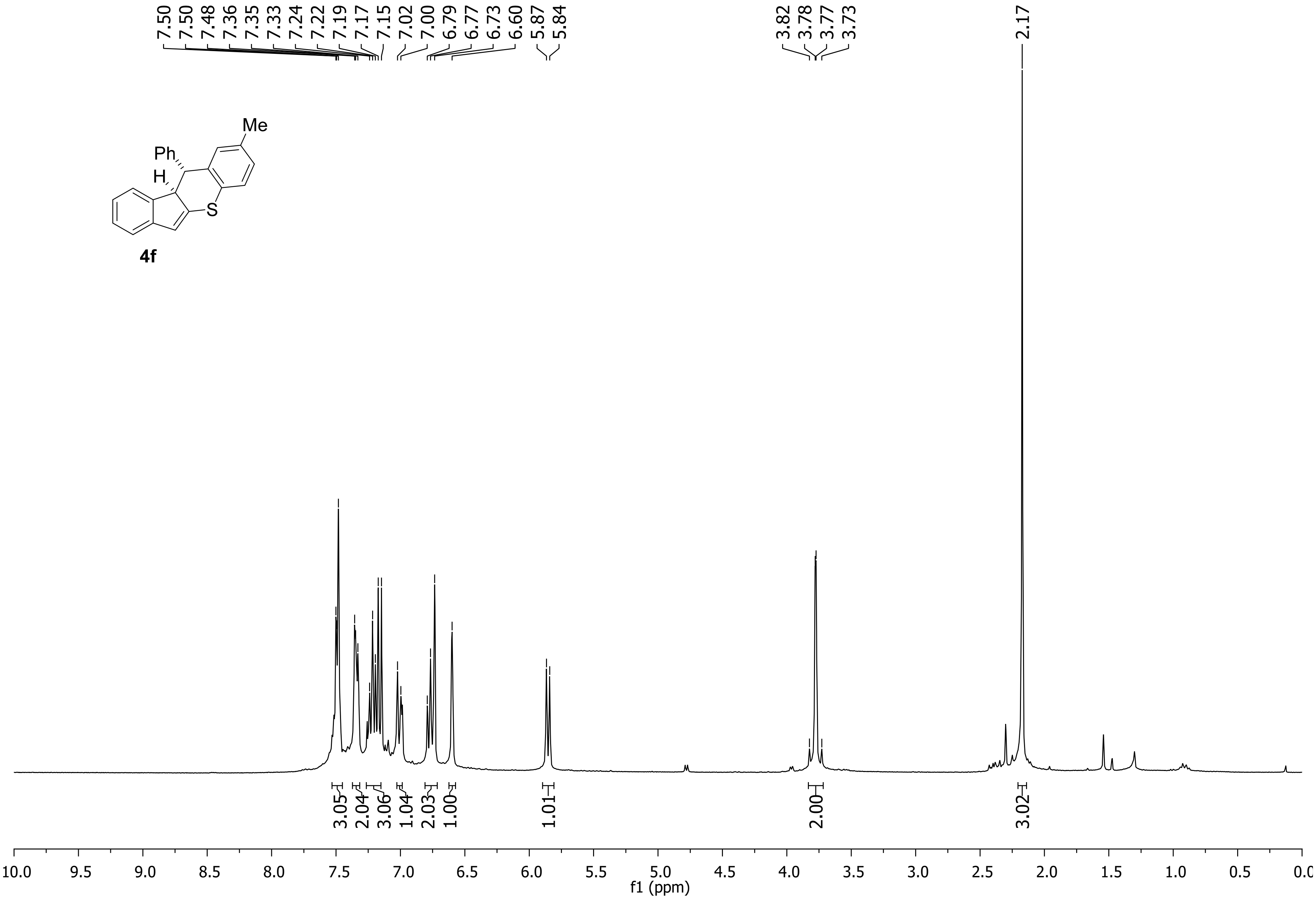

^{13}C NMR (75.4 MHz, $CDCl_3$)

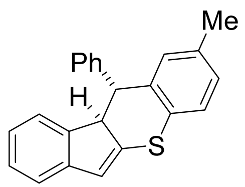

4f

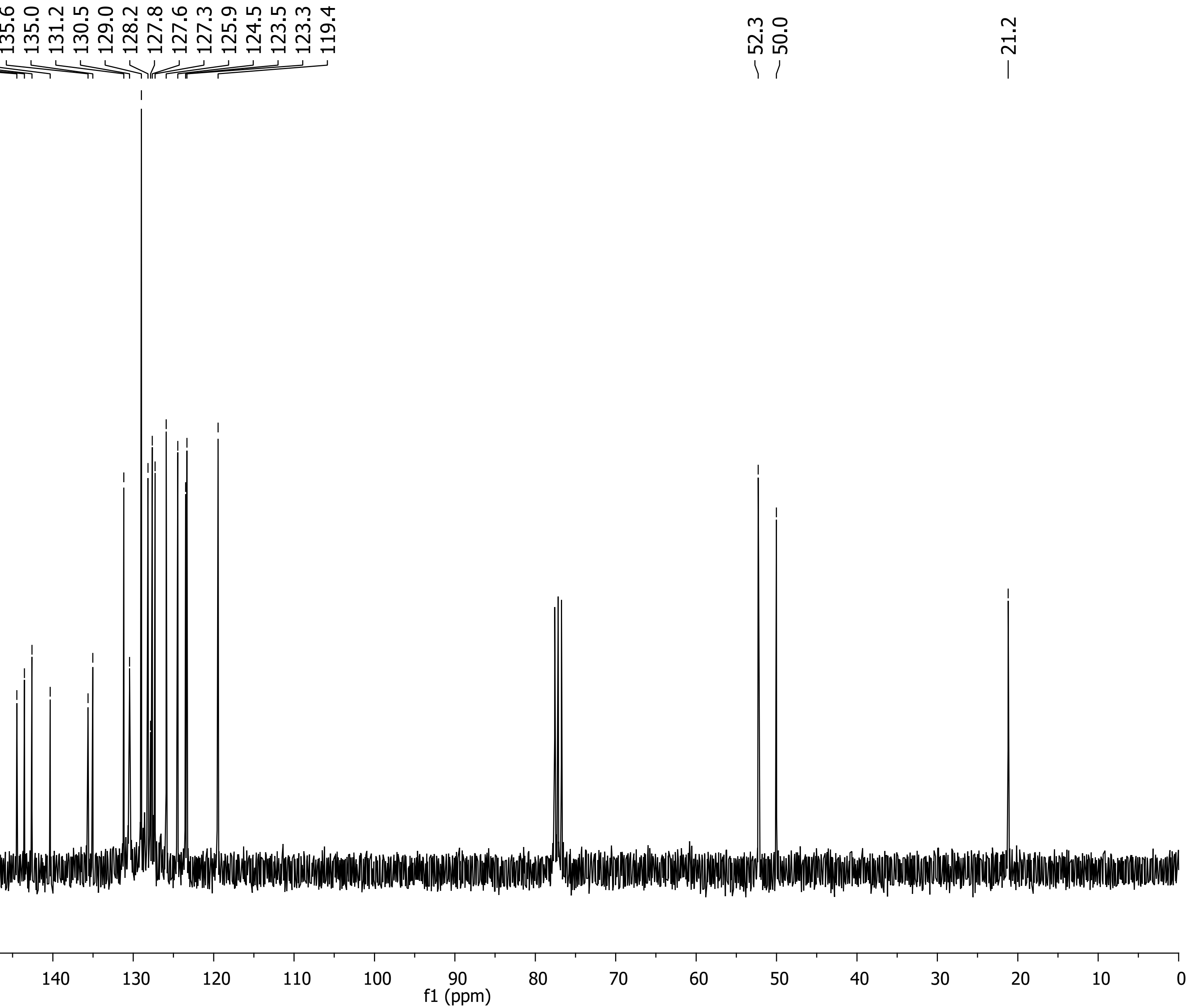

^{1}H NMR (300 MHz, $CDCl_3$)

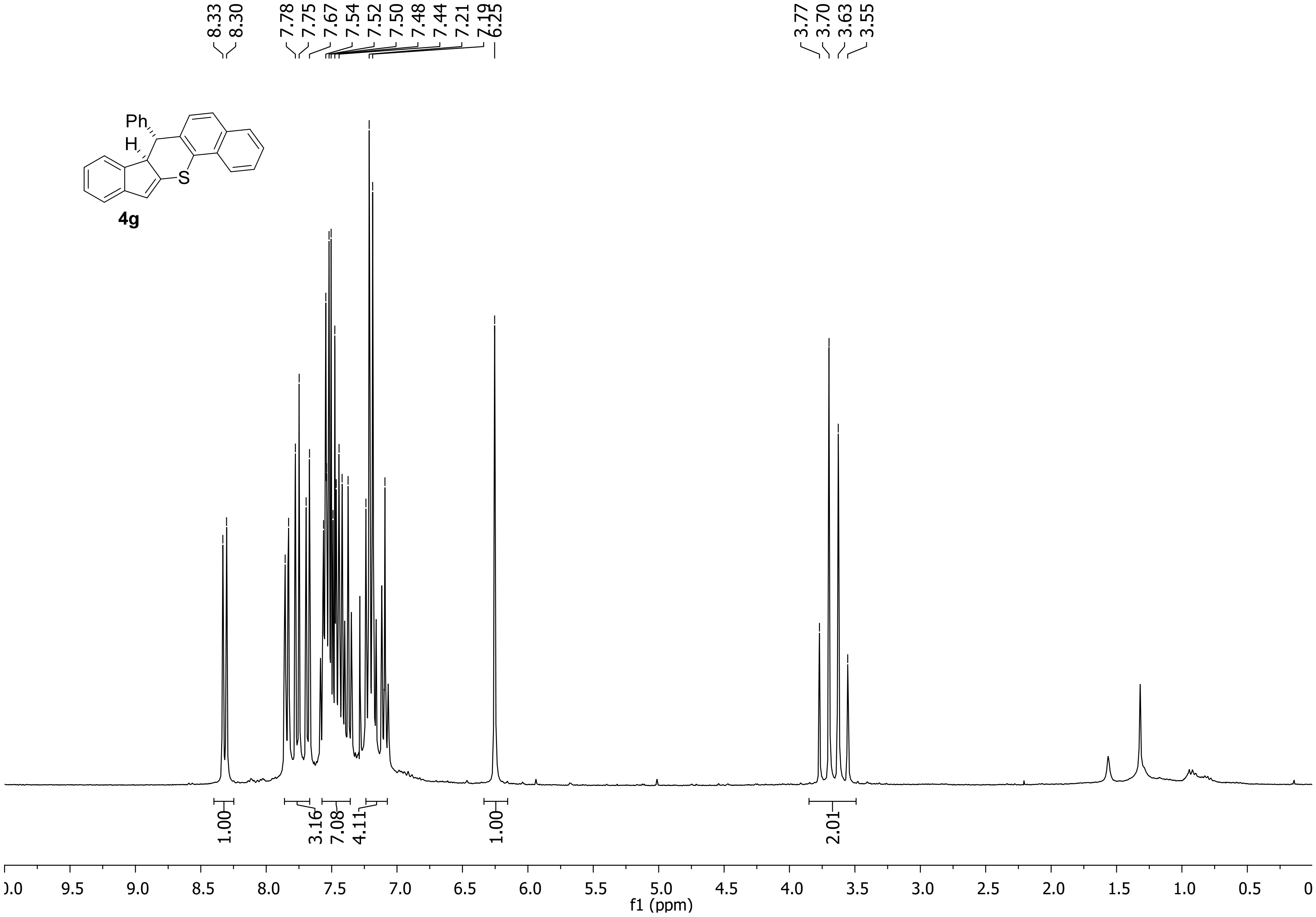

^{13}C NMR (75.4 MHz, $CDCl_3$)

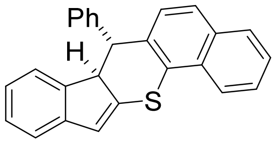

4g

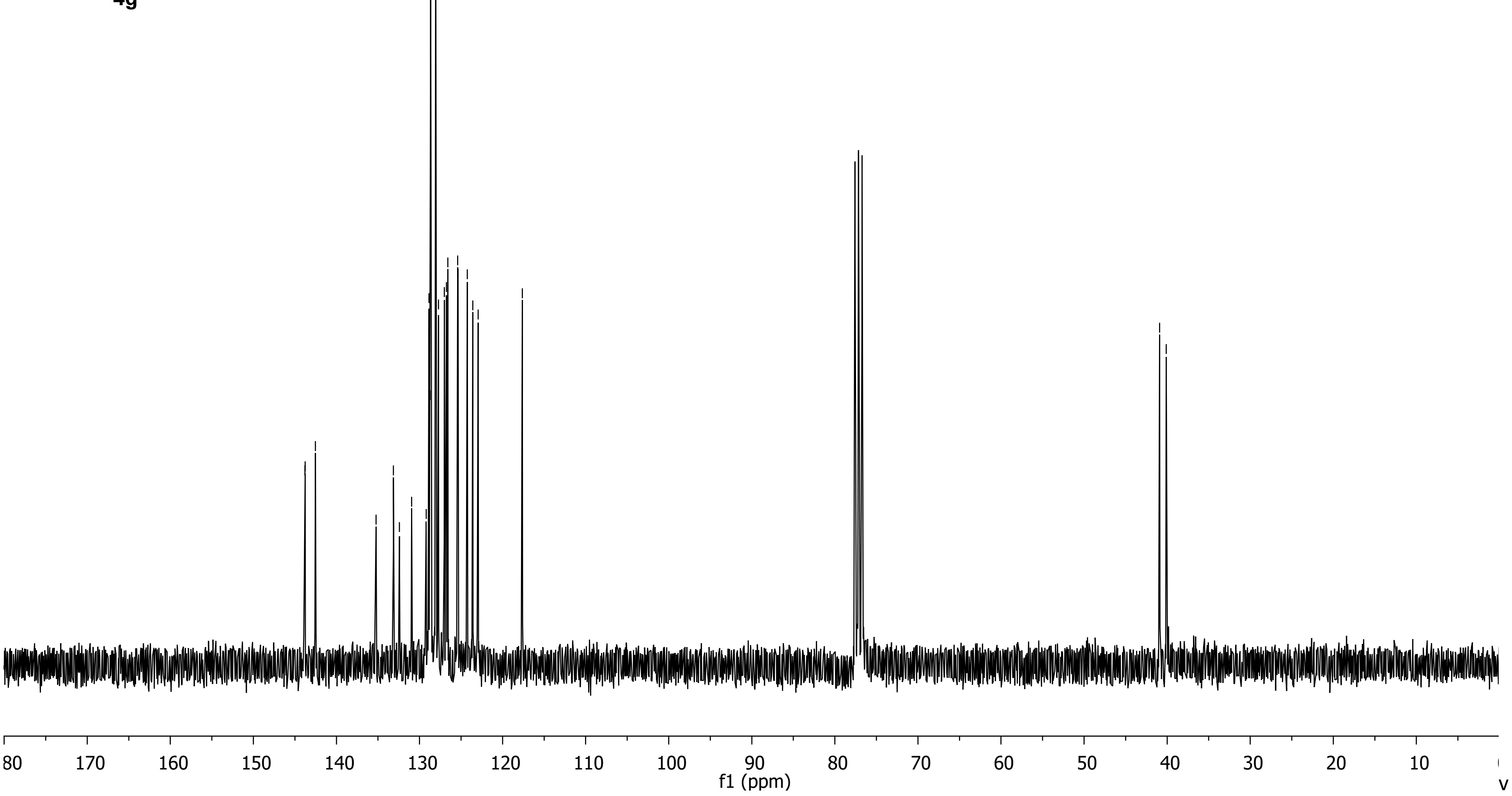

^{1}H NMR (300 MHz, $CDCl_3$)

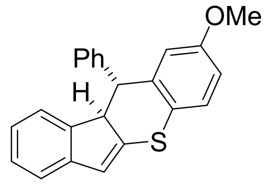

4h

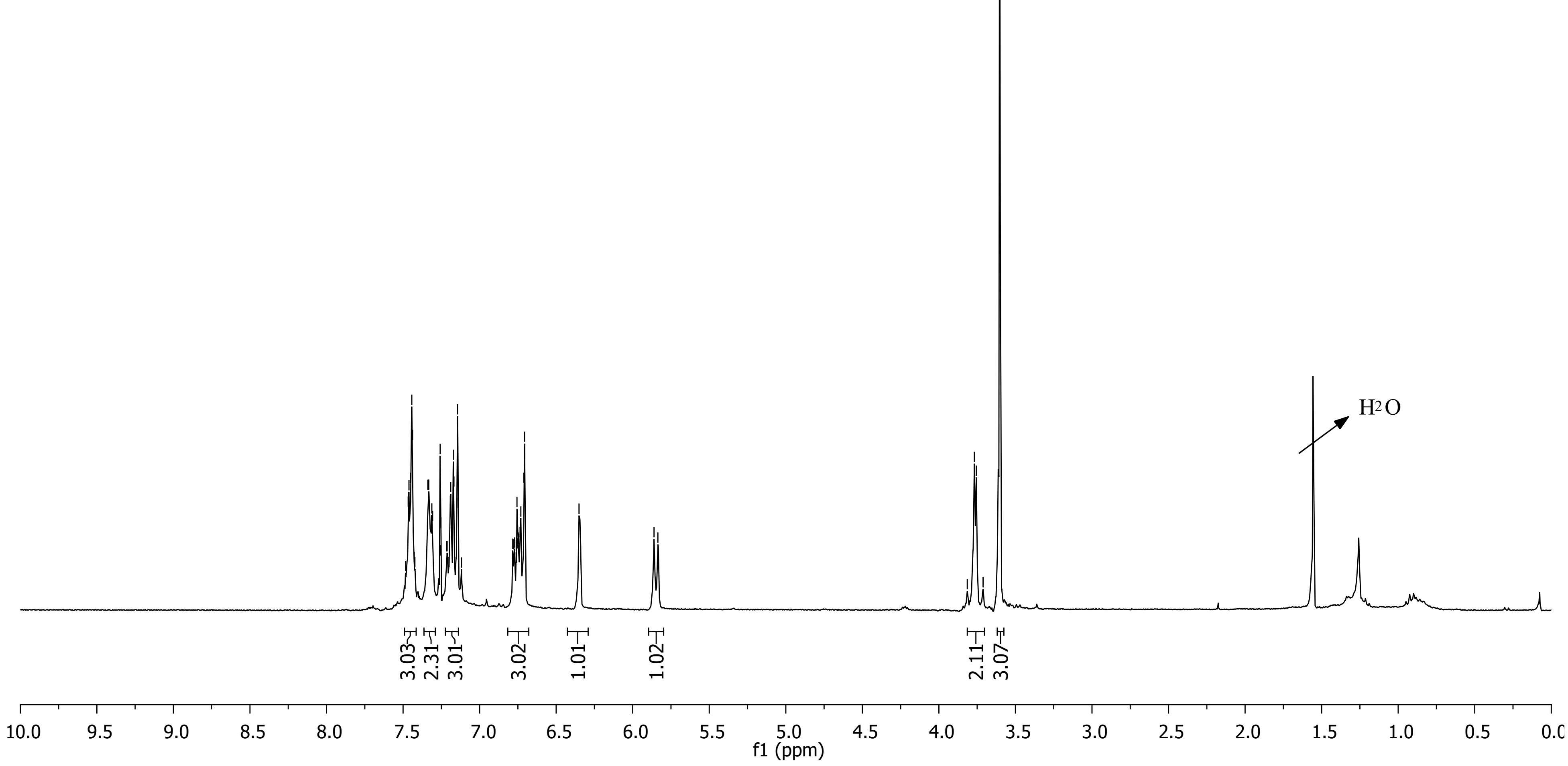

^{13}C NMR (75.4 MHz, $CDCl_3$)

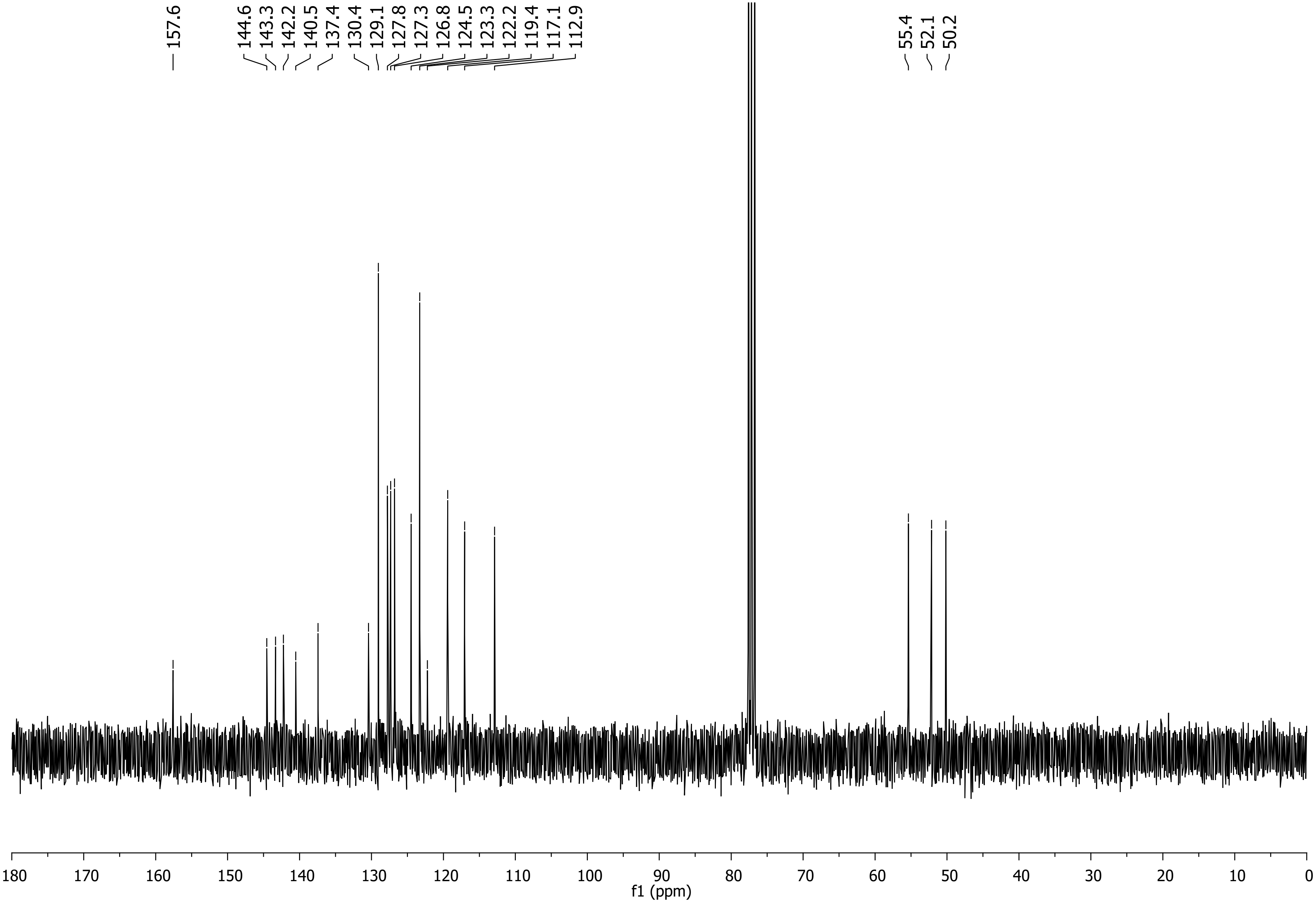

^{1}H NMR (300 MHz, $CDCl_3$)

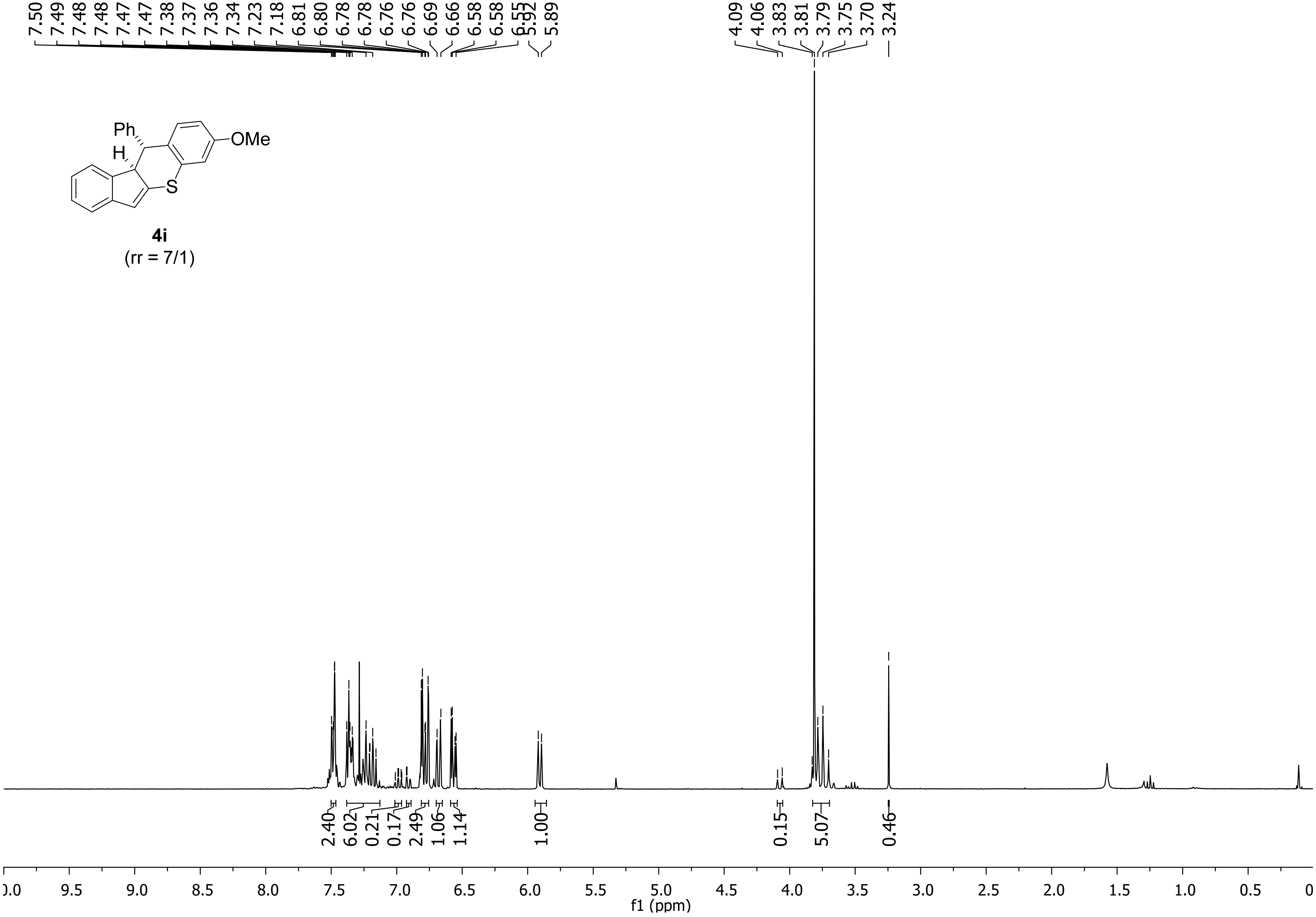

^{13}C NMR (75.4 MHz, $CDCl_3$)

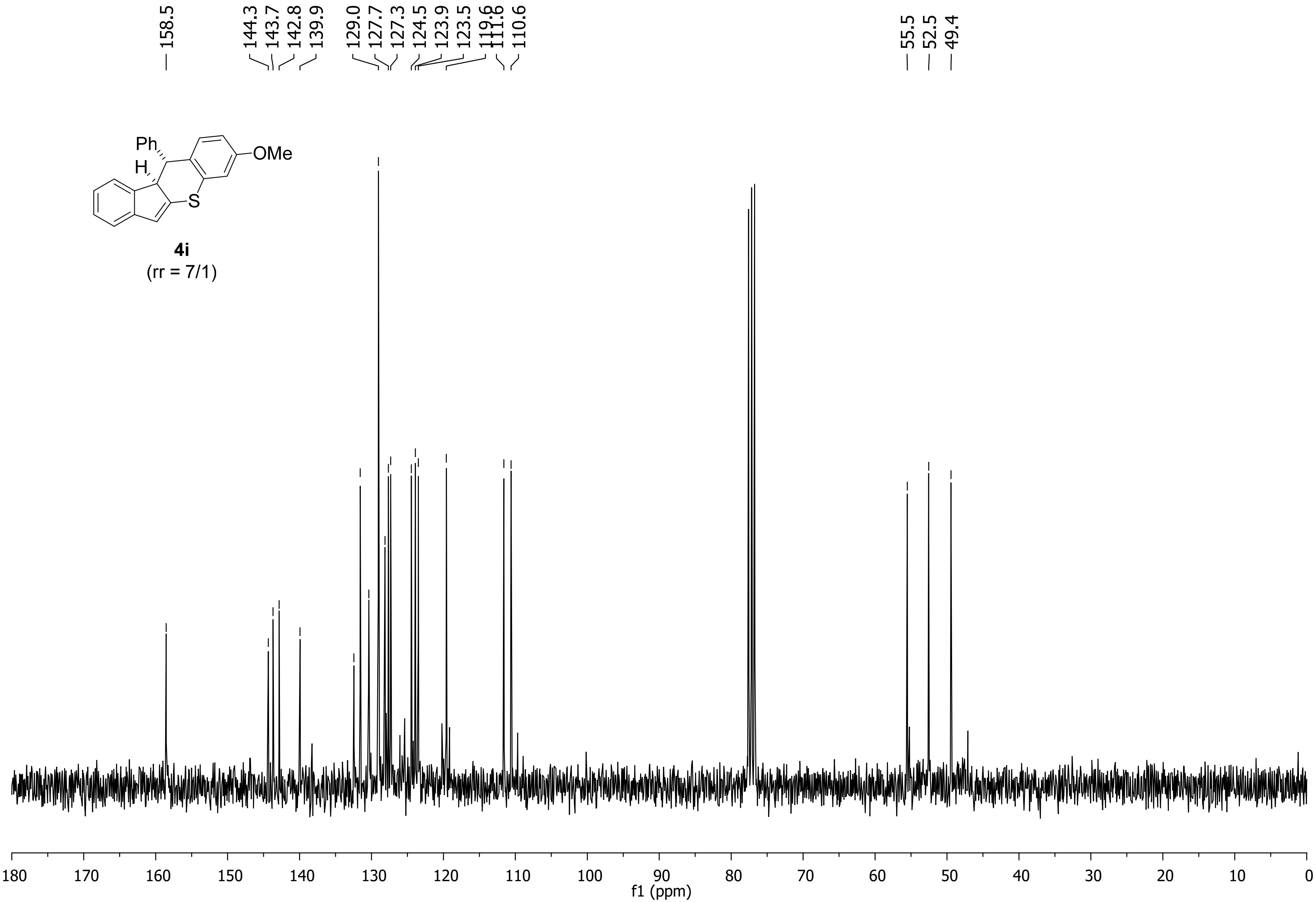

^1H NMR (300 MHz, $CDCl_3$)

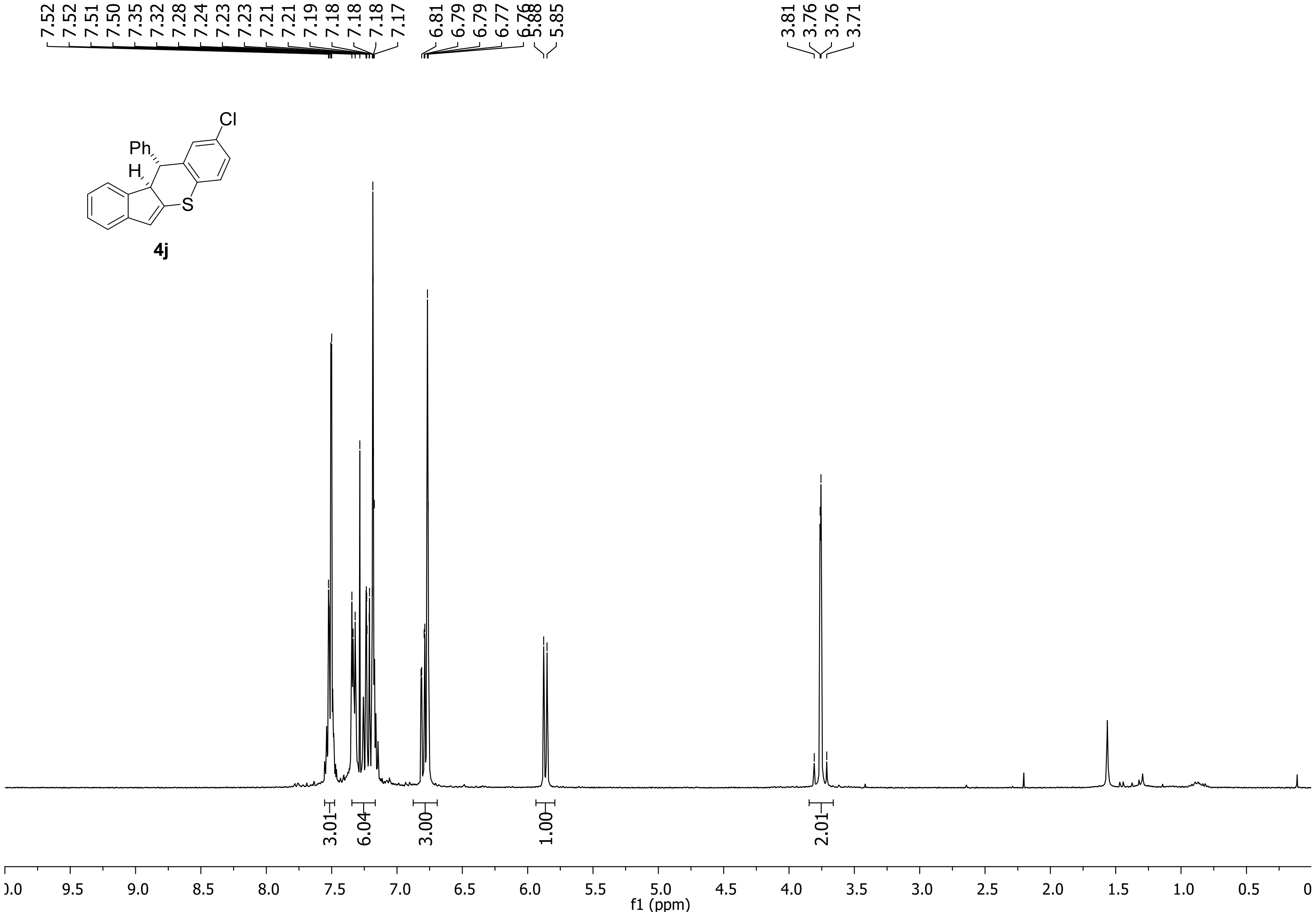

4j

^{13}C NMR (75.4 MHz, $CDCl_3$)

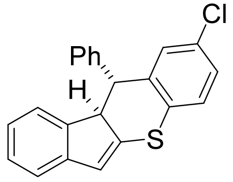

4j

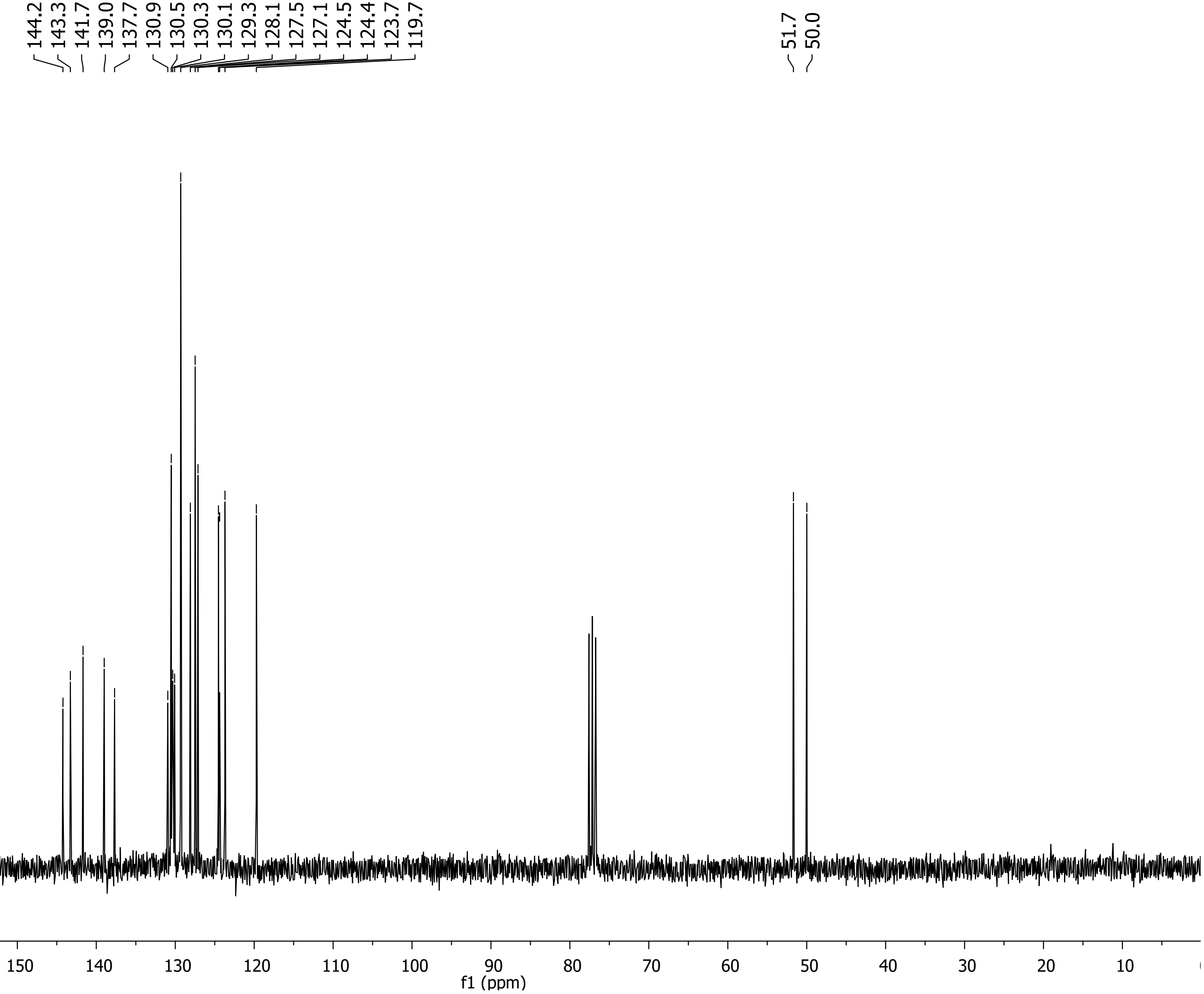

^{1}H NMR (300 MHz, $CDCl_3$)

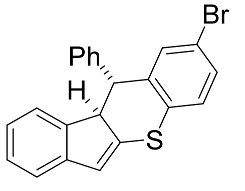

4k

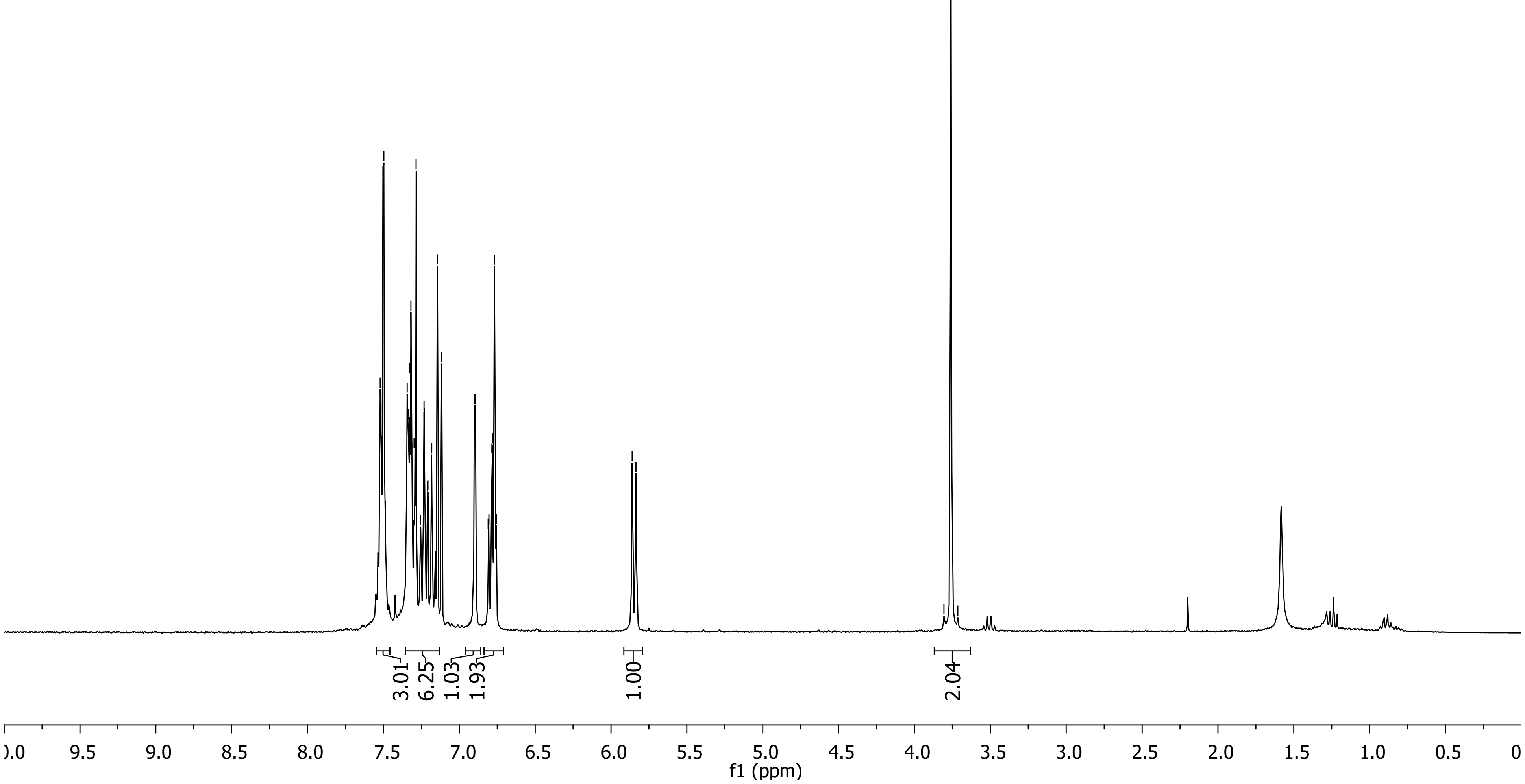

^{13}C NMR (75.4 MHz, $CDCl_3$)

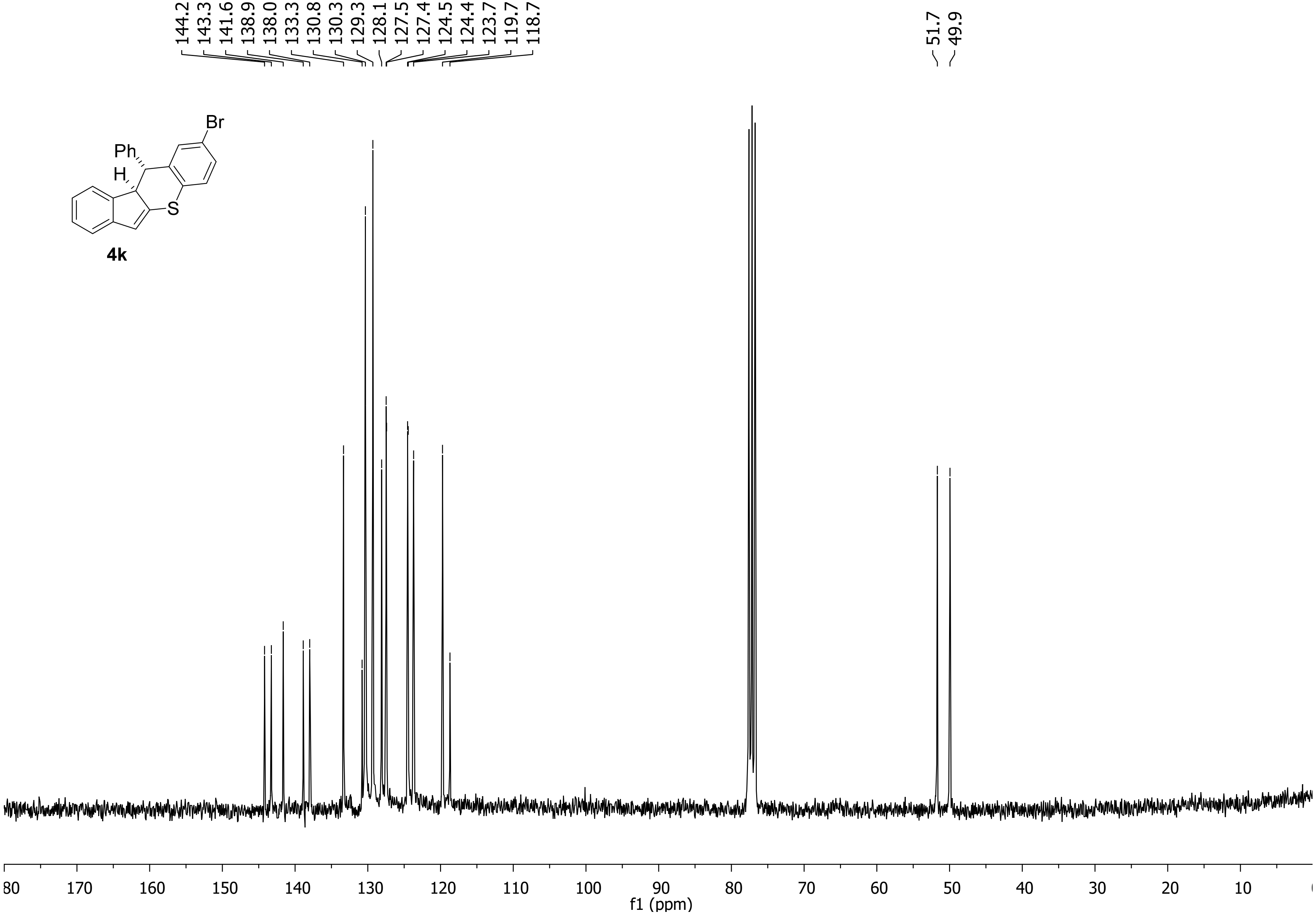

^{1}H NMR (300 MHz, CDCl$_3$)

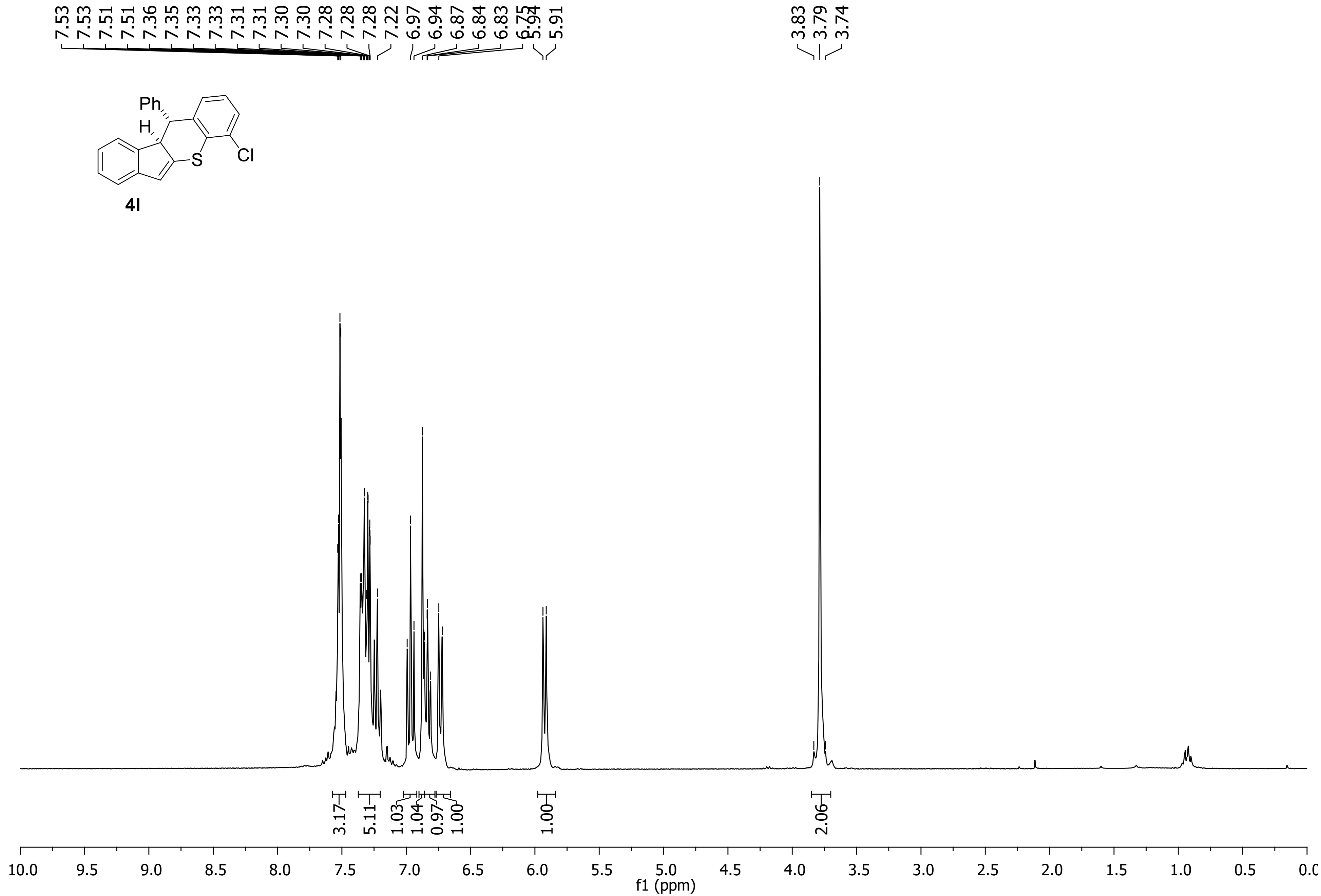

^{13}C NMR (75.4 MHz, $CDCl_3$)

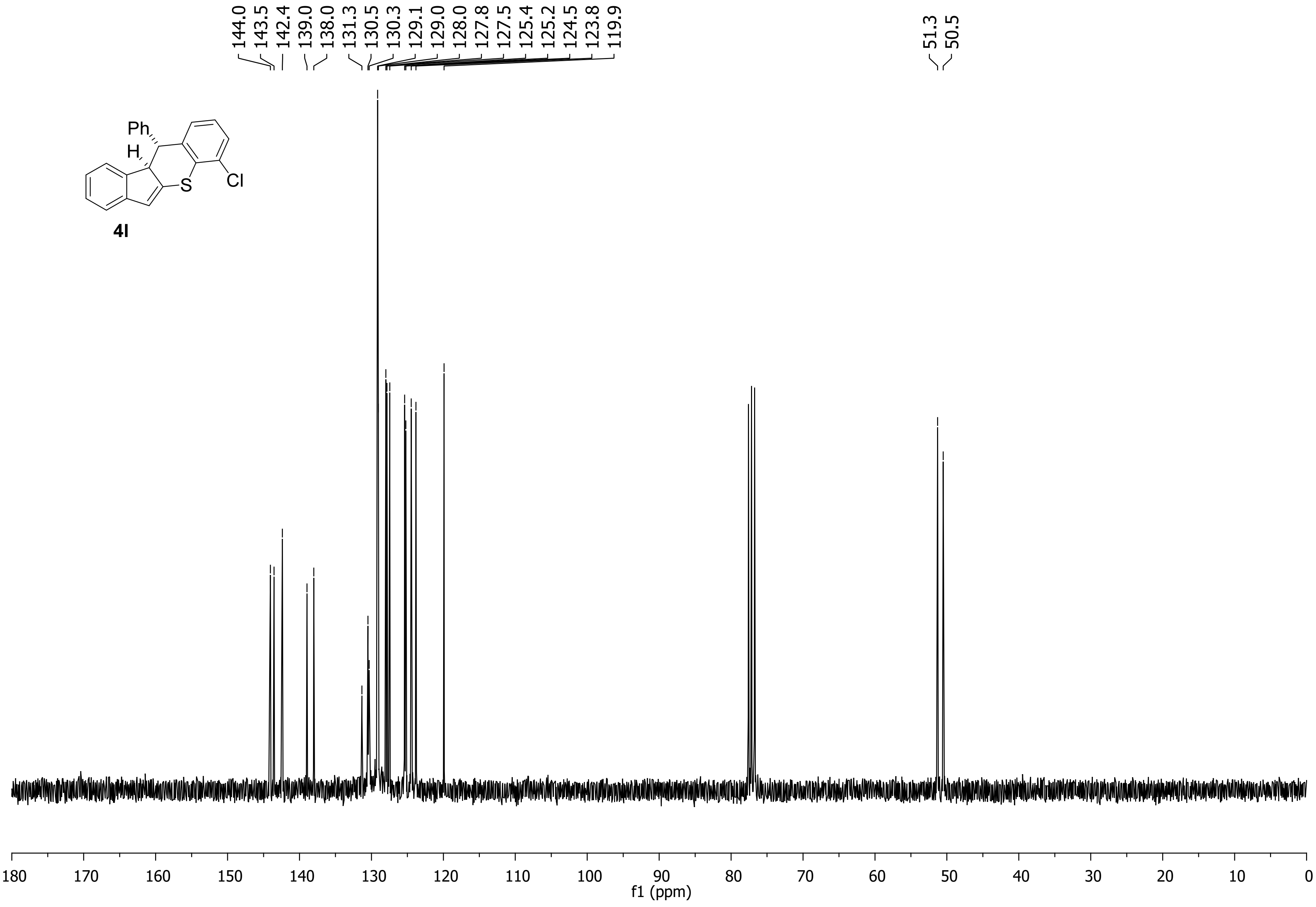

4l

^1H NMR (300 MHz, $CDCl_3$)

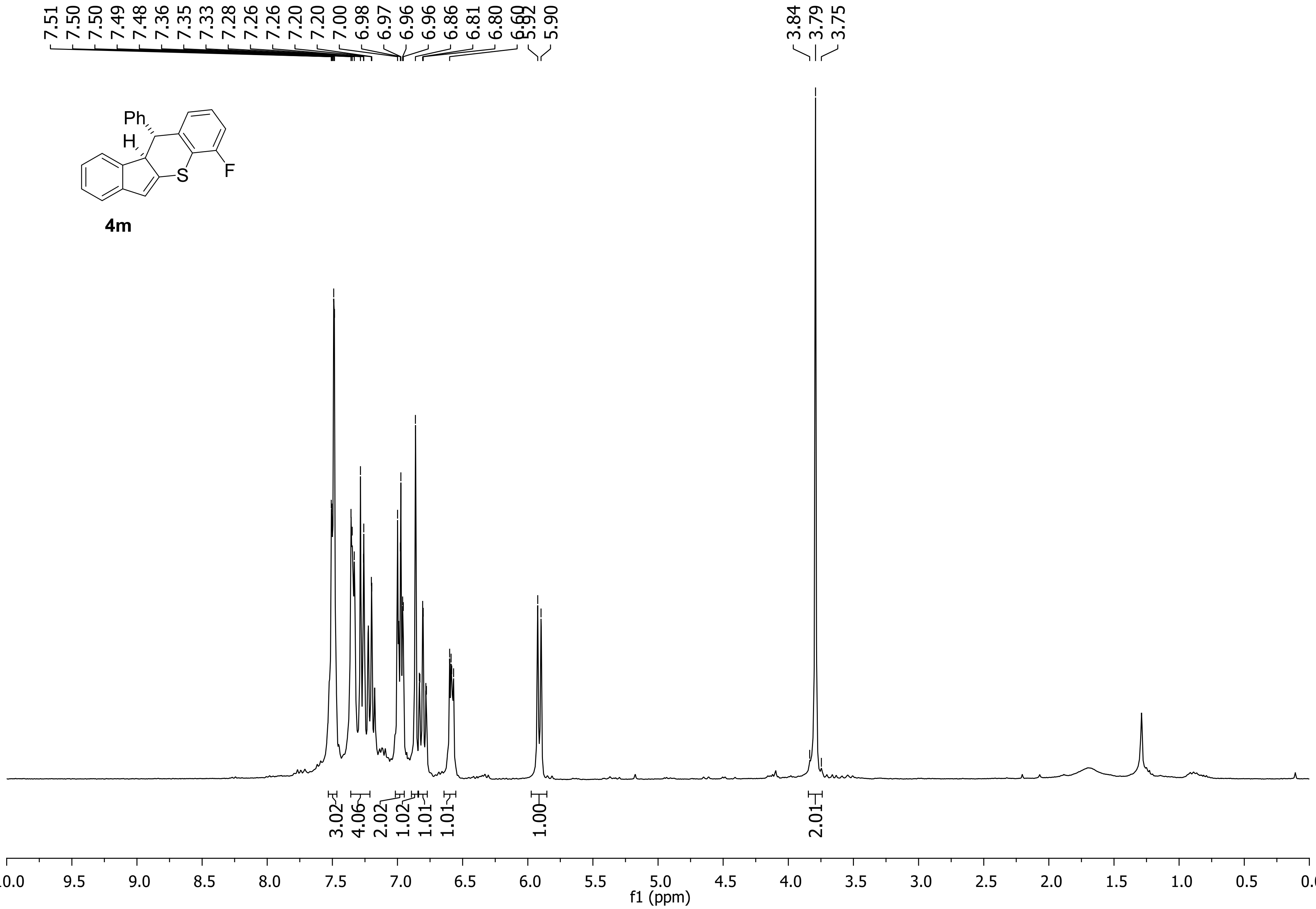

4m

^{13}C NMR (75.4 MHz, $CDCl_3$)

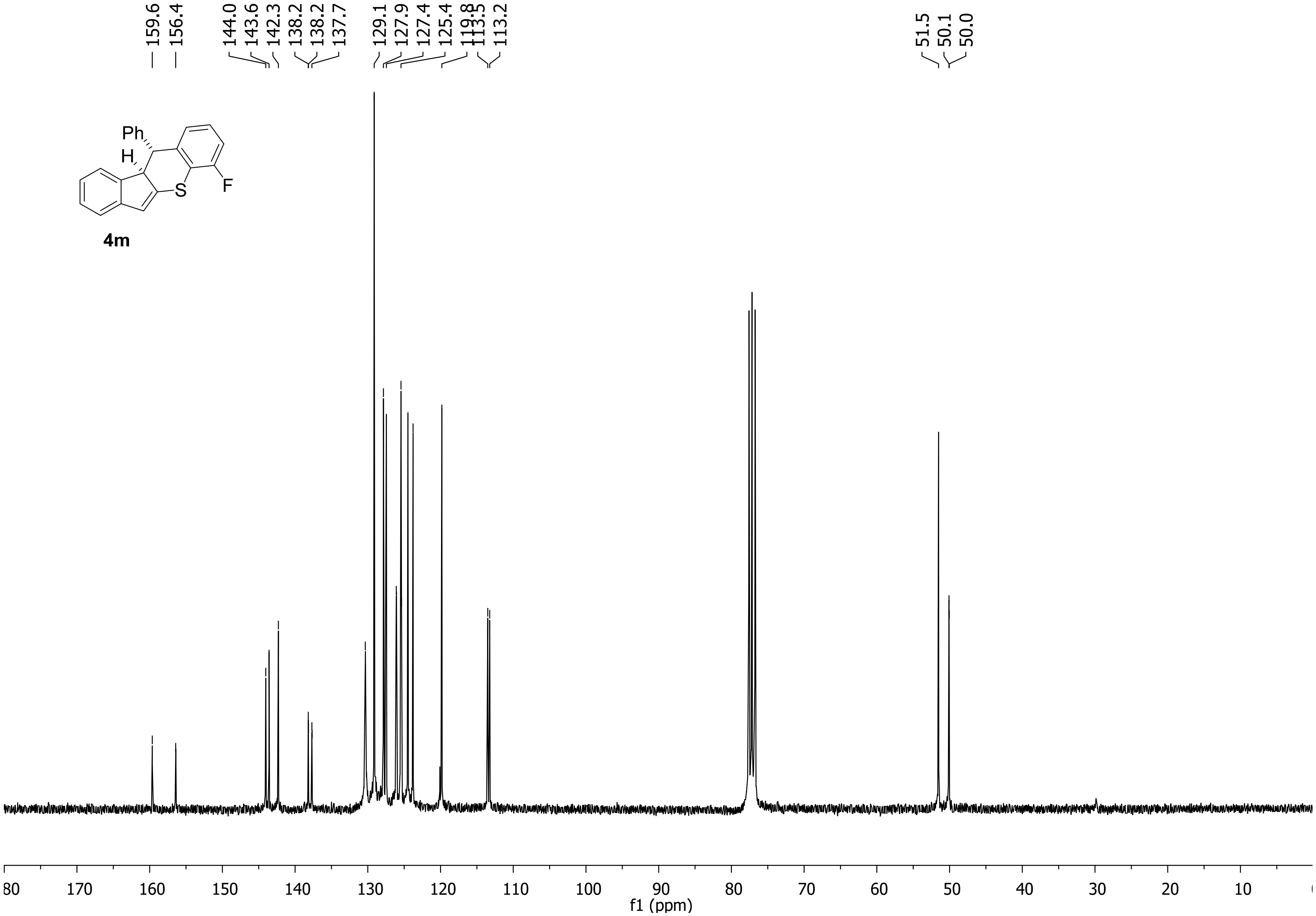

^{1}H NMR (300 MHz, $CDCl_3$)

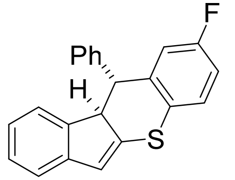

4n

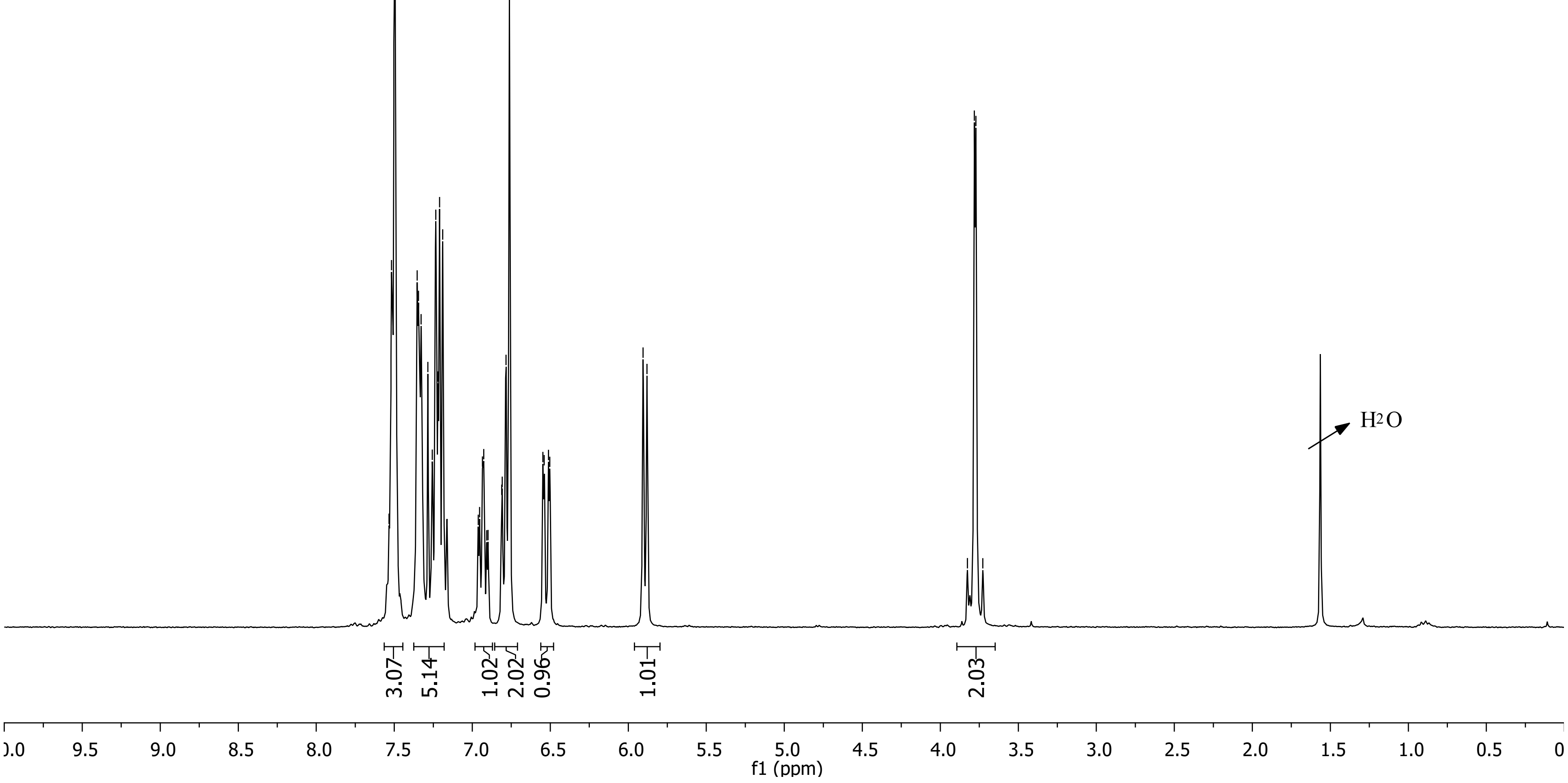

^{13}C NMR (75.4 MHz, $CDCl_3$)

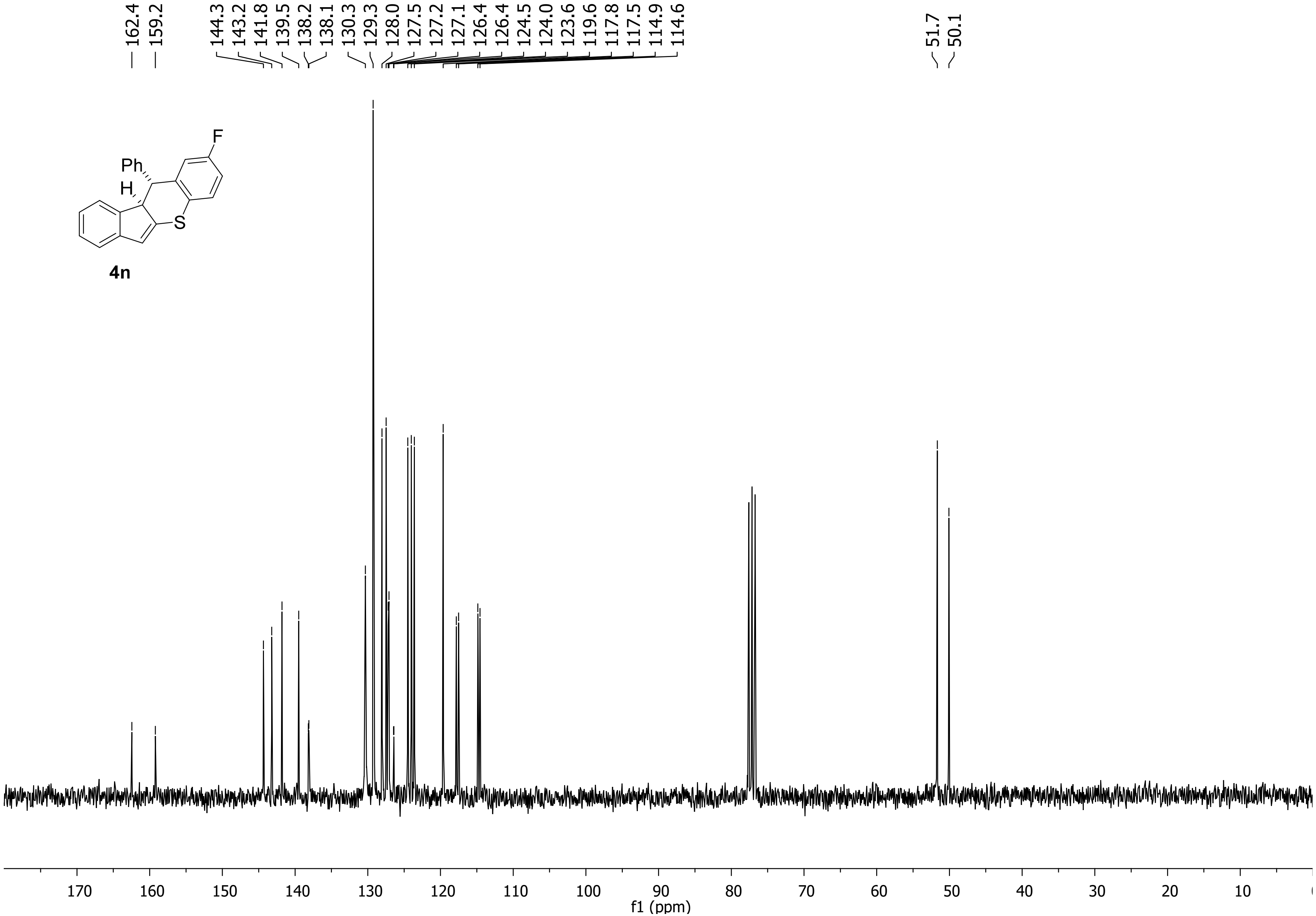

4n

^{1}H NMR (300 MHz, $CDCl_3$)

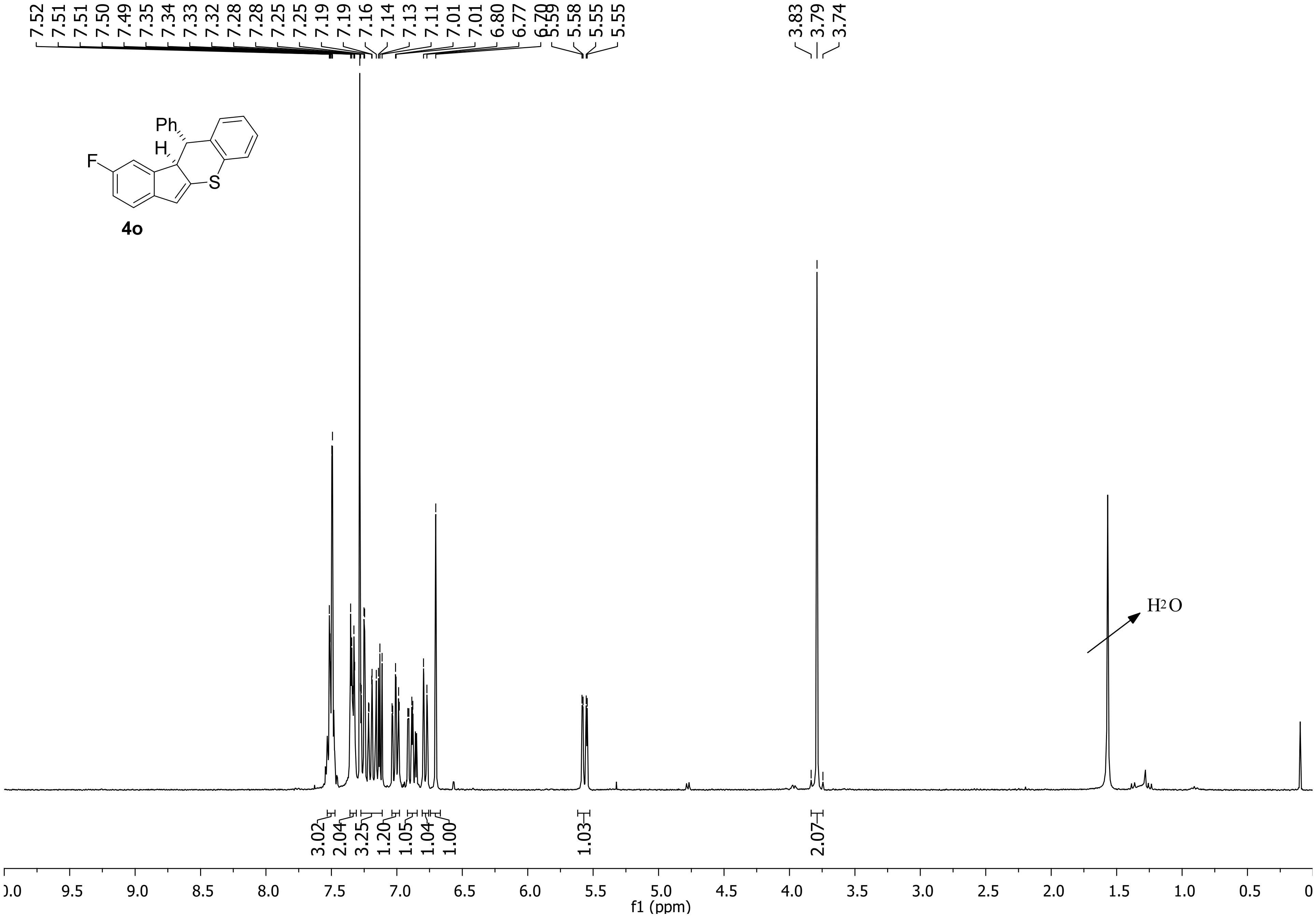

^{13}C NMR (75.4 MHz, $CDCl_3$)

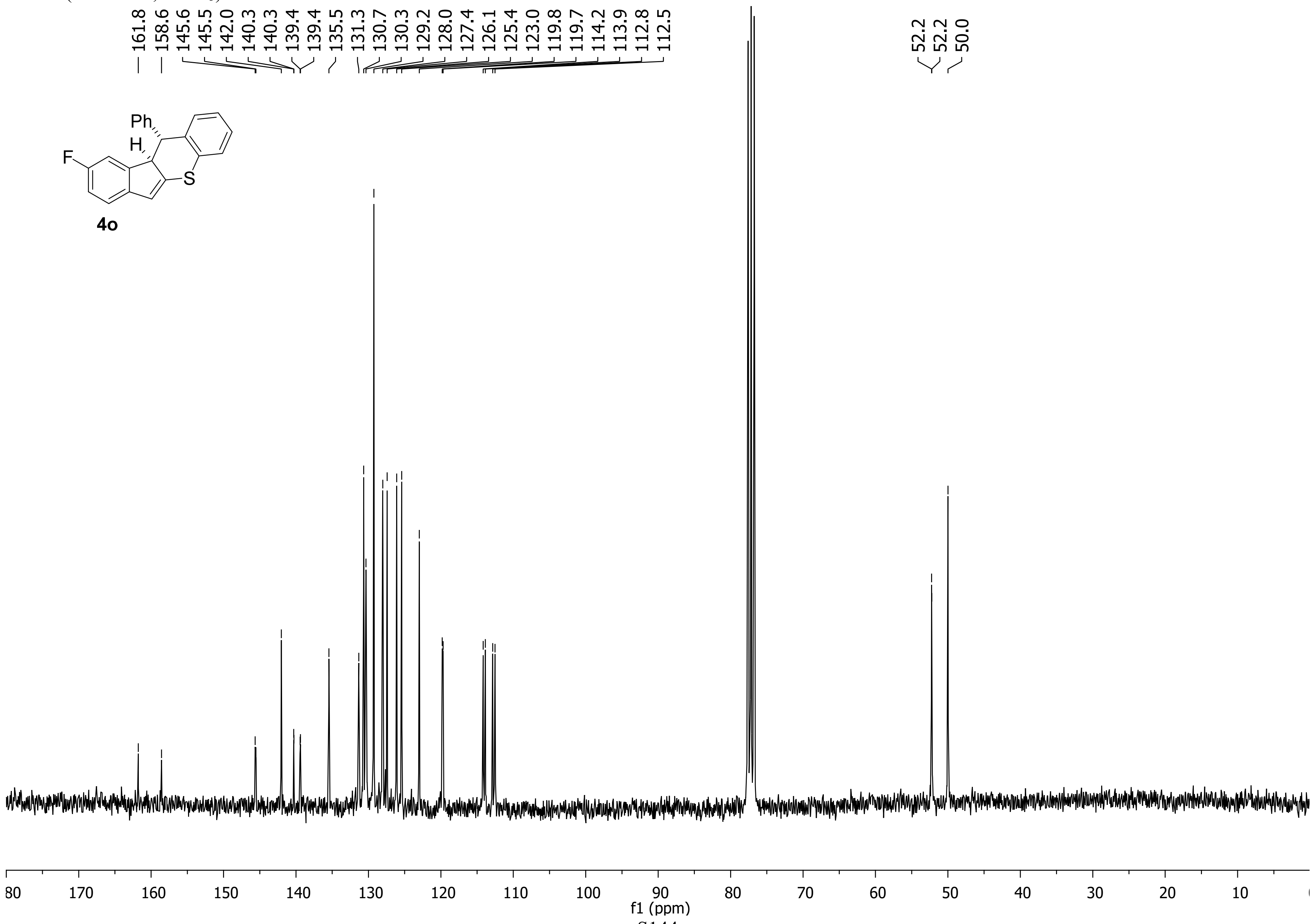

^{1}H NMR (300 MHz, $CDCl_3$)

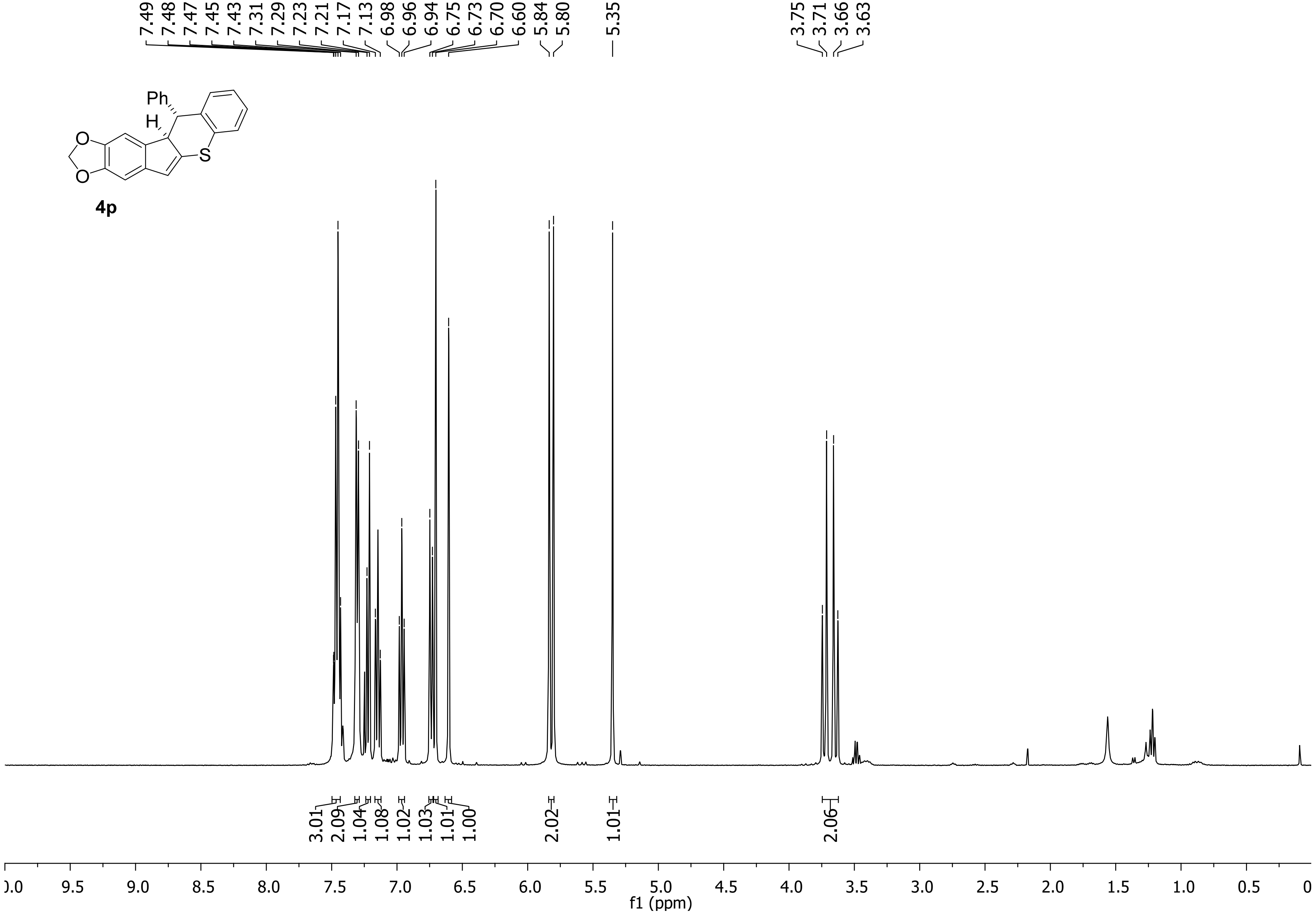

^{13}C NMR (75.4 MHz, $CDCl_3$)

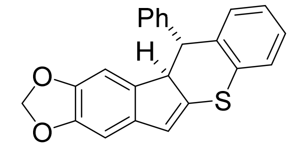

4p

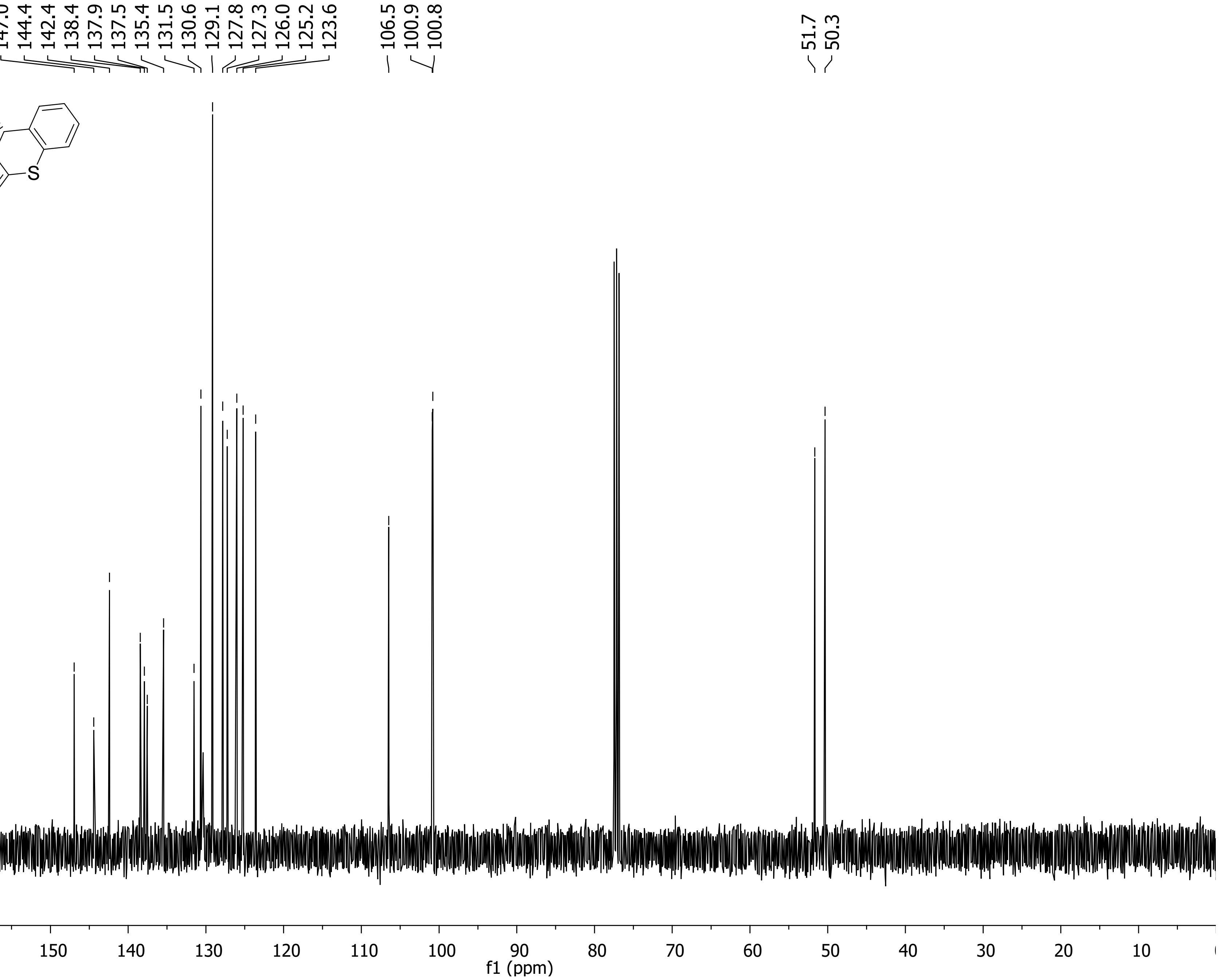

^{1}H NMR (300 MHz, $CDCl_3$)

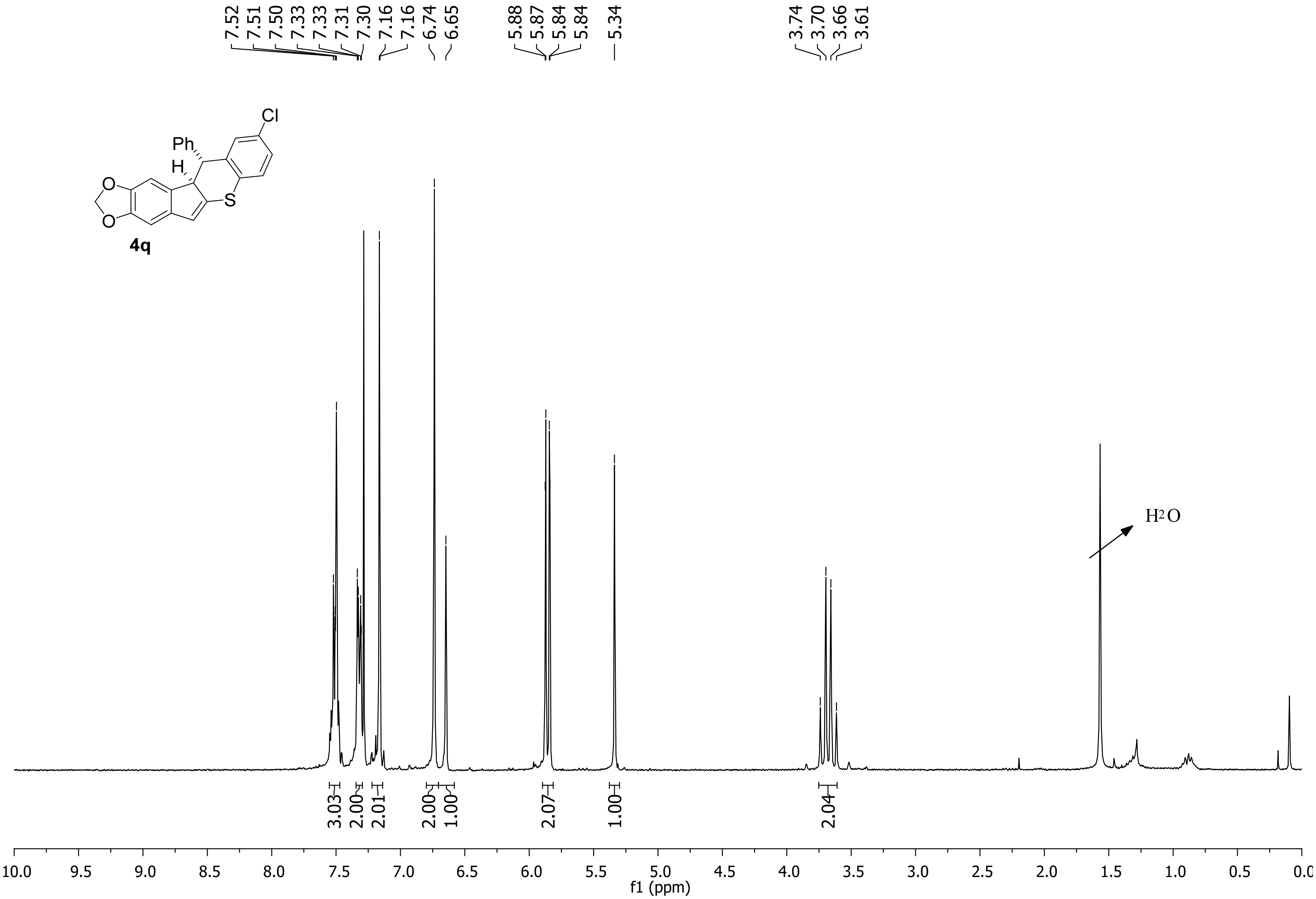

^{13}C NMR (75.4 MHz, $CDCl_3$)

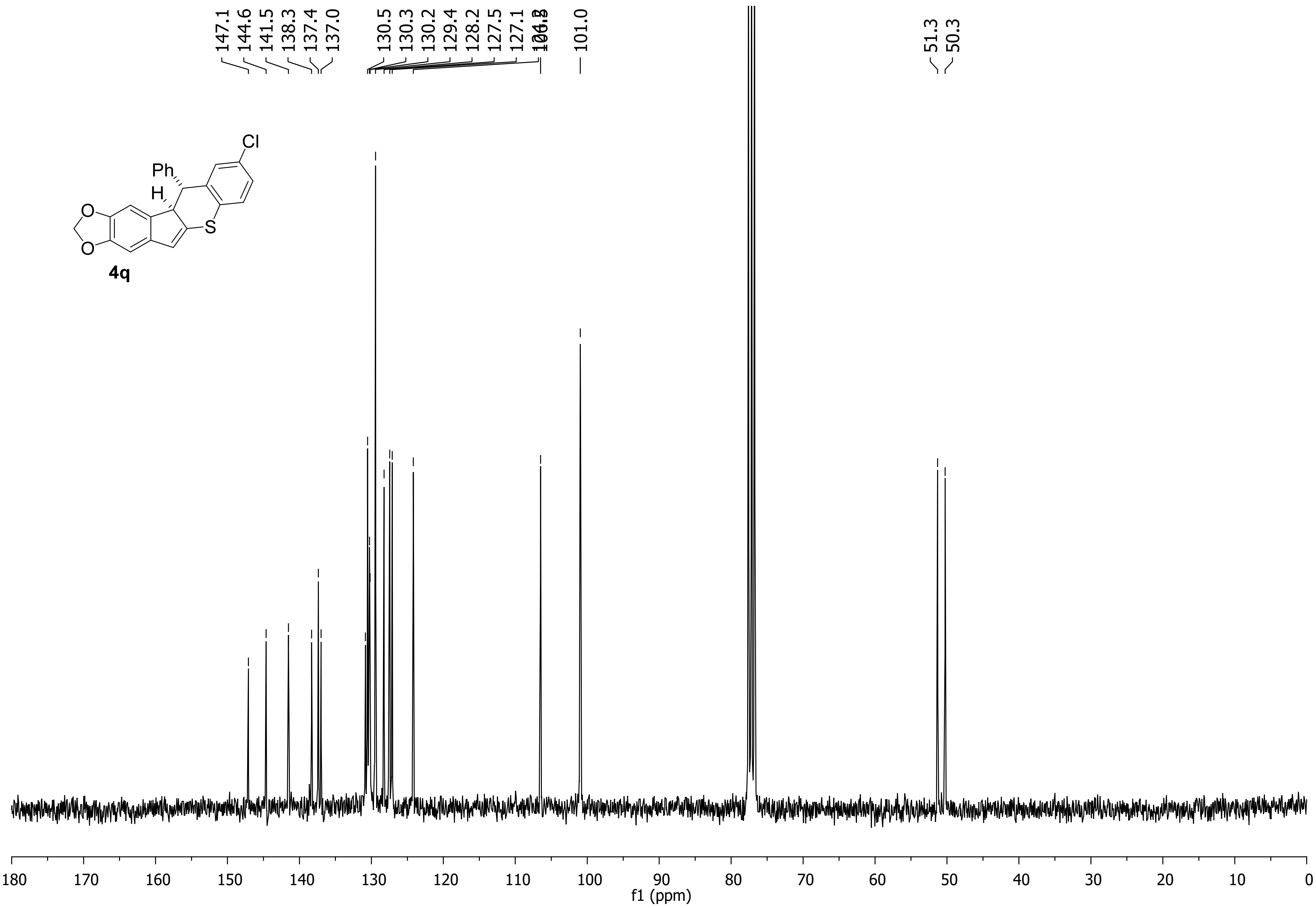

^{1}H NMR (300 MHz, $CDCl_3$)

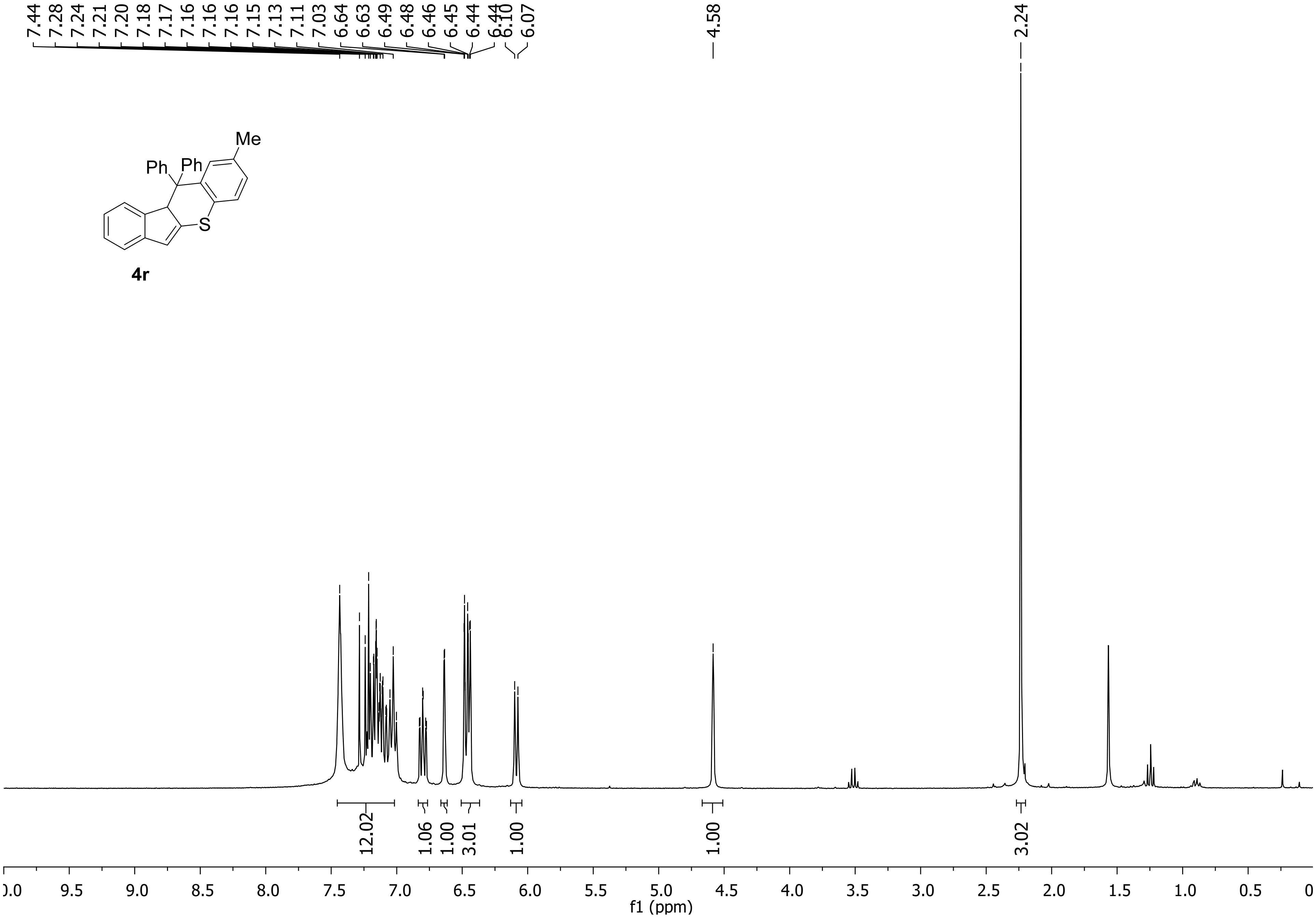

^{13}C NMR (75.4 MHz, $CDCl_3$)

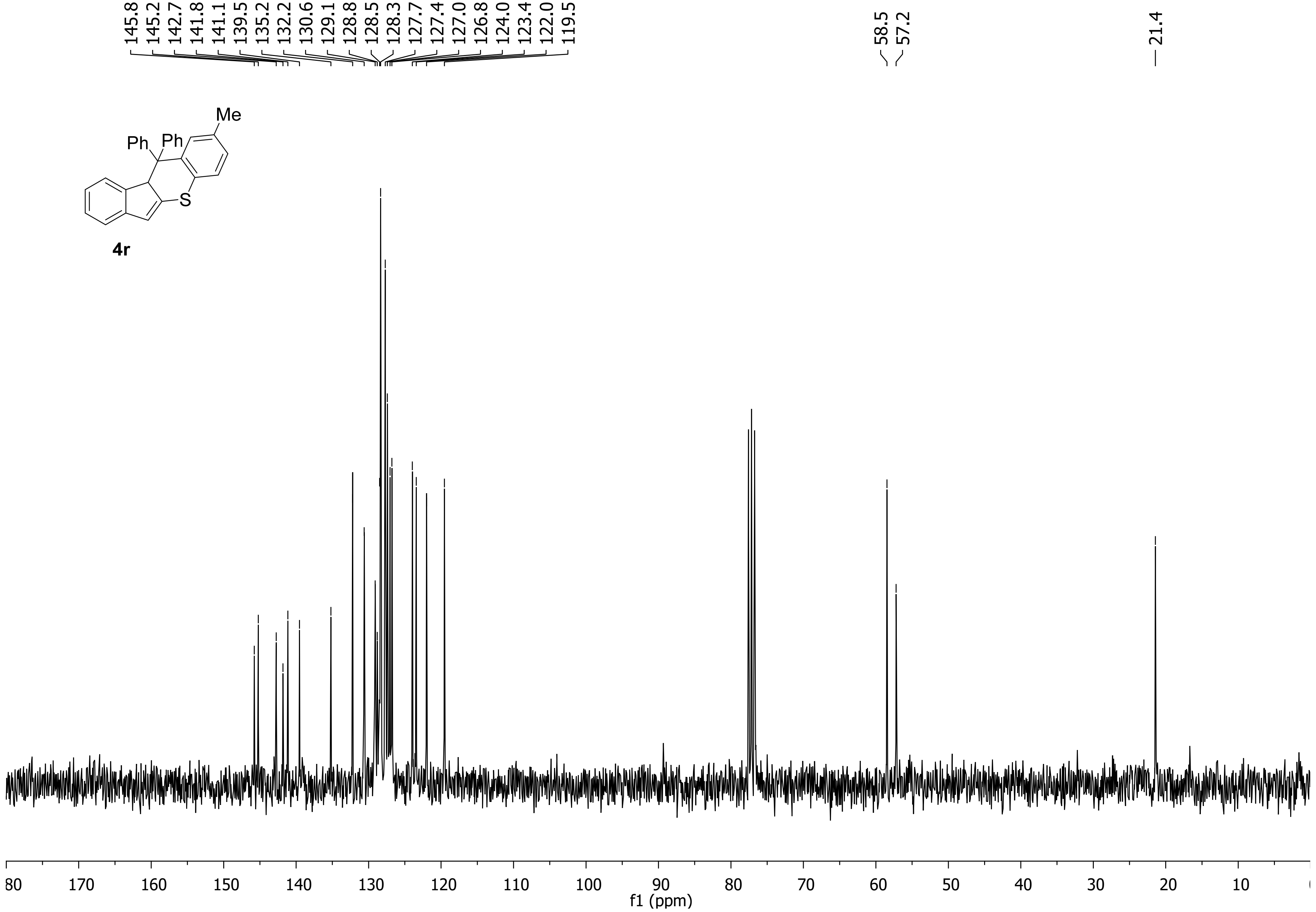

^{1}H NMR (300 MHz, $CDCl_3$)

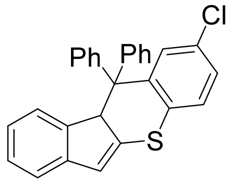

4s

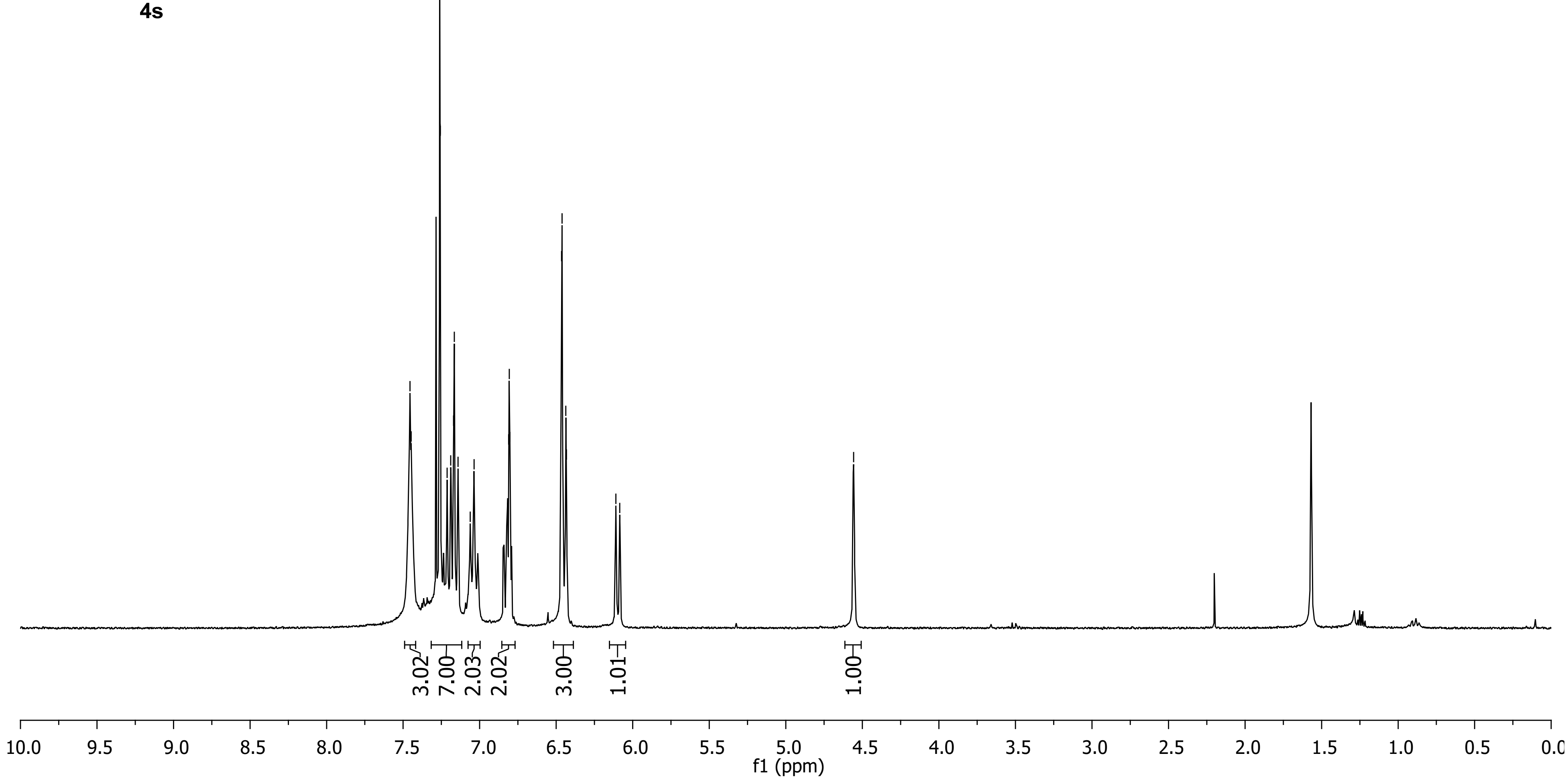

^{13}C NMR (75.4 MHz, $CDCl_3$)

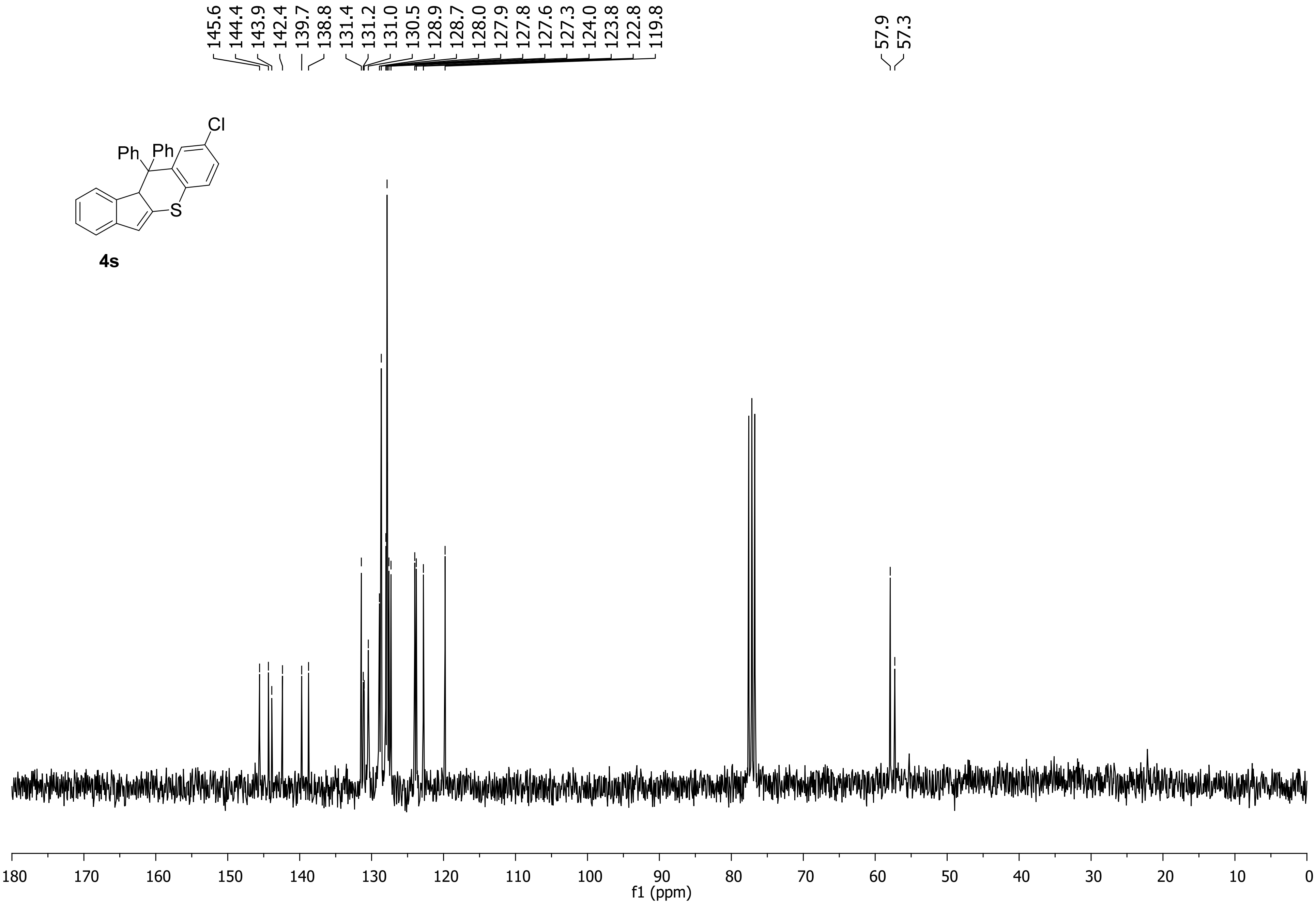

^{1}H NMR (300 MHz, $CDCl_3$)

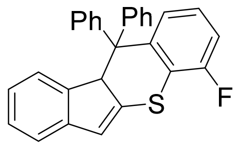

4t/4't = 20/1

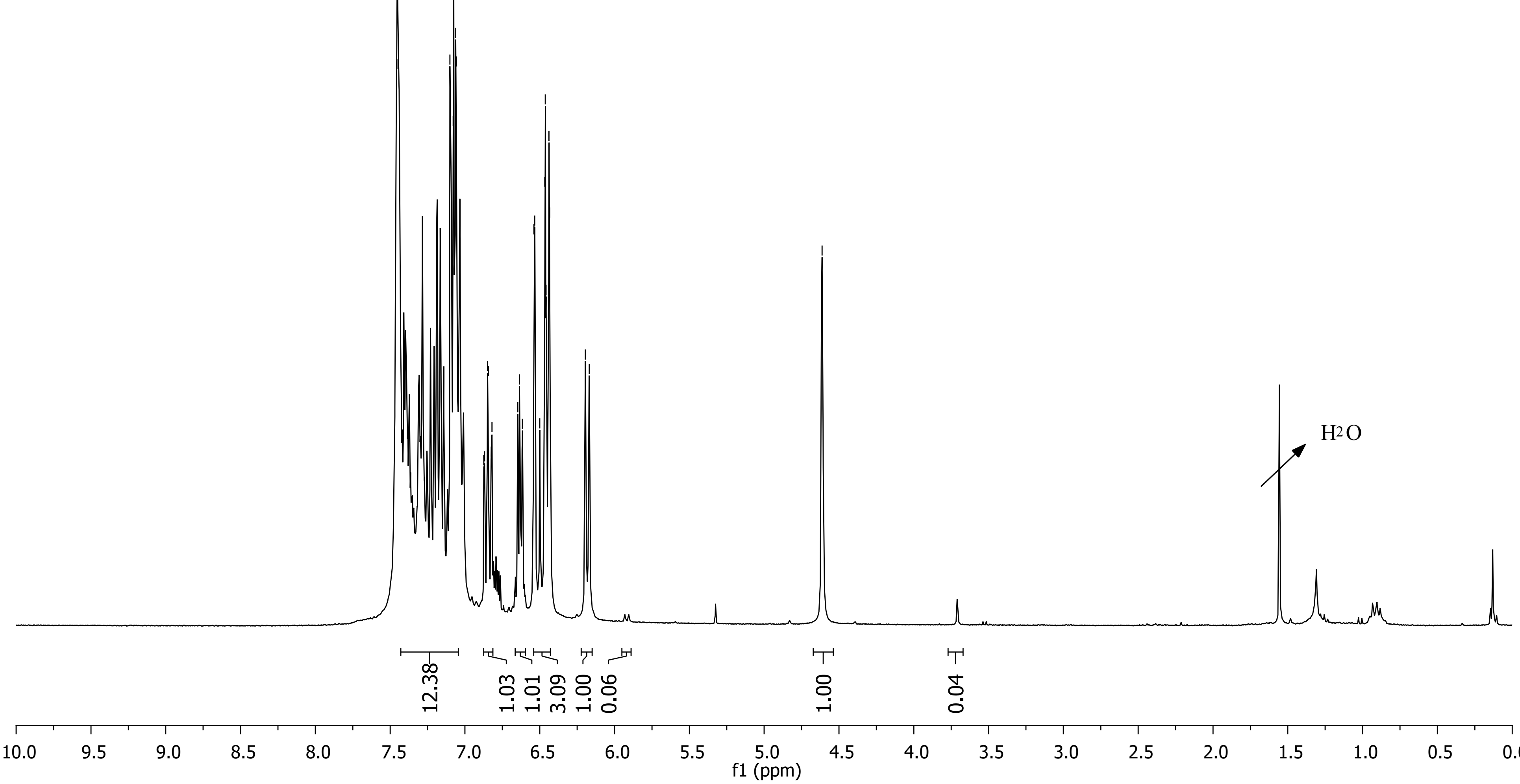

^{13}C NMR (75.4 MHz, $CDCl_3$)

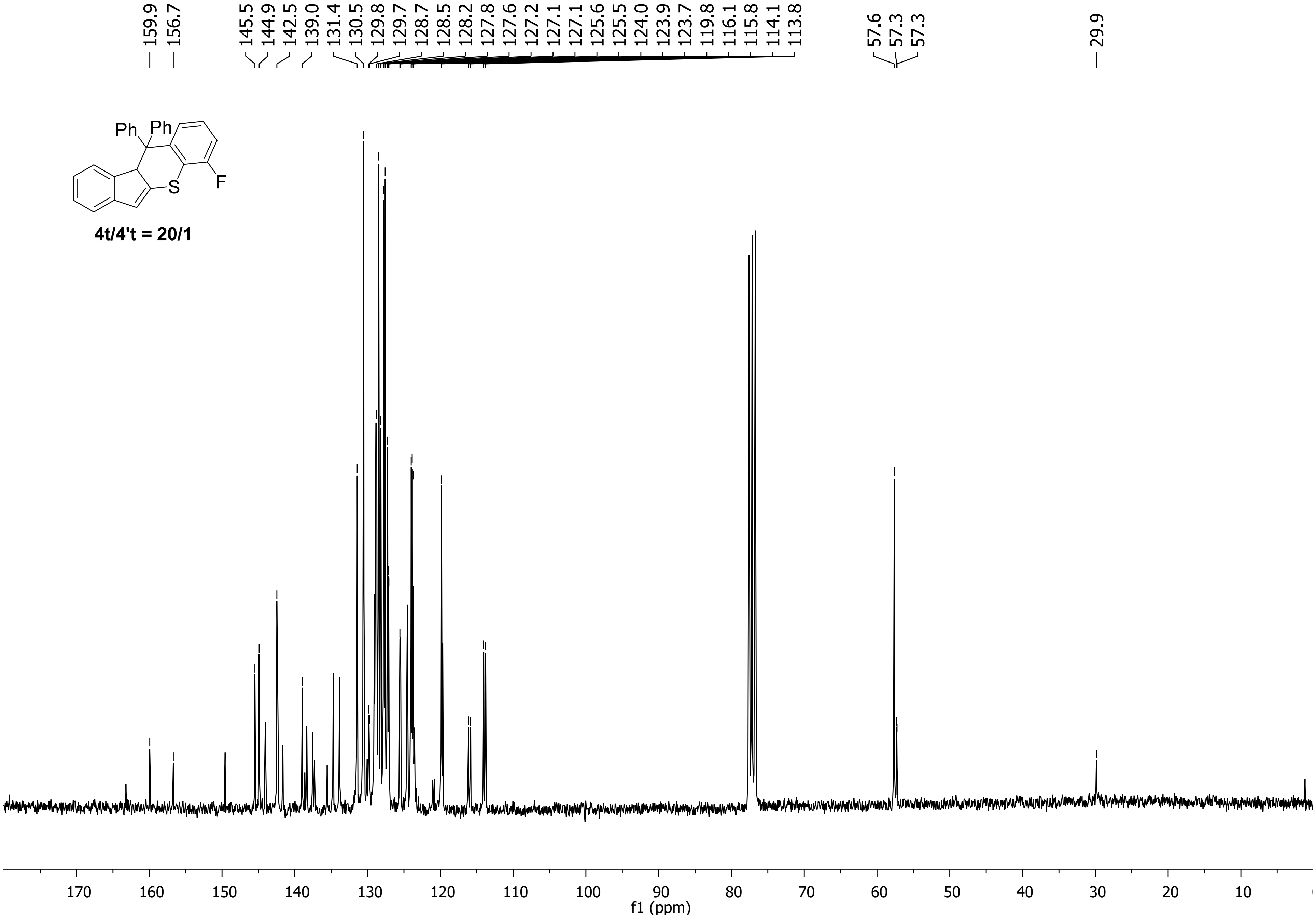

^{1}H NMR (300 MHz, $CDCl_3$)

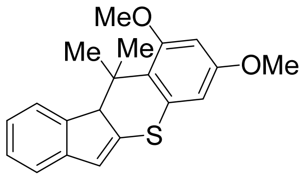

4v/7v = ~2/1

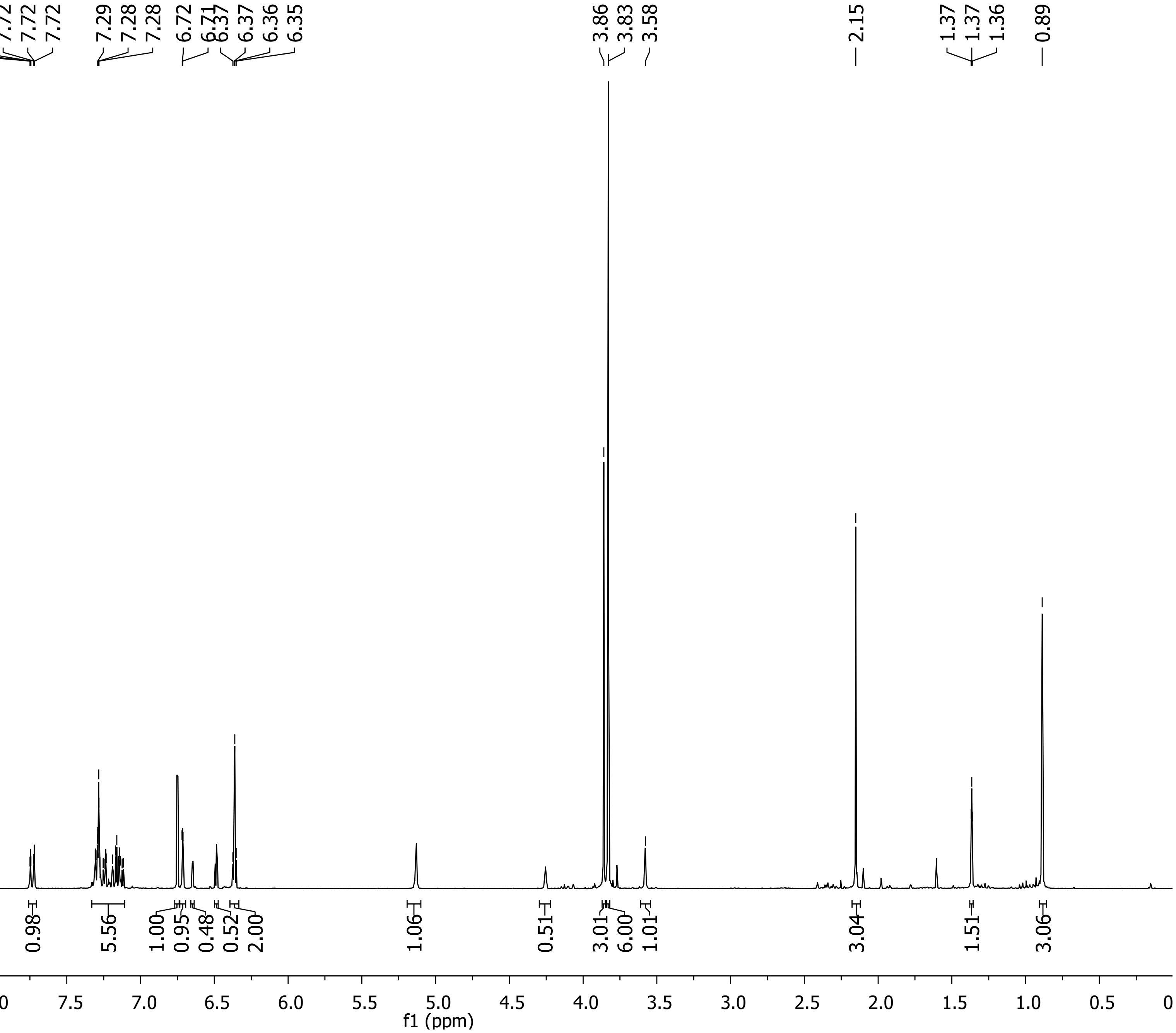

^{13}C NMR (75.4 MHz, $CDCl_3$)

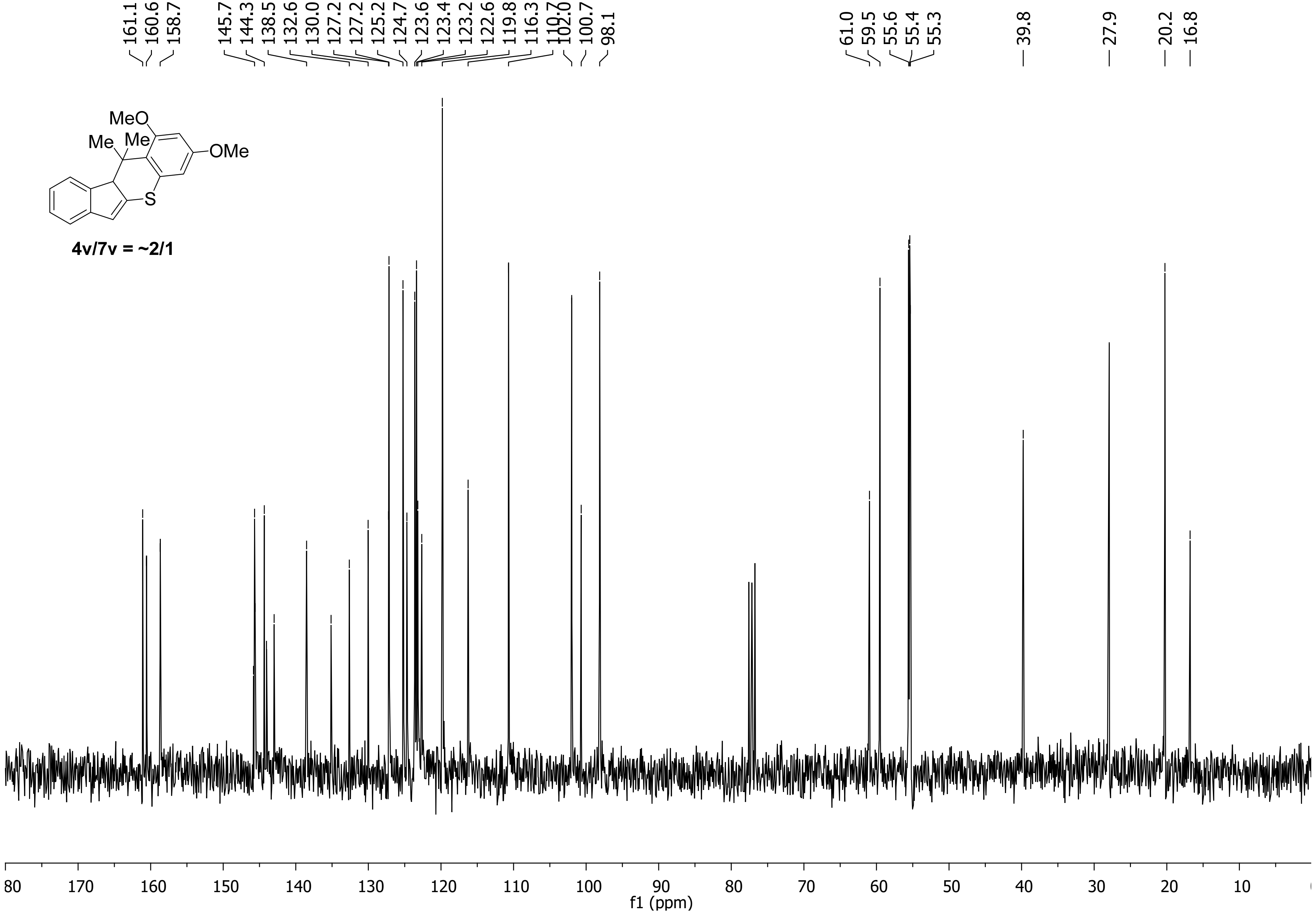

^{1}H NMR (300 MHz, CDCl$_3$)

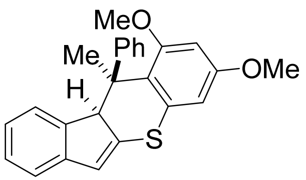

4w

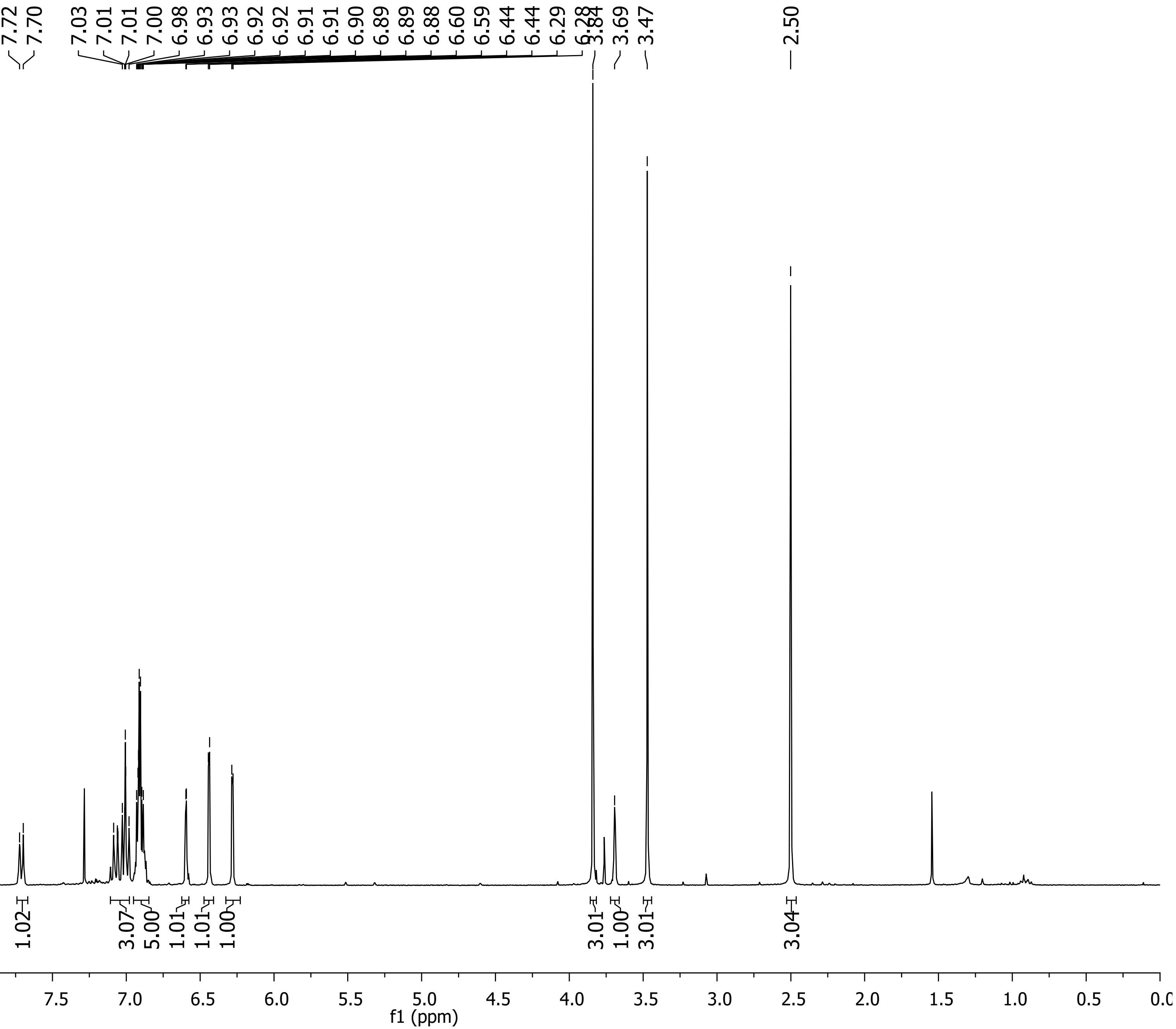

^{13}C NMR (75.4 MHz, $CDCl_3$)

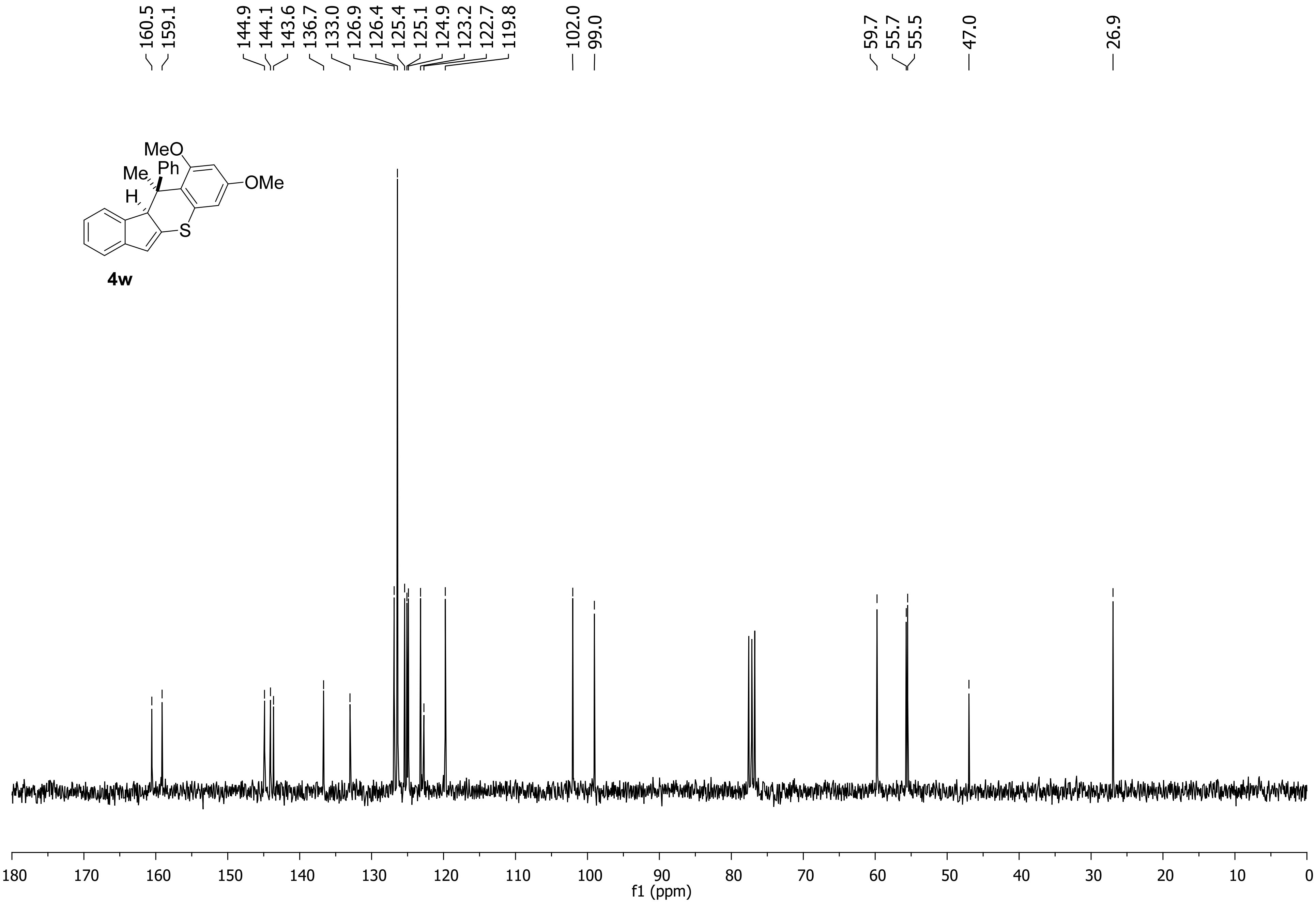

^{1}H NMR (300 MHz, $CDCl_3$)

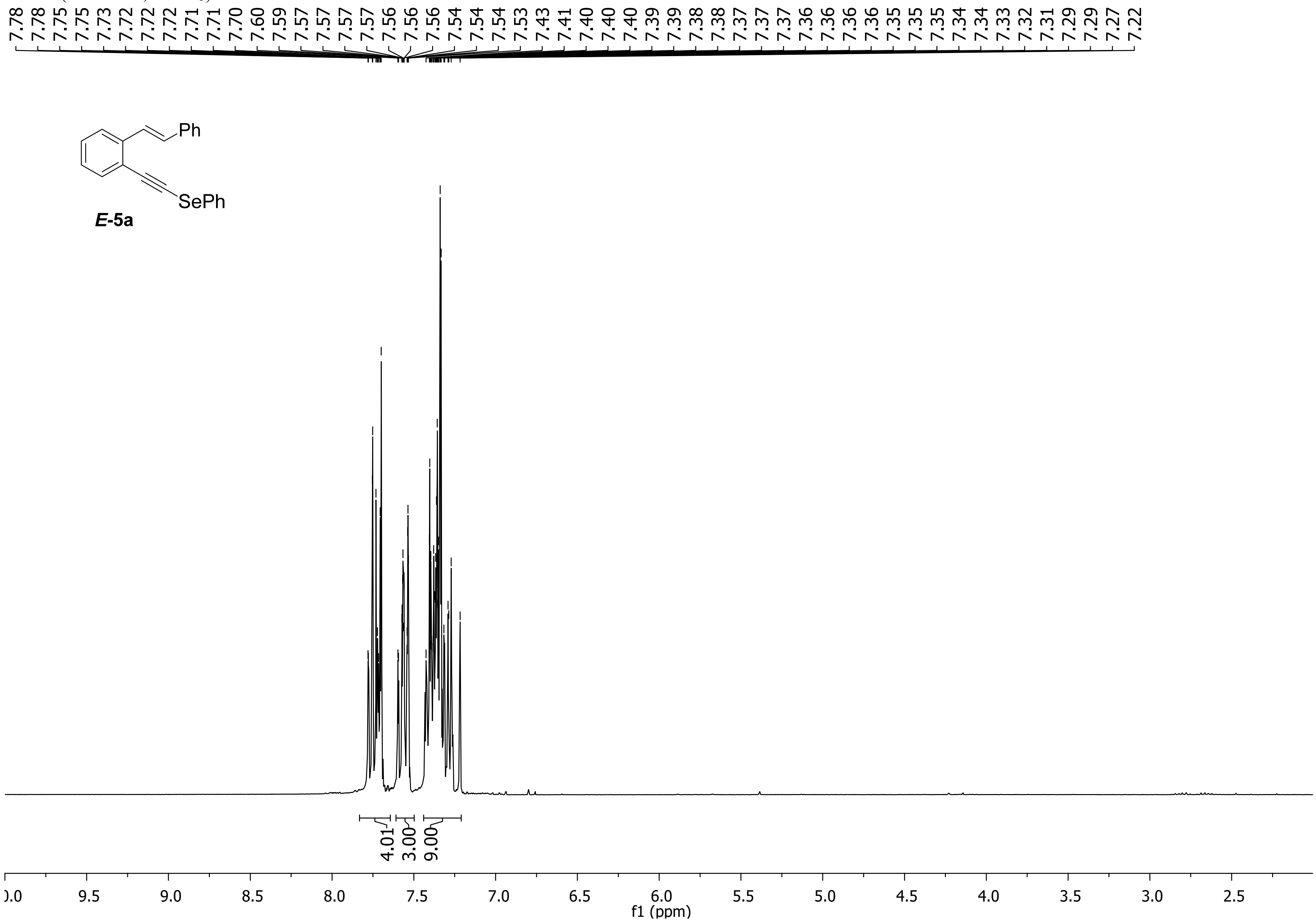

^{13}C NMR (75.4 MHz, $CDCl_3$)

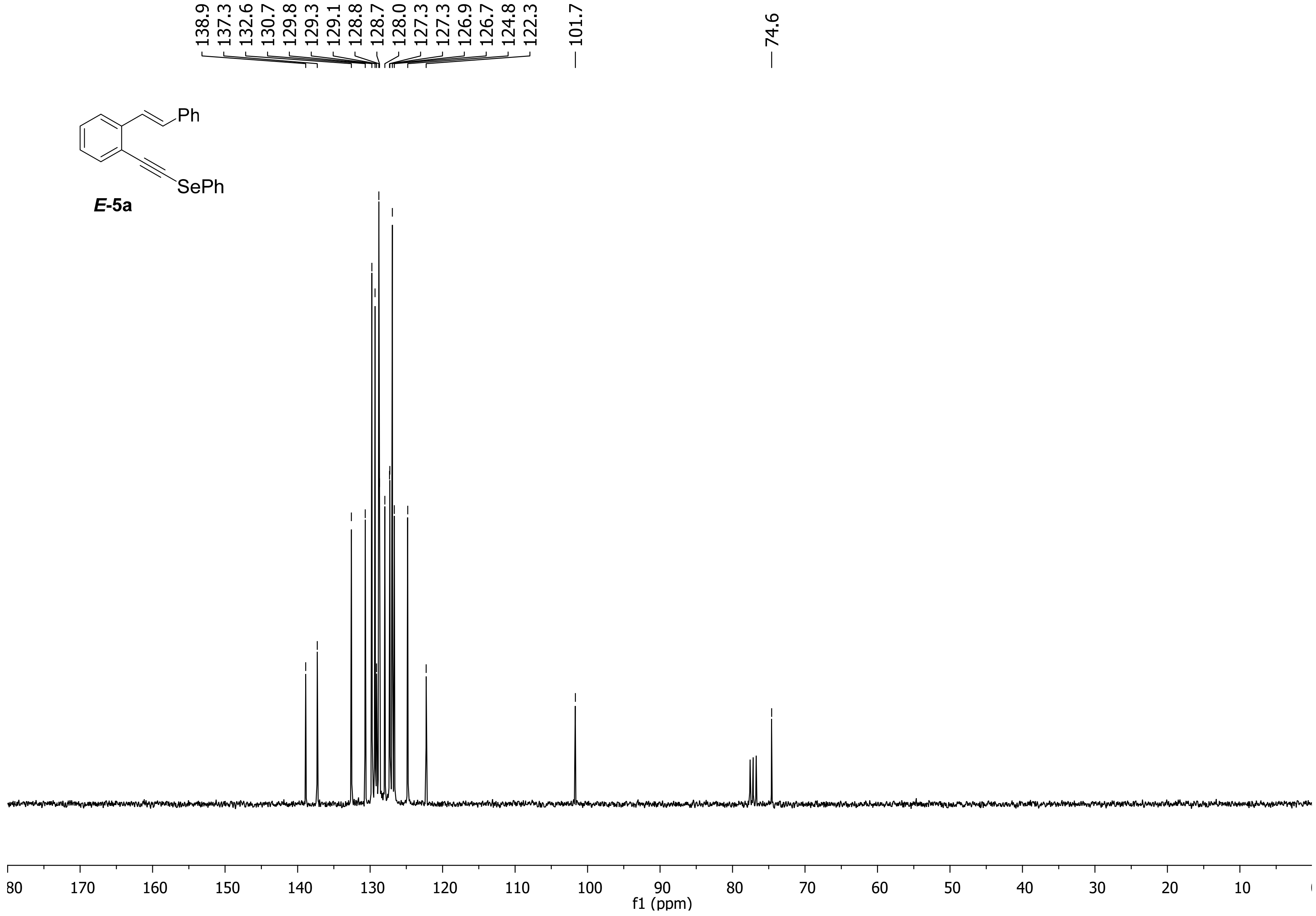

^{1}H NMR (300 MHz, $CDCl_3$)

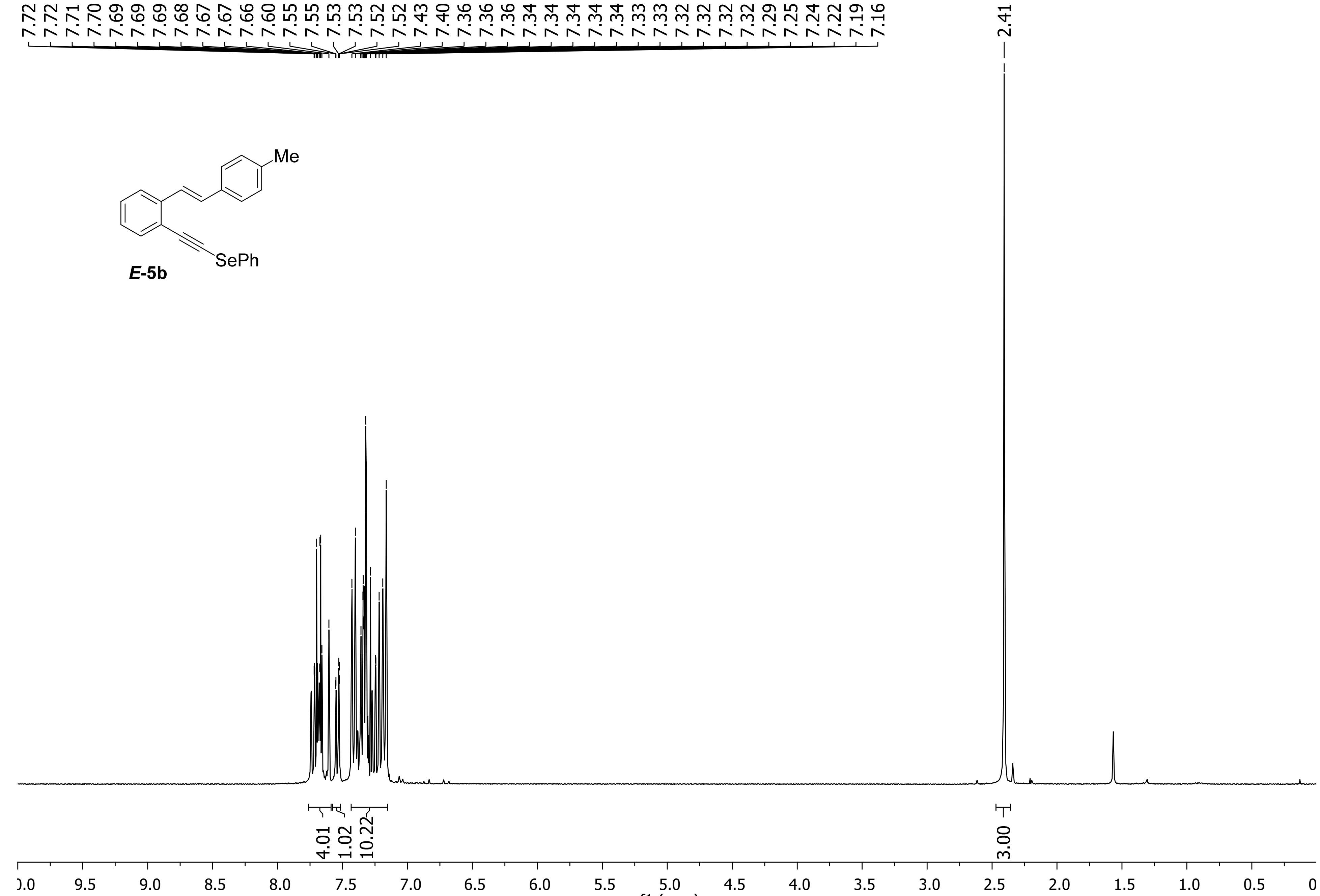

^{13}C NMR (75.4 MHz, $CDCl_3$)

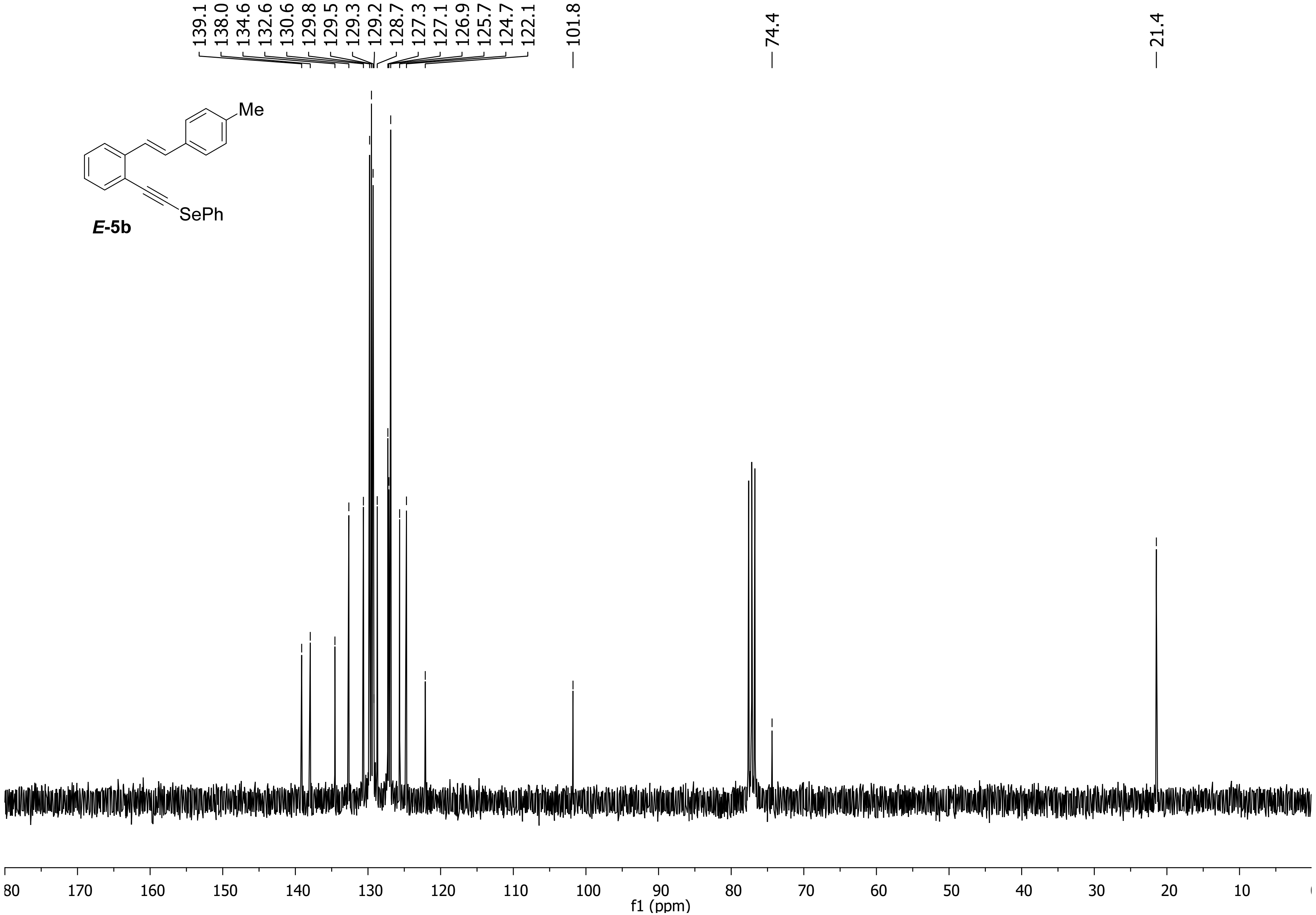

^{1}H NMR (300 MHz, $CDCl_3$)

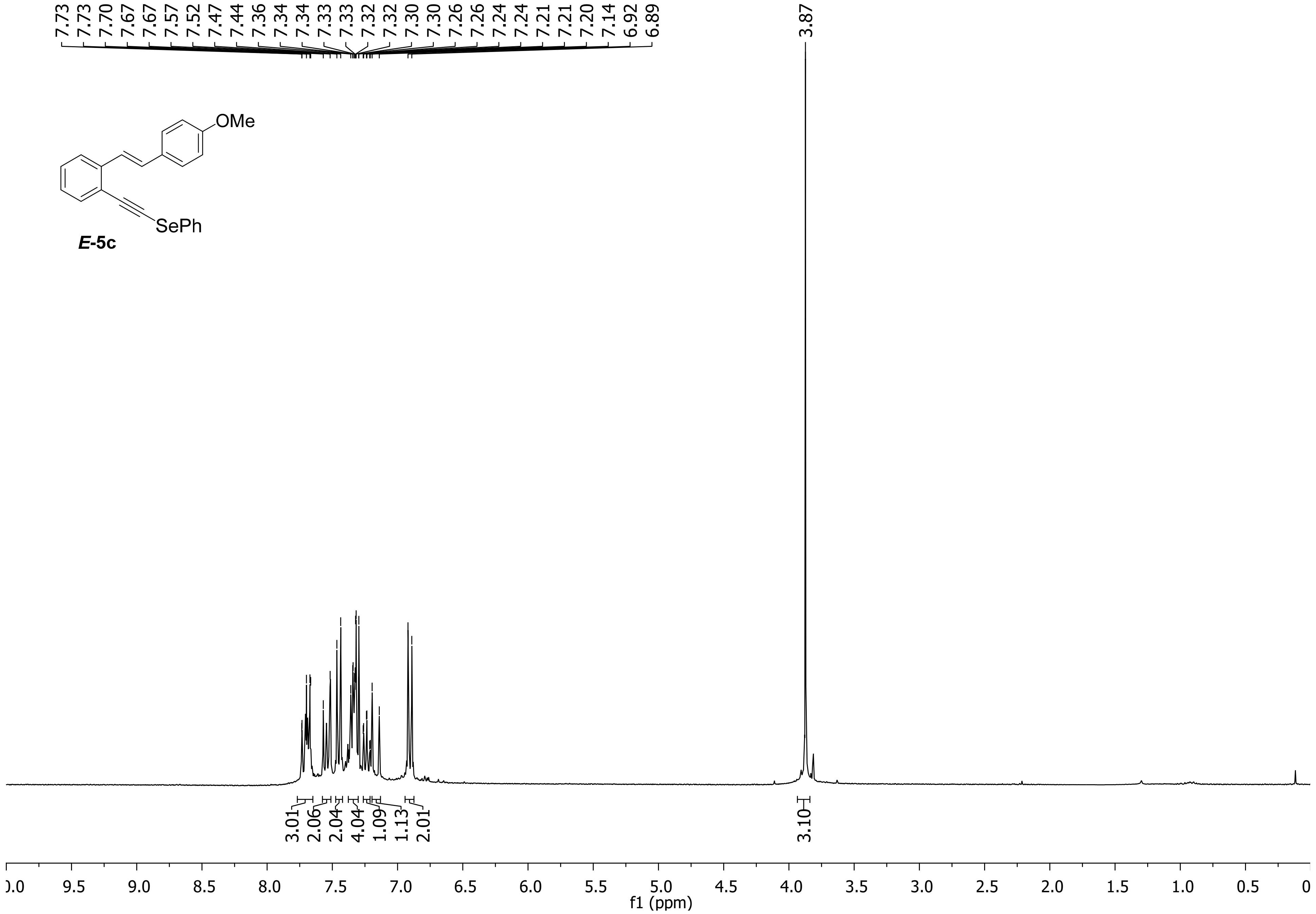

^{13}C NMR (75.4 MHz, $CDCl_3$)

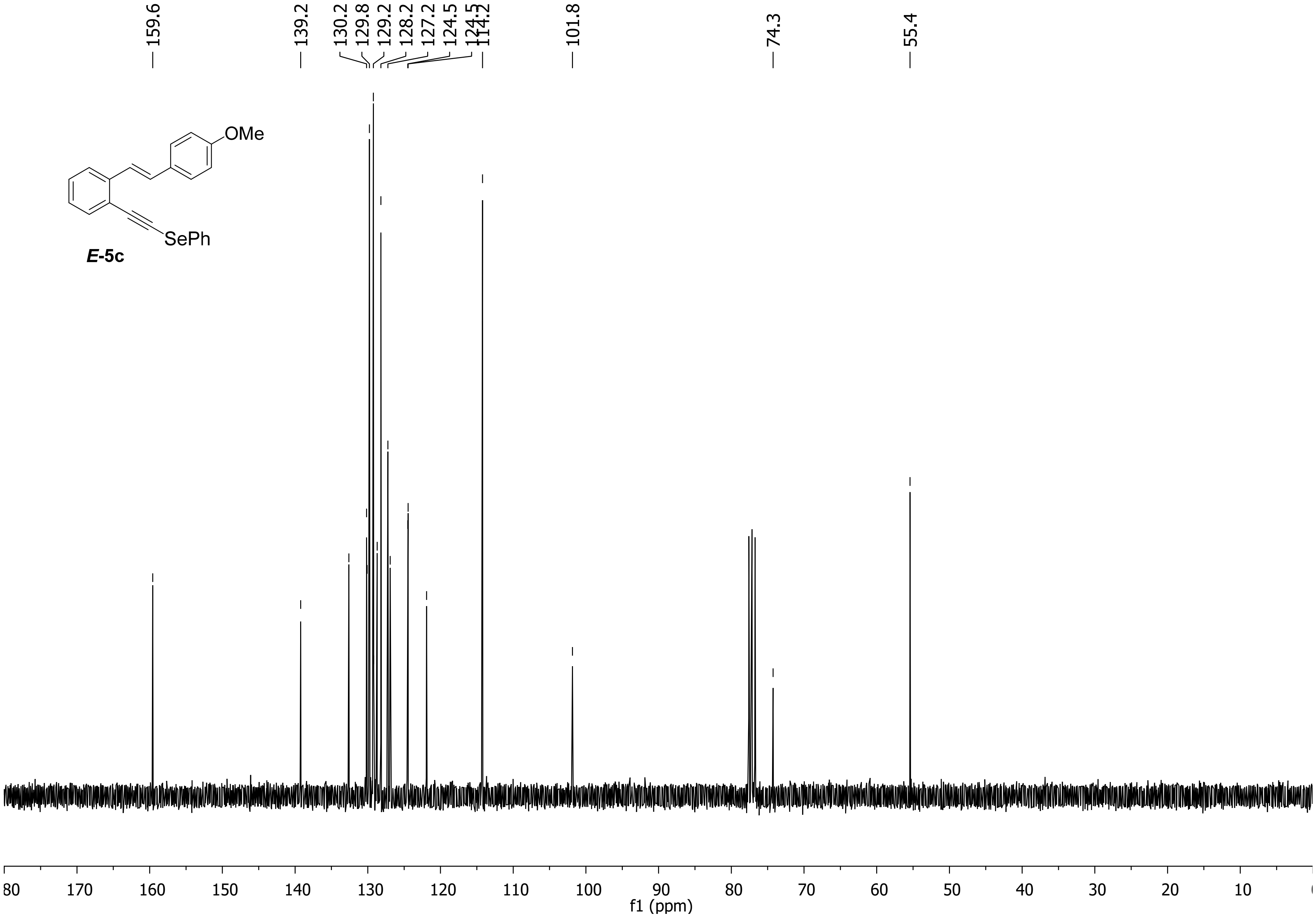

^{1}H NMR (300 MHz, $CDCl_3$)

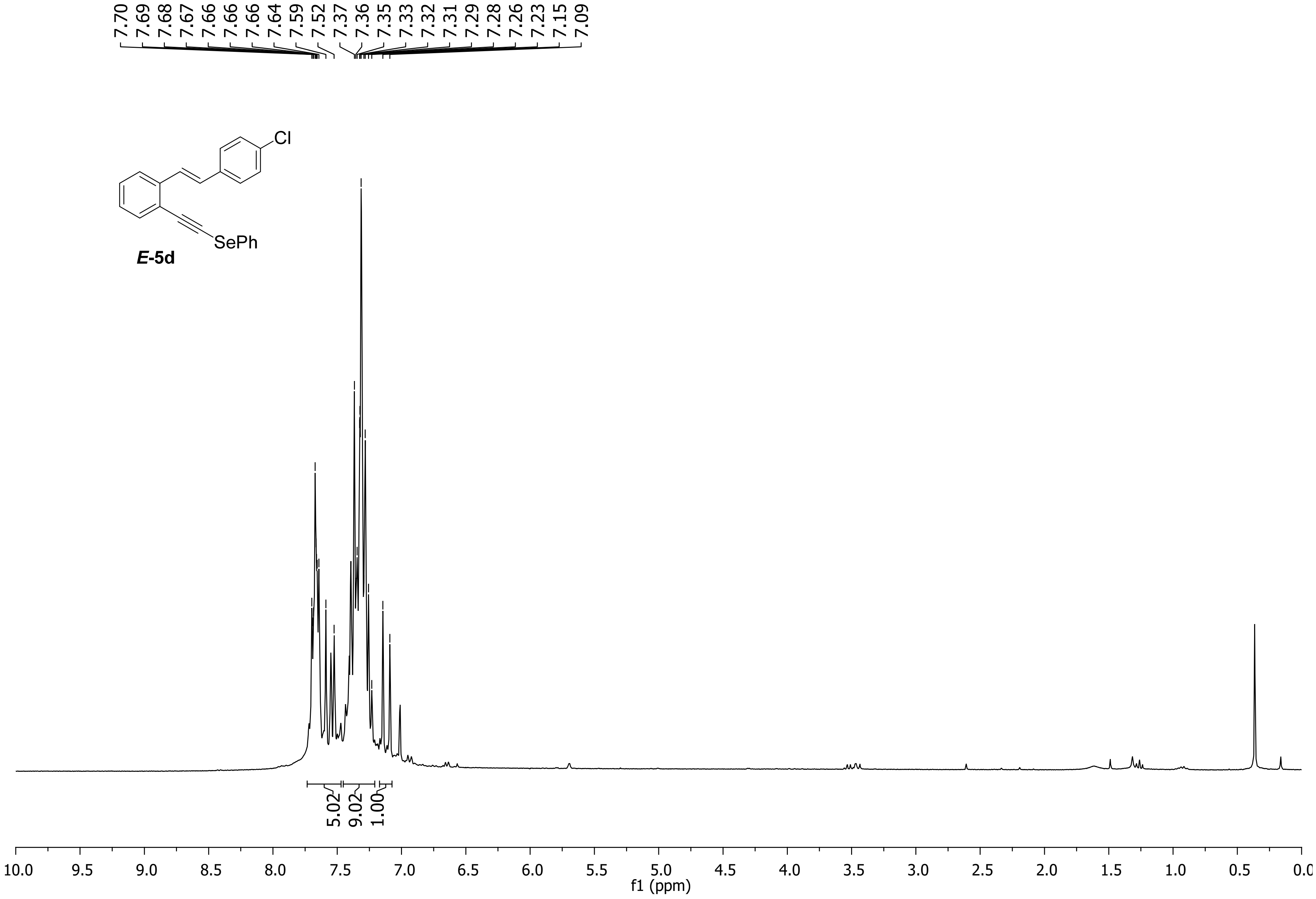

^{13}C NMR (75.4 MHz, $CDCl_3$)

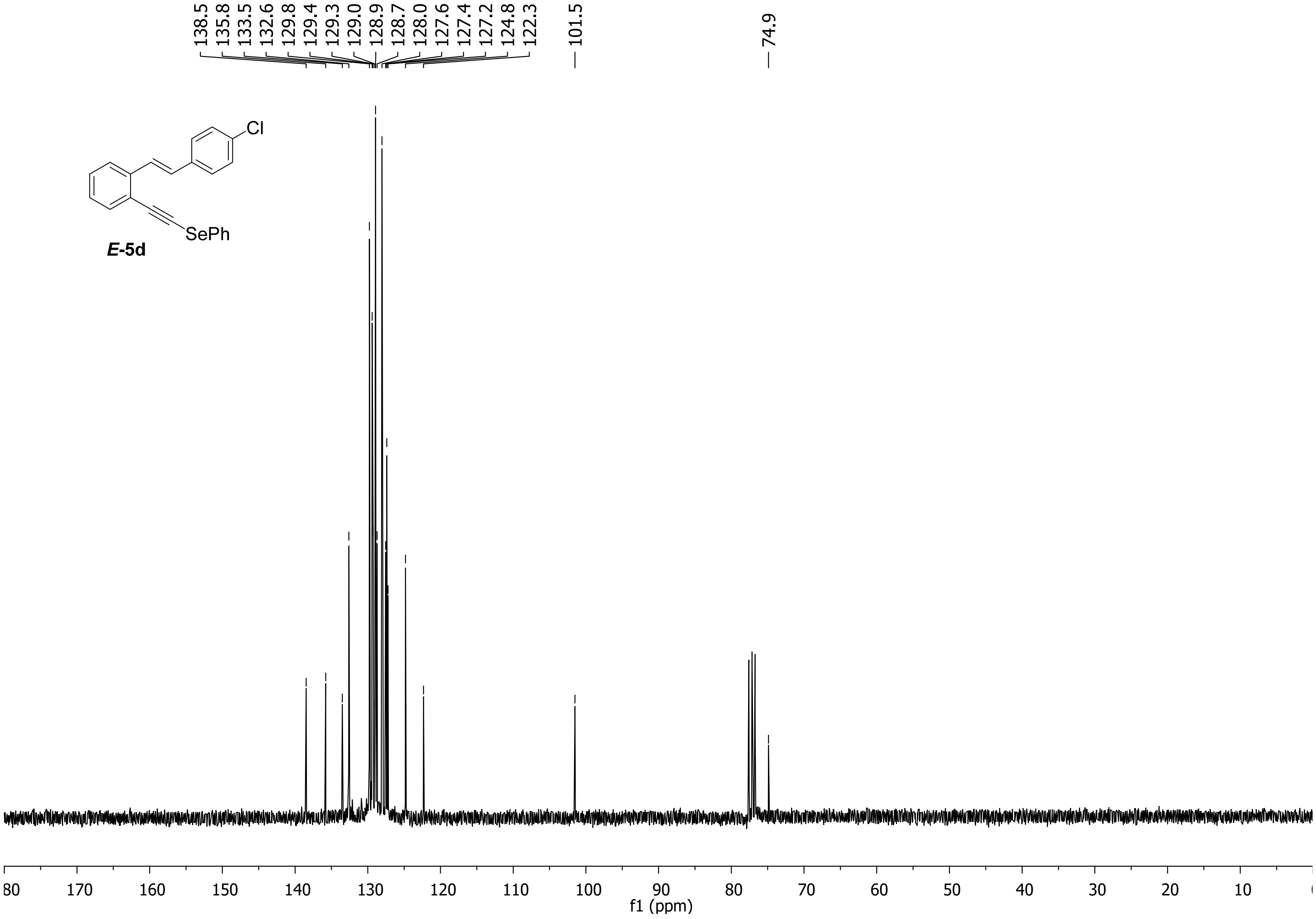

***E*-5d**

^1H NMR (300 MHz, $CDCl_3$)

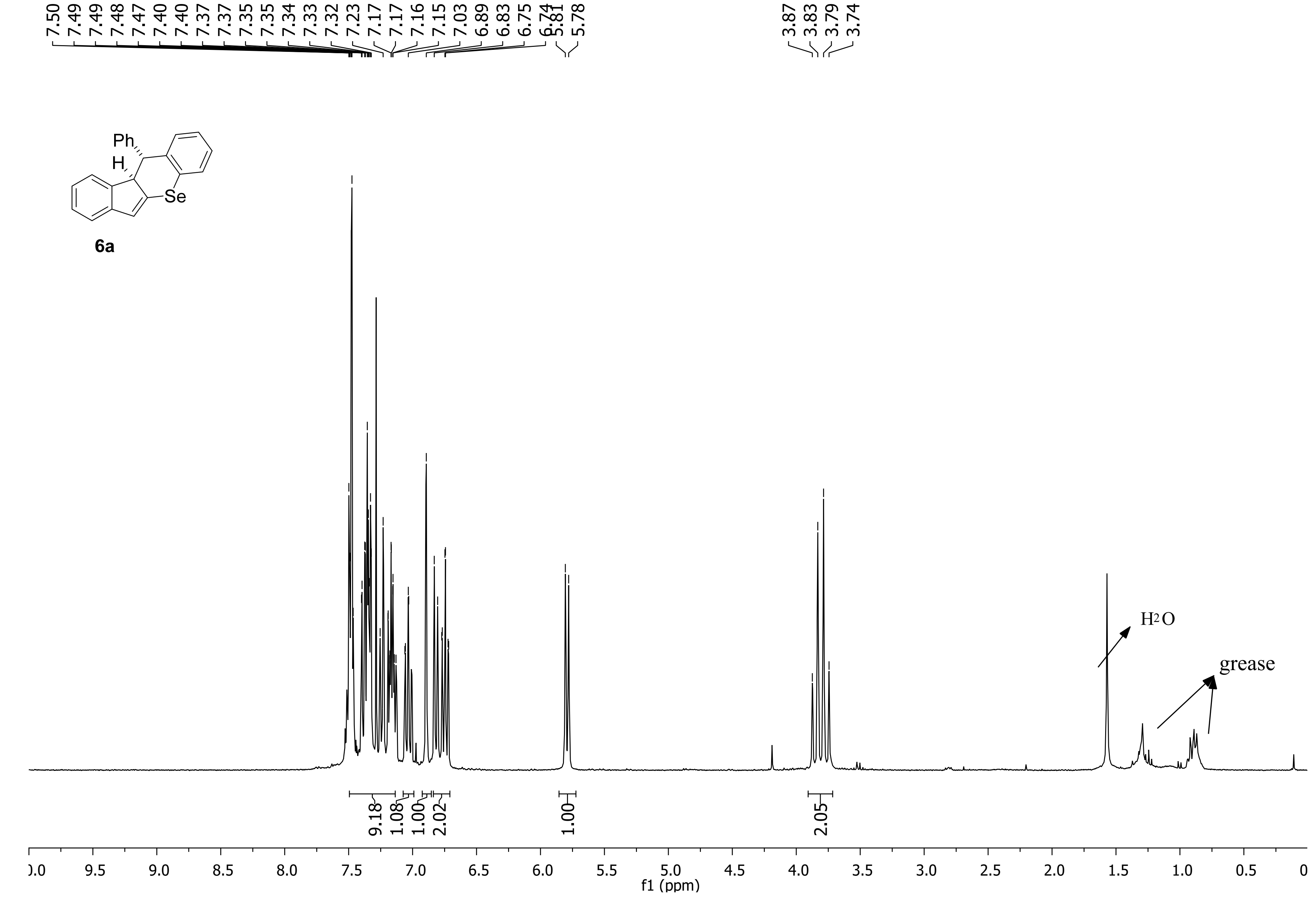

^{13}C NMR (75.4 MHz, $CDCl_3$)

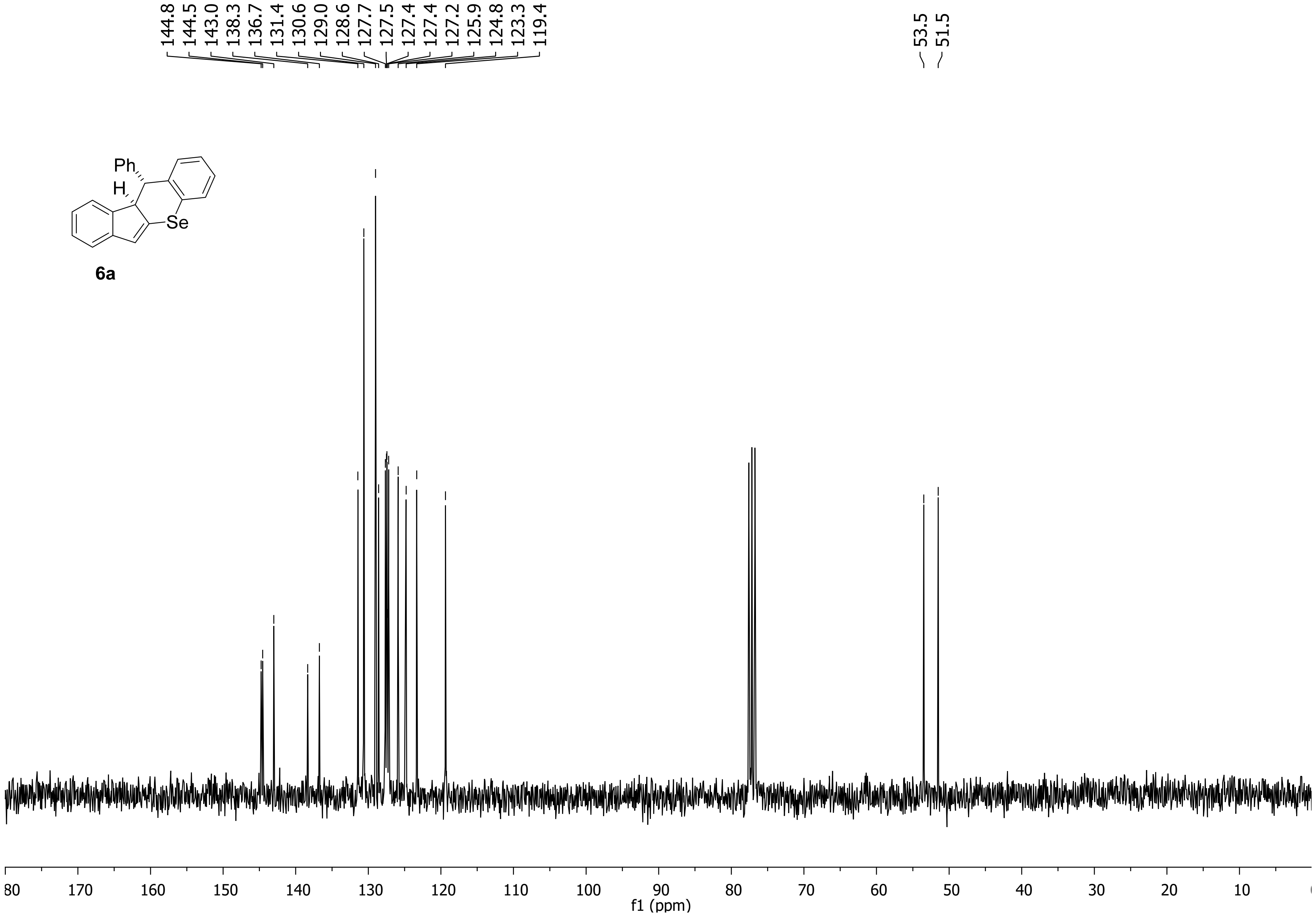

6a

^{1}H NMR (300 MHz, $CDCl_3$)

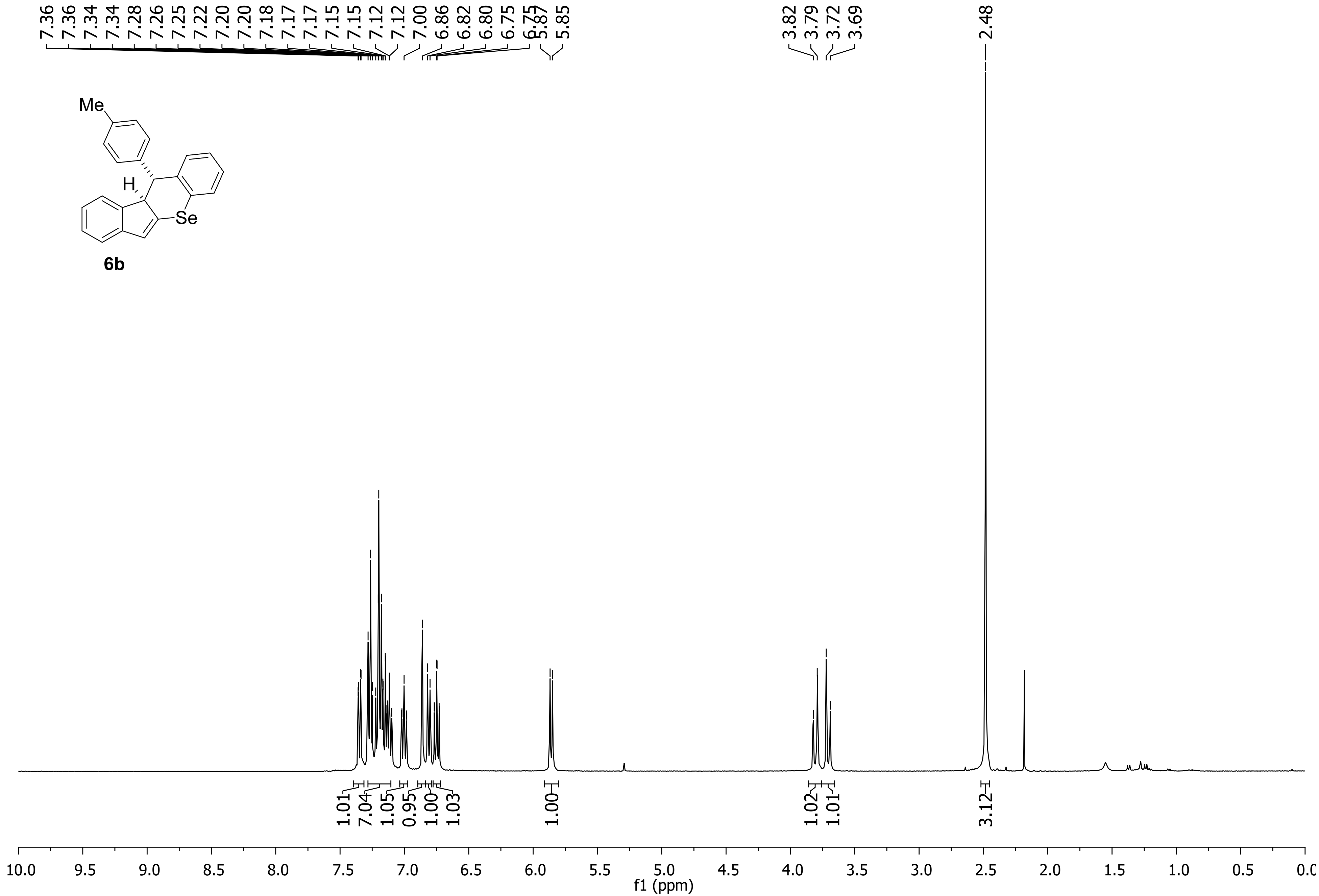

^{13}C NMR (75.4 MHz, $CDCl_3$)

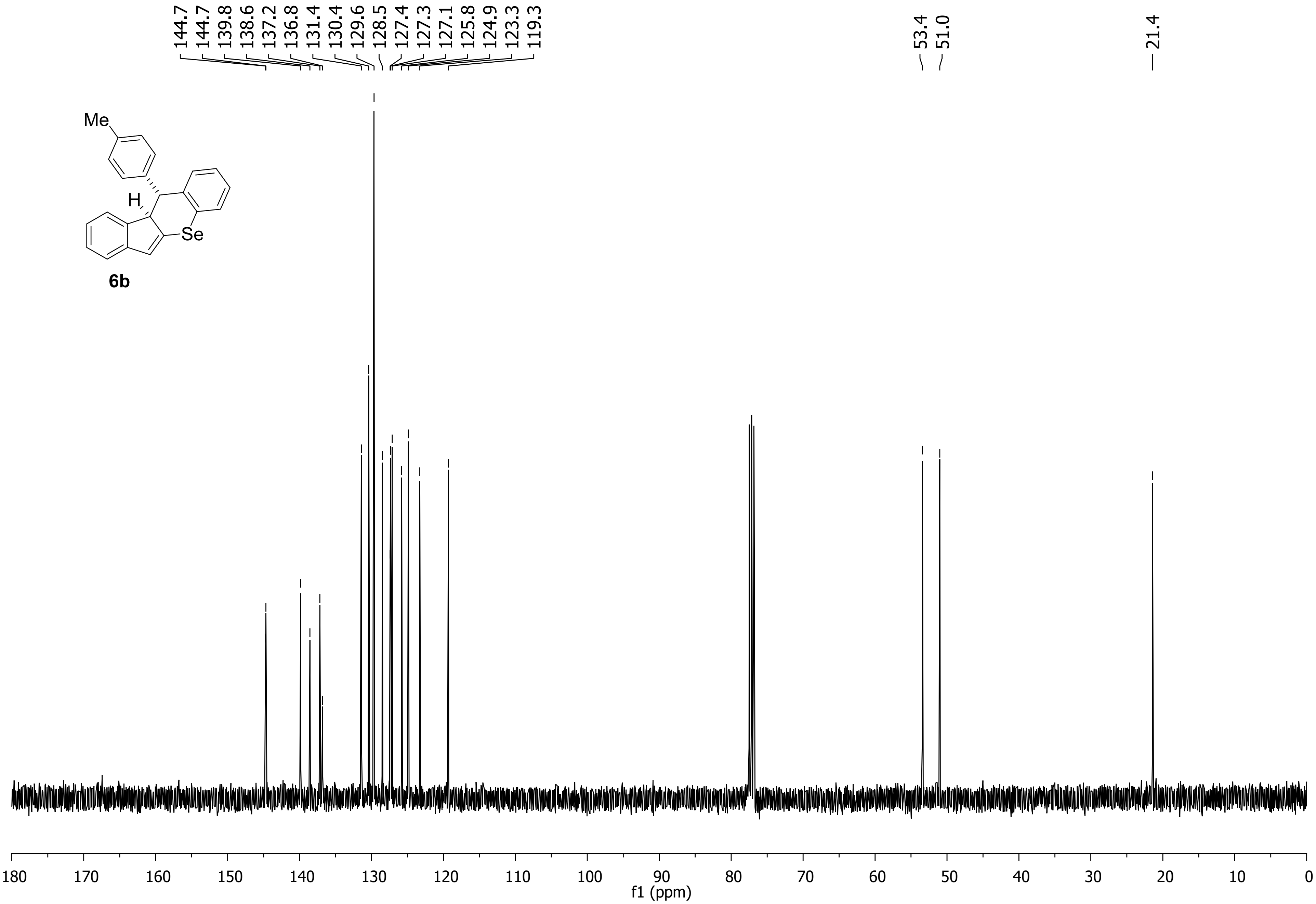

^{1}H NMR (300 MHz, $CDCl_3$)

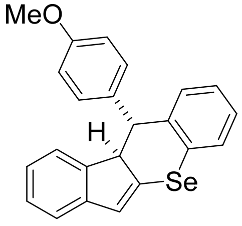

6c

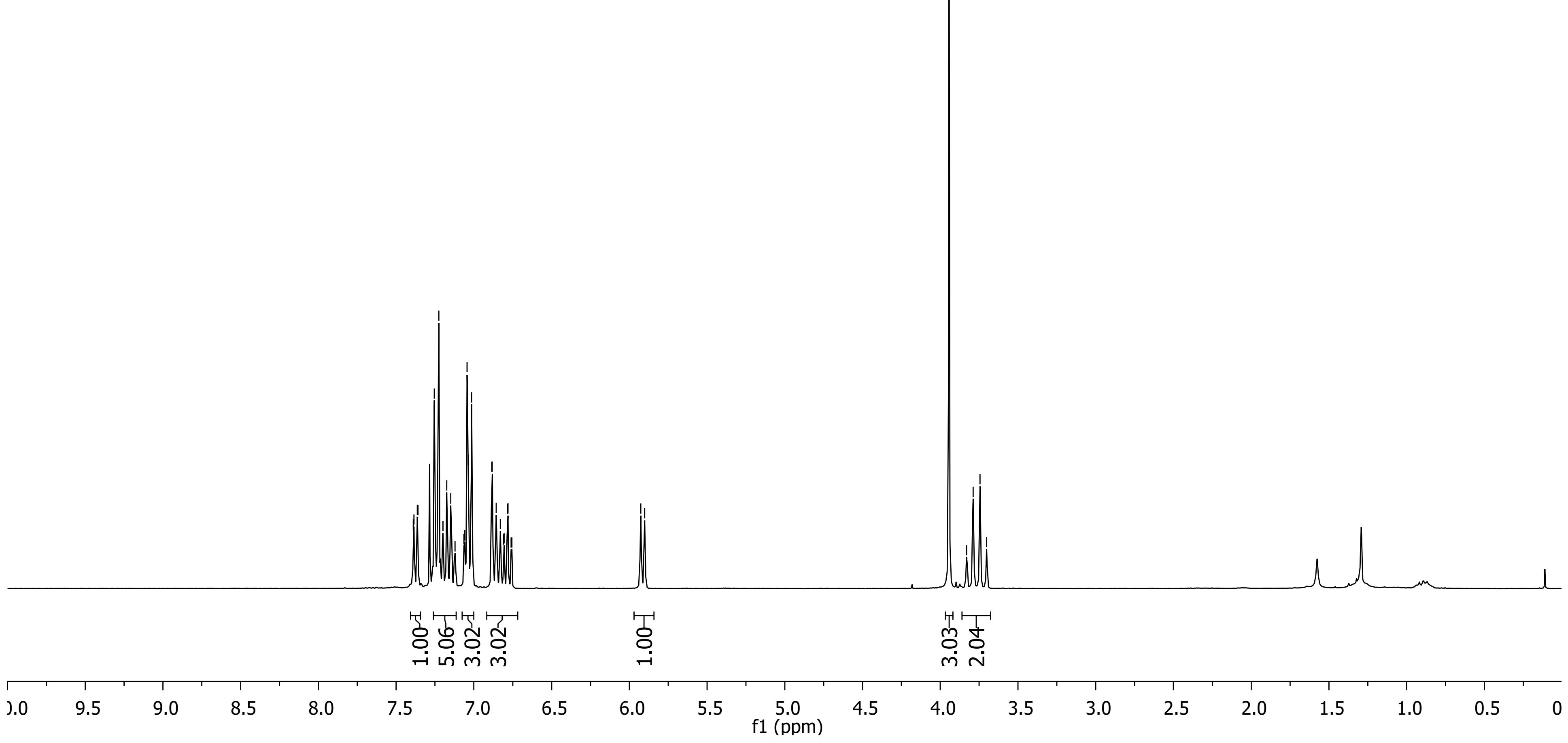

^{13}C NMR (75.4 MHz, $CDCl_3$)

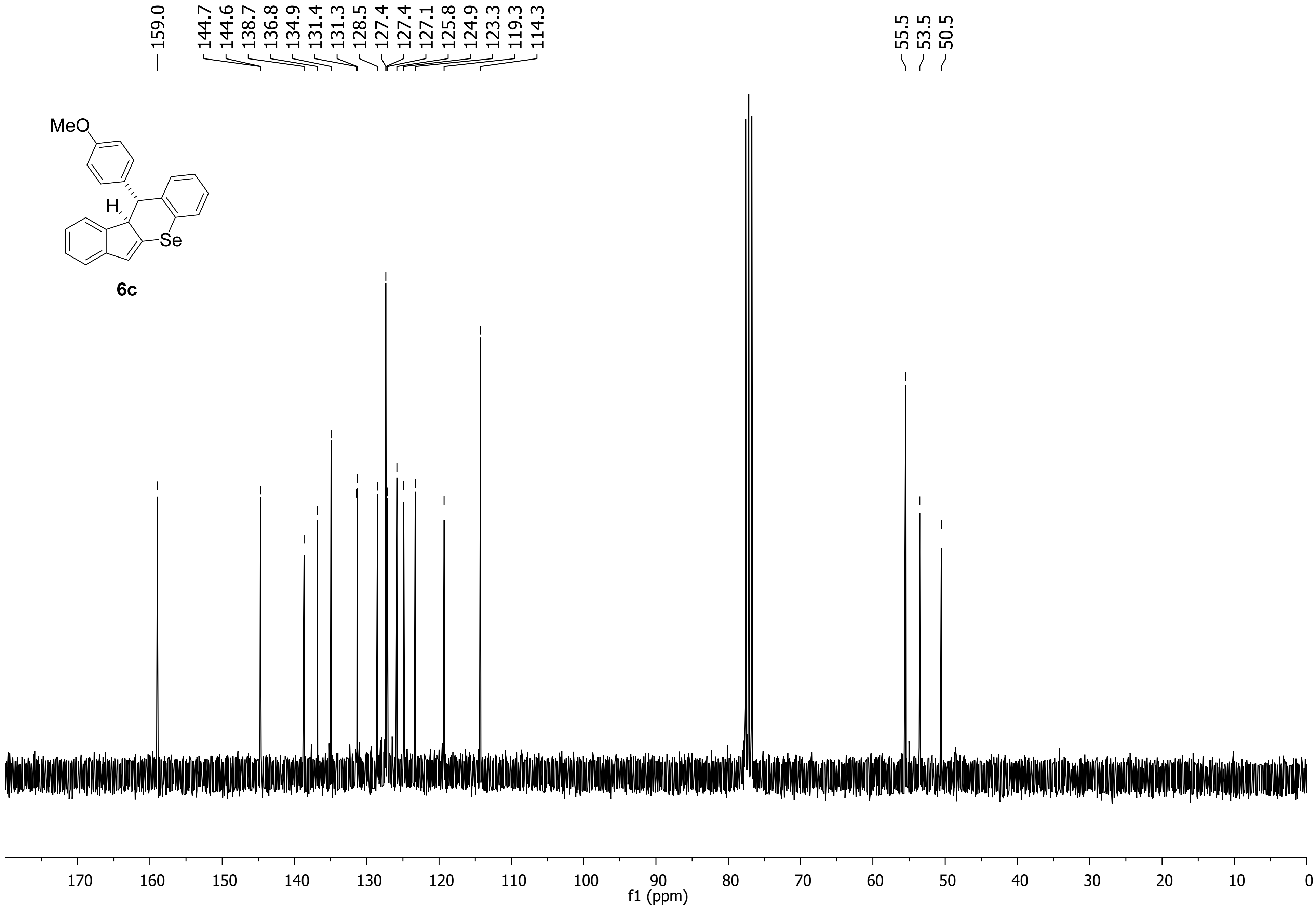

6c

^{1}H NMR (300 MHz, $CDCl_3$)

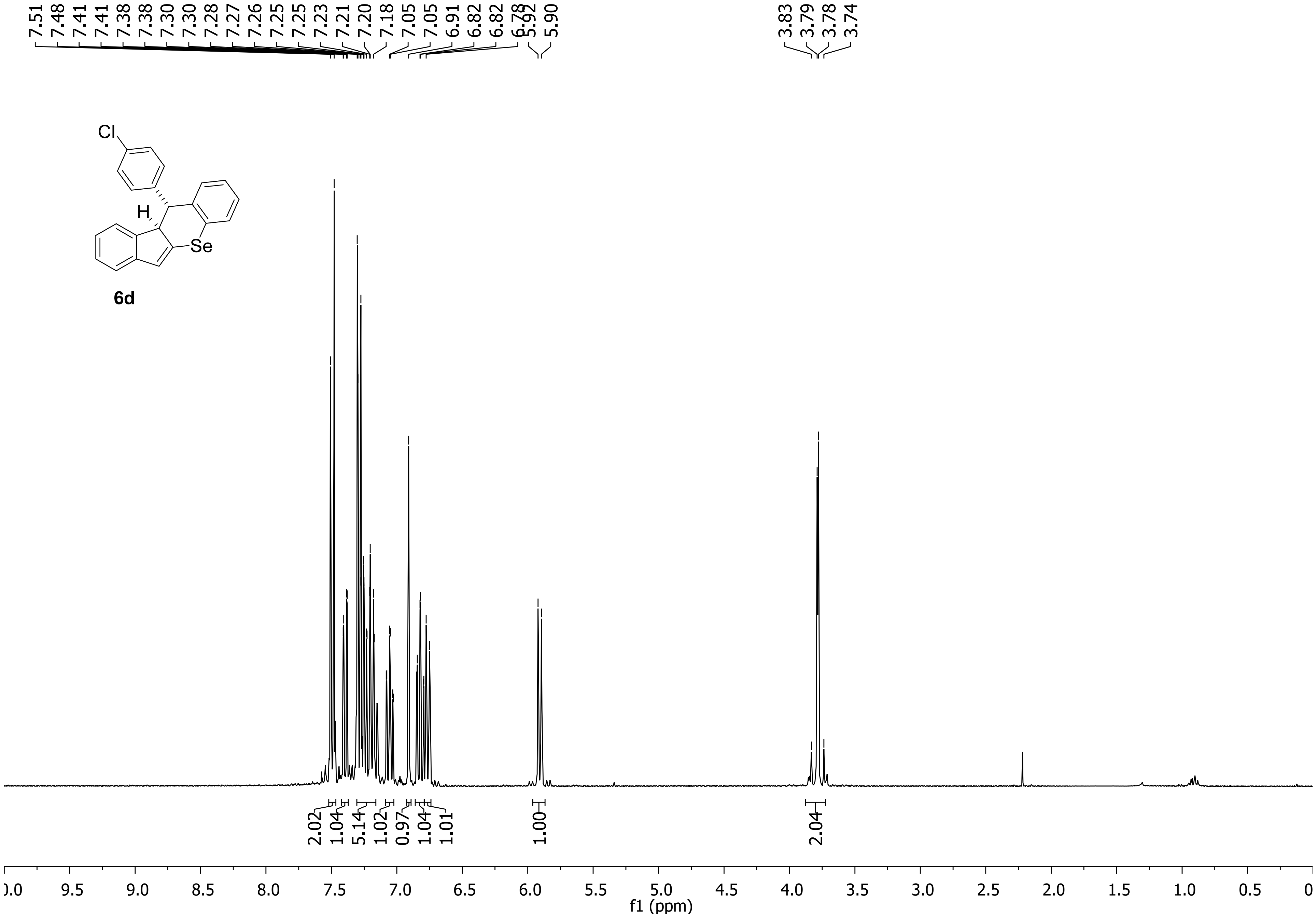

^{13}C NMR (75.4 MHz, $CDCl_3$)

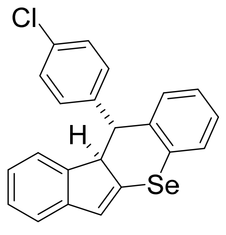

6d

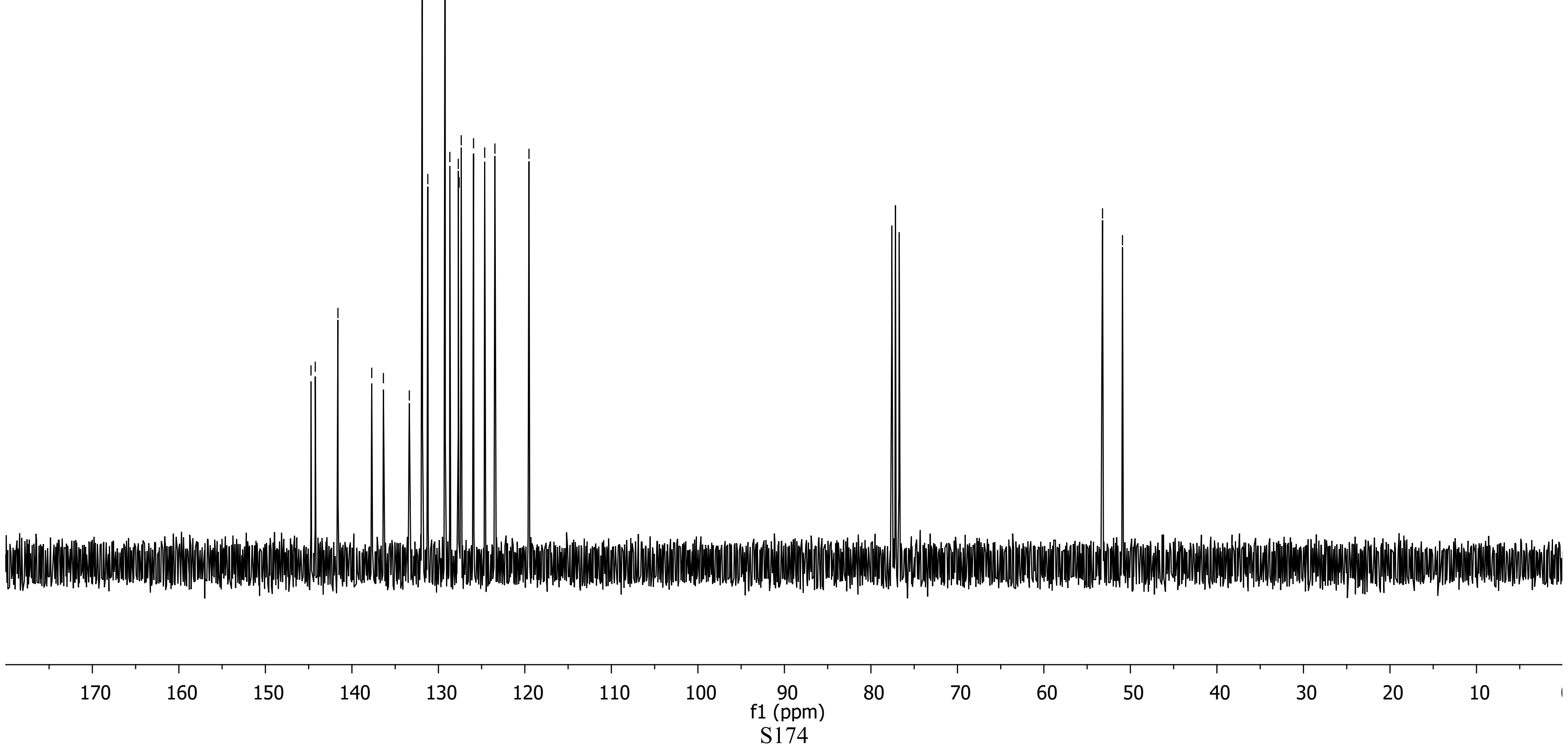

^{1}H NMR (300 MHz, $CDCl_3$)

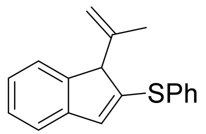

7u

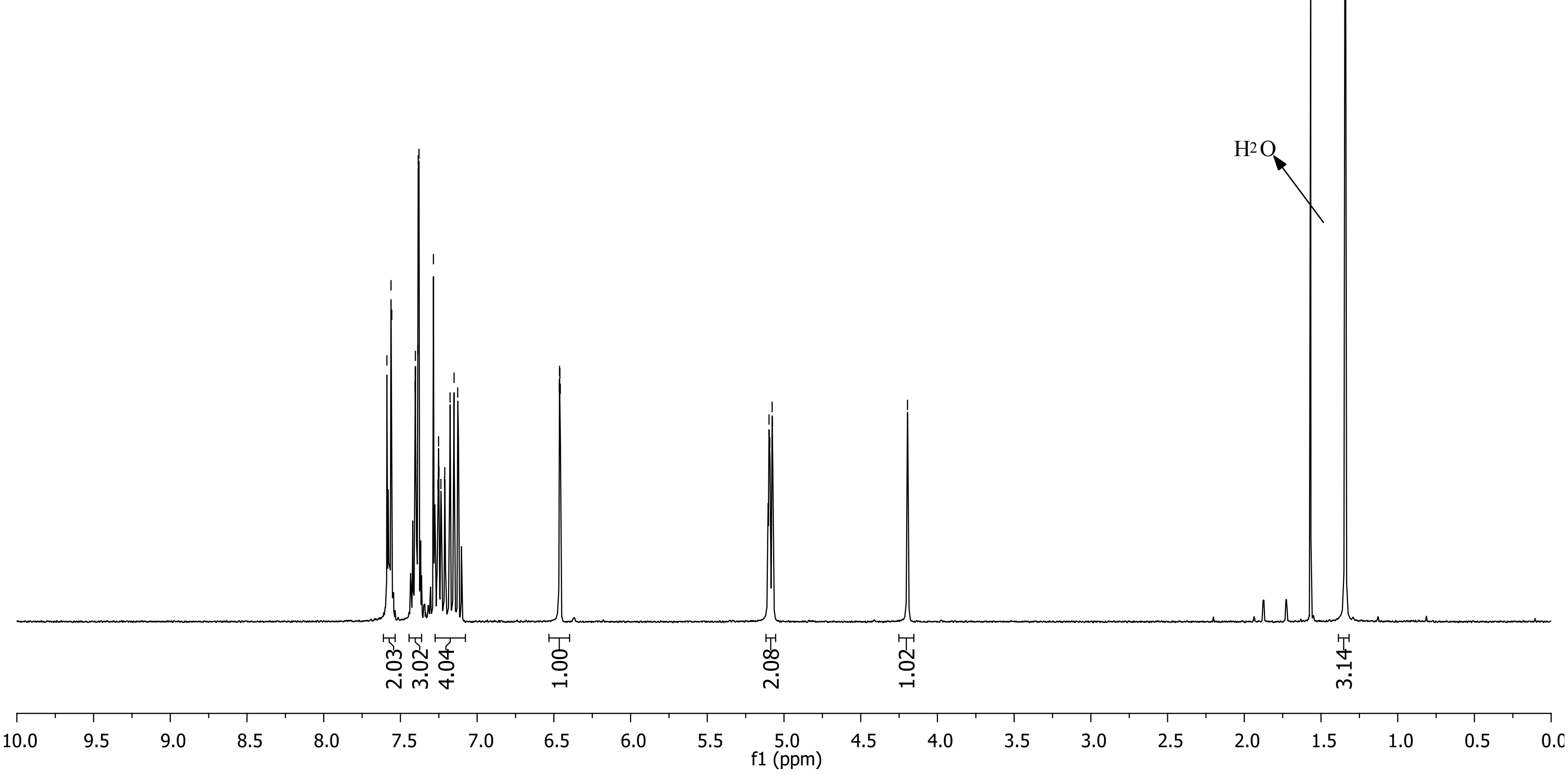

^{13}C NMR (75.4 MHz, $CDCl_3$)

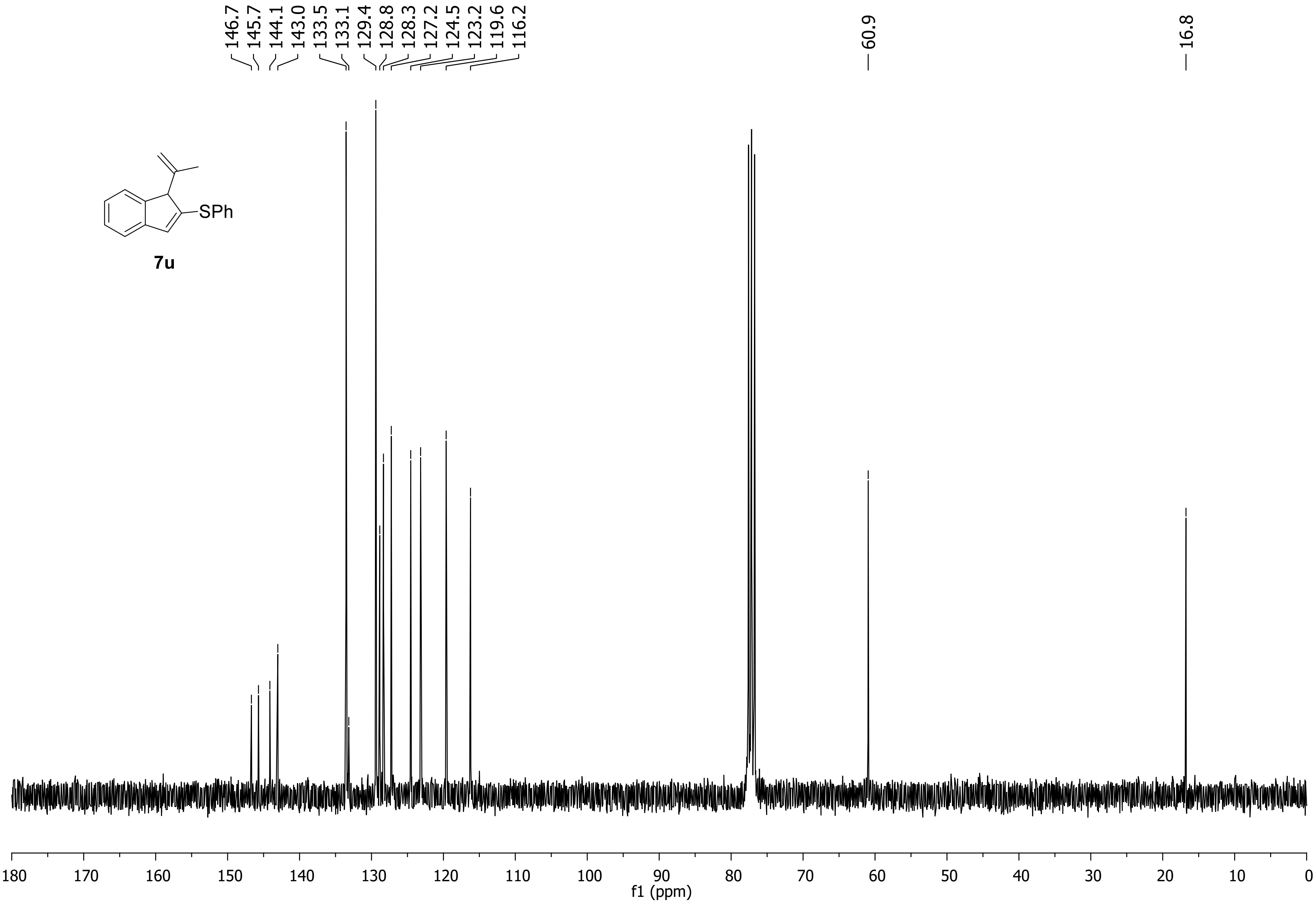

Supplement: Supplementary file 1 — ol2c03411_si_001.pdf [file ol2c03411_si_001.pdf]
