# Supplementary figures and images for: Gold(I) Catalysis Applied to the Stereoselective Synthesis of Indeno[2,1-b]thiochromene Derivatives and Seleno Analogues
Source: Org Lett. 2022 Oct 24;24(43):8077–82. doi: 10.1021/acs.orglett.2c03411 (PMC9641678; doi:10.1021/acs.orglett.2c03411)

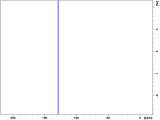

Supplement: Supplementary file 2 — ol2c03411_si_002.zip [file ol2c03411_si_002.zip › FID/2r/13C/pdata/1/thumb.png]

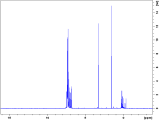

Supplement: Supplementary file 2 — ol2c03411_si_002.zip [file ol2c03411_si_002.zip › FID/2r/1H/pdata/1/thumb.png]

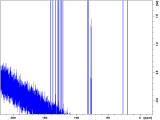

Supplement: Supplementary file 2 — ol2c03411_si_002.zip [file ol2c03411_si_002.zip › FID/2u/13C/pdata/1/thumb.png]

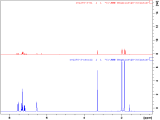

Supplement: Supplementary file 2 — ol2c03411_si_002.zip [file ol2c03411_si_002.zip › FID/2u/1H/pdata/1/thumb.png]

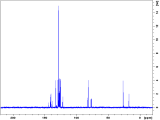

Supplement: Supplementary file 2 — ol2c03411_si_002.zip [file ol2c03411_si_002.zip › FID/2w/13C/pdata/1/thumb.png]

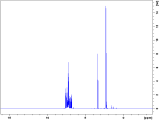

Supplement: Supplementary file 2 — ol2c03411_si_002.zip [file ol2c03411_si_002.zip › FID/2w/1H/pdata/1/thumb.png]

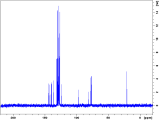

Supplement: Supplementary file 2 — ol2c03411_si_002.zip [file ol2c03411_si_002.zip › FID/3r/13C/pdata/1/thumb.png]

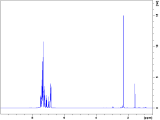

Supplement: Supplementary file 2 — ol2c03411_si_002.zip [file ol2c03411_si_002.zip › FID/3r/1H/pdata/1/thumb.png]

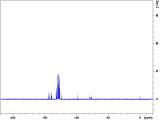

Supplement: Supplementary file 2 — ol2c03411_si_002.zip [file ol2c03411_si_002.zip › FID/3s/13C/pdata/1/thumb.png]

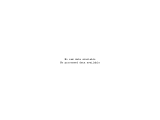

Supplement: Supplementary file 2 — ol2c03411_si_002.zip [file ol2c03411_si_002.zip › FID/3s/1H/pdata/1/thumb.png]
